# In vivo labeling–based proteomic analysis of early follicle oocytes and cisplatin-induced alterations in mice

**DOI:** 10.1038/s41467-026-76665-3

**Published:** 2026-08-15

**Authors:** Zhi-Yan Jiang, Meiyu Bi, Xuan Wu, Miaomiao Ban, Yuyao Lu, Jiangli Cao, Jie Li, Qilong Wang, Xin Zhu, Mo Li, Heng-Yu Fan, Bingteng Xie

**Affiliations:** 1 MOE Key Laboratory for Biosystems Homeostasis and Protection and Innovation Center for Cell Signaling Network, Life Sciences Institute, Zhejiang University, Hangzhou, China; 2 National Clinical Research Center for Obstetrics and Gynecology, State Key Laboratory of Female Fertility Promotion, Department of Obstetrics and Gynecology, Peking University Third Hospital, Beijing, China; 3 Key Laboratory of Assisted Reproduction (Peking University), Ministry of Education, Beijing, China; 4 Beijing Key Laboratory of Reproductive Endocrinology and Assisted Reproductive Technology, Beijing, China; 5 Key Laboratory of Molecular Medicine and Biological Diagnosis and Treatment (Ministry of Industry and Information Technology), School of Life Science, Beijing Institute of Technology, Beijing, China; 6 Assisted Reproduction Unit, Department of Obstetrics and Gynecology, Sir Run Run Shaw Hospital, School of Medicine, Zhejiang University, Hangzhou, China; 7 Center for Biomedical Research, Shaoxing Institute, Zhejiang University, Shaoxing, China

**Keywords:** Oogenesis, Cancer, DNA damage and repair

## Abstract

A systematic proteomic profile of oocytes from early-stage follicles, particularly primordial follicles, is critical to protect female reproductive capacity in the context of chemotherapy, yet progress has been hindered by the rarity of oocyte samples and technical challenges associated with oocyte isolation. In this study, we generated in vivo oocyte protein labeling APEX fluorescent mice. With these mice, we reconstructed the ovary in 3D, enabling precise quantification of follicles and identified 2772 proteins and 2878 gene transcripts in oocytes predominantly from primordial follicles. Proteomic shifts of short-time cisplatin treatment revealed that many altered proteins were involved in DNA damage repair and histone modification. Notably, simultaneous application of cisplatin and EZH2’s inhibitor, GSK126, relieved cisplatin-induced oocyte developmental defects. Our study provides a systematic proteomic characterization of oocytes predominantly from primordial follicles in female mice, and reveals dynamic proteome shifts in response to chemotherapeutic agents, laying the foundation for targeted fertility-preserving strategies.

## Abstract (web summary)

Using in situ APEX labeling, this study maps proteins and RNA in mouse oocytes from early ovarian follicles and shows how cisplatin alters these cells, identifying EZH2 inhibition as a potential strategy to lessen cisplatin-induced oocyte damage.

## Introduction

Currently, with the prevalence of chemotherapy in patients with malignancies, a rising concern is the preservation of ovarian function and reproductive capacity in female patients who desire offspring, which fundamentally depends on the reserve of primordial follicles^1^. A primordial follicle pool is established before birth and houses a woman’s lifelong supply of oocytes. The number of primordial follicles begins to decline from the fetal stage and continues to decrease with age, resulting in reduced fertility. However, for female patients with malignancy, chemotherapeutic treatment severely impairs their quality of life, particularly causing infertility. Certain chemotherapeutic agents have been reported to directly induce oocyte death in immature follicles, leading to irreversible reproductive damage^2^. Thus, elucidating the mechanisms underlying the action of chemotherapeutic compounds on the ovary, especially on primordial follicular oocytes, is essential for developing efficient and targeted pharmacological therapies that could protect and prolong female fertility.

Cisplatin, the first platinum compound approved by the U.S. Food and Drug Administration (FDA) for cancer treatment, has been widely used to treat various solid tumors, including ovarian, breast, and thyroid cancers^3–6^. As a DNA crosslinking agent, cisplatin induces apoptosis by generating interstrand DNA adducts that trigger DNA repair mechanisms and impair cell division, ultimately leading to oocyte death^7–10^. The expression of many proteins has been reported to be altered following short-term cisplatin treatment, indicating a rapid cellular response to cisplatin exposure^11,12^. In addition, cisplatin damages oocyte mitochondria, particularly in immature oocytes, thereby reducing oocyte quality and compromising embryonic developmental potential after fertilization^13,14^. Therefore, unrevealing a comprehensive profile of protein expression alterations of immature oocytes will be critical for elucidating the pathogenesis of chemotherapy-induced ovarian failure.

While the integral ovarian proteome has provided a protein expression atlas encompassing different cell types within the ovary, the specific protein expression profiles of individual cell types remain inaccessible, despite their importance given the high heterogeneity of protein expression across cell types. With advances in mass spectrometry, the proteomes of mouse oocytes at the fully grown oocyte (FGO) stage and metaphase Ⅱ (MⅡ) stage have been identified in vitro^15–17^. However, the proteome of primordial follicular oocytes has not yet been systematically profiled, owing to the low protein abundance of oocytes in primordial follicles and the technical challenges of isolating oocytes from primordial follicles. Traditional proteomics often relies on isolating specific cell types from complex tissues through lengthy processes such as digestion, labeling, and sorting. Aside from being inefficient, these methods can introduce artificial effects, including the loss of cellular context and disruption of protein interactions, which are crucial for understanding the roles of proteins in their native environment. Engineered ascorbate peroxidase (APEX)-mediated reactions, an emerging protein labeling technology, enable the biotinylation of surrounding proteins and have been applied to delineate subcellular proteomes and study protein-protein interactions at the cellular level^18,19^. Recently, we constructed in vivo protein-labeled APEX fluorescent mice, in which the APEX2 enzyme is genetically expressed in the target cell type by mating with different Cre driver mice. Upon triggering the reaction, it covalently tags proximal proteins within the intact, living tissue microenvironment, before any tissue disruption occurs. Following tissue collection and cell lysis, the labeled proteins are enriched and identified, providing a more accurate representation of the proteins as they exist within the tissue microenvironment. This APEX mouse model is a powerful tool for in situ profiling of the primordial follicular oocyte proteome.

In this study, we established an APEX biotinylation system for the intact mouse ovary. Using EGFP-tagged APEX signals, we generated a 3D reconstruction of the entire ovary, enabling accurate quantification of follicles at different developmental stages and visualization of all granulosa cells within individual follicles across stage transitions. Importantly, we applied APEX2-driven biotinylation to capture the proteome and transcriptome of early follicle oocytes, particularly at the primordial follicle stage. Comparative proteomic analysis before and after short-term cisplatin treatment revealed a set of differentially expressed proteins. Among them, the effect of EZH2, a histone methyltransferase responsible for H3K27 methylation, was investigated. Co-administration of cisplatin with an EZH2 inhibitor alleviated cisplatin-induced oocyte damage, highlighting the potential of this study for optimizing cancer treatment regimens and developing strategies to protect female fertility.

## Results

### Targeting active APEX2 to in vivo follicle-enclosed oocytes

**Fig. 1:**
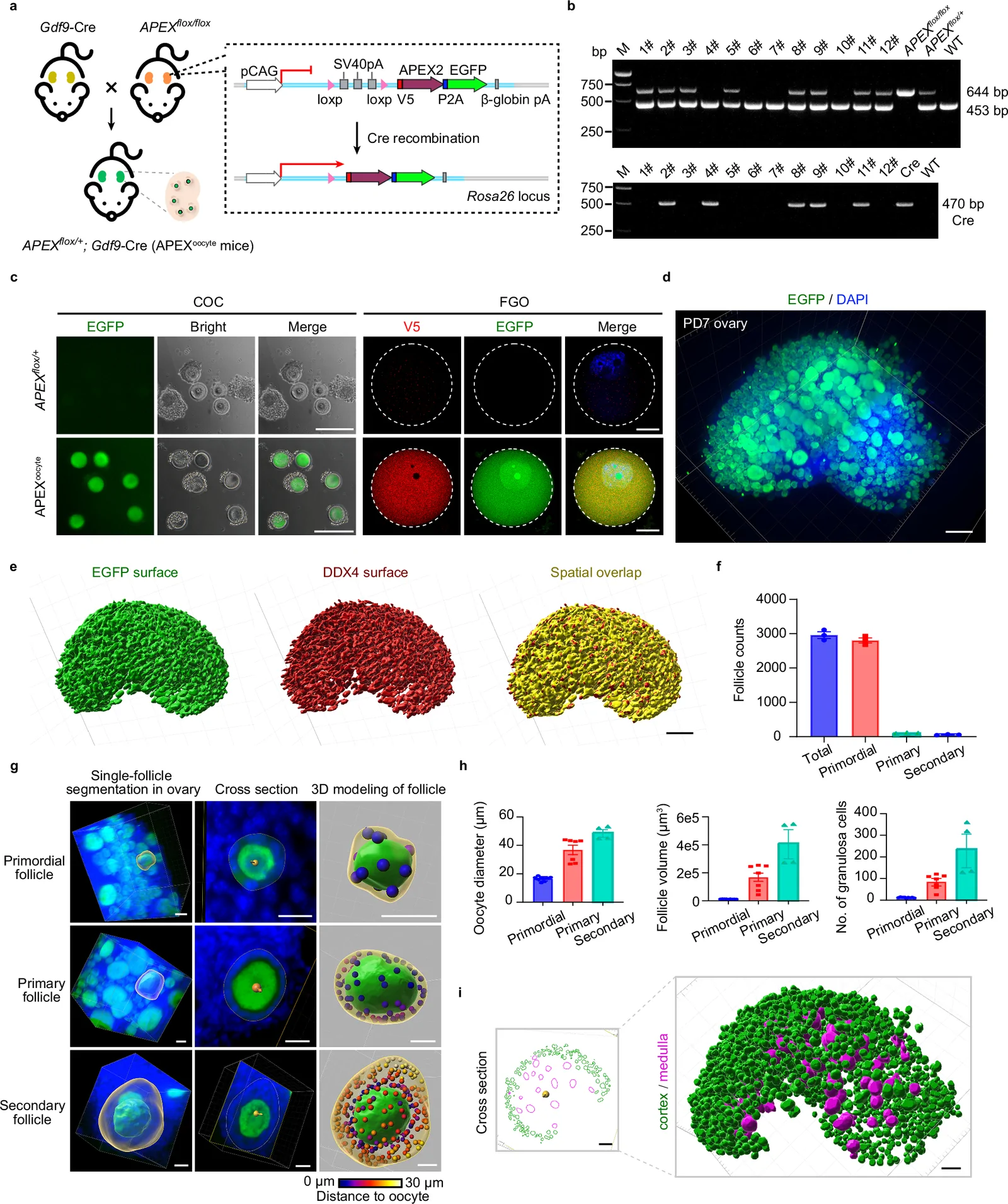
Targeting active APEX2 to in vivo follicle-enclosed oocytes. **a** Generation strategy for APEX^oocyte^ mice with oocyte-specific conditional expression of APEX2 from the primordial follicle stage. APEX2 carries an N-terminal V5 tag and is linked to EGFP via a P2A sequence. Oocyte-specific APEX2 expression is detected in the offspring (*APEX*^*flox/+*^*;Gdf9-*Cre: APEX^oocyte^ mice) of *APEX2*-transgenic mice crossed with *Gdf9-*Cre mice. **b** PCR genotyping confirming offspring with APEX and Cre alleles. Bands corresponding to APEX floxed allele and Cre transgene are shown. The experiment was independently repeated three times with similar results. WT, wild type control. bp, base pair. **c** Oocyte-specific expression and intracellular distribution of APEX2. EGFP fluorescence in cumulus-oocyte complexes (COCs) and IF imaging of V5 and EGFP in fully grown oocytes (FGOs) from *APEX*^*flox/+*^ and APEX^oocyte^ females. DNA was stained with DAPI. Representative images from three independent experiments with similar results are shown. Scale bars, COC: 200 μm; FGO: 20 μm. **d** Whole-ovary 3D volumetric imaging of a tissue-cleared PD7 APEX^oocyte^ ovary based on intrinsic EGFP fluorescence (oocytes) and DAPI (nuclei). Representative images from three independent experiments with similar results are shown. Scale bars, 100 μm. **e** Surface rendering of EGFP and DDX4 signals in a PD7 ovary. EGFP-positive oocytes are shown in green, DDX4-positive germ cells in red, and overlapping signals in yellow. Scale bars, 100 μm. **f** Quantification of follicles at different developmental stages. *n* = 3 biologically independent ovaries from 3 mice. Mean ± SEM. **g** Single-follicle segmentation and structural modeling of primordial, primary, and secondary follicles. Oocytes are shown in green, and granulosa cells are colored from blue to yellow according to increasing distance from the central oocyte. Scale bars, 20 μm. **h** Quantification of oocyte diameter, follicle volume, and granulosa cell number per follicle. Mean ± SEM. *n* = 9, 7, and 4 for primordial, primary, and secondary follicle groups, respectively. **i** 3D reconstruction of ovarian cortex and medulla regions in APEX^oocyte^ mice with a representative cross-section view. Scale bars, 100 μm.

To enable oocyte-specific in situ expression of APEX2 in mice, we employed Cre-inducible APEX2 reporter mice, in which an APEX2 transgene expression cassette was inserted into the *Rosa26* locus, allowing APEX2-expressing cells to be labeled by EGFP fluorescence. The transgene was driven by a ubiquitous CAG promoter and interrupted by a *loxP*-stop (3× polyA signal)-*loxP* (LSL) cassette (Fig. 1a). Growth/differentiation factor-9 (GDF9), a paracrine protein specifically produced by oocytes, is expressed from the primordial follicle stage with detectable levels from approximately postnatal day (PD) 3, and primordial follicles still constitute the majority of the ovarian follicle pool around PD7 (Supplementary Fig. 1a–d)^20–26^. Herein, the APEX mice were crossed with *Gdf9-*Cre mice to achieve oocyte-specific expression of APEX2 from the primordial follicle stage. PCR genotyping confirmed the presence of the *APEX*^*flox/+*^*; Gdf9-*Cre genotype in representative offspring (mice #2, #8, #9, and #11), hereafter referred to as APEX^oocyte^ mice (Fig. 1b and Supplementary Fig. 1e). APEX^oocyte^ females were fertile and exhibited normal ovarian morphology at 4 weeks of age (Supplementary Fig. 1f, g). Following intraperitoneal injection of pregnant mare serum gonadotropin (PMSG), fully grown oocytes (FGOs) retrieved from APEX^oocyte^ females underwent normal meiotic maturation, as evidenced by unaltered rates of germinal vesicle breakdown (GVBD) and first polar body extrusion (PBE) (Supplementary Fig. 1h). Sequentially treated with PMSG and human chorionic gonadotropin (hCG), APEX^oocyte^ females showed normal ovulation, yielding morphologically healthy MⅡ oocytes (Supplementary Fig. 1i, j). Immunofluorescence staining (IF) revealed that APEX2 was specifically expressed in FGOs within cumulus granulosa cell-oocyte complexes (COCs), but not in surrounding granulosa cells. APEX2 was distributed in both nucleus and cytoplasm, as evidenced by the V5 signal dispersing throughout the entire oocyte (Fig. 1c). Thus, we successfully constructed APEX^oocyte^ mice with oocyte-specific expression of APEX2 for subsequent in vivo imaging and protein labeling.

Currently, ovarian condition assessment often relies on two-dimensional (2D) imaging of tissue sections, which limits accuracy because the native 3D architecture is not preserved. Although several studies have reported 3D ovarian imaging using immunostaining combined with tissue clearing, these approaches typically require antibody labeling and lengthy processing^27–34^. In the present study, by exploiting EGFP expression in mouse oocytes together with tissue clearing and volumetric imaging, we generated a 3D reconstruction of an entire ovary from APEX^oocyte^ mice (Fig. 1d and Supplementary Fig. 1k). To validate the fidelity of EGFP-based oocyte labeling, ovaries were co-stained with the germ cell marker DEAD-box helicase 4 (DDX4). Nearly all DDX4-positive oocytes exhibited EGFP signals, confirming that *Gdf9*-driven APEX2-EGFP expression labels the vast majority of oocytes at this stage (Fig. 1e). Subsequent 3D reconstruction and quantitative analysis revealed that the volume of the PD7 ovary was 1.94 ± 0.23 × 10^8^ μm^3^ and contained 2959 ± 123 oocytes. Among these, 2803 ± 102 oocytes were enclosed within primordial follicles, whereas 100 ± 10 and 55 ± 11 oocytes were present in primary and secondary follicles, respectively (Fig. 1f). These values are consistent with previously reported follicle counts in PD7 mouse ovaries^35–38^. To obtain accurate structural information across follicle stages, we performed 3D modeling of individual follicles at different developmental stages (Fig. 1g). In primordial follicles, the oocyte diameter was 16.50 ± 1.49 μm. At this stage, pre-granulosa cells intercalated between adjacent oocytes (Supplementary Fig. 1l), forming primordial follicles with a volume of 1.31 ± 0.06 × 10^4^ μm^3^, each surrounded by 12 ± 2 pre-granulosa cells on average (Fig. 1h). Upon follicle activation, the flattened pre-granulosa cells differentiated into a monolayer of cuboidal granulosa cells^39^. Correspondingly, primary follicles exhibited an oocyte diameter of 36.79 ± 7.56 μm, a follicle volume of 1.68 ± 0.83 × 10^5^ μm^3^, and contained 85 ± 32 granulosa cells (Fig. 1h). Further follicular development was marked by the formation of a second granulosa cell layer, defining the secondary follicle stage. At this stage, the oocyte diameter increased to 48.98 ± 3.90 μm, follicle volume reached 4.12 ± 1.35 × 10^5^ μm^3^, and each follicle contained 237 ± 105 granulosa cells (Fig. 1h). Notably, granulosa cell numbers per follicle increased sharply in parallel with follicle volume expansion (Supplementary Fig. 1m). Spatial analysis further revealed that the ovarian cortex of PD7 mice had an average thickness of 82.63 ± 8.98 μm and harbored densely packed oocytes, which were significantly more abundant than those located in the medulla (Fig. 1i and Supplementary Fig. 1n). Collectively, these results provide an in-depth and quantitative view of ovarian architecture and early follicular development. The APEX^oocyte^ reporter mouse enables precise visualization of spatial ovarian organization and provides a robust platform for quantitative tracking of follicles across developmental and pathological contexts.

### In situ proteomic profiling of oocytes from early-stage follicles

**Fig. 2:**
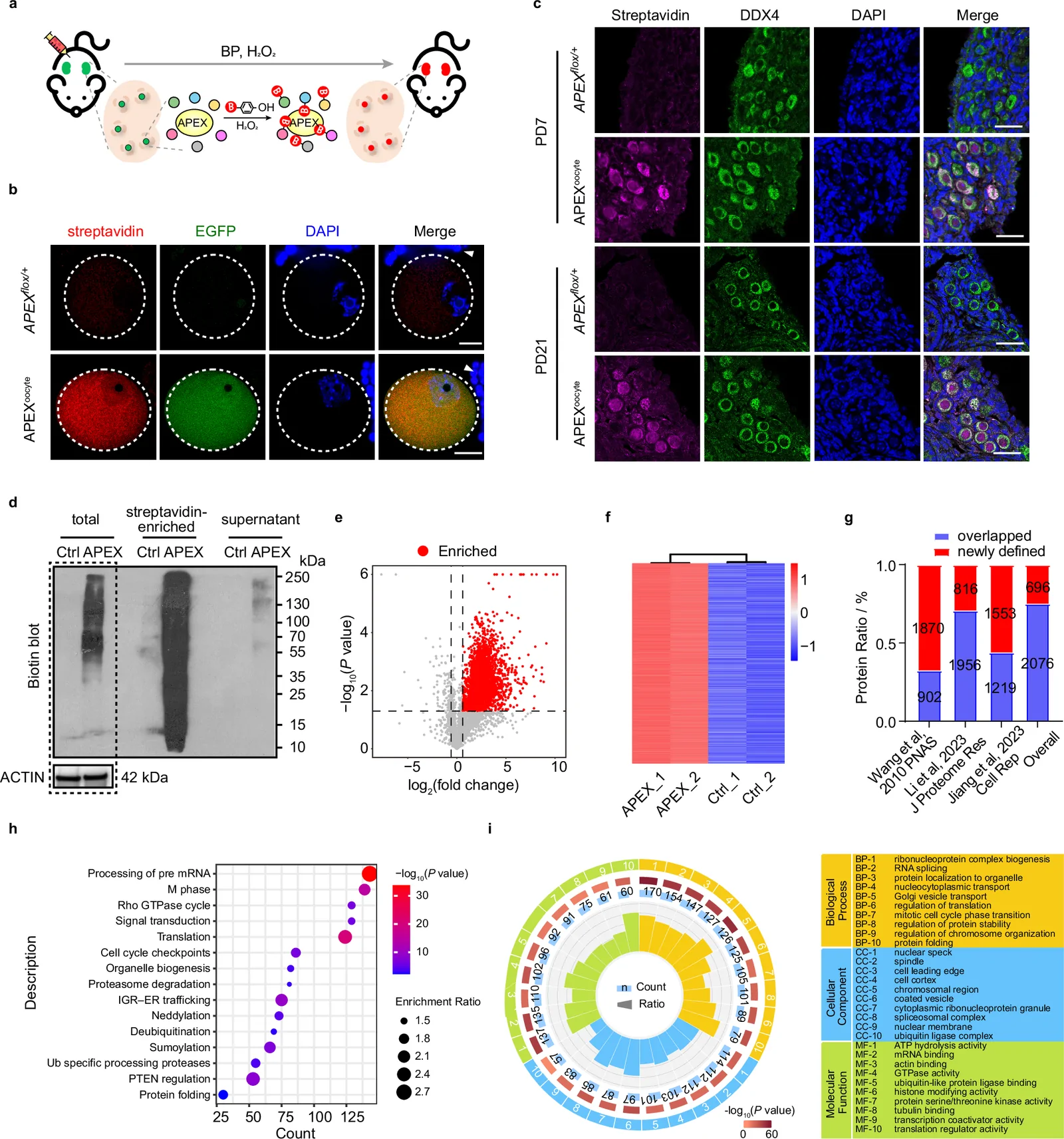
In situ APEX-based proteomic profiling of oocytes from early-stage ovarian follicles. **a** Scheme of APEX2-driven in situ protein biotinylation in APEX^oocyte^ mouse ovaries. Biotin-phenol (BP, 500 μM) was administered intraperitoneally (i.p.), followed 1 h later by H_2_O_2_ (1 mM, i.p.) to initiate a 5-min APEX reaction before ovary collection. **b** Immunofluorescence staining of COCs collected from 3–4-week-old females after biotinylation. Biotinylated proteins were detected specifically in oocytes of APEX^oocyte^ females, but not in surrounding granulosa cells (arrowheads) or oocytes of *APEX*^*flox/+*^ mice. Representative images from three independent experiments with similar results are shown. Scale bars, 20 μm. **c** Streptavidin labeling in ovarian sections from PD7 and PD21 APEX^oocyte^ and *APEX*^*flox/+*^ mice. Co-staining for streptavidin (magenta), DDX4 (green), and DAPI (blue) is shown. Representative images from three independent experiments with similar results are shown. Scale bars, 50 μm. **d** Streptavidin blot of total lysate, streptavidin-enriched fraction, and supernatant from PD7 ovaries of *APEX*^*flox/+*^ control (Ctrl) and APEX^oocyte^ mice. ACTIN served as a loading control. The blot is a representative of three independent experiments. **e** Volcano plot of proteins detected by LC–MS/MS comparing APEX^oocyte^ and *APEX*^*flox/+*^ samples. Significantly enriched proteins are highlighted (red dots, fold change >1.5, *P* < 0.05). *P* values were calculated using a two-sided unpaired Student’s t-test, without adjustment for multiple comparisons. **f** Heatmap of enriched proteins across biological replicates. **g** Overlap of the identified proteome with previously published GV-stage oocyte proteomes. Stacked bars indicate overlapped and newly defined proteins. **h** Bubble plot of enriched Reactome pathways. Bubble size indicates enrichment ratio and color indicates significance (–log_10_ [*P* value]). Significance was assessed using a one-sided hypergeometric test with Benjamini–Hochberg correction for multiple comparisons. **i** Circular plot of the top 10 enriched gene ontology (GO) terms in biological process (yellow), cellular component (blue), and molecular function (green). From inside to outside: enrichment factor (Ratio), gene number (Count), statistical significance (–log_10_ [*P* value]), and GO term IDs corresponding to the descriptions on the right. Significance was assessed using a one-sided hypergeometric test with Benjamini–Hochberg correction for multiple comparisons.

Given the limited knowledge of oocyte proteomes in early-stage ovarian follicles in vivo, we sought to establish an in situ APEX-based labeling system for oocyte proteomic profiling. The reaction substrate biotin-phenol (BP) was administered to mice by intraperitoneal injection (i.p.), and one hour later the APEX reaction was initiated by injection of H_2_O_2_. As a result, proteins close to APEX2 within oocytes were biotinylated (Fig. 2a). To assess the specificity of in vivo labeling, we first examined whether APEX2 selectively biotinylated oocyte proteins in APEX^oocyte^ mice. *APEX*^*flox/+*^ mice receiving the same treatment were used as negative controls. Following in situ APEX reaction, isolated COCs or intact ovaries from PD21 mice were fixed and subjected to IF analysis (Fig. 2b, c). Fluorescently labeled streptavidin was used to visualize biotinylated proteins. Robust streptavidin signals were detected specifically in oocytes from APEX^oocyte^ mice, whereas no detectable labeling was observed in oocytes from *APEX*^*flox/+*^ controls (Fig. 2b). IF analysis of ovarian sections further revealed that the majority of oocytes responded efficiently to the in vivo labeling reaction (Fig. 2c). To confirm that APEX-mediated labeling captured all oocytes essentially, we performed co-immunostaining of streptavidin with the oocyte marker DDX4 on ovarian sections. Streptavidin signals showed substantial overlap with DDX4-positive oocytes, including those residing in quiescent primordial follicles (Fig. 2c), indicating that *Gdf9*-Cre–driven APEX expression is sufficient for labeling oocytes at this stage despite relatively low Cre activity. Comparable biotinylation patterns were also observed in oocytes from PD7 APEX^oocyte^ ovaries (Fig. 2c), and DDX4/streptavidin co-staining confirmed efficient cortical oocyte labeling under the 1 h BP incubation condition with reduced labeling of inner growing oocytes compared with longer BP incubation (Supplementary Fig. 2a). PD7 ovaries labeled under this condition were subsequently used for proteomic analysis of early-stage follicular oocytes, thereby validating the robustness and efficiency of the in situ APEX2-driven labeling system in the ovary. Importantly, the in vivo labeling procedure did not measurably perturb ovarian or oocyte physiology. Histological analyses revealed normal ovarian morphology (Supplementary Fig. 2b, c) and comparable follicle numbers across developmental stages (Supplementary Fig. 2d, e) in BP + H₂O₂–treated and control mice at both PD7 and PD21. No significant increase in follicular apoptosis was detected, as assessed by cleaved caspase-3 staining at PD21 (Supplementary Fig. 2f, g) and TUNEL assays at PD7 (Supplementary Fig. 2h). Moreover, FGOs retrieved from PD21 APEX^oocyte^ females after in vivo labeling exhibited normal meiotic maturation, spindle organization, and polar body extrusion rates comparable to controls (Supplementary Fig. 2i, j). Together, these results establish a robust, specific, and physiologically compatible in situ APEX-based labeling strategy that enables efficient proteomic profiling of oocytes across early-stage ovarian follicles in vivo.

To obtain the oocyte proteome from early-stage ovarian follicles, particularly primordial follicles, ovaries from PD7 mice were subjected to proximity labeling and subsequently collected, as primordial follicles constitute the dominant follicle population at this stage. Biotinylated proteins extracted from lysed ovaries were enriched using streptavidin-coated beads and then separated by gel electrophoresis. Streptavidin blotting revealed a wide range of biotinylated proteins in PD7 APEX^oocyte^ mouse ovaries, whereas only minimal background signals were detected after enrichment from *APEX*^*flox/+*^ control ovaries (Fig. 2d). These results indicated successful capture of oocyte proteome. To assess the potential impact of H₂O₂ treatment on the ovarian proteome, we performed differential proteomic analysis of PD7 ovaries with or without H₂O₂ injection. Expression distributions and sample correlations indicated high reproducibility among biological replicates (Supplementary Fig. 2k, l). Among the 6055 proteins detected, only 142 proteins (~2%) were differentially expressed, including 45 upregulated and 97 downregulated proteins (Supplementary Fig. 2m). Importantly, no oxidative stress–related pathways were significantly enriched among the dysregulated proteins (Supplementary Fig. 2n), indicating that the proteomic perturbation induced by H₂O₂ under our experimental conditions was minimal. Next, streptavidin-enriched samples from PD7 APEX^oocyte^ ovaries were subjected to liquid chromatography–tandem mass spectrometry (LC-MS/MS) to profile the oocyte proteome of early-stage ovarian follicles, with proteins from identically treated *APEX*^*flox/+*^ ovaries serving as negative controls. Protein abundance was quantified based on MS intensity using label-free quantification. Proteomic data from two biological replicates showed a high degree of correlation, with protein abundances following a normal distribution across all samples, and the total abundance of APEX-driven biotinylated proteins in the experimental group was substantially higher than the background levels observed in the control group (Supplementary Fig. 3a–c). In total, 2772 proteins were significantly enriched in the APEX^oocyte^ mice group compared with the control group (FC > 1.5, *P* < 0.05), representing a primordial follicle–enriched oocyte proteome from early-stage ovarian follicles (Fig. 2e, f and Supplementary Data 1).

To assess the reliability and developmental relevance of the oocyte proteome identified in this study, we compared our dataset with three previously published proteomic studies of FGOs (GV stage)^15–17^. Notably, approximately 75% of the 2772 proteins identified here overlapped with proteins previously detected in GV-stage oocytes, indicating that a substantial fraction of proteins required for later oocyte growth and maturation are already expressed at early follicular stages (Fig. 2g). In contrast, 696 proteins identified in our dataset were absent from prior GV-stage oocyte proteomes included in this comparison, suggesting that these proteins may be preferentially enriched in oocytes from primordial or early-stage follicles (Fig. 2g, Supplementary Data 2). Importantly, multiple well-recognized primordial oocyte markers, including DDX4 (FC = 4.38), spermatogenesis and oogenesis specific basic helix-loop-helix 1 (SOHLH1) (FC = 2.23), and NOBOX oogenesis homeobox (NOBOX) (FC = 3.22), were identified in our proteomic dataset (Supplementary Data 1), supporting the accurate capture of oocytes at this developmental stage. In addition, several proteins previously implicated in early oocyte regulation, such as damage-specific DNA-binding protein 1 (DDB1) (FC = 6.12), eukaryotic translation initiation factor 4E (EIF4E) (FC = 6.62), and YTH-domain N-6-methyladenosine RNA-binding protein 2 (YTHDF2) (FC = 5.34), were also detected (Supplementary Data 1). Enrichment of representative proteins was further validated by streptavidin pull-down followed by Western blotting (Supplementary Fig. 3d). Moreover, immunohistochemical analysis showed that EIF4E and cell division cycle 27 (CDC27) were detectable in oocytes across different follicular stages, and CDC27 exhibited relatively higher abundance in primordial follicle oocytes (Supplementary Fig. 3e), further corroborating the physiological relevance of the identified proteins. To gain functional insights into the defined oocyte proteome, pathway enrichment analyses were performed. Reactome analysis revealed significant enrichment of pathways involved in pre-mRNA processing, translation, and cell cycle–related processes, together with pathways associated with protein folding, proteasome degradation, and ubiquitin-related processing (Fig. 2h). In addition, signaling-related pathways such as Rho GTPase cycle and intracellular trafficking, including Golgi vesicle transport and ER-associated transport, were also enriched (Fig. 2h). Consistently, gene ontology (GO) analysis highlighted enrichment of biological processes related to ribonucleoprotein complex biogenesis, RNA splicing, regulation of protein stability, and chromosome organization, as well as molecular functions associated with histone-modifying activity, protein serine/threonine kinase activity, and ubiquitin-like protein ligase binding (Fig. 2i). Together, these results demonstrate that our proteomic dataset reflects the molecular characteristics of oocytes at primordial and early follicular stages.

### Expression dynamics of representative regulatory proteins in oocytes

**Fig. 3:**
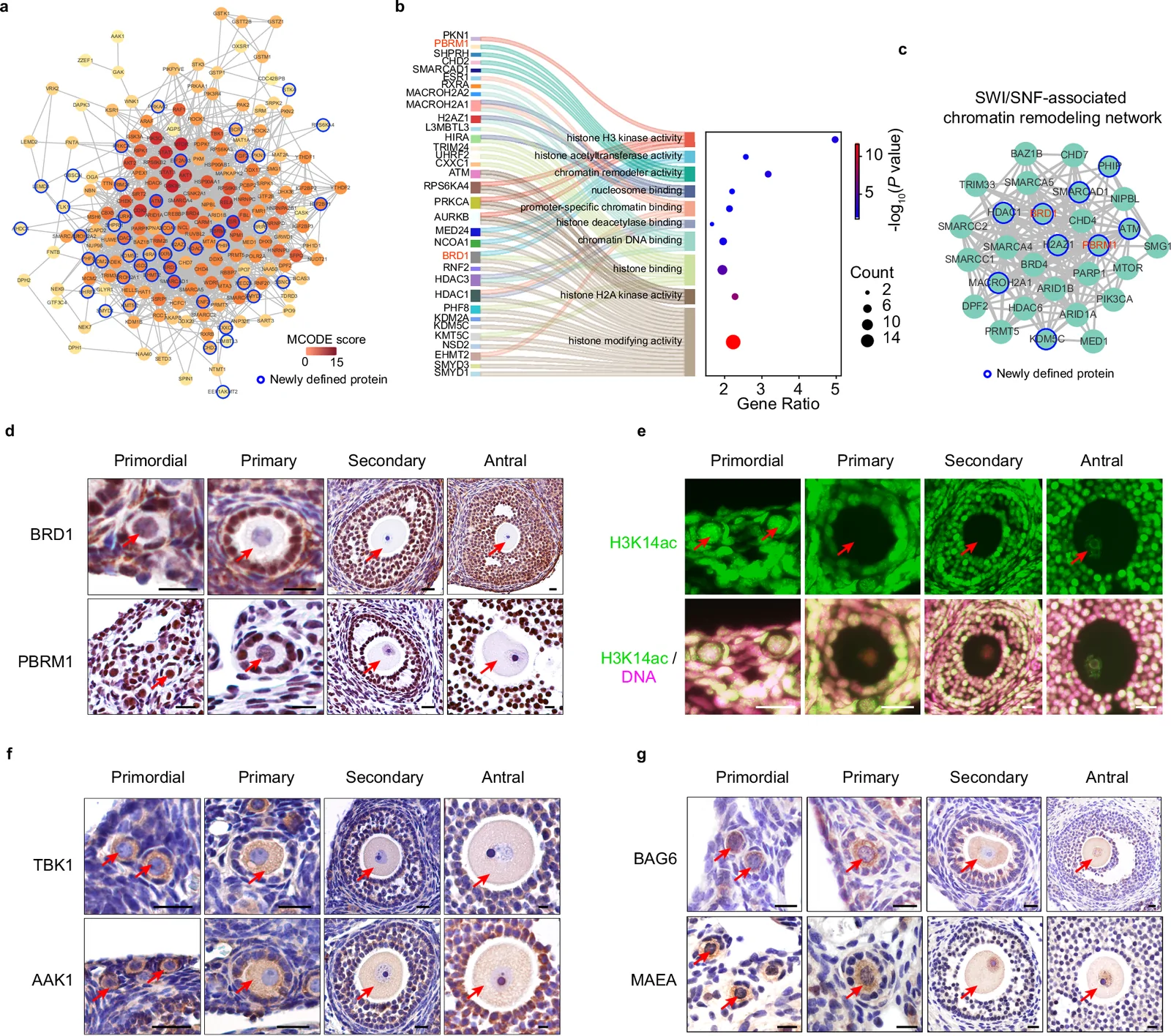
Expression dynamics of representative regulatory proteins in oocytes. **a** Protein–protein interaction network of chromatin regulation–associated proteins identified in the oocyte proteome. Nodes represent proteins, colored according to the MCODE scores, with dark shading representing higher protein interconnection. Newly defined proteins are indicated with bold outlines. **b** Sankey plot showing the relationship between newly defined proteins (left) and functional categories (right). The size and color of the bubbles on the right correspond to the gene number (Count) and –log_10_(*P* value), respectively, representing the enrichment level of each functional category. Significance was assessed using a one-sided hypergeometric test with Benjamini–Hochberg correction for multiple comparisons. **c** Subnetwork of SWI/SNF-associated chromatin remodeling proteins. Newly defined proteins are indicated by bold-outlined circles. **d** Immunohistochemical (IHC) staining of bromodomain containing 1 (BRD1) and polybromo 1 (PBRM1) in ovarian sections across follicle stages (primordial, primary, secondary, and antral). Arrows indicate oocytes. Scale bars, 20 μm. **e** IF staining of histone H3 lysine 14 acetylation (H3K14ac) (green) in ovarian sections across follicle stages. Merged images with DNA (magenta) are shown. Arrows indicate oocytes. Scale bars, 20 μm. **f** IHC staining of TANK binding kinase 1 (TBK1) and AP2 associated kinase 1 (AAK1) in ovarian sections across follicle stages. Arrows indicate oocytes. Scale bars, 20 μm. **g** IHC staining of BAG cochaperone 6 (BAG6) and macrophage erythroblast attacher (MAEA) in ovarian sections across follicle stages. Arrows indicate oocytes. Scale bars, 20 μm. All the IHC and IF results presented here are representative of three independent experiments.

Functional enrichment analyses revealed that the oocyte proteome identified in early-stage follicles is prominently enriched for proteins involved in chromatin regulation, phosphorylation-dependent regulation, and protein stability control. To further explore these features, we integrated protein–protein interaction analysis with immunohistochemical validation of representative proteins. From the 2772 enriched proteins, a chromatin regulation–associated interaction network comprising 204 proteins was constructed (Fig. 3a). This network included chromatin remodelers, histone modifiers, and transcriptional regulators, with significant enrichment of functions related to chromatin remodeling activity, histone modification, nucleosome binding, and chromatin DNA binding (Fig. 3b). Notably, 49 proteins within this network belonged to these 696 proteins (Fig. 3a), indicating that chromatin regulatory programs are already extensively established in primordial and early-stage oocytes. Among these, bromodomain containing 1 (BRD1), a bromodomain-containing protein implicated in histone acetylation–dependent transcriptional regulation, and polybromo 1 (PBRM1), a core subunit of the SWI/SNF chromatin remodeling complex, were selected for validation (Fig. 3c). Immunohistochemical analysis revealed that both BRD1 and PBRM1 were detectable in oocytes across follicular stages, with relatively higher abundance observed in primordial follicle oocytes (Fig. 3d). Secondary antibody–only controls showed no detectable staining, confirming the specificity of the immunohistochemical signals (Supplementary Fig. 4g). Consistent with the chromatin-centered nature of this network, we further examined histone H3 lysine 14 acetylation (H3K14ac), a histone modification associated with transcriptionally active chromatin. H3K14ac signals were readily detected in primordial follicle oocytes and displayed dynamic redistribution during follicular growth (Fig. 3e), supporting active chromatin regulation at early developmental stages.

In parallel, proteins involved in kinase-mediated regulation formed a densely connected interaction network consisting of 236 proteins (Supplementary Fig. 4a), enriched for molecular functions such as protein serine/threonine kinase activity, histone kinase activity, kinase binding, and phosphoprotein binding (Supplementary Fig. 4b). This network contained 55 of these 696 proteins (Supplementary Fig. 4a), supporting the presence of phosphorylation-related regulation in early oocytes. Two representative kinases, TANK binding kinase 1 (TBK1), a serine/threonine kinase involved in stress and cell-cycle–related signaling, and AP2 associated kinase 1 (AAK1), a regulator of clathrin-mediated endocytosis and kinase signaling, were examined. Both proteins were detectable in oocytes throughout folliculogenesis, with stronger signals evident in primordial follicles compared with later stages (Fig. 3f). In addition, enrichment analyses highlighted extensive representation of proteins involved in protein stability and ubiquitin–proteasome regulation. A corresponding interaction network comprising 265 proteins was generated (Supplementary Fig. 4c), enriched for ubiquitin-like protein ligase binding, deubiquitination, proteasome binding, and protein folding–related functions (Supplementary Fig. 4d). Within this network, 37 proteins belonged to these 696 proteins (Supplementary Fig. 4c), suggesting active protein homeostasis mechanisms already operating in early-stage oocytes. BAG cochaperone 6 (BAG6), a chaperone involved in misfolded protein surveillance, and macrophage erythroblast attacher (MAEA), a core component of the CTLH E3 ubiquitin ligase complex, were selected as representative proteins. BAG6 showed expression in oocytes at all follicular stages, with relatively higher abundance in primordial follicles, whereas MAEA exhibited dynamic subcellular localization during folliculogenesis (Fig. 3g). Finally, to evaluate whether the expression patterns of these regulatory proteins were influenced by age, ovaries from PD7, 2-week-old, 3-week-old, and 12-month-old mice were examined. TBK1, AAK1, BAG6, and MAEA displayed comparable expression patterns in early follicle oocytes across all examined ages (Supplementary Fig. 4e, f), indicating that their enrichment in primordial and early-stage oocytes is largely age independent. Together, these results indicate that the identified oocyte proteome is organized into interconnected regulatory networks and reflects active regulatory programs already operating in primordial follicle oocytes.

### Characterization of the transcriptome of early follicle oocytes

**Fig. 4:**
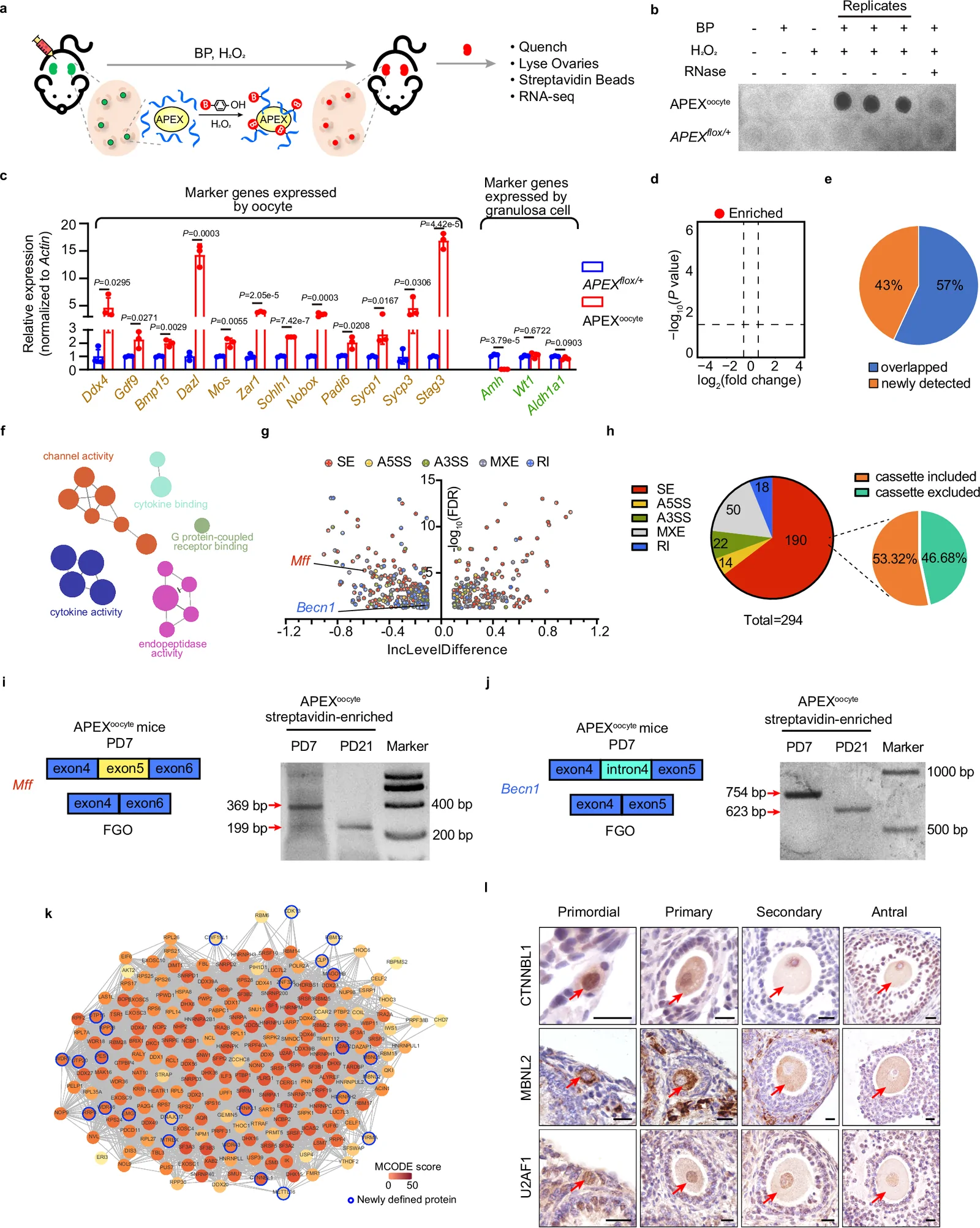
Characterization of the transcriptome of early follicle oocytes using APEX^oocyte^ labeling system. **a** Schematic of in situ RNA biotinylation in APEX^oocyte^ mice. BP was administered to label RNA in oocytes, followed by H₂O₂ injection to trigger the APEX reaction. Labeled RNA was extracted for subsequent analysis. **b** Streptavidin dot blot analysis of total RNA from APEX^oocyte^ and *APEX*^*flox/+*^ mice. Biotinylation signals were detected in APEX^oocyte^ mice but not in the absence of BP or H₂O₂ or following RNase treatment. **c** Quantitative PCR (qPCR) analysis of oocyte- and granulosa cell-specific marker genes in biotinylated RNAs. Strong enrichment of oocyte-specific transcripts (e.g., *Ddx4, Gdf9, Bmp15*) and minimal enrichment of granulosa cell markers (e.g., *Amh*). *n* = 3 biologically independent experiments. Mean ± SEM. *P* values are calculated by unpaired two-tailed Student’s t tests. **d** Volcano plot of differentially expressed gene transcripts between APEX^oocyte^ and *APEX*^*flox/+*^ mice. Red dots indicate significantly enriched genes (FC > 1.5, *P* < 0.05). *P* values were calculated using a two-sided unpaired Student’s t-test, without adjustment for multiple comparisons. **e** Comparison of the identified transcriptome with previously published GV-stage oocyte transcriptome. **f** GO enrichment analysis of newly detected early follicle oocyte transcripts. **g** Alternative splicing events identified between early follicle oocytes and FGOs. Red, skipped exons (SE). Yellow, alternative-5′ spliced sites (A5SS). Green, alternative-3′ spliced sites (A3SS). Grey, mutually exclusive exons (MXE). Blue, retained introns (RI). **h** Pie charts illustrating the proportion of differential alternative splicing events in each category. **i**, **j** Schematics and PCR gel electrophoresis showing alternative splicing of mitochondrial fission factor (*Mff*) and beclin-1 (*Becn1*) transcripts in oocytes at different ovary stages. The images are representative of three independent experiments. **k** Protein–protein interaction network of splicing-related proteins identified in the oocyte proteome. Newly defined proteins are indicated with bold borders. Node shading reflects MCODE score. **l** IHC staining of beta-catenin-like protein 1 (CTNNBL1), muscleblind-like splicing regulator 2 (MBNL2), and U2 small nuclear RNA auxiliary factor 1 (U2AF1), in ovarian sections across follicular stages. Arrows indicate oocytes. Representative images from three independent experiments with similar results are shown. Scale bars, 20 μm.

Given that APEX2 peroxidase can also directly label RNA^40^, we next characterized the transcriptome of oocytes enriched for primordial follicles by in situ biotinylating living oocytes RNA of APEX^oocyte^ mice (Fig. 4a). To test the efficiency of APEX-catalyzed RNA biotinylation, after APEX reaction, we extracted total RNA and analyzed the RNA by streptavidin dot blotting. The results indicated that oocyte-specific APEX2 succeeded in RNA biotinylation, as the APEX^oocyte^ mice group presented positive signals, whereas RNA biotinylation was abolished upon omission of BP or H_2_O_2_ or following treatment with RNase (Fig. 4b). Subsequentially, we assessed the cellular specificity of this approach using APEX labeling and qRT-PCR analysis of biotinylated RNAs. Enriched biotinylated RNAs were reverse-transcribed into complementary DNAs (cDNAs), and the expression of oocyte- and somatic cell-specific genes was verified by quantitative polymerase chain reaction (qPCR). The results revealed strong enrichment of transcripts of known oocyte-specific genes (*Ddx4*, *Gdf9*, *Bmp15*, *Dazl*, *Mos*, *Zar1, Sohlh1*, and *Nobox*), as well as additional oocyte-associated genes involved in early oocyte development and meiotic programs (*Padi6, Sycp1, Sycp3*, and *Stag3*). Conversely, there was no significant enrichment of negative-control mRNAs highly expressed in granulosa cells (*Amh*, *Wt1*, and *Aldh1a1*) (Fig. 4c). Therefore, APEX2-driven platform we constructed in this study is able to label and enrich oocyte mRNA and protein from APEX^oocyte^ mouse ovaries specifically.

Then, we conducted a more comprehensive analysis using transcriptome-wide sequencing. RNAs from PD7 APEX^oocyte^ mouse ovaries were labeled, enriched, and subjected to SMART-seq2 analysis. The transcriptome was analyzed in triplicate with eight ovaries per replicate. The triplicates reflect a high correlation within each group (Supplementary Fig. 5a–d). Gene expression was assessed as transcripts per million mapped reads (TPM). Among them, 2878 genes were enriched (FC > 1.5, *P* < 0.05) in the APEX^oocyte^ mice group than in the *APEX*^*flox/+*^ group, which were designated as the oocyte transcriptome primarily derived from primordial follicles (Fig. 4d and Supplementary Data 3). Numerous germ cell markers were included in the transcriptome, whereas most granulosa cell markers were not enriched in the APEX2-driven analysis (Supplementary Fig. 5e, f). The APEX-directed transcriptome also included genes detected in previous single-cell analyses during primordial follicle assembly^41^, with high relevance to the set 3 genes mainly expressed at the pre- and early follicle formation stages (Supplementary Fig. 5g, h). Approximately 57% of the gene transcripts overlapped between the transcriptomes of primordial follicular oocytes and oocytes at later developmental stages (Fig. 4e)^42^. GO analysis of the newly detected oocyte transcripts revealed that genes with cytokine activity and channel activity were detected in early follicular oocytes (Fig. 4f). Overall, the RNA and protein expression profiles were positively correlated (Supplementary Fig. 5i), yet a subset of genes displayed weak RNA–protein concordance, suggesting extensive post-transcriptional regulation and an intermediate state of gene expression during oocyte development.

Alternative splicing of RNA is a critical regulator for gene expression and protein diversity, playing a role in oocyte development^43^. In our study, we also found multiple alternative splicing events via a transcriptome-wide comparison between primordial follicular oocytes and FGOs (Fig. 4g, h and Supplementary Data 4). Mature mRNA of the mitochondrial fission factor (*Mff*) contains an alternatively skipped exon at exon 5. Visual assessment of the PCR products using agarose gel electrophoresis showed that the exon 5-containing *Mff* isoform was specifically enriched in oocytes at the primordial follicle stage, as this longer isoform was absent from the biotinylated RNAs retrieved from PD21 APEX^oocyte^ mouse ovaries (Fig. 4i). Beclin-1 (*Becn1*) is responsible for membrane trafficking and autophagy. Its transcripts retained intron 4 in primordial follicle oocytes, which would be excluded in FGOs (Fig. 4j). Correspondingly, proteomic profiling revealed a cohort of RNA splicing and spliceosome-related proteins. These proteins formed a highly connected interaction module (Fig. 4k), underscoring active post-transcriptional regulation during folliculogenesis. Notably, this network contained 25 proteins identified through our analysis (Fig. 4k, Supplementary Fig. 5j), further supporting the presence of splicing-associated components in early-stage oocytes. To validate representative splicing-related proteins, we examined ovarian staining of three factors, including beta-catenin-like protein 1 (CTNNBL1), muscleblind-like splicing regulator 2 (MBNL2), and U2 small nuclear RNA auxiliary factor 1 (U2AF1). CTNNBL1 is a spliceosome-associated protein implicated in snRNP recycling and spliceosomal dynamics, and it showed prominent nuclear enrichment in oocytes across multiple follicular stages. MBNL2 is a well-established regulator of alternative splicing and RNA processing, and it was broadly detectable in oocytes with predominant cytoplasmic localization. U2AF1 is a core splicing factor required for 3′ splice-site recognition during pre-mRNA splicing, and it exhibited clear nuclear staining in oocytes across follicles of different stages (Fig. 4l). Overall, our transcriptomic and proteomic analyses jointly support active RNA splicing–related regulation in oocytes during folliculogenesis, with representative factors showing distinct subcellular localization patterns.

### Cisplatin affects the proteome of primordial follicle oocytes

**Fig. 5:**
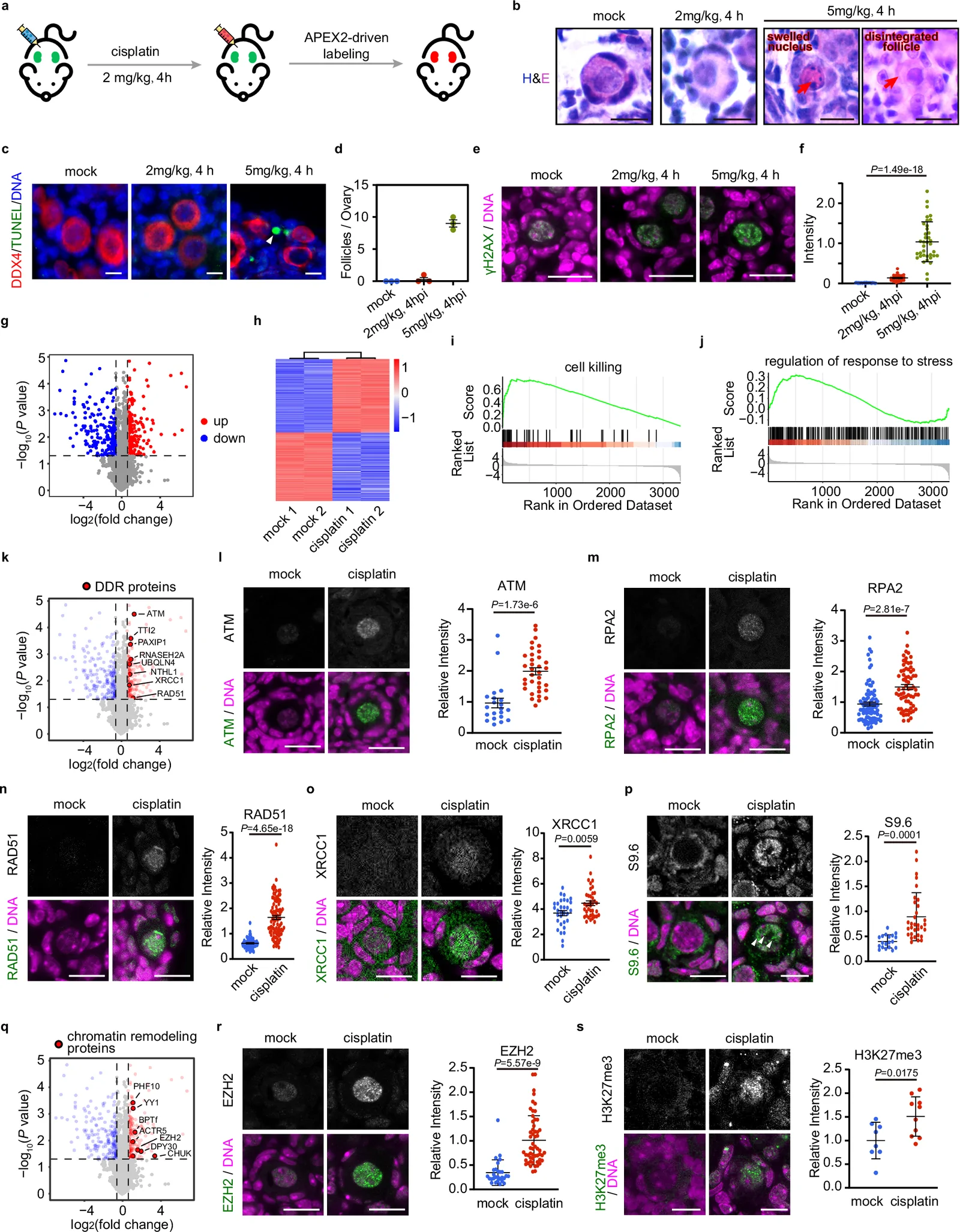
Cisplatin induces proteomic alterations in primordial follicle oocytes. **a** Schematic of cisplatin treatment in PD7 female mice. Cisplatin (2 mg/kg) was administered intraperitoneally; PBS-treated mice served as controls. After 4 h, APEX2-driven biotinylation was performed. **b** Hematoxylin and Eosin (H&E) staining of primordial follicles before and after cisplatin treatment. **c** TUNEL staining (green) with DDX4 (red) and DNA (blue) of primordial follicles before and after cisplatin treatment. **d** Counts of apoptotic primordial follicles in PD7 ovaries. Only primordial follicles with TUNEL signals were quantified. *n* = 3 biologically independent ovaries. **e** IF of phosphorylated H2A.X (γH2AX) before and after cisplatin treatment from PD7 mice. **f** Quantification of γH2AX signals in (**e**). *n* = 34, 30, and 37 follicles for the mock, 2 mg/kg (4 hpi), and 5 mg/kg (4 hpi) groups. **g** Volcano plot of differentially expressed proteins after low-dose cisplatin treatment. Upregulated (fold change > 1.5, *P* < 0.05) and downregulated (fold change < 0.67, *P* < 0.05) proteins are shown in red and blue, respectively. **h** Heatmap of altered proteins between mock and cisplatin groups. **i**, **j** GSEA plots showing enrichment of cell killing and stress response pathways after cisplatin treatment. **k** Volcano plot highlighting DNA damage response (DDR) proteins. **l**–**p** Immunostaining and quantification of ATM, RPA2, RAD51, XRCC1, and RNA–DNA hybrids (R-loops) before and after cisplatin treatment. R-loops were detected by S9.6 staining, and white arrows indicate puncta in treated oocytes. *n* = 21 and 35 follicles (**l**), 87 and 66 follicles (**m**), 56 and 88 follicles (**n**), 31 and 38 follicles (**o**), and 19 and 32 follicles (**p**) for the mock and cisplatin groups, respectively. **q** Volcano plot highlighting chromatin remodeling proteins. **r**, **s** EZH2 and H3K27me3 immunostaining and quantification before and after cisplatin treatment. *n* = 28 and 55 follicles (**r**) and 8 and 10 follicles (**s**) for the mock and cisplatin groups, respectively. Scale bars, 20 μm in (**b**, **c**, **e**, **l**–**p**, **r**, **s**). Mean ± SEM in (**d**, **f**, **l**–**p**, **r**, **s**). *P* values are calculated by unpaired two-tailed Student’s t tests in **f**, **g**, **k**–**s**, without adjustment for multiple comparisons.

Cisplatin is known to impair female fertility in patients with malignancy, in part through damage to the primordial follicle pool that constitutes the ovarian reserve^6,44^. While follicle loss and DNA damage have been documented, the associated proteomic alterations in oocytes enriched from primordial follicles remain incompletely characterized. We therefore investigated proteomic changes before and after cisplatin treatment. To examine the potential effects of cisplatin on oocytes in mice, PD7 females were administered cisplatin i.p. at 5 mg/kg body weight (bw) (a dose commonly used in mouse studies and corresponding to clinically relevant exposure)^45–47^, or at 2 mg/kg bw (a low dose modeling residual drug levels after chemotherapy or combination regimens). Ovarian morphology, oocyte viability, and follicle number were assessed at 4 h post-injection (hpi), with PBS-injected mice serving as controls (Fig. 5a). Low-dose cisplatin (2 mg/kg, 4 h) did not produce obvious changes in follicle number or morphology, whereas the higher dose (5 mg/kg, 4 h) resulted in a significant reduction in follicle number, abnormal morphology, and increased follicular apoptosis (Fig. 5b–d and Supplementary Fig. 6a–d). Consistently, phospho-H2AX (γH2AX) staining showed that the higher dose induced marked DNA double-strand break (DSB) signals, whereas the lower dose induced only a modest increase in γH2AX signal intensity without obvious follicular morphological abnormalities (Fig. 5e, f and Supplementary Fig. 6a). Because the lower dose induced a detectable DNA damage response without overt follicular disruption or extensive cell death, we selected this condition for proteomic analysis to capture early cisplatin-induced molecular alterations while avoiding dominant cell death-associated changes caused by high-dose treatment. Under this condition, we analyzed the proteome of oocytes predominantly derived from primordial follicles in APEX^oocyte^ mice. In total, 210 proteins were upregulated (FC > 1.5, *P* < 0.05) and 192 were downregulated (FC < 0.67, *P* < 0.05) relative to untreated controls (Fig. 5g, h, Supplementary Fig. 6e, f and Supplementary Data 5). Gene set enrichment analysis (GSEA) showed that cisplatin-responsive proteins were enriched in processes related to cell killing, regulation of response to stress, ATP metabolic process, and ribonucleotide metabolic process (Fig. 5i, j and Supplementary Fig. 6g).

Since cisplatin primarily induces DNA alkylation and DSB-associated responses, we first examined DNA damage response (DDR)-related proteins in the proteomic dataset. Proteomic analysis highlighted multiple DDR components (Fig. 5k), consistent with the presence of low-level DSBs after low-dose cisplatin exposure, which was further supported by immunostaining showing nuclear upregulation of several key DDR factors. ATM serine/threonine kinase (ATM), a central serine/threonine kinase in the DNA damage response that phosphorylates H2A.X and other DDR proteins, showed weak and diffuse signals in control oocytes but became markedly stronger and formed nuclear foci following treatment (Fig. 5l). Consistently, single-stranded DNA–binding protein replication protein A2 (RPA2) and homologous recombination–associated protein RAD51 recombinase (RAD51) both showed prominent nuclear accumulation in cisplatin-treated oocytes (Fig. 5m, n), indicating activation of DSB repair pathways. X-ray repair cross complementing 1 (XRCC1), a scaffold protein in base excision and single-strand break repair, was also increased (Fig. 5o), suggesting engagement of multiple DNA repair routes. In parallel, staining with the S9.6 antibody revealed punctate nuclear RNA–DNA hybrid (R-loop) signals in treated oocytes but not in controls (Fig. 5p). RNase H2A, a factor involved in resolving RNA–DNA hybrids, was correspondingly elevated (Supplementary Fig. 6h), consistent with enhanced turnover of R-loops during genotoxic stress. Beyond canonical DDR proteins, proteomic profiling also revealed enrichment of chromatin regulatory factors. Enhancer of zeste 2 polycomb repressive complex 2 subunit (EZH2), the catalytic subunit of polycomb repressive complex 2 (PRC2) responsible for H3K27 trimethylation (H3K27me3), showed low basal nuclear levels but was markedly increased after cisplatin exposure (Fig. 5q, r and Supplementary Fig. 6h). Concordantly, H3K27me3 signals were significantly elevated with the duration of cisplatin treatment (Fig. 5s), indicating remodeling of the chromatin epigenetic landscape in response to DNA damage. Together, these findings show that low-dose cisplatin induces a detectable DNA damage response in primordial follicle oocytes, while APEX-based proteomic analysis further reveals broader molecular alterations beyond canonical DDR markers, including stress-related pathways, metabolic changes, and chromatin regulatory machinery exemplified by elevated EZH2 and H3K27me3. The functional consequences of these chromatin changes require further investigation.

In addition, the effect of low-concentration cisplatin on the transcriptome of oocytes was also analyzed. Using the established system above, we found that 482 genes were upregulated (FC > 1.5, *P* < 0.05) and 582 genes were downregulated (FC < 0.67, *P* < 0.05) after cisplatin treatment (Supplementary Fig. 6i and Supplementary Data 6). Enrichment analysis showed that the upregulated genes were enriched in the pyroptosis, drug response regulation, GPCR downstream signaling pathways, etc., while the downregulated genes were enriched in the apoptotic signaling regulation pathway, ubiquitin-conjugating enzyme activity, glycolytic process through glucose-6-phosphate pathways, etc. (Supplementary Fig. 6j, k). This might explain the rapid fashion of protein shift with cisplatin treatment. Compared with the clinical effect of cisplatin on tumor cells^48,49^, the response of oocytes to cisplatin was quite different (Supplementary Fig. 6l–n), implying specific damage of cisplatin to female germ cells. Collectively, these results demonstrate that our APEX in vivo labeling system uncovers a molecular shift in primordial follicle oocytes upon cisplatin treatment and warns of substantial risk to female tumor patients who are subjected to low dosages of cisplatin chemotherapy.

### EZH2 inhibition mitigates cisplatin-induced oocyte defects

**Fig. 6:**
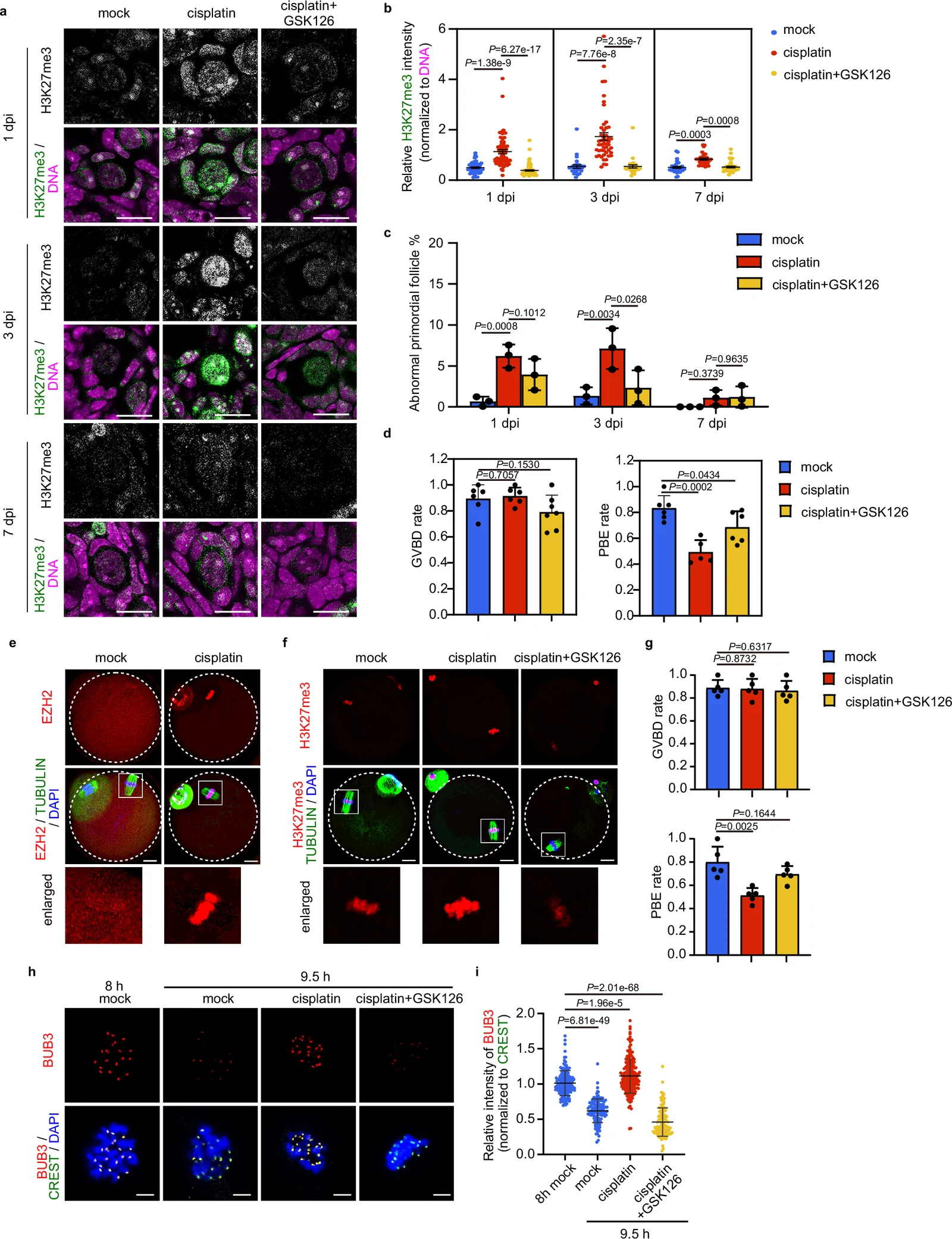
EZH2 inhibition mitigates cisplatin-induced oocyte defects. **a** Representative immunofluorescence images of H3K27me3 (green) and DNA (magenta) in primordial follicle oocytes at 1, 3, and 7 days post-injection (dpi) in mock, cisplatin, and cisplatin+GSK126 groups treated at PD7. Scale bar, 20 μm. **b** Quantification of H3K27me3 IF intensity in (**a**). Follicles were analyzed at 1 dpi (*n* = 49, 61, 79), 3 dpi (*n* = 28, 49, 27), 7 dpi (*n* = 33, 34, 37) for mock, cisplatin, and cisplatin + GSK126 groups, respectively. **c** Morphologically abnormal primordial follicle ratio at indicated time points. *n* = 4, 4, and 3 biologically independent ovaries at 1, 3, and 7 dpi, respectively. **d** Meiotic maturation of FGOs collected 7 days after cisplatin treatment at PD14, with or without GSK126 co-treatment, followed by in vitro maturation analysis. GVBD (*n* = 6, 6, 7 biologically independent mice) and PBE (*n* = 6, 5, 6) for mock, cisplatin, and cisplatin + GSK126 groups, respectively. **e** Subcellular localization of EZH2 (red) and tubulin (green) in MⅡ oocytes cultured with or without cisplatin (10 μM). Scale bar, 20 μm. **f** H3K27me3 distribution on meiotic chromosomes in MⅡ oocytes cultured with or without cisplatin (10 μM) and GSK126 (0.1 μM). Scale bar, 20 μm. **g** Quantification of GVBD and PBE rates during in vitro maturation of oocytes cultured in the presence or absence of cisplatin (10 μM) and GSK126 (0.1 μM). *n* = 5 biologically independent mice. **h** Immunostaining of the spindle assembly checkpoint protein BUB3 (red) and centromere marker CREST (green) at metaphase. Representative chromosome spreads of MI oocytes cultured for 8 h under mock conditions or for 9.5 h under mock, cisplatin, or cisplatin + GSK126 conditions. Scale bar, 5 μm. **i** Quantification of BUB3 signals normalized to CREST signals in (**h**). Oocytes were analyzed at 8 h mock (*n* = 150); 9.5 h mock (*n* = 116), cisplatin (*n* = 196), and cisplatin + GSK126 (*n* = 119). Mean ± SEM. Statistical significance was assessed using one-way ANOVA without adjustment for multiple comparisons in (**b**–**d**, **g**, **i**).

To assess the early effects of cisplatin exposure on primordial follicle oocytes and to test whether EZH2 inhibition mitigates these early abnormalities, we first examined epigenetic and follicular changes in vivo. PD7 mice treated with cisplatin, with or without the EZH2 inhibitor GSK126, were analyzed at 1, 3, and 7 days post-injection. Cisplatin induced a dynamic increase in H3K27me3 in primordial follicle oocytes, peaking at day 3, which was largely suppressed by GSK126 co-treatment (Fig. 6a, b). Although H3K27me3 levels largely returned to baseline by day 7, histological analysis revealed irreversible follicular damage, with apoptotic and necrotic primordial follicles evident as early as day 1 and aberrant activation becoming apparent by day 3. These abnormalities were significantly reduced in the GSK126 co-treatment group, indicating that EZH2 inhibition mitigated early follicular injury and abnormal activation (Fig. 6c and Supplementary Fig. 7a). Consistently, quantification of absolute follicle numbers showed that cisplatin markedly reduced primordial and total follicle counts, whereas GSK126 co-treatment partially alleviated this loss (Supplementary Fig. 7b, c). We next examined the functional consequences of cisplatin exposure at the growing oocyte stage, focusing on oocytes from secondary follicles. For this purpose, PD14 mice were treated with cisplatin, with or without GSK126, and FGOs were collected seven days later. Nuclear maturation parameters (SN/NSN configuration and GVBD rates) were comparable among groups (Fig. 6d and Supplementary Fig. 7d). However, cisplatin exposure markedly impaired meiotic progression, as reflected by a reduced PBE rate. Co-treatment with GSK126 partially restored PBE rates, supporting a protective effect of EZH2 inhibition (Fig. 6d).

We then examined whether EZH2-mediated chromatin alterations directly affect oocyte maturation under in vitro maturation (IVM) conditions. During normal maturation, chromatin-associated EZH2 in FGOs dispersed into the cytoplasm, leaving low levels on MⅡ chromosomes. In contrast, cisplatin-treated oocytes retained strong chromosomal EZH2 signals together with elevated H3K27me3 (Fig. 6e, f). Cisplatin exposure impaired meiotic progression. Although GVBD rates were comparable between groups, the PBE rate was markedly reduced, and abnormal asymmetric divisions were observed in the cisplatin-treated group (Fig. 6g and Supplementary Fig. 7e). Spindle organization was also disrupted. Whereas most mock MⅡ oocytes formed bipolar spindles with aligned chromosomes, a substantial proportion of cisplatin-treated oocytes exhibited distorted spindles and chromosome misalignment (Supplementary Fig. 7g, h). Pharmacological inhibition of EZH2 with GSK126 did not alter EZH2 protein abundance but normalized H3K27me3 levels (Fig. 6f and Supplementary Fig. 7f). Importantly, GSK126 partially restored PBE rates and improved spindle integrity (Fig. 6g and Supplementary Fig. 7g, h). Consistent with these defects, the spindle assembly checkpoint (SAC) component BUB3 persisted at centromeres in cisplatin-treated oocytes, indicating prolonged SAC activation, which was reversed by GSK126 (Fig. 6h, i). Collectively, these findings indicate that cisplatin induces EZH2-associated H3K27me3 accumulation and abnormalities in primordial follicle oocytes, while also impairing meiotic competence in both growing and fully grown oocytes. EZH2 inhibition mitigates cisplatin-induced H3K27me3 accumulation and early follicular abnormalities and partially improves these meiotic maturation defects (Supplementary Fig. 8).

## Discussion

**Fig. 7:**
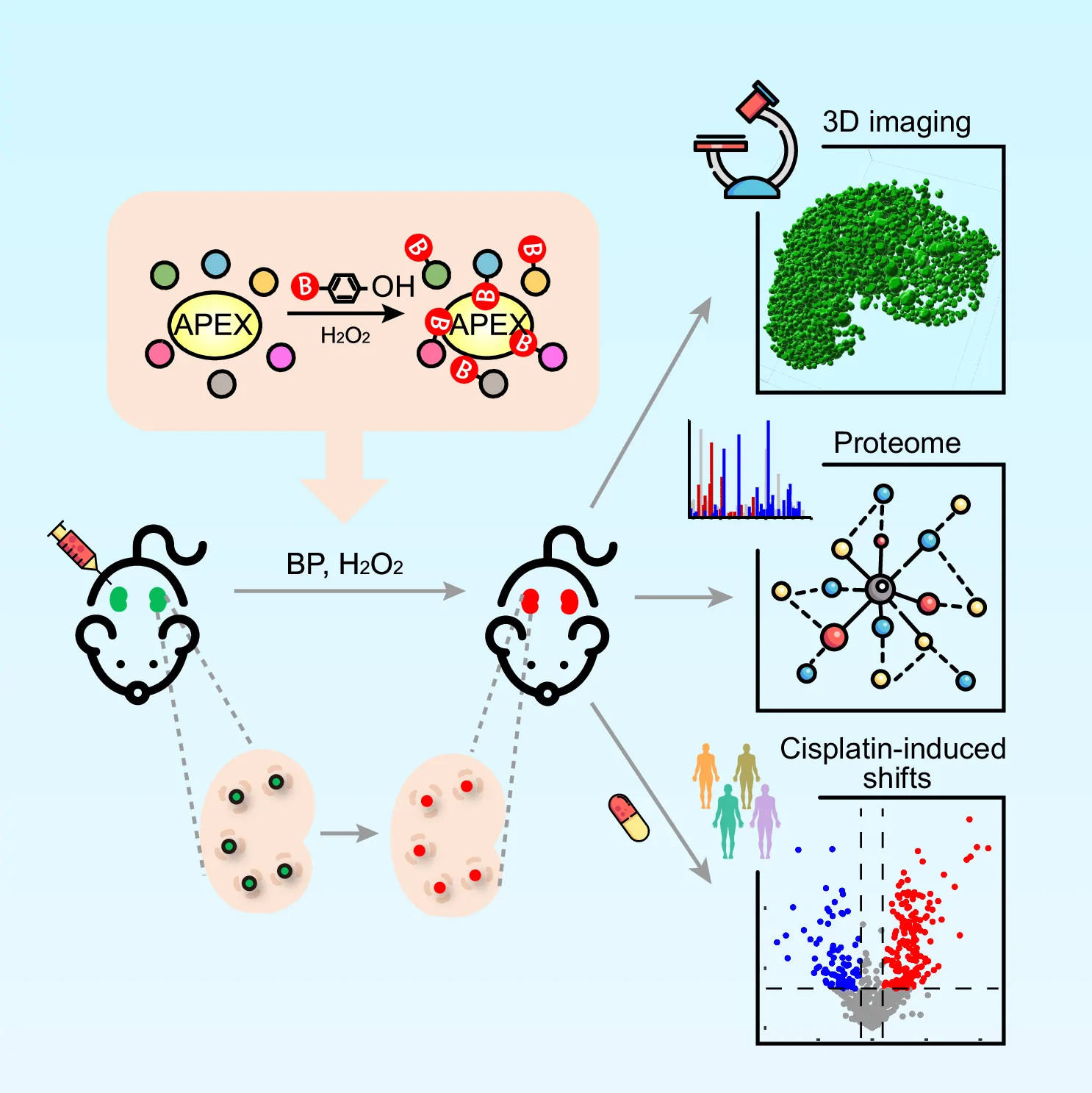
Schematic overview of the in vivo APEX labeling strategy. BP and H₂O₂ were sequentially administered to induce APEX-mediated biotinylation of proteins within oocytes of living mice. The labeled ovaries were subsequently used for 3D imaging, proteomic profiling, and analysis of cisplatin-induced proteome shifts.

Due to the rarity of oocyte samples and technical challenges associated with isolating oocytes from primordial follicles, only the proteins of FGOs, and MⅡ oocytes during meiotic maturation have been explored, leaving the question of which proteins, as well as which associated biological pathways and molecular functions, are expressed in earlier follicle stages. Therefore, a systematic proteomic profile of oocytes in primordial follicles is unavailable but urgently. In this study, we applied APEX2-driven proximal biotinylation for proteome analysis, which was performed in situ in mouse oocytes within the ovaries (Fig. 7). Without ovarian fractionation and oocyte collection, this approach requires a lower input. With the help of germ cell-specific Cre expression, oocytes at early growth stages, *i.e*., the primordial follicle stage, were carried APEX2, and their proteins were efficiently biotinylated. We established a multi-omics mapping platform in mouse oocytes, enabling characterization of both the proteome and transcriptome of in situ oocytes predominantly from primordial follicles. In addition, this transgenic APEX mouse system was advanced in and sensitive to uncover proteome alterations, even with a low dosage of cisplatin and a relatively short duration. Given chemotherapy is one of the common first-line treatments for a variety of cancers, revealing protein shifts induced by cisplatin would render a candidate pool for potential targets to alleviate corresponding cell damages. We identified upregulated 210 oocyte proteins at the primordial follicle stage after cisplatin treatment, including DNA damage repair factors as the primary response. Of note, the histone methylase EZH2 was upregulated in oocytes after cisplatin treatment and was associated with increased H3K27me3 levels in primordial follicle oocytes. GSK126 mitigated cisplatin-induced H3K27me3 accumulation and early follicular abnormalities in PD7 ovaries. In parallel, cisplatin impaired meiotic progression in growing and fully grown oocytes, and this defect was partially improved by GSK126 treatment. However, the maturation defects were observed after direct treatment of oocytes, including PD14 ovary treatment and in vitro FGO maturation, rather than by directly tracing the fate of cisplatin-exposed primordial follicles. It thus remains undemonstrated whether primordial follicles that survive cisplatin exposure at PD7 subsequently develop meiotic competence defects. Long-term follow-up was not technically feasible because PD7 mice treated with cisplatin did not survive to adulthood, likely due to impaired maternal care for stressed litters and systemic toxicity at this early age. Moreover, neonatal ovaries cannot be reliably maintained ex vivo long enough to trace primordial follicles through to the FGO stage in our laboratory conditions. Together, EZH2 could be a clinical target to benefit female cancer patients. It also demonstrates the versatile applications of the APEX2 in vivo labeling approach in detecting dynamic proteomics.

It should be noted that the cisplatin-induced increase in H3K27me3 may not solely reflect epigenetic reprogramming. H3K27me3 and its methyltransferase EZH2/PRC2 have been reported to accumulate at DNA damage sites and participate in DNA damage-associated chromatin responses, including local transcriptional repression and repair-associated chromatin regulation^50^. Therefore, the increased H3K27me3 observed after cisplatin treatment may reflect a DNA damage-associated chromatin response, broader epigenetic dysregulation, or both. Our current data cannot directly distinguish between these mechanisms. Future genome-wide profiling of H3K27me3 together with DNA damage markers, via ChIP-seq or CUT&Tag analyses, in oocytes from different treatment groups may help resolve this question. In our experiments, GSK126 did not aggravate the damage phenotypes but instead improved follicle survival and meiotic progression, suggesting that EZH2 inhibition does not appear to severely compromise essential repair processes under our experimental conditions. The protective effect of GSK126 further suggests that excessive or sustained EZH2 activity / H3K27me3 abundance may be detrimental in cisplatin-induced oocyte injury. We speculate that GSK126 may protect oocytes by limiting excessive EZH2/H3K27me3-mediated chromatin repression after cisplatin-induced DNA damage, thereby preserving a chromatin state more compatible with follicle survival and oocyte maturation. However, the precise protective mechanism of GSK126 in this context remains to be further investigated.

The number of primordial follicles has been considered an indicator of fertility^51,52^, and accurately reflecting oocyte number and status is critical for effective clinical management in women who have surpassed the optimal reproductive age or require chemotherapy. In research settings, ovarian cross-sections have traditionally been used to estimate follicle numbers, but this approach is inherently limited as it only shows granulosa cells surrounding the oocyte in a single plane and does not allow precise follicle counting. Previous studies have addressed this challenge by reconstructing ovarian architecture and quantifying follicles through immunostaining combined with tissue clearing^27–34^. In our work, the APEX mouse model was primarily designed for in vivo protein labeling and identification of specific cell types, with 3D visualization as a complementary feature enabled by EGFP fluorescence. The APEX-EGFP signal, combined with nuclear staining, can be used for 3D in situ visualization without lengthy antibody staining, allowing identification of oocytes and surrounding granulosa cell nuclei, estimation of oocyte and follicle volumes, and assessment of the spatial distribution of primordial follicles in the ovarian cortex^53^. We acknowledge that similar 3D visualization could also be achieved using other Cre-dependent LSL fluorescent reporter mice, such as LSL-tdTomato/Ai9 lines^54^. Compared with antibody-based approaches, such reporter-based imaging is rapid, antibody-free, and compatible with diverse Cre-driver lines, although it necessarily requires genetically engineered mouse models.

There are some approach-associated limitations. Due to the characteristics of the APEX2-driven reaction, some proteins could not be recovered using our approach. Still in an intact cell, as proteins and RNAs could exist in macromolecular complexes, proteins masked by other molecules were unable to be biotinylated. In addition, proteins with lower abundance might be lost in the mass spectrometry analysis. Furthermore, as traditional streptavidin pull-down assay cannot entirely eliminate the risk of false positives, non-biotinylated proteins that strongly interact with actual biotinylated proteins may be co-precipitated, especially during the post-lysis process. Because super-resolution proximity labeling^55^ or direct identification of biotinylation sites at the peptide level was not performed, the dataset inherently carries the risk of including indirect interactors, and it was not possible to uncover which residues in the detected proteins were biotinylated under the current resolution, limiting the ability to provide steric information and insights into protein folding. In addition, because the actual concentration of probes, for both BP and H_2_O_2_, reaching the ovary during in vivo labeling is influenced by the administered dose and pharmacokinetics, the labeling outcomes may be subject to potential variation without dose- and time-dependent optimization. Although we set a stringent age limit at PD7 to minimize multi-stage oocyte incorporation, primary and secondary follicles did exist in PD7 mouse ovaries. Moreover, it is possible that the proteome of primordial follicle oocytes varies across developmental or physiological stages, such as between early postnatal and adult ovaries, due to differences in the hormonal environment. Comparison of proteomes directed by multiple Cre-driven APEX2, using two oocyte-specific Cres, *Gdf9-*Cre and *Zp3*-Cre, as examples, could help remove protein contaminants that are also expressed in oocytes across various follicle stages and establish a more comprehensive and precise primordial follicle stage-specific proteome.

Collectively, we established an in vivo APEX-labeling system enabling the investigation of ovary at proteomic and transcriptomic levels, and the visualization of the 3D ovary with accurately quantitively analyzing oocytes at different developmental stages. Protein dynamic changes induced by cisplatin exposure provided protein candidates to attenuate, if not rescue, chemotherapy-induced oocyte damage. We believe that our findings will serve as a reference for studies on female reproduction and provide additional target lists for female fertility protection and female cancer chemotherapy.

## Methods

### Animals

All mice were handled according to the Animal Research Committee guidelines of Zhejiang University (approval #ZJU20250080). All mouse strains had a C57BL/6J genetic background. The *APEX*^*flox/flox*^ mice, established to enable conditional expression of APEX2–EGFP following Cre recombination, was generated and is maintained in our laboratory^56^. These mice were crossed with *Gdf9*-Cre transgenic mice, originally derived from Austin J. Cooney’s lab^21^, to generate *Gdf9*-Cre; *APEX*^*flox/+*^ offspring (referred to as APEX^oocyte^ mice), in which APEX2 is selectively expressed in oocytes, including those at the primordial follicle stage. Mice were maintained under SPF conditions in a controlled environment of 20–22 °C, with a 12 h/12 h light/dark cycle, 50–70% humidity, and food and water provided *ad libitum*. For oocyte-related experiments, female mice were exclusively used as donors, since male mice were not applicable to this experimental model. For fertility assessment, 8-week-old female mice for both genotypes were mated with 10-week-old male mice. For cisplatin-related experiments, PD7 or PD14 mice received intraperitoneal injection of cisplatin (2 mg/kg) with or without co-administration of the EZH2 inhibitor GSK126 (1 mg/kg). Ovaries were collected at the indicated time points (day 1, day 3, and day 7 after treatment) for histological, immunohistochemical, and molecular analyses.

### Superovulation

Female mice at postnatal day 21–23 (PD21–23) were intraperitoneally injected with 5 IU of pregnant mare serum gonadotropin (PMSG, Ningbo Sansheng Pharmaceutical Co., Ltd., P. R. China) to stimulate preovulatory follicle development. FGOs and COCs were collected as described in oocyte and cumulus-oocyte complex collection and in vitro culture. After 48 h, mice were further injected with 5 IU human chorionic gonadotropin (hCG, Ningbo Sansheng Pharmaceutical Co., Ltd., P. R. China) to stimulate ovulation. Oocytes at metaphase Ⅱ(MⅡ) were then collected as described in oocyte and cumulus-oocyte complex collection and in vitro culture.

### Oocyte and cumulus-oocyte complex collection and in vitro culture

Female mice treated with the indicated hormones as described in superovulation were humanely euthanized. FGOs were harvested from 48 h post PMSG-treated mice in M2 medium (Sigma-Aldrich, M7167, USA) plus 1% penicillin-streptomycin solution (Hyclone, SV30010, USA) and cultured in mini-drops of M16 medium (Sigma-Aldrich, M7292) in a humidified incubator, at 37 °C in 5% (v/v) CO_2_ atmosphere. The drops were covered with mineral oil. For cisplatin application at the FGO stage, cisplatin (10 µM) with or without GSK126 (0.1 µM) were added to the M16 culture medium prior to oocyte incubation. After 4 h and 16 h of treatment, oocytes were collected for assessment of GVBD, first PBE, and molecular analyses, respectively. MⅡs were surgically removed from the oviducts of 16 h post hCG-treated mice in M2 medium.

### Three-dimensional immunofluorescence analysis of the whole ovary

The immunostaining of ovaries was performed according to the protocol described previously^57^. Ovaries were harvested from PD7 female mice and fixed overnight at 4 °C using BD Cytofix on a shaker. The fixed ovaries were washed for 1 h with washing buffer (1× PBS supplemented with 0.3% Triton X-100 and 0.5% 1-thioglycerol) three times. Subsequently, the ovaries were blocked and permeabilized at 37 °C for 24 h with blocking buffer (1× PBS plus 0.3% Triton X-100, 1% BSA, and 1% normal mouse serum). After that, the ovaries were incubated with hochest 33342 (10 µg/mL) at 37 °C for 4 d. After three more washes, the ovaries were cleared by immersion in clearing solution (2.75 mL of 40% N-methyl acetamide, 4 g of Histodenz, and 5 μL Triton X-100) with a refractive index of 1.495-1.505, for 24 h at room temperature before being imaged. The fluorescence images were captured using Lightsheet Z.1 microscope with a 5× detection objective (NA = 1.0). The raw image stacks were processed by dual-side fusion and converted to Imaris-readable files using Imaris File Converter (version 10.1.1, Bitplane). The 3D reconstruction and quantitative analyses were performed using Imaris (version 10.1.1, Bitplane, RRID: SCR_007370). For follicle quantification, *Spot* modeling was applied to the EGFP fluorescence channel, as it provided reliable object-based counting without merging adjacent oocytes, a common issue encountered in *Surface* reconstruction. The expected *Spot* diameters were set to 18 μm for primordial follicular oocytes and 45 μm for growing oocytes to ensure accurate and efficient follicle quantification. For the measurement of oocyte diameter, follicle volume, and granulosa-cell number, manual *Contour* definition was combined with automated *Surface* and *Spot* modeling in Imaris. Follicular boundaries were delineated based on nuclear (Hoechst 33342) staining, and within each manually defined contour, EGFP signals were used for oocyte *Surface* modeling, while nuclear signals were used for granulosa-cell *Spot* modeling. This workflow enabled precise measurement of follicular morphology and cellular composition across different developmental stages. The ovarian cortex was defined morphologically as the peripheral zone enriched in compact primordial follicles, with the medulla designated as the inner region containing larger, distinct growing follicles. The cortical–medullary transition was identified at the point where the distribution of primordial follicles became discontinuous.

### Immunofluorescence

Oocytes were fixed in 4% paraformaldehyde (PFA, Sigma-Aldrich, 158127, USA) in PBS at room temperature (RT) for 30 min. They were permeabilized in PBS containing 0.5% Triton X-100 at RT for 20 min and blocked with 1% bovine serum albumin (BSA, A3311, Sangon Biotech, Shanghai, China) at RT for 30 min. For determination of APEX2 expression, oocytes were incubated with V5-tag (D3H8Q) primary antibody (Cell Signaling Technology, 13202, USA) at 4 °C overnight and further labeled with Alexa Fluor Cy3-conjugated secondary antibody and 4’, 6-diamidino-2-phenylindole (DAPI) at RT for 30 min. For examination of APEX2-driven biotinylation, oocytes were incubated with Cy3-conjugated streptavidin (Sigma-Aldrich, S6402) and DAPI at RT for 20 min. Oocytes were imaged using a Zeiss LSM710 confocal microscope (Germany). Quantification of fluorescence intensity was performed using ImageJ version 2.16.0/1.54p (National Institutes of Health, USA). The mean gray value within the region of interest (ROI) was measured after background subtraction. BUB3 immunofluorescence intensity was quantified and normalized to CREST signals to account for variations in staining and imaging conditions.

### Chromosome spreading

Zona pellucida-free oocytes 8 h and 9.5 h post meiotic resumption were fixed in a solution containing 1% PFA, 0.15% Triton-X100, and 3 mM dithiothreitol (DTT, Sigma-Aldrich, 43819) for 30 min at RT and air dried. Immunofluorescent staining was performed as described in Immunofluorescence.

### Histological analysis

Ovaries were collected and fixed in 4% formalin in PBS at 4 °C overnight, processed, embedded in paraffin and sectioned at 5 μm. For immunofluorescence (IF), ovarian sections were probed with indicated primary antibodies as listed in Supplementary Data 7 and labeled with Alexa Fluor 488-, Fluor 555- or Fluor 647-conjugated secondary antibody and DAPI. For immunohistochemistry (IHC), ovarian sections were probed and stained according to standard protocols. For Hematoxylin and Eosin (H&E) staining, ovarian sections were treated according to standard protocols (Mabwell Bioscience, co. Ltd., P.R. China). Digital images were acquired using an epifluorescence microscope (Nikon Eclipse 80i, Japan) with 4 - 100× objectives. Quantification of staining intensity for both IF and IHC images was performed using ImageJ. For each section, the mean gray value within the defined region of interest (ROI) was measured after background subtraction.

### Follicle counts

Ovaries were serially sectioned at 7 μm and stained with H&E. Amounts of follicles were determined by recording the numbers of oocyte nuclei on all sections of each ovary. The stages of the follicles were determined following these criteria: primordial follicles, small oocytes surrounded by a single layer of flat pre-granulosa cells; primary follicles, medium-sized oocytes surrounded by a single layer of cubical granulosa cells; secondary follicles, large oocytes surrounded by more than two layers of granulosa cells; antral follicles, large follicles with the presence of follicle cavities.

### TUNEL assay

TUNEL assays were performed on paraffin-embedded sections using the ApopTag Plus Peroxidase In Situ Apoptosis Detection Kit (Serological Corporation, Norcross, GA, USA) following a modified protocol according to the manufacturer’s instructions. In brief, 0.1% Trypsin-EDTA (Gibco, 25200) was applied for digestion at 25 °C for 2 min instead of proteinase K (Sigma-Aldrich, 2308). The slides were further treated with 3% (v/v) H_2_O_2_ at 25 °C for 5 min, Equilibration Buffer at 25 °C for 10 s, and subjected to TdT enzyme reaction at 37 °C for 1 h. The reaction was terminated by the addition of Stop/Wash buffer and rest at 25 °C for 10 min. After rinsing in 1×PBS, the samples were blocked and incubated with the indicated antibodies as described in Histological analysis. FITC-anti digoxigenin was applied to detect the deoxynucleotide-conjugated 3′ -hydroxyl terminus of DNA breaks.

### In situ APEX2-driven biotinylation

The APEX2-driven biotinylation was carried out using the previous protocols with minor modifications^14,58^. For labeling in oocytes, FGOs and COCs were collected from APEX^oocyte^ females and incubated in M2 drops with 500 μM biotin-phenol (Adipogen, CDX-B0270) at 37 °C for 30 min. The biotinylation was initiated when FGOs and COCs were transferred into M2 drops containing 500 μM biotin-phenol and 1 mM H_2_O_2_ using a mouth pipette. The reaction was maintained at RT for 1 min and was quenched by immediately transferring the oocytes into quench buffer containing 10 mM sodium ascorbate (Sangon Biotech, A500830), 5 mM Trolox (Sigma-Aldrich, 238813), and 10 mM NaN_3_ in DPBS and rinsed three times. The FGOs and COCs were subjected to further immunofluorescence analysis. For in situ labeling of whole ovaries, we injected the reaction substrate of 500 μM biotin-phenol (BP) dissolved in PBS by i.p.. After 1 hour of incubation, the APEX reaction was triggered by i.p. of 1 mM H₂O₂, and the ovaries were collected 5 minutes after injection for downstream processing. The tissues were then either rapidly lysed for biotinylated protein and mRNA enrichment or fixed for histological staining. *APEX*^*flox/+*^ females served as the control group.

### Biotinylated protein enrichment

Ovaries isolated from APEX^oocyte^ mice or *APEX*^*flox/+*^ females after in vivo APEX labeling, and subsequently lysed in RIPA lysis buffer containing 50 mM Tris-HCl, pH 7.5, 150 mM NaCl, 1% SDS (w/v), 0.5% sodium deoxycholate (w/v, Sigma-Aldrich, D6750), 1% Triton X-100 (v/v), 5 mM Trolox, 10 mM NaN_3_, 10 mM sodium ascorbate and 1× PMSF using an ultrasonic homogenizer as previously described^58^. Proteins were precipitated with 9× methanol (v/v) at -80 °C overnight and resuspended in a modified RIPA lysis buffer with 0.2% SDS. Accordingly, protein precipitates extracted from 16 ovaries in PD7 mice were resolved in 100 μL RIPA lysis buffer with 1% SDS, denatured for 5 min at 95 °C, and then diluted to 500 μL with SDS at a final concentration of 0.2%. 25 μL Streptavidin Magnetic Beads (Pierce, 88817) were added and the incubation was carried out for 4 h at 4 °C. Protein-bound beads were gathered using a magnet and serially rinsed twice with 1 mL RIPA lysis buffer with 0.2% SDS, twice with 1 M KCl, twice with 0.1 M Na_2_CO_3_, twice with 2 M Urea and twice with RIPA lysis buffer with 0.2% SDS. Biotinylated proteins were eluted by SDS sample buffer with 2 mM biotin (Sigma, B4501) and 20 mM DTT for 10 min at 95 °C.

### Western blotting analysis

Biotinylated proteins were separated through SDS-PAGE, electrophoretically transferred to PVDF membranes (Millipore Crop., Bedford, MA, IPVH00010, USA) and blocked in TBST containing 1.5 mM NaCl and 1 mM Tris with 5% BSA for 30 min at RT. For HRP blot analysis, the membranes were probed with Streptavidin-HRP (Cell Signaling Technology, 3999) for 1 h at RT. The membranes were then washed three times with TBST and the biotinylated proteins were detected using the Super Signal West Femto maximum sensitivity substrate (Thermo Fisher Scientific lnc., Waltham, MA, USA). For confirming oocyte proteome at the primordial follicle stage, the membranes were probed with primary antibodies overnight at 4 °C, washed three times with TBST, and subjected to incubation with corresponding HRP-linked secondary antibodies for 1 h at RT and bound proteins were detected. The antibodies and dilution factors used are listed in Supplementary Data 7. The original uncropped western blot images are provided in Source Data file.

### Mass spectrometry analysis

Samples were separated on 10% SDS-PAGE gels. Gels were stained with Coomassie brilliant blue R-250 (Bio Basic Canada Inc., CB0037). After washing, gels were reduced with DTT, alkylated with 2-iodoacetamide (IAA, Sigma-Aldrich, I6125), and digested with trypsin overnight at 37 °C. The resulting peptide mixtures were extracted, dried, and reconstituted in 2% acetonitrile (v/v, ACN, ThermoFisher, 51101) and 0.1% formic acid (v/v, ThermoFisher, 85178). Samples were analyzed by Obitrap mass spectrometry (HFX, ThermoFisher Scientific). Raw data were processed using MaxQuant software version 1.6.10.43 (Max Planck Institute for Biochemistry, Martinsried, Germany), and proteins were identified against Swiss-Prot house mouse reference proteome using 10 ppm peptide tolerance. The peptide information from the *APEX*^*flox/+*^, APEX^oocyte^, and cisplatin-treated APEX^oocyte^ groups is available in Supplementary Data 8.

### H₂O₂-induced differential proteomic analysis

To assess the potential impact of H₂O₂ treatment on the ovarian proteome, PD7 ovaries were collected from mice treated with H₂O₂ alone under the same in vivo conditions used for APEX labeling, except that BP was omitted. PD7 female mice were injected intraperitoneally with 1 mM H₂O₂ at 10 μL/g body weight, and ovaries were collected 5 min after injection for downstream proteomic analysis. Control ovaries from PBS-treated mice were collected in parallel. Each group consisted of four ovaries, with two independent biological replicates. Tissues were rapidly lysed and processed for LC–MS/MS using the same workflow, filtering criteria, and statistical analysis applied to the above main proteomic datasets. Differential proteomic comparisons between H₂O₂-treated and control groups were performed to evaluate potential changes induced by H₂O₂ treatment.

### RNA extraction and biotinylated RNA enrichment

RNAs were extracted from ovaries using the Invitrogen Ambio TRIzol reagent (Thermo Fisher Scientific) according to manufacturer’s instructions. Turbo^TM^ DNase (Thermo Fisher Scientific) was applied to remove DNAs. RNAs were further subjected to biotinylated RNA enrichment and reverse transcription. To enrich biotinylated RNAs, streptavidin-biotin interaction-based affinity pulldown was performed using Streptavidin Magnetic Beads as previously described with minor modifications^40^. In brief, the beads were incubated with the RNAs extracted from APEX^oocyte^ mouse ovaries at a ratio of 10 μL beads to 25 μg RNAs. The beads were washed with B&W buffer containing 5 mM Tris-HCl [pH 7.5], 0.5 mM EDTA, 1 M NaCl, and 0.1% Tween-20 for 3 times, Solution A with 0.1 M NaOH and 50 mM NaCl for 2 times, and 0.1 M NaCl for 1 time. After being suspended by 150 μL 0.1 M NaCl, the beads were mixed with RNAs dissolved in 150 μL RNase-free H_2_O. The incubation was carried out for 4 h at 4 °C on a rotator. The beads were collected by a magnet, and the supernatant was discarded. After washing with B&W buffer 3 times, the beads were resuspended with 54 μL RNase-free H_2_O. Biotinylated RNAs were eluted by a proteinase K mixture with 2% N-lauryl sarcosine sodium (w/v, Sigma-Aldrich, 61743), 10 mM EDTA, 5 mM DTT, 1 U/μL RRI (TAKARA, 2313), and 2 μg/μL proteinase K in PBS [pH 7.4] sitting for 1 h at 42 °C and shaking for 2 h at 55 °C. The elutions were extracted by Phenol:Chloroform (1:1, v/v), and biotinylated RNAs were precipitated in an ethanol-based buffer containing 0.3 M sodium acetate [pH 5.5] (Sigma-Aldrich, S2889), 3 μg/μL linear acrylamide (Invitrogen, AM9520), and 2.5 volumes of 100% ethanol overnight at -20 °C. The precipitate was resuspended in RNase-free H_2_O for dot blotting analysis, reverse transcription and quantitative real-time PCR and transcriptome analysis as follows.

### Biotinylated RNA dot blotting

After the indicated treatment, RNAs were extracted from APEX^oocyte^ and *APEX*^*flox/+*^ ovaries by the TRIzol reagent and followed by Turbo^TM^ DNase application. The purified RNAs were denatured for 5 min at 75 °C, and chilled for 1 min on ice. ~500 ng RNA samples were spotted onto a nitrocellulose membrane. After drying in the air, the membrane was blocked with TBST buffer supplemented with 5% BSA and extensively washed with TBST. Then, the membrane was incubated with HRP-conjugated streptavidin (1:2000) for 1 h at room temperature. The dot blot was detected by enhanced chemiluminescence detection system. For the RNase digestion, the RNA samples were treated with RNase for 30 min at 25 °C, followed by purification and subjected to dot blotting.

### Reverse transcription and quantitative real-time PCR

RNAs extracted from granulosa cells and the tissues were reverse-transcribed, and the cDNA abundance was evaluated using quantitative PCR (qPCR). The RNAs were reverse-transcribed with random primers using M-MLV Reverse Transcriptase (Invitrogen, 28025). For oocyte samples and biotinylated RNAs enriched from APEX^oocyte^ mice, PrimeScript^TM^ II Reverse Transcriptase was employed (Takara Bio, Shiga, Japan) according to the manufacturer’s instructions. The cDNAs were subjected to qPCR using Power SYBR Green PCR Master Mix (Applied Biosystems, Life Technologies) and a Bio-Rad CFX96 Touch Real-Time PCR system. For each experiment, qPCR was performed in triplicate. The corresponding primers of the indicated transcripts are listed in Supplementary Data 9.

### APEX-seq library preparation and transcriptome analysis

The biotinylated RNAs from APEX^oocyte^ mice were subjected to RNA-seq using the Smart-seq2 method with minor modifications. In brief, RNAs were diluted to 0.2 ng/μL and 2.5 μL samples were performed library constructions. The samples were incubated with oligo(dT) primers (see the corresponding primer sequence in Supplementary Data 9) a deoxynucleoside triphosphate mixture for 3 min at 72 °C, and cDNA pools were obtained by Smart-seq2 reverse transcription reactions. After the first-strand reaction, the cDNAs were pre-amplified with limited cycles (~ 12 cycles). Sequencing libraries were then constructed with 0.5 ng cDNA using the TruePrep DNA Library Prep Kit V2 for Illumina (TD503; Vazyme, Nanjing, China) according to the manufacturer’s instructions. Barcoded libraries were pooled and sequenced on the Illumina NovaSeq 6000 platform in the 151 bp paired-end mode. Raw reads were trimmed to remove low-quality bases and adaptor sequences using Trim Galore version 0.6.7. Reads were further mapped to the mouse (mm9) genomes using STAR version 2.7.10a^59^. Uniquely mapped reads were applied for gene expression quantification using FeatureCounts version 2.0.2^60^. Biotinylated gene transcript abundance was further analyzed using R (version 4.3.2) and the DESeq2 package (version 1.42.0). Primordial follicle oocyte-enriched mRNAs were defined as genes with a linear fold change (APEX^oocyte^/*APEX*^*flox/+*^) > 1.5 and *P* < 0.05, together with genes detected only in the APEX^oocyte^ group. Transcripts per million mapped reads (TPM) were calculated to validate gene expression and normalized to gene length and sequencing depth.

### Alternative splicing event analysis

The alternative splicing events (ASEs) between the primordial follicle oocytes and FGOs were analyzed using replicate multivariate analysis of transcript splicing (rMATS, version 4.1.2)^61^. Five categories of ASEs, including skipped exons (SEs), alternative-5′ spliced sites (A5SSs), alternative-3′ spliced sites (A3SSs), mutually exclusive exons (MXEs) and retained introns (RIs) were considered. Only events with false discovery rate (FDR) < 0.05 and inclusion level differences over 0.1 or below -0.1 were designated significant ASEs. The ASEs are listed in Supplementary Data 4.

### Bioinformatics analysis

Enrichment analysis of primordial follicular oocyte proteins was performed using R (version 4.3.2) with the clusterProfiler package (version 4.10.0; RRID: SCR_016884), WebGestalt (WEB-based Gene SeT AnaLysis Toolkit, https://www.webgestalt.org/) and a Cytoscape plugin, CluGO (version 2.5.10). *P* values for the representative terms shown in the present study were adjusted with the Benjamini-Hochberg procedure. The protein-protein interaction (PPI) network of primordial follicular oocyte proteins was constructed and visualized by STRING database (version 12.0; RRID: SCR_005223) and Cytoscape software (version 3.10.1; RRID: SCR_003032), respectively. Network clustering was performed using the MCODE plugin (version 2.0.3).

### Statistical analyses

Statistical analyses were performed using GraphPad Prism (version 10.2.3, GraphPad Software). Unless otherwise specified, all experiments were conducted in three biologically independent replicates. Means and standard error of mean (SEM) were plotted. Student’s *t*-test or one-way ANOVA was used for statistical analyses. All statistical tests were two-tailed unless otherwise stated. *P* < 0.05 was considered statistically significant. Statistical details are included in figure legends.

### Reporting summary

Further information on research design is available in the Nature Portfolio Reporting Summary linked to this article.

## Supplementary information

Supplementary Information

Description of Additional Supplementary Files

Dataset 1

Dataset 2

Dataset 3

Dataset 4

Dataset 5

Dataset 6

Dataset 7

Dataset 8

Dataset 9

Reporting Summary

Transparent Peer Review file

## Source data

Source Data

## Data Availability

The RNA-seq data generated from early follicle oocytes in this study have been deposited in the NCBI Gene Expression Omnibus (GEO) under accession code GSE248745. The APEX-based proteomics data generated in this study, including raw mass spectrometry files and search results, have been deposited in the ProteomeXchange Consortium via the iProX partner repository under accession codes PXD074986 and IPX0015821000. The previously published FGO proteomics datasets used for comparison are available through ProteomeXchange under accession codes PXD038447 (https://www.iprox.cn/page/project.html?id=IPX0004832000) and PXD043935 (https://www.iprox.cn/page/project.html?id=IPX0006778000), and as Supplementary Data associated with the original publication^15^. The single-cell ovarian RNA-seq dataset used for analysis of primordial follicle assembly is available in GEO under accession code GSE134339^41^. The oocyte RNA-seq dataset from later developmental stages is available in GEO under accession code GSE135787^42^. The previously published gene-expression datasets used to compare cisplatin-induced transcriptional responses in tumor cells are available as Supplementary Data associated with the original publications^48,49^. Source data are provided with this paper.

## Code availability

No custom code was developed for this study. All statistical analyses and custom scripts used for omics data analysis in this study are provided in the “Methods” section.
