# Supplementary material for: In vivo labeling–based proteomic analysis of early follicle oocytes and cisplatin-induced alterations in mice
Source: Nat Commun. 2026 Aug 15;17:9815. doi: 10.1038/s41467-026-76665-3 (PMC13574812; doi:10.1038/s41467-026-76665-3)
Supplement: Supplementary file 1 [file 41467_2026_76665_MOESM12_ESM.pdf]

nature portfolio

Corresponding author(s): Mo Li

Last updated by author(s): Jul 14, 2026

Reporting Summary

Nature Portfolio wishes to improve the reproducibility of the work that we publish. This form provides structure for consistency and transparency in reporting. For further information on Nature Portfolio policies, see our Editorial Policies and the Editorial Policy Checklist.

Statistics

For all statistical analyses, confirm that the following items are present in the figure legend, table legend, main text, or Methods section.

n/a	Confirmed	
[ ]	[x]	The exact sample size (*n*) for each experimental group/condition, given as a discrete number and unit of measurement
[ ]	[x]	A statement on whether measurements were taken from distinct samples or whether the same sample was measured repeatedly
[ ]	[x]	The statistical test(s) used AND whether they are one- or two-sided<br>*Only common tests should be described solely by name; describe more complex techniques in the Methods section.*
[x]	[ ]	A description of all covariates tested
[x]	[ ]	A description of any assumptions or corrections, such as tests of normality and adjustment for multiple comparisons
[ ]	[x]	A full description of the statistical parameters including central tendency (e.g. means) or other basic estimates (e.g. regression coefficient) AND variation (e.g. standard deviation) or associated estimates of uncertainty (e.g. confidence intervals)
[ ]	[x]	For null hypothesis testing, the test statistic (e.g. *F*, *t*, *r*) with confidence intervals, effect sizes, degrees of freedom and *P* value noted<br>*Give P values as exact values whenever suitable.*
[x]	[ ]	For Bayesian analysis, information on the choice of priors and Markov chain Monte Carlo settings
[x]	[ ]	For hierarchical and complex designs, identification of the appropriate level for tests and full reporting of outcomes
[x]	[ ]	Estimates of effect sizes (e.g. Cohen's *d*, Pearson's *r*), indicating how they were calculated

Our web collection on statistics for biologists contains articles on many of the points above.

Software and code

Policy information about availability of computer code

Data collection	Imaging data were acquired using a Lightsheet Z.1 microscope (Zeiss) and a Zeiss LSM710 confocal microscope. Mass spectrometry data were generated using an Orbitrap HFX mass spectrometer (Thermo Fisher Scientific). Sequencing data were generated on an Illumina NovaSeq 6000 platform according to the manufacturers' instructions.
Data analysis	Proteomics raw data were processed using MaxQuant (version 1.6.10.43). RNA-seq data processing and analysis were performed using Trim Galore (version 0.6.7), STAR (version 2.7.10a), FeatureCounts (version 2.0.2), R (version 4.3.2), and DESeq2 (version 1.42.0). Alternative splicing events were analyzed using rMATS (version 4.1.2). Enrichment analyses were conducted using clusterProfiler (version 4.10.0), WebGestalt, and ClueGO (version 2.5.10) in Cytoscape. Protein–protein interaction networks were constructed using STRING (version 12.0) and visualized using Cytoscape (version 3.10.1); network clustering was performed using MCODE (version 2.0.3). Image quantification was performed using ImageJ (version 2.16.0/1.54p), and statistical analyses were conducted using GraphPad Prism (version 10.2.3).

For manuscripts utilizing custom algorithms or software that are central to the research but not yet described in published literature, software must be made available to editors and reviewers. We strongly encourage code deposition in a community repository (e.g. GitHub). See the Nature Portfolio guidelines for submitting code & software for further information.

Data

Policy information about availability of data

All manuscripts must include a data availability statement. This statement should provide the following information, where applicable:

- Accession codes, unique identifiers, or web links for publicly available datasets
- A description of any restrictions on data availability
- For clinical datasets or third party data, please ensure that the statement adheres to our policy

The RNA-seq data generated from early follicle oocytes in this study have been deposited in the NCBI Gene Expression Omnibus (GEO) under accession code GSE248745 (https://www.ncbi.nlm.nih.gov/geo/query/acc.cgi?acc=GSE248745). The APEX-based proteomics data generated in this study, including raw mass spectrometry files and search results, have been deposited in the ProteomeXchange Consortium via the iProX partner repository under accession codes PXD074986 (https://proteomecentral.proteomexchange.org/cgi/GetDataset?ID=PXD074986) and IPX0015821000 (https://www.iprox.cn/page/project.html?id=IPX0015821000). The previously published FGO proteomics datasets used for comparison are available through ProteomeXchange under accession codes PXD038447 (https://www.iprox.cn/page/project.html?id=IPX0004832000) and PXD043935 (https://www.iprox.cn/page/project.html?id=IPX0006778000), and as Supplementary Data associated with the original publication. The single-cell ovarian RNA-seq dataset used for analysis of primordial follicle assembly is available in GEO under accession code GSE134339 (https://www.ncbi.nlm.nih.gov/geo/query/acc.cgi?acc=GSE134339). The oocyte RNA-seq dataset from later developmental stages is available in GEO under accession code GSE135787 (https://www.ncbi.nlm.nih.gov/geo/query/acc.cgi?acc=GSE135787). The previously published gene-expression datasets used to compare cisplatin-induced transcriptional responses in tumor cells are available as Supplementary Data associated with the original publications. Source Data are provided with this paper.

Research involving human participants, their data, or biological material

Policy information about studies with human participants or human data. See also policy information about sex, gender (identity/presentation), and sexual orientation and race, ethnicity and racism.

Reporting on sex and gender	This study did not involve human participants. Therefore, sex and gender were not considered in the study design. All animal experiments were performed using female mice, as the study focused on ovarian and oocyte biology.
Reporting on race, ethnicity, or other socially relevant groupings	This study did not involve human participants, and therefore race, ethnicity, or other socially relevant groupings were not considered in the study design or analyses.
Population characteristics	Not applicable, as this study did not involve human participants.
Recruitment	Not applicable, as this study did not involve human participants.
Ethics oversight	This study did not involve human participants. All animal experiments were conducted in accordance with the guidelines of the Animal Research Committee of Zhejiang University, which approved the experimental protocols.

Note that full information on the approval of the study protocol must also be provided in the manuscript.

Field-specific reporting

Please select the one below that is the best fit for your research. If you are not sure, read the appropriate sections before making your selection.

[x] Life sciences [ ] Behavioural & social sciences [ ] Ecological, evolutionary & environmental sciences

For a reference copy of the document with all sections, see nature.com/documents/nr-reporting-summary-flat.pdf

Life sciences study design

All studies must disclose on these points even when the disclosure is negative.

Sample size	No statistical methods were used to predetermine sample size. Sample sizes were chosen based on previous studies in the field and our experience with similar experimental systems. The numbers of biological replicates and oocytes analyzed are indicated in the figure legends. These sample sizes are commonly used in oocyte and reproductive biology research and were sufficient to detect reproducible differences between groups.
Data exclusions	No data were excluded from the analyses. All inclusion and exclusion criteria were defined prior to data analysis.
Replication	All experiments were performed with at least three biologically independent replicates unless otherwise specified. The key findings were independently reproduced, and representative results are shown.
Randomization	Mice were randomly assigned to experimental groups where applicable. Oocytes and samples were processed in parallel under identical experimental conditions to minimize potential bias.
Blinding	Investigators were not blinded to group allocation during data collection and analysis, as objective quantitative methods (e.g., automated image analysis and software-based quantification) were used.

Reporting for specific materials, systems and methods

We require information from authors about some types of materials, experimental systems and methods used in many studies. Here, indicate whether each material, system or method listed is relevant to your study. If you are not sure if a list item applies to your research, read the appropriate section before selecting a response.

Materials & experimental systems

n/a	Involved in the study
☐	☒ Antibodies
☒	☐ Eukaryotic cell lines
☒	☐ Palaeontology and archaeology
☐	☒ Animals and other organisms
☒	☐ Clinical data
☒	☐ Dual use research of concern
☒	☐ Plants

Methods

n/a	Involved in the study
☒	☐ ChIP-seq
☒	☐ Flow cytometry
☒	☐ MRI-based neuroimaging

Antibodies

Antibodies used

Protein/Target name Manufacture (catalogue number) Application (working dilution)
V5 Cell Signaling Technology (13202) IF (1:400)
Cleaved Caspase-3 (CC3) Cell Signaling Technology (9664) IHC (1:200)
DDX4 Abcam (ab13840) IF (1:200); western blot (1:1000)
ACTIN Thermo Fisher Scientific (PA1-22167) western blot (1:1000)
DDB1 Abcam (ab9194) western blot (1:2000)
YTHDF2 ProteinTech (24744) western blot (1:1000)
EIF4E ProteinTech (11149) western blot (1:1000); IHC (1:300)
LAMP1 Santa cruz (20011) western blot (1:1000)
CDC27 ProteinTech (10918) western blot (1:1000); IHC (1:200)
BRD1 Abcam (ab181060) IHC (1:100)
PBRM1 ABclonal (A0334) IHC (1:200)
H3K14ac Cell Signaling Technology (7627) IF (1:200)
TBK1 ABclonal (A3458) IHC (1:200)
AAK1 Abcam (ab134971) IHC (1:200)
BAG6 ProteinTech (26417) IHC (1:200)
MAEA ProteinTech (28363) IHC (1:100)
CTNNBL1 ProteinTech (13665) IHC (1:200)
MBNL2 ABclonal (A12838) IHC (1:400)
U2AF1 ABclonal (A1046) IHC (1:200)
ATM Cell Signaling Technology (2873) IF (1:200)
RPA2 Abcam (ab76420) IF (1:200)
RAD51 Abcam (ab63801) IF (1:100)
XRCC1 Cell Signaling Technology (2735) IF (1:200)
S9.6 MERCK (1095) IF (1:100)
EZH2 Cell Signaling Technology (5246) IF (1:200); western blot (1:1000)
H3K27me3 Cell Signaling Technology (9733) IF (1:400)
RNase H2A ProteinTech (16132) western blot (1:1000)
GAPDH ProteinTech (60004) western blot (1:1000)
BUB3 Santa cruz (376506) IF (1:100)
CREST Fitzgerald Industries International (70R-21494) IF (1:100)

Validation

Protein/Target name Website link
V5 https://www.cellsignal.com/products/primary-antibodies/v5-tag-d3h8q-rabbit-mab/13202
Cleaved Caspase-3 (CC3) https://www.cellsignal.cn/products/primary-antibodies/cleaved-caspase-3-asp175-5a1e-rabbit-monoclonal-antibody/9664
DDX4 https://www.abcam.cn/products/primary-antibodies/ddx4--mvh-antibody-ab13840.html
ACTIN https://www.thermofisher.cn/cn/zh/antibody/product/beta-Actin-Antibody-Polyclonal/PA1-21167
DDB1 https://www.abcam.cn/products/primary-antibodies/ddb1-antibody-ab9194.html
YTHDF2 https://www.ptgcn.com/products/YTHDF2-Antibody-24744-1-AP.htm
EIF4E https://www.ptgcn.com/products/CBP,EIF4E-Antibody-11149-1-AP.htm
LAMP1 https://www.scbt.com/p/lamp-1-antibody-h4a3
CDC27 https://www.ptgcn.com/products/CDC27-Antibody-10918-1-AP.htm
BRD1 https://www.abcam.cn/products/primary-antibodies/brd1-brl-antibody-epr12960-ab181060
PBRM1 https://abclonal.com.cn/catalog/A0334

H3K14ac https://www.cellsignal.cn/products/primary-antibodies/acetyl-histone-h3-lys14-d4b9-rabbit-monoclonal-antibody/7627
TBK1 https://abclonal.com.cn/catalog/A3458
AAK1 https://www.abcam.cn/products/primary-antibodies/aak1-antibody-epr51262-ab134971
BAG6 https://www.ptglab.com/products/BAG6-Antibody-26417-1-AP.htm
MAEA https://www.ptgcn.com/products/MAEA-Antibody-28363-1-AP.htm
CTNNBL1 https://www.ptglab.com/products/CTNNBL1-Antibody-13665-1-AP.htm
MBNL2 https://abclonal.com.cn/catalog/A12838
U2AF1 https://abclonal.com.cn/catalog/A1046
ATM https://www.cellsignal.cn/products/primary-antibodies/atm-d2e2-rabbit-mab/2873
RPA2 https://www.abcam.cn/products/primary-antibodies/rpa32-rpa2-antibody-epr2877y-ab76420
RAD51 https://www.abcam.cn/products/primary-antibodies/rad51-antibody-ab63801
XRCC1 https://www.cellsignal.cn/products/primary-antibodies/xrcc1-antibody/2735
S9.6 https://www.sigmaaldrich.cn/CN/en/product/mm/mabe1095
EZH2 https://www.cellsignal.cn/products/primary-antibodies/ezh2-d2c9-xp-rabbit-mab/5246
H3K27me3 https://www.cellsignal.cn/products/primary-antibodies/tri-methyl-histone-h3-lys27-c36b11-rabbit-mab/9733
RNase H2A https://www.ptgcn.com/products/RNASEH2A-Antibody-16132-1-AP.htm
GAPDH https://www.ptgcn.com/products/GAPDH-Antibody-60004-1-Ig.htm
BUB3 https://www.scbt.com/zh/p/bub3-antibody-e-7?requestFrom=search
CREST https://fitzgerald-fii.com/crest-antibody-70r-21494.html

Animals and other research organisms

Policy information about studies involving animals; ARRIVE guidelines recommended for reporting animal research, and Sex and Gender in Research

Laboratory animals	All mice used in this study were on a C57BL/6J genetic background. Female APEX^flox/flox and Gdf9-Cre transgenic mice were crossed to generate Gdf9-Cre; APEX^flox/+ (APEXoocyte) mice. Mice were maintained under specific pathogen-free (SPF) conditions at 20–22 °C with a 12 h light/dark cycle, 50–70% humidity, and ad libitum access to food and water. Experiments were performed using postnatal day 7, 14, or 21–23 female mice, as specified in the Methods.
Wild animals	This study did not involve wild animals.
Reporting on sex	All experiments were performed using female mice, as the study focused on ovarian biology and oocyte development. Therefore, sex-based analyses were not applicable.
Field-collected samples	This study did not involve samples collected from the field.
Ethics oversight	All animal procedures were approved by the Animal Research Committee of Zhejiang University and conducted in accordance with institutional guidelines and regulations.

Note that full information on the approval of the study protocol must also be provided in the manuscript.

Plants

Seed stocks	n/a
Novel plant genotypes	n/a
Authentication	n/a
