# Supplementary material for: In vivo labeling–based proteomic analysis of early follicle oocytes and cisplatin-induced alterations in mice
Source: Nat Commun. 2026 Aug 15;17:9815. doi: 10.1038/s41467-026-76665-3 (PMC13574812; doi:10.1038/s41467-026-76665-3)
Supplement: Supplementary file 2 [file 41467_2026_76665_MOESM13_ESM.pdf]

nature portfolio

Peer Review File

In Vivo Labeling–Based Proteomic Analysis of Early Follicle Oocytes and Cisplatin-Induced Alterations in Mice

Corresponding Author: Professor Mo Li

This file contains all reviewer reports in order by version, followed by all author rebuttals in order by version.

Version 0:

Reviewer comments:

Reviewer #1

(Remarks to the Author)
The submitted manuscript by Xie et al. explores the tissue-specific omics of Primordial Follicle Oocytes using the APEX2 technique. Unlike previous studies that have only profiled oocytes at more mature stages, likely due to the difficulty of isolating these cells from complex tissues, the authors overcame this challenge by employing Cre-dependent APEX2 expression and labeling exclusively in oocytes. Through the APEX-mediated proximity labeling method, they isolated biotin-labeled proteins and biotinylated RNA, validating stage-specific expression of particular proteins and transcripts. They further investigated the impact of cisplatin, a chemotherapeutic agent, on oocytes using the same proximity labeling omics approach. Their data highlighted the upregulated expression of EZH2 under stress conditions, revealing that EZH2-induced histone modification led to meiotic arrest. Treatment with GSK126, an EZH2 inhibitor, restored meiotic progression. Overall, this study presents an oocyte-specific molecular landscape using an advanced in vivo proximity labeling approach, with experimental data of sufficient quality to warrant publication in Nature Communications following revisions.

1. In this study, the authors performed in vivo APEX2 labeling by injecting BP for 2 hours and H2O2 for 5 minutes. However, the direct administration of these chemicals to live mice can be toxic. The authors should carefully validate whether these injections affect the physiological processes in the mice.

2. In Figure 2E, numerous biotinylated proteins were detected in the negative control (APEX2 flox/+), despite negligible SA-HRP western blot signals in the same samples. If mass spectrometry similarly detects biotinylated proteins in control samples and APEX2-oocyte samples, the authors should consider re-optimizing the mass analysis to eliminate false positives during SA-bead enrichment. Alternatively, they could use a super-resolution proximity labeling method to identify "true-positive" biotinylated peptides (PMID: 38724559). Otherwise, it is hard to believe that all the identified proteins in current dataset are really biotinylated by APEX2.

3. The authors should improve the clarity of their omics data presentation in the figures and figure legends. For instance, the meaning of the numbers in Figure 2I is unclear. Making figures and figure legends more reader-friendly would be beneficial.

4. It would also be helpful if the authors provided a schematic figure in the final figure that illustrates the oocyte-specific molecular events following cisplatin treatment, based on their results. The current figure in Fig. 7 is more akin to a Graphical Abstract.

6. in the Data Availability section, the authors described that the proteomics data has been deposited to the ProteomeXchange Consortium (https://proteomecentral.proteomexchange.org/cgi/GetDataset?ID=PXD048332). However, it appears that a username and password are required to access dataset PXD048332. The authors should revise the access option to allow public access to their raw data.

7. All the supplementary dataset files (Excel files) should include Uniprot ID and Entrez Gene ID (NCBI Gene ID). Additionally, it would be beneficial to provide an additional supplementary dataset file (Excel file) containing all peptide information (e.g. analysed peptide sequence, MS1 intensity, sequence matched protein name, Uniprot ID, NCBI Gene ID, etc) per each replicate sample before data processing. This data may help to understand real mass result and quality of the data.

Reviewer #2

(Remarks to the Author)
In their manuscript, Jiang et al. introduce a novel mouse model for Cre-driven APEX-mediated proteome labeling in vivo. The model enables identification of cell-type-specific changes in protein abundance during development and in response to treatments. Using this method, authors aim to identify therapeutic targets for fertility preservation in cancer patients. First, the authors demonstrated that this system specifically labels oocytes with APEX and EGFP driven by Gdf9-Cre recombinase. Next, they demonstrated that APEX-labelled proteins, as well as RNAs, were effectively pulled down and identified using mass spectrometry (MS) and RNA sequencing (RNAseq), respectively. Finally, they applied APEX to identify proteins differentially expressed in response to short-term and low-dose treatment with chemotherapy drug cisplatin. Using this approach, authors identified EZH2 as one of the proteins that changed abundance in oocytes after cisplatin treatment and thus could be a target for fertility preservation. To test this, authors used EZH2 inhibitor GSK126 and showed that it prevents cisplatin-induced maturation defects in fully grown oocytes. While this novel mouse model is poised to be a very valuable and powerful tool for the study of oocyte proteomes and responses to a variety of conditions, concerns related to experimental design and data interpretation need to be addressed. Specifically, based on presented data, it remains unclear that the proteomes and transcriptomes captured by APEX originate from primordial follicle stage oocytes. Addressing this and other concerns will strengthen the manuscript’s impact and relevance.
Specific comments
1. Expression of APEX system in oocytes: EGFP expression driven by Gdf9-Cre is evident in oocytes; however, to confirm that all oocytes were captured, authors should perform co-immunostaining with a known oocyte marker such as DDX4 in whole ovary and on sections. For example, in Figure 2C, primordial follicles don’t seem to be labeled in the PD21 ovary while growing follicles from the first wave of folliculogenesis are clearly labeled. Therefore, there may be some quiescent primordial follicles from the second wave of folliculogenesis that may not yet express Gdf9-Cre. It is also concerning that MS and RNAseq of APEX-labelled proteome and transcriptome lack major markers of quiescent oocytes but include many proteins for fully grown oocytes (FGO). See other comments below.
2. APEX-labeled proteome in primordial follicle oocytes: Authors use PD7 ovaries to identify proteins expressed in primordial follicle oocytes. Based on that, authors conclude that some proteins previously detected at later stages in FGO, including meiotic maturation, are already present in primordial follicle stage oocytes. However, it is important to note that the pulled-down proteins may originate from large-growing primary follicles present in PD7 ovaries (as seen in Figure 2C). Immunohistochemistry on full sections confirms that these proteins are most abundant in growing primary and secondary follicles. Additionally, some instances in Figure 3, chosen to illustrate protein expression in primordial follicles, actually depict primary follicles (e.g., Fig. 3F for GOPC staining or Fig. S2F for DNMT1). Fig.3B for NDUFA5 does not really look like a primordial or even a follicle).
a. Furthermore, MS of APEX-labelled proteins (Table S1) failed to detect many known markers abundantly expressed in primordial follicle oocytes, including TRP63, SOHLH1/2, and NOBOX. Authors should comment on the limitations of their approach and why these markers were not identified but DDX4 was. Is it possible that click chemistry is not efficient in primordial follicle oocytes? To confirm that APEX labels primordial oocyte proteins authors should show by western blot that these markers are present in pulled-down proteomes, e.g., in Fig.2D.
b. Similar lack of primordial oocyte markers in transcriptome data: In lines 289-291: authors state that “a large number of germ cell markers were included in the transcriptome”; however, Table S2 lacked major oocyte marker genes such as Nobox, Sohlh1/2, and Trp63. Authors should mark few classical germ cell and granulosa marker genes in the plot in Fig. S4E and validate their presence by QPCR in pulled down RNA.
3. In Fig. S4I and lines 295-299, the authors report that APEX-transcriptome sequencing failed to overlap with ~50% of proteins detected in oocytes. This unexpected finding—where proteins are present without corresponding RNA—warrants discussion. The limitations of APEX labeling should be acknowledged, and potential reasons for this discrepancy explained.
4. Missed opportunity for higher dose cisplatin labeling: Not performing APEX proteome labeling after a higher dose of cisplatin represents a missed opportunity. This higher dose induces DNA damage and significant primordial follicle depletion and may offer better targets for follicle protection. While the lower dose had no impact on oocyte morphology and DNA damage, the authors suggest that RNAseH2A protein abundance was increased due to RNA:DNA hybrids during DSB repair (lines 357-366). The authors also show an increase in the abundance of ATM kinase, which responds to DSBs. To address this, other markers (e.g., RAD51 or RPA) could be used to detect DSBs in oocytes after low-dose cisplatin. Additionally, identifying other DDR proteins in volcano plot in Figure 5K is recommended.
5. EZH2 as a target for oocyte protection and fertility preservation: The authors identified EZH2 as a protein with changed abundance after cisplatin treatment in PD7 ovaries and a potential target for follicle protection. The authors then conclude in lines 375-376 that epigenetic abnormalities such as H3K27me3 related to changes in EZH2 expression may affect subsequent oocyte maturation and female fertility. This led authors to test EZH2 inhibition during cisplatin treatment and its effect on cisplatin-treated oocytes. However, it is unclear why authors used treated FGO rather than PD7 ovaries with primordial follicles to assess maturation defects. The manuscript lacks evidence that primordial follicles exposed to cisplatin at PD7 had maturation defects when they reached the FGO state. Treating FGO with EZH2 inhibitor and cisplatin seems unrelated to the rest of the manuscript. Furthermore, details on FGO treatment with cisplatin (dose, duration, etc.) are missing in methods. Authors should clarify the rationale for using FGO or consider treating PD7 ovaries with cisplatin (both low and high doses) and GSK126 to demonstrate a protective effect.
6. H_2O_2 injection and oxidative stress: The system relies on H_2O_2 injection, which may induce oxidative stress in oocytes. The authors should address whether this dose of H_2O_2 causes damage in the ovary and potentially confounds proteome studies. Additional experiments to assess oxidative damage would be valuable.
7. Novelty of in-situ imaging and follicle quantification: While the authors emphasize the novelty of their system for in-situ imaging of oocytes and follicle quantification, it’s essential to acknowledge existing studies. Previous research has demonstrated 3D ovarian structure and performed follicle quantification using immunostaining and clearing methods (e.g., PMID: 31965156, PMID: 34542534, PMID: 38798456, PMID: 37464244). Although this method is antibody-free and quicker,

its applicability to complex genetic models (e.g., conditional knockouts) should be considered.
8. Follicle stage identification and immunostaining examples: Authors incorrectly identified follicle stages and used incorrect stages as examples in immunostaining figures. This should be corrected, and authors are encouraged to consult with follicle development specialists if they lack expertise in this area.

Reviewer #3

(Remarks to the Author)
Manuscript background information:
In this manuscript by Jiang, et al, the authors develop a proteomic profile of oocytes in primordial follicles using a novel ascorbate peroxidase (APEX)-based protein labeling mouse model. Using this model, the authors are able to visualize the ovary in 3D, capture and identify proteins specific to oocytes, and capture and sequence RNA from oocytes. This model and findings can be applied in future research to improve tools for determining reproductive capability in females, particularly those undergoing chemotherapy treatments. Cisplatin, a common cancer treatment, is known to negatively affect female fertility. More specifically, cisplatin induces apoptosis in oocytes and mitochondrial damage to immature oocytes. Therefore, it is worthwhile to investigate proteomic shifts in oocytes from primordial follicles after cisplatin treatment. Comparing APEX-labeled ovaries from mice treated with cisplatin with control mice, they found there were rapid changes in proteins involved in DNA damage repair and histone modification. With these findings, the authors were able to protect oocytes from the effects of cisplatin using a EZH2 inhibitor, GSK126. This study validates the first in vivo APEX-based proteins labeling model and characterizes the proteome of oocytes from primordial follicles both in normal development and when perturbed by cisplatin exposure.

Major comments/limitations:

1. The authors claim that by performing their captures in PD7 ovaries, they are profiling only primordial follicles, as they are the dominant population in postnatal day 7 ovaries. However, they do not seem to account for the population of growing oocytes present in the medullary region of the ovary at this stage. While it is true that this population is much smaller (as the authors show in figure 1), it is possible that their proteome could be over-represented in the dataset, as these are much bigger and active oocytes. This is also suggested by the fact that many of the proteins the authors identify are clearly found in the oocyte throughout follicle growth, as illustrated by the IHC experiments in figures 3 and 4. One way the authors could address this would be to perform APEX profiling using a growing-oocyte specific cre line, such as Zp3-Cre (Lewandowski et al, 1997) and cross-reference this profile to the present one to identify primordial-specific proteins. I recognize this is a big experiment, so if the authors are not in a position to perform this, I suggest softening the conclusions and language throughout the text to clarify that this is not a primordial-specific profile.

2. Authors should include more details about the methods across the manuscript. As they often use multiple ages of mice in this study, including that information is crucial. Additionally, in images of oocytes/follicles, it is not clear if these are isolated oocytes or oocytes within the whole ovary. If these are isolated oocytes, their method of extraction should be clear.

3. More detail is needed in the Methods section, especially pertaining to the methods of IHC and IF image analysis. How is labeling intensity being measured? Adding an image of tissue treated only with secondary antibodies would be useful for convincing the reader that the signal they see is specific staining.

4. The authors state that this system is advantageous because they are able to characterize the in situ proteome. I'm having trouble understanding how this model is in situ. I think clarifying this in the introduction would be beneficial.

5. There are already many established protocols to visualize the entire perinatal ovary. I recommend adding citations to this previous body of work to credit them for their contributions. For example, we recommend citing Soygur et al. Sci. Adv. (2021).DOI:10.1126/sciadv.abc6747, Boateng et al. J. Vis. Exp. (2021).doi:10.3791/62972, Faire et al. Developmental Biology.(2015). https://doi.org/10.1016/j.ydbio.2015.04.006., McKey et al. eLife.(2022). https://doi.org/10.7554/eLife.81088, Folts et al. bioRxiv.(2024).doi: https://doi.org/10.1101/2024.05.13.593993, McKey et al. PNAS. (2019).https://doi.org/10.1073/pnas.1814930116, Feng et al. Sci Rep (2017). https://doi.org/10.1038/srep44810.

6. The APEX-labeling system to visualize the whole ovary is useful for their proteomic approach but seems impractical as a platform to image the whole perinatal ovary. Consider moving Figure 1 to supplemental data as validation that the APEX-labeling system does target oocytes efficiently. If the authors compared their findings from Figure 1 to previously described 3D ovary imaging techniques, that would make it more significant.

7. The authors do not describe the mouse lines they used for their experiments (Line 487 - 494) How was the APEX line generated, and which Gdf9-cre line was used? How was it obtained?This is absolutely critical information for reproducibility. The authors should also confirm that Gdf9-Cre targets all oocytes, including primordial follicles, since the GDF9 protein is typically only expressed in growing follicles. If they do confirm expression of the transgene in primordial oocytes, they should clearly address this difference in expression pattern.

8. In the explanation of Figure 1, provide more detail on image analysis and how you account for oocyte segmentation and oocytes that were miss-segmented. We also recommend adding an overlay in Figures 1E and 1H with the corresponding IF image to illustrate the segmentation efficiency.

9. In Figure 1, further validation is needed to show that all oocytes are being labeled through the APEX system. I recommend co-staining with DDX4, a germ cell marker, and GFP. If the APEX system is capturing all oocytes, then DDX4 and GFP will co-localize in all oocytes. Additionally, more information is needed on how image analysis was performed, specifically how oocyte diameter and follicle volume was calculated.

10. Fig 2 - 2C - With the current image, I am not convinced that all the primordial follicles are being labeled with streptavidin. It looks like growing follicles but not primordial follicles are labeled with streptavidin. I recommend performing a co-stain with DDX4, a germ cell marker, to show that all the oocytes, especially the primordial oocytes, are captured with APEX.

11. Line 150 - It is unclear how hydrogen peroxide is administered. Is the mouse injected for 5 minutes or is the mouse injected and collected after 5 minutes? Injecting hydrogen peroxide does not seem healthy for the animal. Did you confirm that hydrogen peroxide injections do not negatively impact the health of the mouse, more specifically did you confirm that this inject does not affect ovarian health? Consider looking at apoptosis in the ovary after treatment and/or confirming these mice are still fertile after this treatment.

12. I recommend the authors add more citations on current techniques and knowledge in the ovarian biology field. There are claims of novel findings that have been previously reported in the literature, such as the ability to optically clear, segment and count follicles in the 3D ovary (lines 140-145), or the fact that the ovarian cortex contains more primordial follicles (line 463 to 465), which was clearly demonstrated in Cordeiro et al, 2015: doi: 10.1095/biolreprod.115.131227.

13. It is important to consider how the proteome of oocytes might be different between early postnatal ovaries and adult ovaries, especially considering how the hormonal environment differs between early postnatal and adult mice. This could be discussed as a limitation of the study.

14. I recommend changing the title and discussion to reflect that APEX is capturing primordial, primary, and secondary follicles. Based on validation in Figure 1 and Figure 2, it seems that APEX is not capturing many primordial follicles.

Minor comments/limitations:
Line 30 - The word “realized” in this sentence is confusing. Consider rewording.
Line 73 - A more in depth explanation of APEX systems is needed.
Line 83 - Reconsider using the word “panorama” to describe a 3D image.
Line 124 - Compare the number of oocytes counted to previously published counts. Are all the oocytes being captured?
Line 138 - How is the border of the cortex being defined?
Line 162 - Should “7D ovaries” be PN7 ovaries?
Figure 3A - What plot is this? What are the axes? I think this plot could be explained better in the text.
Figure 4F - Text behind the pink bubbles can’t be read. Consider reformatting this plot.
Figure 5M - It would be beneficial to add arrows to show the reader where the spots are.
Line 454 - Include citations.
Line 457 - The sentence beginning with “The conventional cross sections..” could be reworded for clarity.
Line 463 - This has been confirmed before, add citation (Cordeiro et al, 2015)
Line 478 - Good discussion of limitations.
Line 996 - In the legend for Figure 1C, It would be helpful to include how these oocytes were isolated and what age the mice were.
Line 1015 - In the legend for Figure 2B, include if these are isolated follicles or if these images are from whole ovaries.
Line 1043 - In the legend for Figure 3D, include how these oocytes were isolated and what age the mice were.
Line 1128 - Include a sentence or two to summarize the graphical abstract.

Reviewer #4

(Remarks to the Author)
I co-reviewed this manuscript with one of the reviewers who provided the listed reports. This is part of the Nature Communications initiative to facilitate training in peer review and to provide appropriate recognition for Early Career Researchers who co-review manuscripts.

Version 1:

Reviewer comments:

Reviewer #1

(Remarks to the Author)
The authors present a highly significant spatial proteomics study that maps the first in situ proteome of early-stage oocytes and uncovers the epigenetic mechanism underlying cisplatin-induced ovarian damage. While the authors have made significant efforts to optimize the proteomic workflow and improve the data quality during revision, there are still inherent technical limitations to the current methodology that must be explicitly acknowledged in the Discussion/Limitations section of the manuscript. I request the authors to add brief discussions addressing the following two points:

1. Although the authors employed stringent washing steps (e.g., High-salt, Urea) to reduce background noise, traditional streptavidin pull-down cannot entirely eliminate the risk of false positives. Specifically, non-biotinylated proteins that strongly interact with actual biotinylated proteins can be co-precipitated, especially during the post-lysis process. Because Super-Resolution Proximity Labeling (SR-PL) or direct identification of biotinylation sites at the peptide level was not performed, the dataset inherently carries the risk of including these indirect interactors. This limitation must be explicitly addressed in a Discussion part.

2. The labeling intensity and the resulting proteomic profile are highly dependent on the administered dose and duration of BP and H_2O_2. Since the labeling is performed in vivo, pharmacokinetic variations can significantly affect the actual concentration of probes reaching the target ovary. As a dose-dependent optimization of the probes to maximize ovarian bioavailability was not performed, the potential variation in labeling outcomes depending on dose and time should be clearly stated in a Discussion or Method part.

Minor:

There are several typos in figure numbers and grammatical errors that need to be corrected before publication.

Reviewer #2

(Remarks to the Author)
The authors have undertaken a substantial revision of this manuscript, including additional samples, expanded datasets, new co-immunostaining experiments (DDX4/streptavidin), comprehensive H_2O_2 toxicity assessment, additional DDR protein validation (RAD51, RPA2, XRCC1), in vivo PD7 cisplatin ± GSK126 treatment experiments (although limited to short-term outcomes), and an appropriately modified title and softened language throughout. These represent a commendable amount of new work. However, the authors should ensure that all new results presented in the rebuttal are fully incorporated into the manuscript text, not only as figures. Several remaining concerns require attention before publication.
Specific comments
1. Primordial follicle representation in the proteome/transcriptome: The additional data confirming that the proteome and transcriptome include primordial follicle oocytes are appreciated and substantially address the original concern. However, the manuscript (p. 10, lines 222–223) still describes the population of growing follicles in the PD7 ovary as “a few primary and secondary follicles,” whereas the authors’ own quantification data show roughly 200 growing follicles—a population that could contribute substantially to the labeled proteome. This understatement should be corrected. Additionally, the authors performed experiments with a shortened BP incubation time, claiming this should limit labeling in growing oocytes; however, this was not validated experimentally. Cross-sections of the ovary labeled with DDX4/streptavidin under both the original and shortened labeling protocols should be presented to support the claim that labeling in growing oocytes is decreased without compromising labeling efficiency in primordial follicles.
2. H_2O_2 toxicity and oxidative stress: The authors have provided comprehensive data addressing this concern, including histological analysis showing normal ovarian morphology and comparable follicle numbers, no increase in apoptosis, and normal meiotic maturation. Although some minor changes were detected, the presented data effectively eliminate the concern that H_2O_2 confounds the proteomic data. However, the methodology for the H_2O_2 differential proteomic analysis is not described in the Methods section. This should be corrected.
3. Connecting cisplatin exposure in early-stage oocytes to later meiotic defects: The authors have added considerable new experimental data showing in vivo treatment of neonatal ovaries with cisplatin ± GSK126. However, these data still do not support their statement that cisplatin treatment in early-stage oocytes leads to later meiotic defects (p. 20, lines 470–474). First, treatment with cisplatin ± GSK126 at PD14 followed by FGO collection 7 days later is too short to capture damage originating in primordial or primary oocytes, as it takes considerably longer than 7 days for these to develop into FGOs. This experiment effectively shows the consequences of cisplatin-induced toxicity on secondary follicle oocytes already present in the PD21 ovary. The rebuttal explains that PD7-treated mice did not survive to adulthood, precluding direct long-term follow-up of primordial follicles—this is an important practical limitation that should be explicitly stated in the manuscript. Whether primordial follicles that survive low-dose cisplatin exposure at PD7 eventually develop meiotic competence defects remains undemonstrated. The authors should clearly acknowledge in the Discussion that the maturation defect evidence derives from treating growing oocytes (PD14 ovary treatment and in vitro FGO), not from tracing the fate of cisplatin-exposed primordial follicles. The current text at times implies this connection without stating the limitation. For instance, the Discussion states that “EZH2 was overexpressed in oocytes and led to enhanced H3K27me3 levels after cisplatin treatment, accompanied by meiotic maturation defects” (p. 21, lines 493–500) without specifying which experimental paradigm supports each part of this claim. Moreover, this new set of experiments raises the question of whether primordial follicles treated with low-dose cisplatin survive beyond 7 days when co-treated with GSK126. Figure 6c shows the percentage of abnormal primordial follicles in GSK126-treated ovaries, but the authors should also provide absolute follicle numbers to demonstrate whether GSK126 treatment prevents follicle loss, not merely delays morphological abnormalities.
4. Role of EZH2 in DNA repair versus epigenetic regulation: Based on changes in H3K27me3 levels, the authors suggest that cisplatin induces early EZH2-mediated epigenetic changes that lead to later meiotic defects. However, the authors neglect that H3K27me3 is also recruited to DNA damage sites as part of the DNA repair process (e.g., PMID: 31395347). The accumulation of H3K27me3 following cisplatin treatment may therefore reflect a direct role of EZH2/PRC2 in the DNA damage response rather than—or in addition to—epigenetic reprogramming. The dual role of EZH2 in epigenetic regulation

versus DNA repair should be discussed, as this distinction has important implications for interpreting whether GSK126 is mitigating epigenetic damage or interfering with DNA repair at damage sites.
5. Internal contradiction regarding DNA damage after low-dose cisplatin: The authors' conclusions about DNA damage following low-dose cisplatin (2 mg/kg, 4 h) contain an internal contradiction that undermines one of the manuscript's central claims. On one hand, the text states that γH2AX staining showed "the higher, but not the lower, dose induced marked DNA double-strand breaks" (p. 16, lines 381–386), framing the low dose as causing no overt DNA damage by conventional assessment. On the other hand, the authors demonstrate that the same low-dose cisplatin induces ATM nuclear foci, RAD51 and RPA2 nuclear accumulation, and XRCC1 upregulation in primordial follicle oocytes—all of which are direct, canonical markers of active DSB repair. These proteins cannot accumulate in the absence of DSBs. Therefore, the APEX-detected proteomic changes do not reveal a hidden molecular response invisible to conventional methods, as the authors claim, but rather a standard DNA damage response that should have been assessed alongside γH2AX with appropriate sensitivity. The authors should acknowledge that low-dose cisplatin does induce DSBs and reframe the narrative around what APEX proteomics reveals beyond what immunostaining already shows.
6. 3D imaging novelty: The revised Discussion now appropriately cites prior 3D ovarian imaging studies and repositions the 3D visualization as a complementary feature rather than the primary innovation. The advantages (antibody-free, rapid, compatible with Cre-driver lines) and limitations (requires genetically engineered mice) are clearly stated. However, the authors still overstate the novelty of their 3D imaging workflow. The same results could be achieved with other Cre-driver lines crossed to any fluorescent reporter (e.g., a TdTomato-expressing Ai9 line) combined with DNA staining to visualize granulosa cell nuclei. The text should more accurately reflect this aspect of APEX mouse model.
7. Inconsistent fold-change thresholds and statistical reporting: The revised manuscript contains significant inconsistencies in fold-change thresholds and notation across analyses that must be resolved. (i) For the proteome: the volcano plot axis is labeled $\log_2$(fold change), and the text states "FC > 1.5, P < 0.05," which appears to mean linear fold change > 1.5. The rebuttal to Reviewer 1 confirms this. However, the original submission used $\log_2$FC > 1 (equivalent to linear FC > 2)—an entirely different threshold. The switch is not discussed. (ii) For the transcriptome: the Methods section indicates FC > 2 and adjusted P-value < 0.05, yet the Results text states "FC > 1.5." With FC > 2, there are 1,137 DEGs; with FC > 1.5, approximately 2,002—neither matches the 2,878 reported. (iii) For cisplatin-responsive transcriptomics: $\log_2$FC > 1 is used (equivalent to FC > 2), inconsistent with FC > 1.5 used for the baseline transcriptome. (iv) For cisplatin proteomics: upregulated proteins are reported with "FC > 1.5" and downregulated with "FC < 0.67," which is the reciprocal—consistent with linear FC—but the original text used $\log_2$FC. The authors must standardize all thresholds, use consistent notation (always specifying whether FC is linear or $\log_2$-transformed), report adjusted P-values where applicable, and ensure that all reported numbers match the supplementary data tables. This is essential for reproducibility.
8. Figure cross-references: Several figure cross-references in the revised text appear incorrect. These should be systematically checked and corrected throughout the manuscript.

Reviewer #3

(Remarks to the Author)
The authors have provided a very thorough response to reviewers and have clearly addressed all concerns raised by the reviewers. The clarity and rigor of the manuscript are much improved, and I appreciate the authors' efforts to discuss limitations of their study, to add acknowledgments of previous work in this area, and to soften their conclusions where needed. I have no further concerns.

Version 2:

Reviewer comments:

Reviewer #2

(Remarks to the Author)
Authors addressed my key points. I have no further comments.

Open Access This Peer Review File is licensed under a Creative Commons Attribution 4.0 International License, which permits use, sharing, adaptation, distribution and reproduction in any medium or format, as long as you give appropriate credit to the original author(s) and the source, provide a link to the Creative Commons license, and indicate if changes were made.
In cases where reviewers are anonymous, credit should be given to 'Anonymous Referee' and the source.
The images or other third party material in this Peer Review File are included in the article's Creative Commons license, unless indicated otherwise in a credit line to the material. If material is not included in the article's Creative Commons license and your intended use is not permitted by statutory regulation or exceeds the permitted use, you will need to obtain permission directly from the copyright holder.
To view a copy of this license, visit https://creativecommons.org/licenses/by/4.0/

Response Letter

Dear Reviewers,

We are very grateful for the constructive suggestions from the Reviewers. These comments are very valuable and helpful for improving our paper. As requested, we have addressed all the concerns point by point. Accordingly, we have added and updated experimental data and modified the text in the revised manuscript. We hope this revised version will meet the quality for publication. For details, please see the responses below.

REVIEWER COMMENTS

Reviewer #1:

The submitted manuscript by Xie et al. explores the tissue-specific omics of Primordial Follicle Oocytes using the APEX2 technique. Unlike previous studies that have only profiled oocytes at more mature stages, likely due to the difficulty of isolating these cells from complex tissues, the authors overcame this challenge by employing Cre-dependent APEX2 expression and labeling exclusively in oocytes. Through the APEX-mediated proximity labeling method, they isolated biotin-labeled proteins and biotinylated RNA, validating stage-specific expression of particular proteins and transcripts. They further investigated the impact of cisplatin, a chemotherapeutic agent, on oocytes using the same proximity labeling omics approach. Their data highlighted the upregulated expression of EZH2 under stress conditions, revealing that EZH2-induced histone modification led to meiotic arrest. Treatment with GSK126, an EZH2 inhibitor, restored meiotic progression. Overall, this study presents an oocyte-specific molecular landscape using an advanced in vivo proximity labeling approach, with experimental data of sufficient quality to warrant publication in Nature Communications following revisions.

1. In this study, the authors performed in vivo APEX2 labeling by injecting BP for 2 hours and H2O2 for 5 minutes. However, the direct administration of these

chemicals to live mice can be toxic. The authors should carefully validate whether these injections affect the physiological processes in the mice.

Response: We sincerely thank the reviewer for this important concern. To address this, we performed additional validation experiments to carefully evaluate whether our labeling procedure interferes with normal physiological processes in the mice.

First, histological analysis of whole ovaries at PD7 and PD21 showed normal gross morphology **(Supplementary Figs. 2a-b; Also see below)**. At PD7, stage-based follicle counts and total follicle numbers were comparable between the BP+H_2O_2- and PBS-treated groups (follicles at each stage/ovary, primordial: 2733.75 ± 379.93 vs. 2890.33 ± 224.52; primary: 134.02 ± 20.11 vs. 155.67 ± 4.51; secondary: 49.51 ± 13.11 vs. 68.42 ± 26.66; all n.s., $P > 0.05$). At PD21, a similar result was observed, with no significant differences between treated and control groups (follicles at each stage/ovary, primordial: 1462.33 ± 58.14 vs. 1494.04 ± 30.51; primary: 99.36 ± 18.03 vs. 97.11 ± 10.15; secondary: 127.24 ± 7.00 vs. 144.33 ± 21.01; antral: 49.33 ± 2.89 vs. 44.14 ± 7.21; all n.s., $P > 0.05$) **(Supplementary Figs. 2c-d; Also see below)**. Second, cleaved caspase-3 (CC3) staining of PD21 ovaries revealed similar numbers of apoptotic follicles between treated and control groups ($CC3^+$ follicles ratios/ovary, secondary: 2.50 ± 1.04% vs. 3.26 ± 0.96%; antral: 4.16 ± 1.21% vs. 5.58 ± 0.84%; all n.s., $P > 0.05$), with no $CC3^+$ cells detected at the primordial or primary stages **(Supplementary Figs. 2e-f; Also see below)**. To further verify the impact on primordial follicles, TUNEL assays were performed at PD7 and revealed no significant elevation in apoptotic follicles in the BP+H_2O_2 group relative to PBS ($TUNEL^+$ primordial follicles/ovary: 0.72 ± 0.63% vs. 0; n.s., $P > 0.05$) **(Supplementary Fig. 2g; Also see below)**. Third, fully grown oocytes (FGOs) collected at PD21 after *in vivo* reaction exhibited normal *in vitro* maturation, with GVBD and PBE rates comparable to controls, and normal spindle morphology (GVBD: 98.74 ± 2.80% vs. 99.51 ± 1.10%; PBE: 72.40 ± 8.74% vs. 74.28 ± 10.32%; all n.s., $P > 0.05$) **(Supplementary Figs. 2h-i; Also see below)**.

Taken together, these results indicate that intraperitoneal injection of BP and H_2O_2 under our conditions did not measurably affect follicle/oocyte morphology or

oocyte developmental competence. We have added these data to the **revised Supplementary Figs. 2a-i (also see below)** and updated **the corresponding text (lines 182–192)** in the Results section.

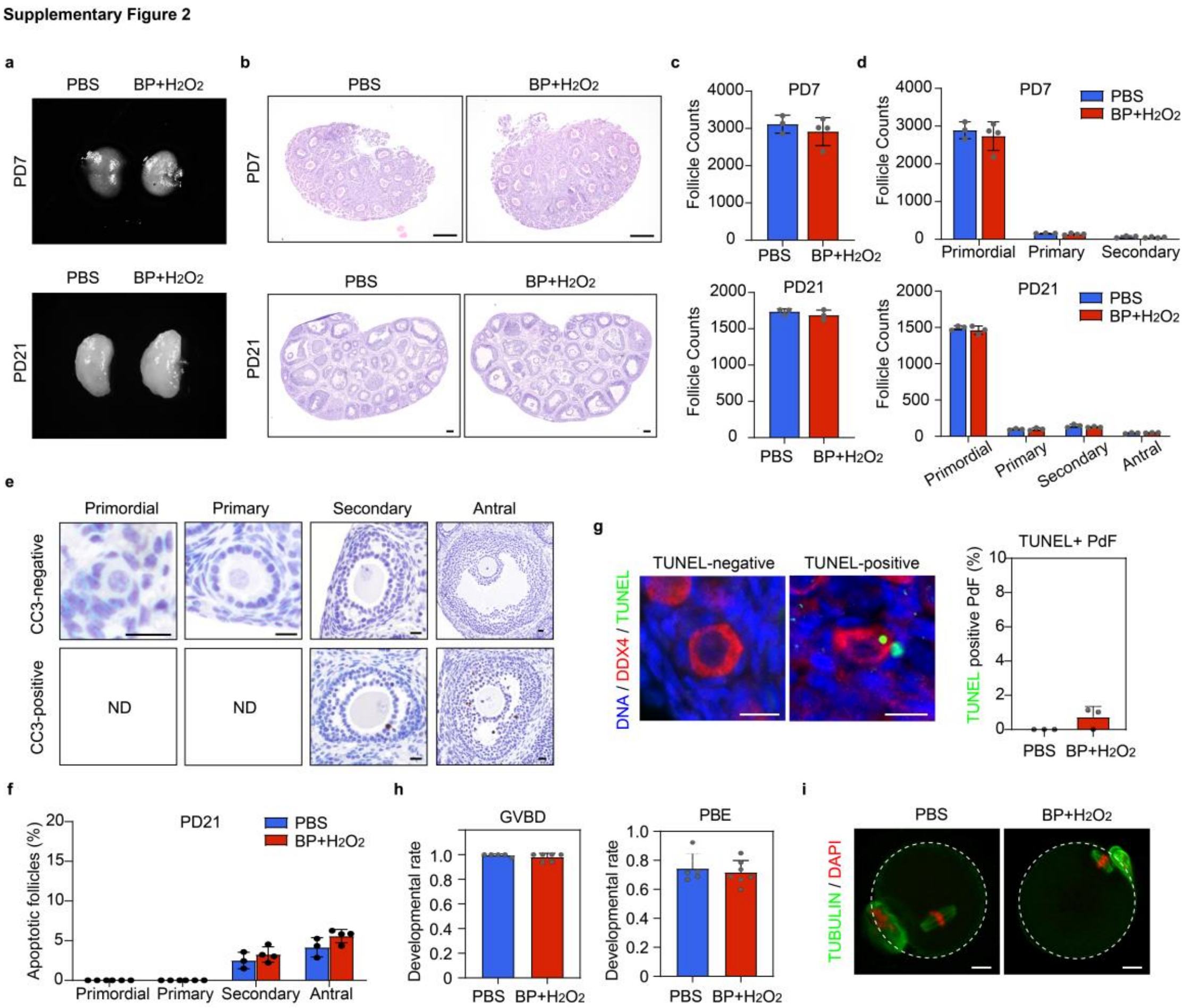

Supplementary Figure 2. The effects of APEX labeling reaction on ovarian physiological processes. a Gross morphology of ovaries collected at PD7 and PD21 from PBS- or BP+H_2O_2-treated mice. **b** H&E-stained ovarian sections showing comparable follicular architecture between groups at PD7 and PD21. Scale bars, 100 µm. **c–d** Quantification of total follicle numbers (**c**) and stage-specific follicle counts (**d**) at PD7 (top) and PD21 (bottom). Mean ± SEM. PD7 (n = 3, 4) and PD21 (n = 3, 3) for PBS and BP + H_2O_2, respectively. **e** Representative images of cleaved caspase-3 (CC3) staining in follicles at different stages at PD21. “ND”, not detected. Scale bars, 20 µm. **f** Quantification of apoptotic follicles detected by CC3 staining at PD21. Mean ± SEM. PD21 (n = 3, 4) for PBS and BP + H_2O_2, respectively. **g** TUNEL staining of

primordial follicles at PD7 showing representative TUNEL-negative and TUNEL-positive examples (left) and statistical comparison of $TUNEL^+$ primordial follicles per ovary (right). Scale bars, 20 μm. Mean ± SEM. n = 3 for each group. **h** *In vitro* maturation of FGOs collected at PD21 following *in vivo* labeling. Mean ± SEM. GVBD (n = 5, 6) and PBE (n = 5, 7) for PBS and BP + H_2O_2, respectively. **i** Representative spindle morphology of *in vitro* matured oocytes, visualized by α-tubulin (green) and DNA staining (red), demonstrating intact meiotic spindle formation in both groups. Scale bars, 20 μm.

2. In Figure 2E, numerous biotinylated proteins were detected in the negative control (APEX2 flox/+), despite negligible SA-HRP western blot signals in the same samples. If mass spectrometry similarly detects biotinylated proteins in control samples and APEX2-oocyte samples, the authors should consider re-optimizing the mass analysis to eliminate false positives during SA-bead enrichment. Alternatively, they could use a super-resolution proximity labeling method to identify "true-positive" biotinylated peptides (PMID: 38724559). Otherwise, it is hard to believe that all the identified proteins in current dataset are really biotinylated by APEX2.

Response: We sincerely thank the reviewer for raising this important point. To address this concern, we re-performed the APEX2 labeling experiments, including sample collection, streptavidin bead enrichment, and mass spectrometry analysis, with the following improvements.

First, we expanded the biological replicates, using 12 ovaries per repeat (previously 5). Second, we optimized the enrichment procedure by incorporating additional stringent wash steps with high-salt and urea buffers (1 M KCl, 0.1 M Na_2CO_3, 2 M urea; each twice) to minimize nonspecific binding. Third, we analyzed the samples on an updated mass spectrometer, which improved peptide coverage and quantitative accuracy. Quality control analyses confirmed the robustness of the revised dataset **(Supplementary Figs. 3a-b; Also see below)**. Importantly, the total abundance of

APEX-driven biotinylated proteins in the experimental group was substantially higher than the background levels observed in the control group **(Supplementary Fig. 3C; Also see below)**. We further applied a quantitative enrichment cutoff (fold change > 1.5, $P < 0.05$) by directly comparing APEX^{oocyte} versus *APEX$^{flox/+}$* proteomes. After filtering, 2772 proteins were designated as high-confidence oocyte proteome associated with primordial and early-stage follicles. Functional enrichment analyses demonstrated that these proteins are strongly aligned with key biological features of early oocytes, including chromatin regulation, RNA metabolism, kinase-mediated regulation, and protein stability control **(Figs. 2e-i; Also see below)**. In addition to global pathway enrichment, multiple well-recognized primordial and early oocyte–associated proteins were identified, and representative proteins from major functional categories were further validated by streptavidin pull-down followed by Western blotting and immunohistochemical analyses **(Fig. 3, Supplementary Figs. 3d-e, Supplementary Fig. 4; Also see below)**. Together, these analyses provide strong support for the reliability and biological relevance of the revised proteomic dataset.

Regarding the super-resolution proximity labeling method described in PMID: 38724559, we fully agree that this elegant strategy enables direct identification of biotinylation sites and significantly reduces false positives. However, this approach entails a more complex workflow, involving peptide-level enrichment and specialized MS analysis, together with higher sample input requirements, and is not yet routinely established in our laboratory. Therefore, we adopted the conventional approach with careful optimizations to ensure reliable results. We fully recognize the value of the method described in PMID: 38724559, and we anticipate exploring its application in future work.

In summary, by expanding sample size, introducing stringent washes, applying quantitative cutoffs, and performing orthogonal validation, we established a high-confidence proteomic dataset representative of oocytes in early-stage follicles, particularly at the primordial stage. We have added these data to the **revised Figure (Figs. 2e-i, Fig. 3, Supplementary Fig. 3, Supplementary Fig. 4; Also see below)** and updated **the corresponding text (lines 207–302)** in the Results section.

Figure 2

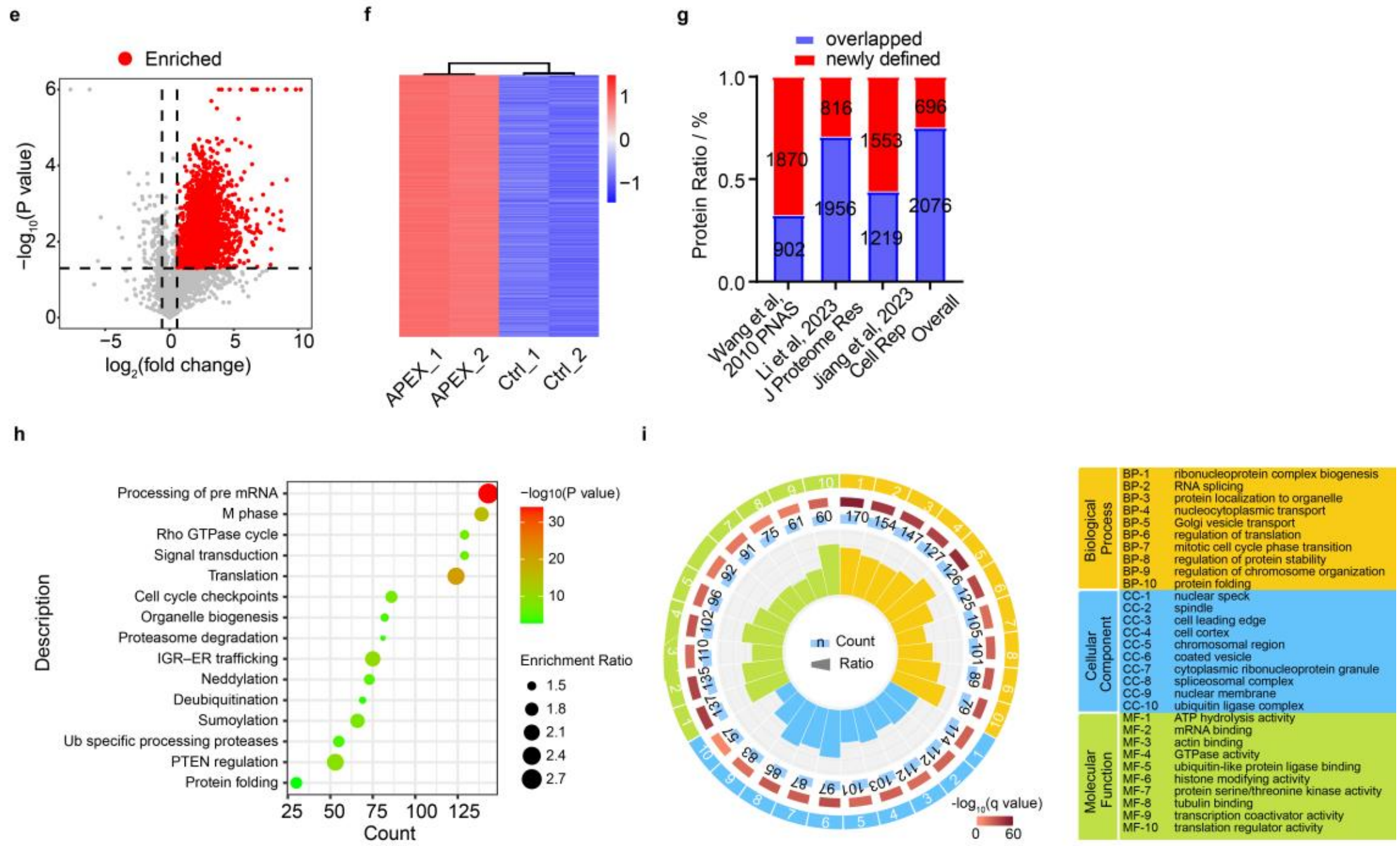

Figure 2. *In situ* APEX-based proteomic profiling of oocytes from early-stage ovarian follicles. e Volcano plot of proteins detected by LC–MS/MS comparing APEX^{oocyte} and *APEX*$^{flox/+}$ control samples. Significantly enriched proteins are highlighted (red dots, fold change > 1.5, P < 0.05). **f** Heatmap of enriched proteins across biological replicates of APEX^{oocyte} and *APEX*$^{flox/+}$ (Ctrl) samples. **g** Overlap analysis of the identified proteome with previously published GV-stage oocyte proteomes. Stacked bars indicate overlapped and newly defined proteins. **h** Bubble plot of enriched Reactome pathways. The x-axis shows the gene number (Count), the y-axis lists pathway names, bubble size reflects enrichment ratio, and color indicates significance (–$\log_{10}$ [P-value]). **i** Circular plot of the top 10 enriched gene ontology (GO) terms in biological process (yellow), cellular component (blue), and molecular function (green). From inside to outside: enrichment factor (Ratio), gene number (Count), statistical significance (–$\log_{10}$ [q-value]), and GO term IDs corresponding to the descriptions on the right.

Supplementary Figure 3

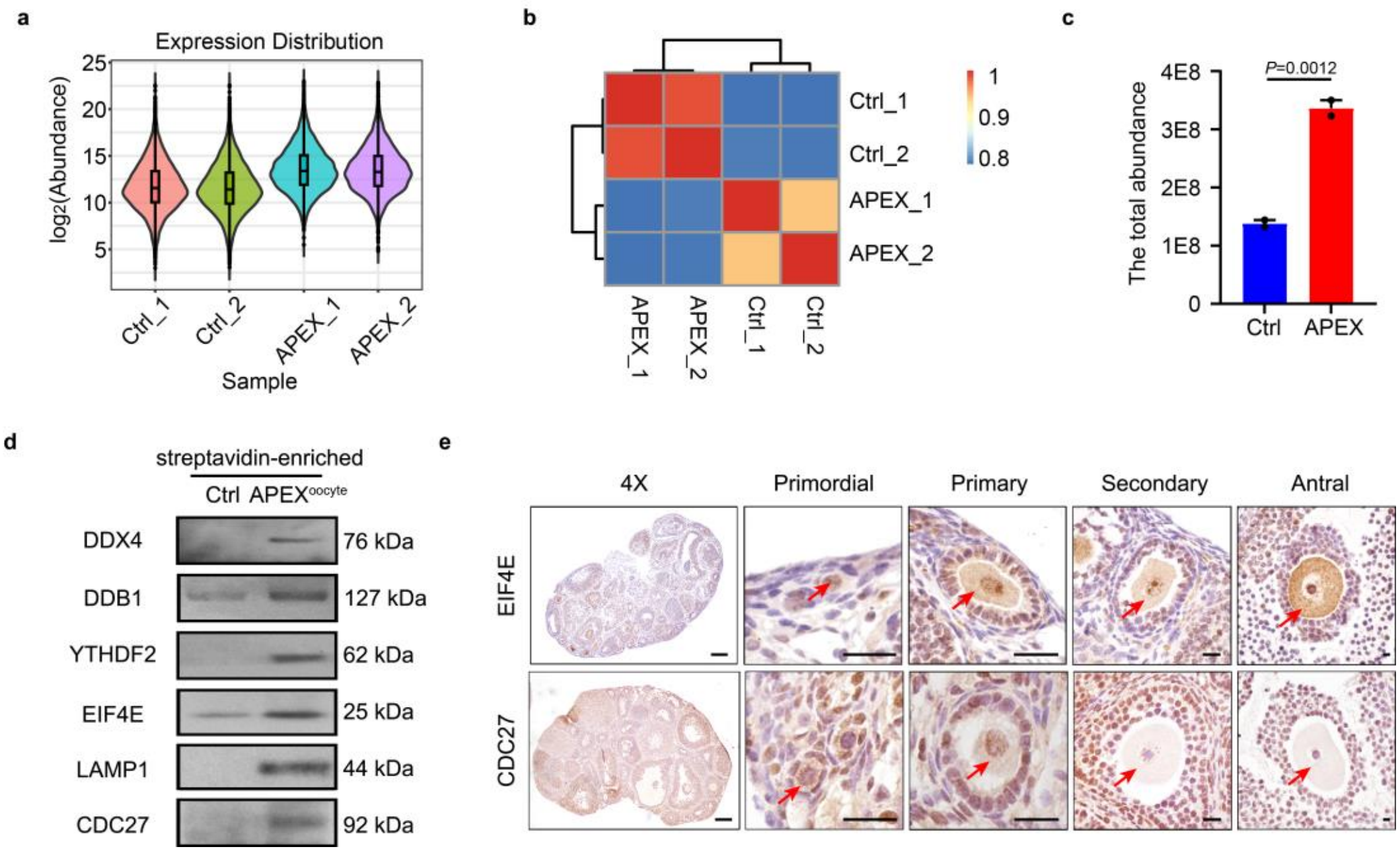

Supplementary Figure 3. Quality assessment and validation of the streptavidin-enriched oocyte proteome. a Distribution of protein abundance across samples shown as $\log_2$-transformed intensity values. Center line, median; box limits, upper and lower quartiles; whiskers, 1.5× interquartile range; points, outliers. **b** Sample correlation heatmap based on protein abundance profiles. **c** Total protein abundance detected in control and APEX samples. Mean ± SEM. n = 2 for each group. *P* values are calculated by unpaired two-tailed Student's t tests. **d** Western blot analysis of representative proteins in streptavidin-enriched fractions from control (Ctrl) and $APEX^{oocyte}$ ovaries. **e** Immunohistochemical staining of eukaryotic translation initiation factor 4E (EIF4E) and cell division cycle 27 (CDC27) in PD21 ovarian sections. Images include low-magnification overview and higher-magnification views of primordial, primary, secondary, and antral follicles. Arrows indicate oocytes. Scale bars, 100 μm in 4× microscopy and 20 μm in enlarged views.

Figure 3

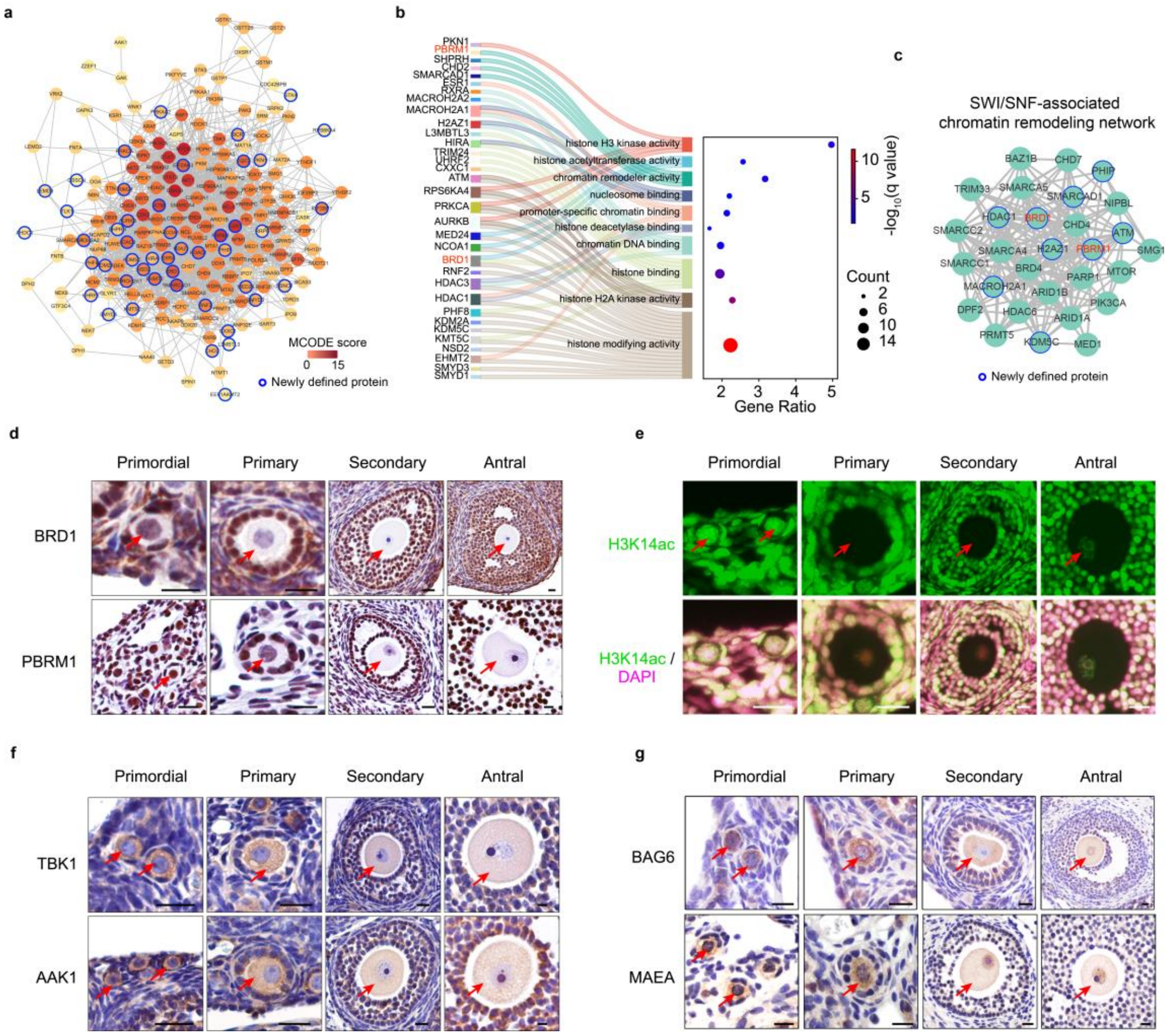

Figure 3. Expression dynamics of representative regulatory proteins in oocytes. a Protein–protein interaction network of chromatin regulation–associated proteins identified in the oocyte proteome. Nodes represent proteins, with newly defined proteins indicated by blue-bordered circles. Red nodes represent proteins with higher MCODE scores, indicating highly interconnected proteins within the network. **b** Sankey plot showing the relationship between newly defined proteins (left) and functional categories (right). The size and color of the bubbles on the right correspond to the gene number (Count) and –$\log_{10}$(P-value), respectively, representing the enrichment level of each functional category. **c** Subnetwork of SWI/SNF-associated chromatin remodeling proteins. Newly defined proteins are indicated by blue-bordered circles. **d** Immunohistochemical staining of bromodomain containing 1 (BRD1) and polybromo 1 (PBRM1) in ovarian sections across follicle stages (primordial, primary, secondary, and antral). Arrows indicate oocytes. Scale bars, 20 μm. **e** Immunofluorescence staining of histone H3 lysine 14 acetylation (H3K14ac) (green) in ovarian sections across follicle stages. Merged images with DNA (magenta) are

shown. Arrows indicate oocytes. Scale bars, 20 μm. **f** Immunohistochemical staining of TANK binding kinase 1 (TBK1) and AP2 associated kinase 1 (AAK1) in ovarian sections across follicle stages. Arrows indicate oocytes. Scale bars, 20 μm. **g** Immunohistochemical staining of BAG cochaperone 6 (BAG6) and macrophage erythroblast attacher (MAEA) in ovarian sections across follicle stages. Arrows indicate oocytes. Scale bars, 20 μm.

Supplementary Figure 4

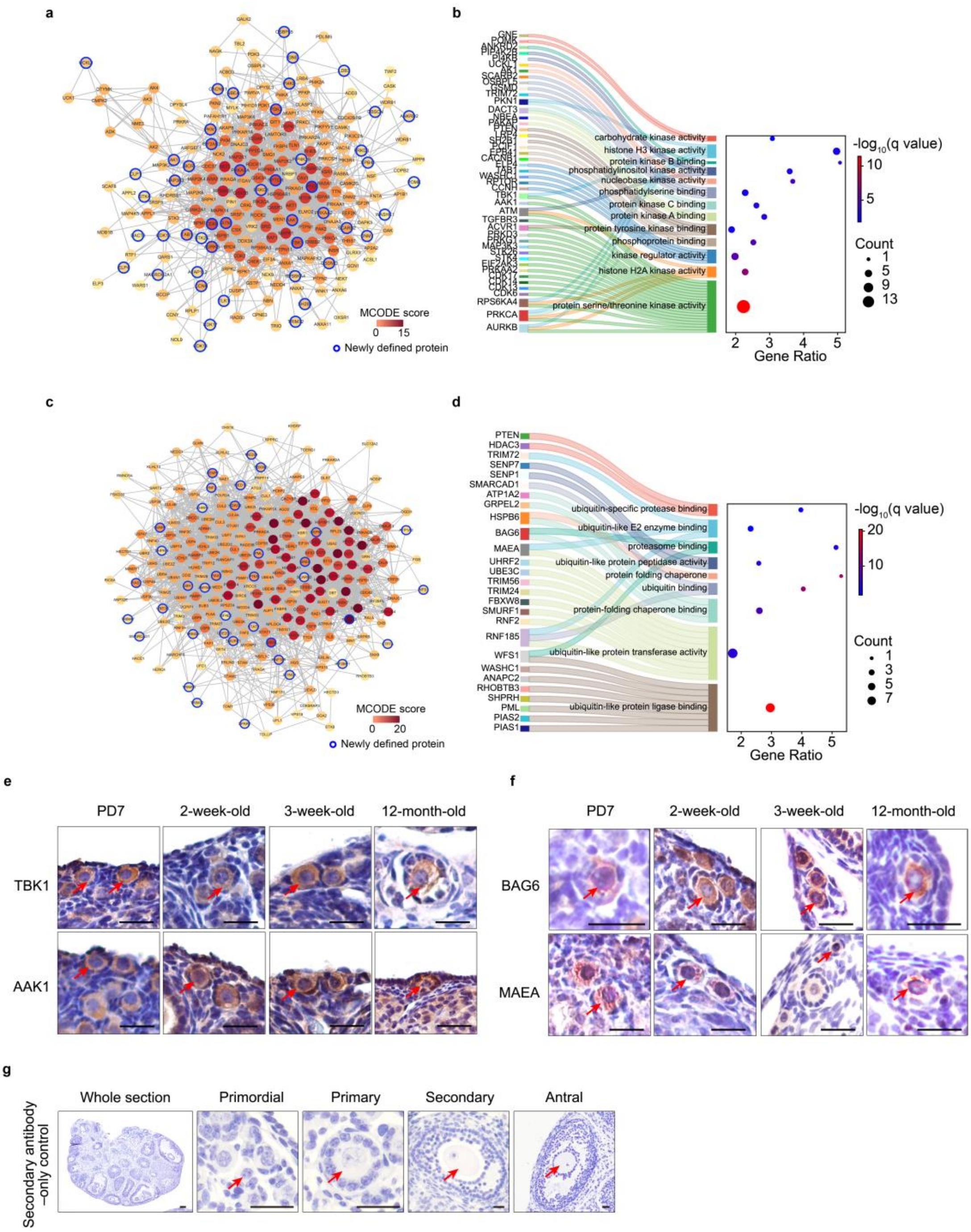

Supplementary Figure 4. Interaction networks and age-independent expression of regulatory proteins in oocytes. a Protein–protein interaction network of kinase-related regulatory proteins identified in the oocyte proteome. Nodes represent proteins, with newly defined proteins indicated by blue-bordered circles. Node color intensity reflects MCODE score. **b** Functional enrichment analysis of proteins in the kinase-associated network. The Sankey diagram links newly defined proteins (left) to enriched molecular function categories (right). In the bubble plot, bubble size represents protein count and color indicates $-\log_{10}$ (q value). **c** Protein–protein interaction network of proteins associated with protein stability and ubiquitin–proteasome regulation. Newly defined proteins are indicated by blue-bordered circles. Node color intensity reflects MCODE score. **d** Functional enrichment analysis of proteins in the protein homeostasis network. The Sankey diagram links newly defined proteins (left) to enriched molecular function categories (right). In the bubble plot, bubble size represents protein count and color indicates $-\log_{10}$ (q value). **e** Immunohistochemical staining of TANK binding kinase 1 (TBK1) and AP2 associated kinase 1 (AAK1) in ovarian sections from PD7, 2-week-old, 3-week-old, and 12-month-old mice. Arrows indicate primordial follicle oocytes. Scale bars, 20 µm. **f** Immunohistochemical staining of BAG cochaperone 6 (BAG6) and macrophage erythroblast attacher (MAEA) in ovarian sections from PD7, 2-week-old, 3-week-old, and 12-month-old mice. Arrows indicate primordial follicle oocytes. Scale bars, 20 µm. **g** Secondary antibody–only control staining across follicle stages in PD21 ovarian sections. Arrows indicate oocytes. Scale bars, 100 µm in 4× microscopy and 20 µm in enlarged views.

3. The authors should improve the clarity of their omics data presentation in the figures and figure legends. For instance, the meaning of the numbers in Figure 2I is unclear. Making figures and figure legends more reader-friendly would be beneficial.

Response: We thank the reviewer for this helpful suggestion. In the revised manuscript, we have comprehensively improved the clarity and readability of the omics data figures.

All figure legends have been rewritten to include clear definitions of axes, numerical annotations, and statistical values, making them self-explanatory and reader-friendly **(text lines 1039–1214 in revised manuscript)**. In particular, for Fig. 2i, we clarified the meaning of the concentric rings, which had caused ambiguity in the original version. In the revised figure legend, the innermost ring now clearly denotes the enrichment factor (gene ratio), the second ring indicates the number of identified genes (Count), the third ring reflects the statistical significance (–log10 q value), and the outermost ring lists the corresponding GO term IDs. This clarification has been incorporated into the figure legend **(text lines 1088–1091 in revised manuscript)**. Together, these improvements substantially enhance the accuracy and accessibility of the omics data presentation.

4. It would also be helpful if the authors provided a schematic figure in the final figure that illustrates the oocyte-specific molecular events following cisplatin treatment, based on their results. The current figure in Fig. 7 is more akin to a Graphical Abstract.

Response: We appreciate the reviewer's insightful suggestion. In the revised manuscript, we have added a new schematic figure **(Supplementary Fig. 8, text lines 470–473 in revised manuscript; Also see below)** to illustrate the key oocyte-specific molecular events following cisplatin treatment. This figure integrates our main findings into a concise model: cisplatin induces DNA double-strand breaks and activates the DDR pathway, while simultaneously promoting epigenetic remodeling (EZH2 accumulation and elevated H3K27me3). These changes lead to follicular damage, including apoptotic and necrotic primordial follicles, aberrant follicular activation, and meiotic maturation defects characterized by reduced polar body extrusion, spindle abnormalities, and prolonged SAC activity. Importantly, these defects were partially rescued by the EZH2 inhibitor GSK126. This schematic highlights the essential molecular alterations and functional consequences of cisplatin exposure in primordial follicle oocytes, thereby providing a clearer mechanistic overview for readers.

Supplementary Figure 8

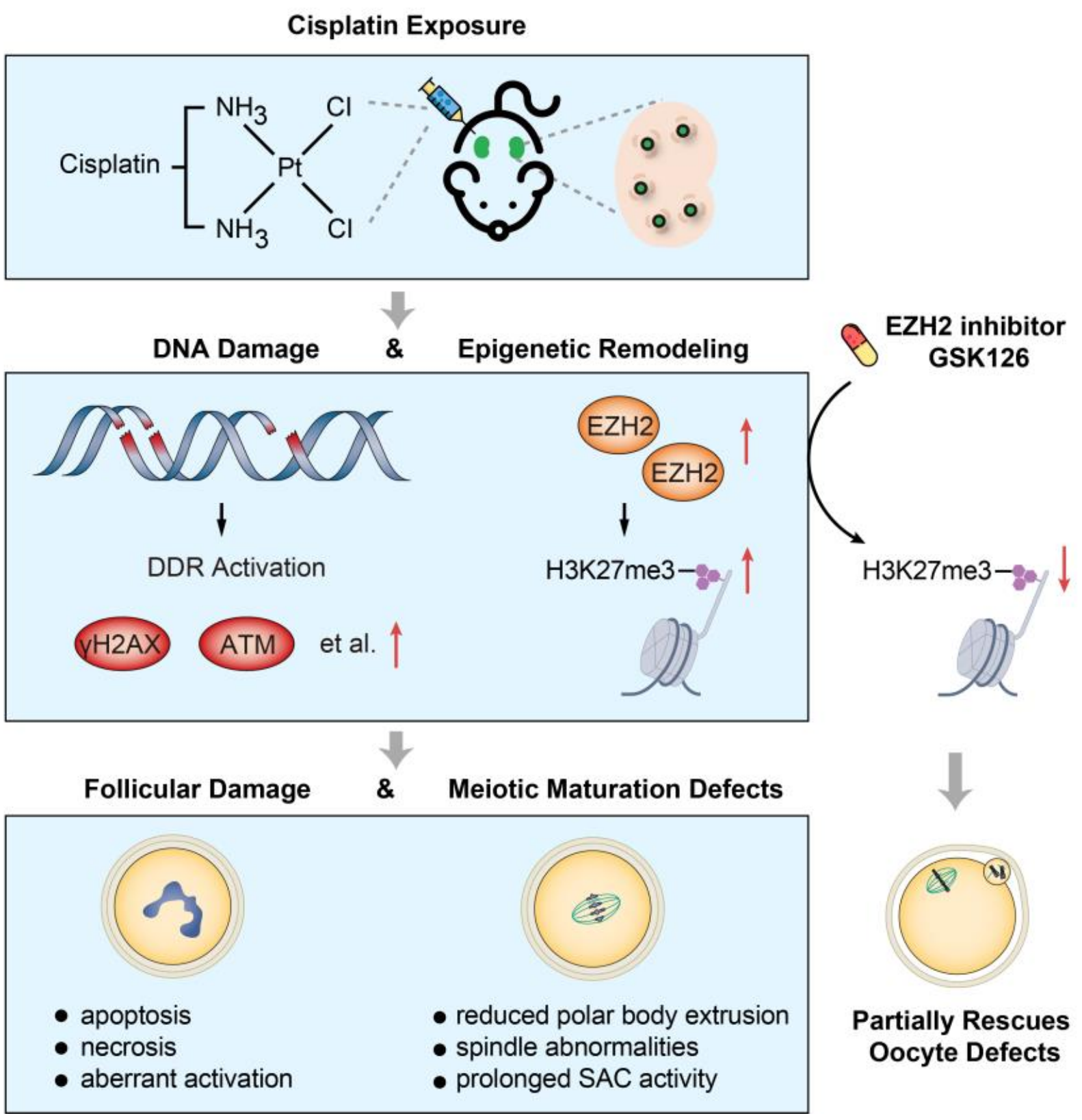

Supplementary Figure 8. Schematic model illustrating oocyte-specific molecular events following cisplatin treatment. Cisplatin induces DNA double-strand breaks in primordial follicle oocytes and activates the DNA damage response (DDR) pathway. Concurrently, cisplatin promotes epigenetic remodeling, characterized by EZH2 accumulation and increased H3K27me3 levels. These alterations lead to follicular damage, including apoptosis and necrosis of primordial follicles, aberrant follicular activation, and meiotic maturation defects such as reduced polar body extrusion, spindle abnormalities, and prolonged spindle assembly checkpoint (SAC) activity. Treatment with the EZH2 inhibitor GSK126 partially rescues these defects.

5. in the Data Availability section, the authors described that the proteomics data has been deposited to the ProteomeXchange Consortium (https://proteomecentral.proteomexchange.org/cgi/GetDataset?ID=PXD048332).

However, it appears that a username and password are required to access dataset PXD048332. The authors should revise the access option to allow public access to their raw data.

Response: We thank the reviewer for pointing this out. We have now updated the access settings for our dataset to ensure it is publicly available without the need for login credentials. All raw files and search results for MS of proteomics have been deposited in ProteomeXchange (RRID:SCR_004055) via iProX (www.iprox.org) with the identification no. IPX0015821000 (for iProX).

6. All the supplementary dataset files (Excel files) should include Uniprot ID and Entrez Gene ID (NCBI Gene ID). Additionally, it would be beneficial to provide an additional supplementary dataset file (Excel file) containing all peptide information (e.g. analysed peptide sequence, MS1 intensity, sequence matched protein name, Uniprot ID, NCBI Gene ID, etc) per each replicate sample before data processing. This data may help to understand real mass result and quality of the data.

Response: We appreciate the reviewer's valuable suggestion. In the revised submission, all protein-level supplementary Excel files **(Supplementary Data 1 and 5)** have been updated to include both Uniprot accession numbers and NCBI Entrez Gene IDs for each identified protein. In addition, we have provided a new supplementary dataset **Supplementary Data 8)** that contains comprehensive peptide-level information for each replicate sample prior to data processing, including the analysed peptide sequences, MS1 intensity values, matched protein names, Uniprot accession numbers, and NCBI Gene IDs. These updates will enable readers to directly assess the raw peptide evidence and better evaluate the reliability and quality of our mass spectrometry data.

Reviewer #2:

In their manuscript, Jiang et al. introduce a novel mouse model for Cre-driven APEX-

mediated proteome labeling in vivo. The model enables identification of cell-type-specific changes in protein abundance during development and in response to treatments. Using this method, authors aim to identify therapeutic targets for fertility preservation in cancer patients. First, the authors demonstrated that this system specifically labels oocytes with APEX and EGFP driven by Gdf9-Cre recombinase. Next, they demonstrated that APEX-labelled proteins, as well as RNAs, were effectively pulled down and identified using mass spectrometry (MS) and RNA sequencing (RNAseq), respectively. Finally, they applied APEX to identify proteins differentially expressed in response to short-term and low-dose treatment with chemotherapy drug cisplatin. Using this approach, authors identified EZH2 as one of the proteins that changed abundance in oocytes after cisplatin treatment and thus could be a target for fertility preservation. To test this, authors used EZH2 inhibitor GSK126 and showed that it prevents cisplatin-induced maturation defects in fully grown oocytes. While this novel mouse model is poised to be a very valuable and powerful tool for the study of oocyte proteomes and responses to a variety of conditions, concerns related to experimental design and data interpretation need to be addressed. Specifically, based on presented data, it remains unclear that the proteomes and transcriptomes captured by APEX originate from primordial follicle stage oocytes. Addressing this and other concerns will strengthen the manuscript's impact and relevance.

Specific comments

1. Expression of APEX system in oocytes: EGFP expression driven by Gdf9-Cre is evident in oocytes; however, to confirm that all oocytes were captured, authors should perform co-immunostaining with a known oocyte marker such as DDX4 in whole ovary and on sections. For example, in Figure 2C, primordial follicles don't seem to be labeled in the PD21 ovary while growing follicles from the first wave of folliculogenesis are clearly labeled. Therefore, there may be some quiescent primordial follicles from the second wave of folliculogenesis that may not yet express Gdf9-Cre. It is also concerning that MS and RNAseq of APEX-labelled

proteome and transcriptome lack major markers of quiescent oocytes but include many proteins for fully grown oocytes (FGO). See other comments below.

Response: We thank the reviewer for this important comment. To confirm that APEX expression captures all oocytes, we performed co-immunostaining of Streptavidin with the well-established oocyte marker DDX4. Substantial overlap of Streptavidin and DDX4 signals was observed in histological sections at both PD7 and PD21 **(Fig. 2c, text lines 173–182 in revised manuscript; Also see below)**, indicating that oocytes, including those in primordial follicles, were labeled by *Gdf9*-Cre–driven APEX expression. Notably, Streptavidin-positive signals were also detected in quiescent primordial follicles at PD21, suggesting that although *Gdf9*-Cre activity is weaker in these oocytes, it remains sufficient for APEX-mediated labeling.

In the revised proteomic dataset obtained from re-performed mass spectrometry experiments, well-recognized primordial oocyte markers (e.g., SOHLH1, NOBOX, DDX4), as well as additional functionally relevant proteins (e.g., DDB1, EIF4E, YTHDF2) were identified, with enrichment further confirmed by Western blotting **(Supplementary Fig. 3d, text lines 225–234 in revised manuscript; Also see below)**. Similarly, APEX-seq detected primordial transcripts including *Ddx4*, *Gdf9*, *Bmp15*, *Dazl*, *Mos*, *Zar1*, *Sohlh1*, and *Nobox*, together with additional oocyte-associated genes involved in early oocyte development and meiotic programs (*Padi6*, *Sycp1*, *Sycp3*, and *Stag3*), which were validated by RT-qPCR **(Fig. 4c, text lines 311–320 in revised manuscript; Also see below)**. Taken together, these results demonstrate that both proteomic and transcriptomic analyses capture quiescent as well as growing oocytes, confirming that APEX expression under *Gdf9*-Cre effectively labels primordial follicle oocytes.

Figure 2

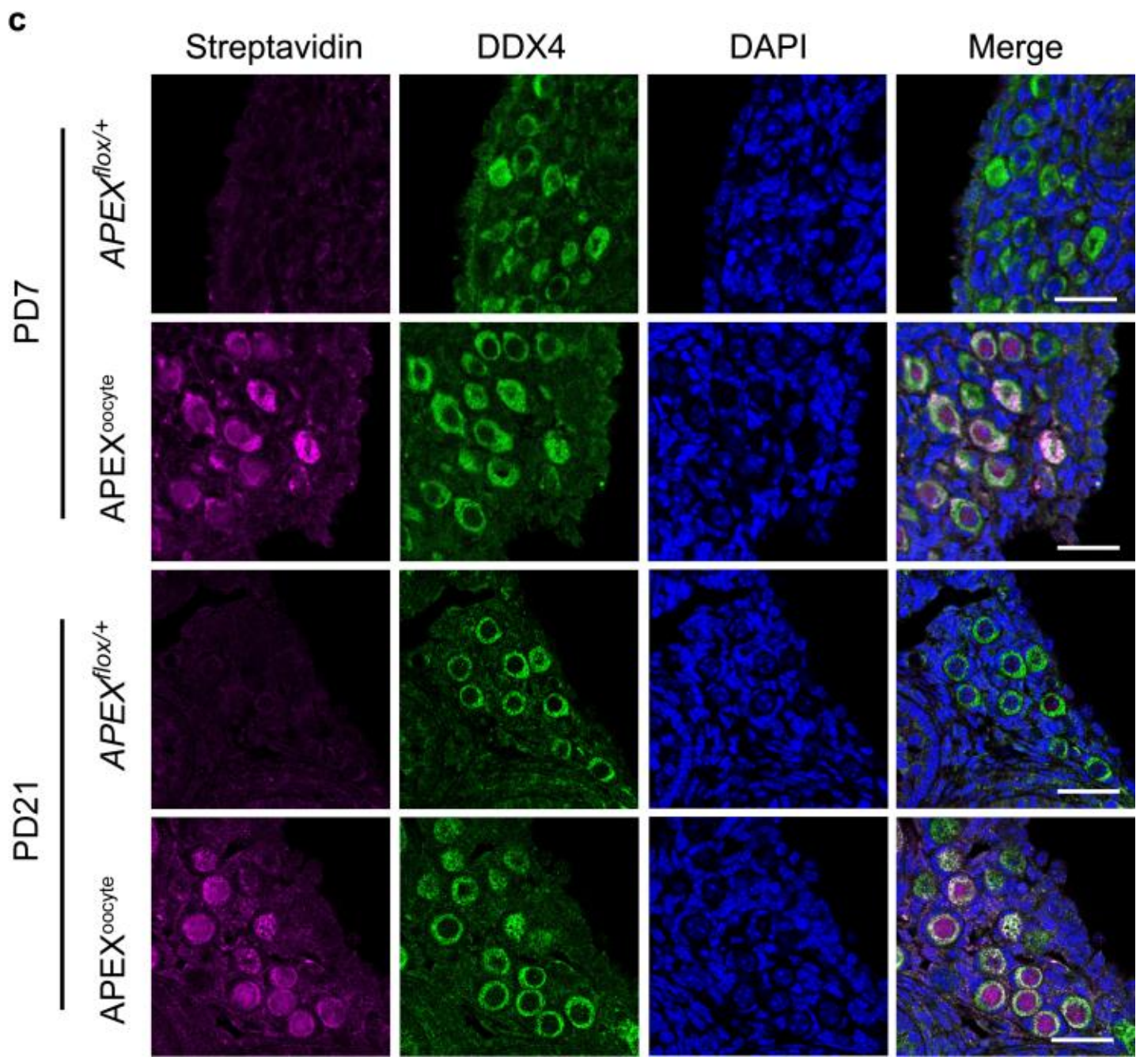

Figure 2. *In situ* APEX-based proteomic profiling of oocytes from early-stage ovarian follicles. c Streptavidin labeling in ovarian sections at PD7 and PD21 in APEX^{oocyte} and *APEX*$^{flox/+}$ mice, respectively. Co-staining for streptavidin (magenta), DDX4 (green), and DAPI (blue) is shown. Scale bars, 50 μm.

Supplementary Figure 3

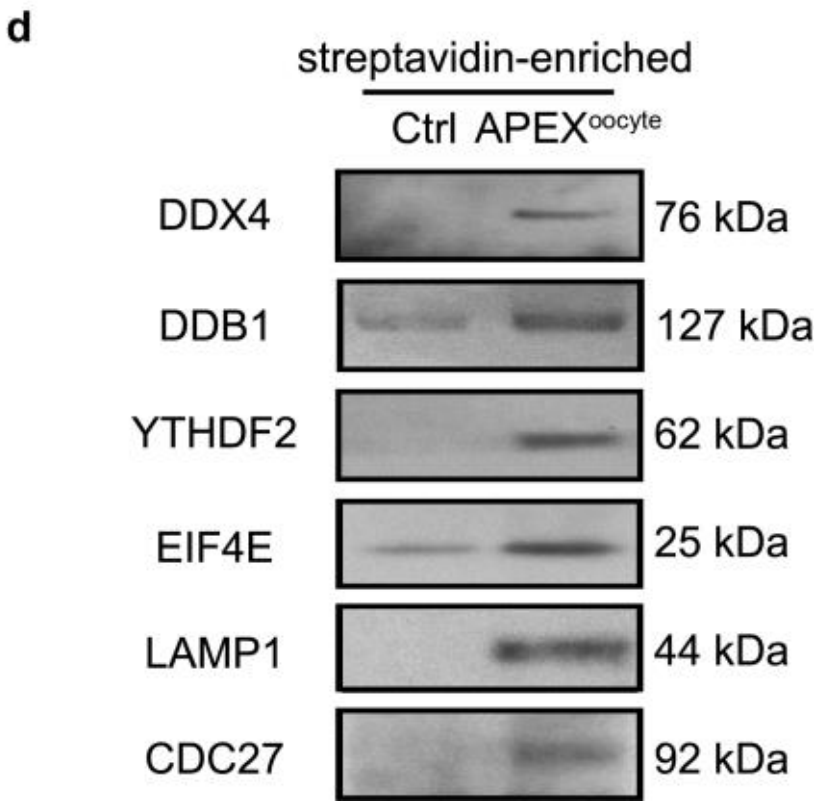

Supplementary Figure 3. Quality assessment and validation of the streptavidin-enriched oocyte proteome. d Western blot analysis of representative proteins in streptavidin-enriched fractions from control (Ctrl) and APEX^{oocyte} ovaries.

Figure 4

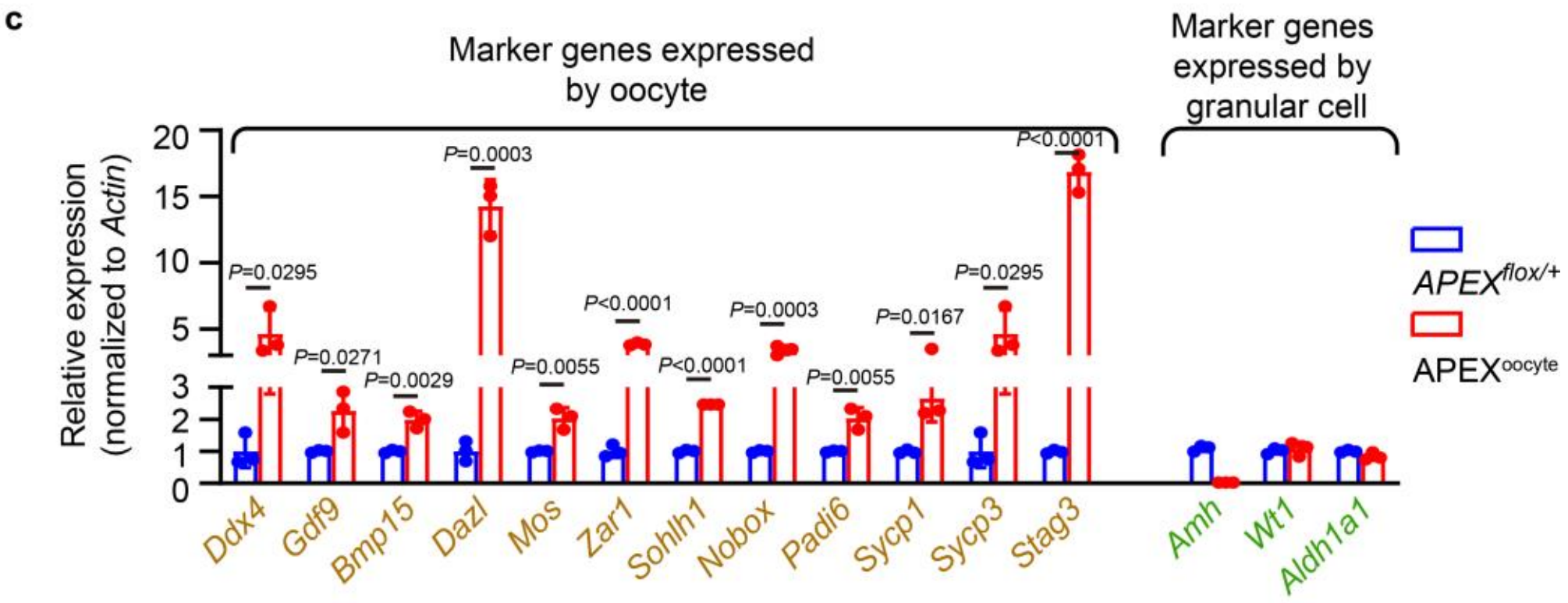

Figure 4. Characterization of the transcriptome of early follicle oocytes using APEX^{oocyte} labeling system. c Quantitative PCR (qPCR) analysis of oocyte- and granulosa cell-specific marker genes in biotinylated RNAs from APEX^{oocyte} mice. Strong enrichment of oocyte-specific transcripts (e.g., *Ddx4, Gdf9, Bmp15*) and minimal enrichment of granulosa cell markers (e.g., *Amh*). Three biologically independent replicates were performed. Mean ± SEM. *P* values are calculated by unpaired two-tailed Student's t tests.

2. APEX-labeled proteome in primordial follicle oocytes: Authors use PD7 ovaries to identify proteins expressed in primordial follicle oocytes. Based on that, authors conclude that some proteins previously detected at later stages in FGO, including meiotic maturation, are already present in primordial follicle stage oocytes. However, it is important to note that the pulled-down proteins may originate from large-growing primary follicles present in PD7 ovaries (as seen in Figure 2C). Immunohistochemistry on full sections confirms that these proteins are most abundant in growing primary and secondary follicles. Additionally, some instances in Figure 3, chosen to illustrate protein expression in primordial follicles, actually depict primary follicles (e.g., Fig. 3F for GOPC staining or Fig. S2F for DNMT1). Fig.3B for NDUFA5 does not really look like a primordial or even a follicle).

Response: We thank the reviewer for these insightful comments. We acknowledge that PD7 ovaries, while predominantly composed of primordial follicles, still contain a small proportion of growing primary follicles, which may contribute to some of the pulled-down proteins. To minimize this potential influence, we have shortened the BP incubation time (1h) to limit substrate diffusion into inner growing follicles, thereby minimizing unintended labeling and ensuring that the proteomic signals predominantly reflect primordial oocytes. We also increased the biological input to 12 ovaries per replicate (previously 5), optimized the enrichment procedure with stringent wash steps (1 M KCl, 0.1 M Na_2CO_3, and 2 M urea, each twice) to reduce nonspecific binding, and analyzed the samples on an updated mass spectrometer, which improved peptide coverage and quantitative accuracy.

In the revised dataset, a total of 2,772 proteins were designated as a high-confidence oocyte proteome associated with primordial and early-stage ovarian follicles (fold change > 1.5, $P < 0.05$). Functional enrichment analyses demonstrated that these proteins are strongly aligned with key biological features of early oocytes, including chromatin regulation, RNA metabolism, kinase-mediated regulation, and protein stability control **(Supplementary Data 1, Figs. 2e-i; Also see below)**. To further reduce potential confounding from labeling of growing follicles, we specifically focused on proteins that had not been detected in previously published proteomic datasets of fully grown oocytes. Multiple well-recognized primordial and early oocyte–associated proteins were identified, and representative proteins from major functional categories were further examined by streptavidin pull-down followed by Western blotting and immunohistochemical analyses. Among these, chromatin regulatory proteins associated with histone H3 lysine 14 acetylation (H3K14ac), including polybromo 1 (PBRM1) and bromodomain containing 1 (BRD1), showed relatively higher abundance in primordial follicle oocytes, followed by a decline at later follicular stages, consistent with the elevated H3K14ac levels observed in primordial oocytes. In parallel, representative proteins from other enriched categories, including the kinases TANK-binding kinase 1 (TBK1) and AP2-associated kinase 1 (AAK1), as well as protein homeostasis regulators such as BAG cochaperone 6 (BAG6) and macrophage

erythroblast attacher (MAEA), were detectable in oocytes across folliculogenesis, with enrichment evident at early follicular stages **(Fig. 3, Supplementary Figs. 3d-e, Supplementary Fig. 4; Also see below)**. Together, these analyses provide strong support for the reliability and biological relevance of the revised proteomic dataset.

We also carefully re-examined the histological figures and confirmed that the follicle stages shown in the current images are correctly identified. Taken together, these analyses support the reliability of the revised dataset and its relevance to early-stage oocyte biology. We have added these data to the **revised Figure (Figs. 2e-i, Fig. 3, Supplementary Figs. 3d-e, Supplementary Fig. 4; also see below)** and updated **the corresponding text (lines 207–302)** in the Results section.

Figure 2

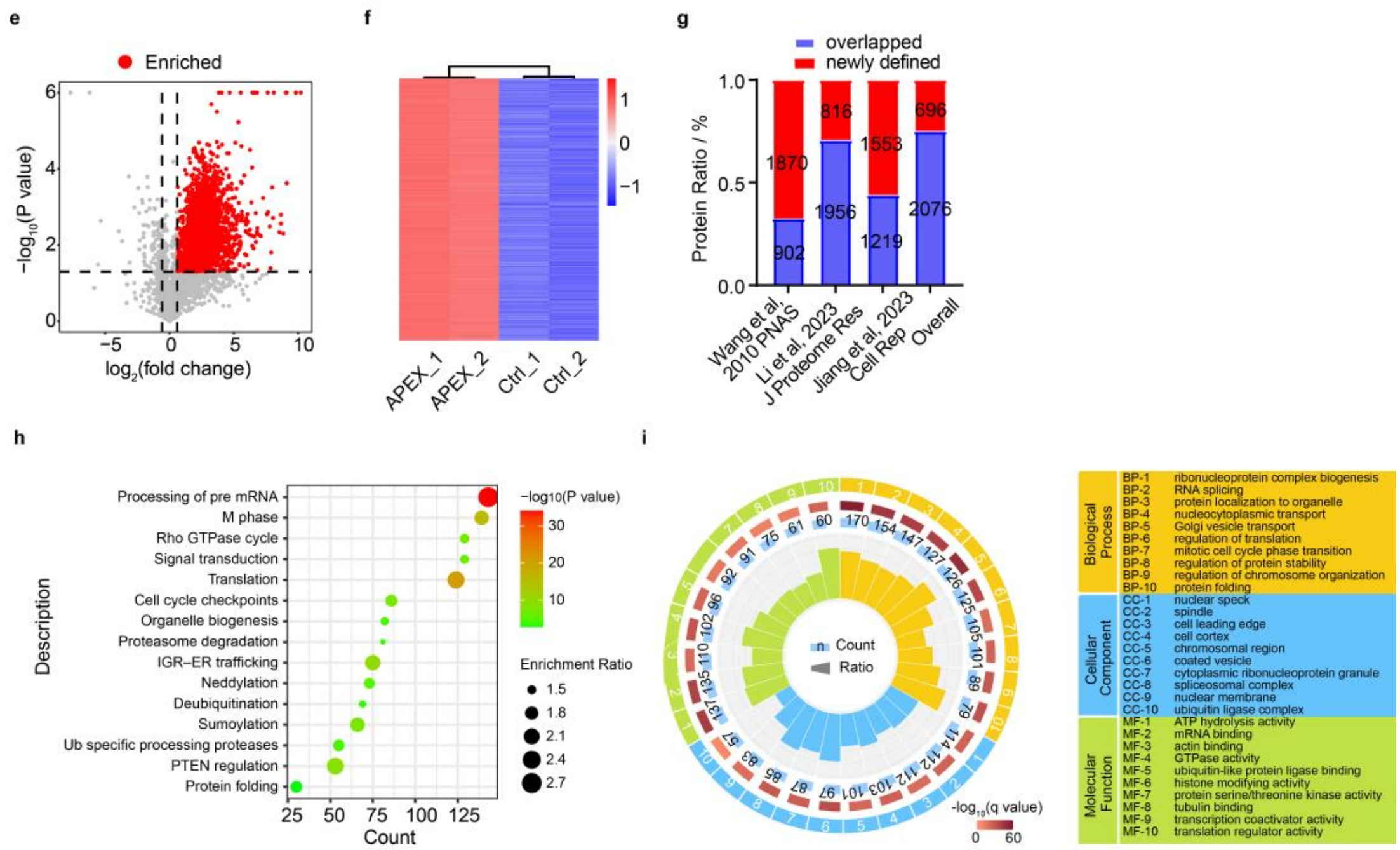

Figure 2. *In situ* APEX-based proteomic profiling of oocytes from early-stage ovarian follicles. e Volcano plot of proteins detected by LC–MS/MS comparing APEX^{oocyte} and *APEX*$^{flox/+}$ control samples. Significantly enriched proteins are highlighted (red dots, fold change > 1.5, $P < 0.05$). **f** Heatmap of enriched proteins across biological replicates of APEX^{oocyte} and *APEX*$^{flox/+}$ (Ctrl) samples. **g** Overlap analysis of the identified proteome with previously published GV-stage oocyte proteomes. Stacked bars indicate overlapped and newly defined proteins. **h** Bubble plot of enriched Reactome pathways. The x-axis shows the gene number (Count), the y-axis

lists pathway names, bubble size reflects enrichment ratio, and color indicates significance (–$\log_{10}$ [P-value]). **i** Circular plot of the top 10 enriched gene ontology (GO) terms in biological process (yellow), cellular component (blue), and molecular function (green). From inside to outside: enrichment factor (Ratio), gene number (Count), statistical significance (–$\log_{10}$ [q-value]), and GO term IDs corresponding to the descriptions on the right.

Supplementary Figure 3

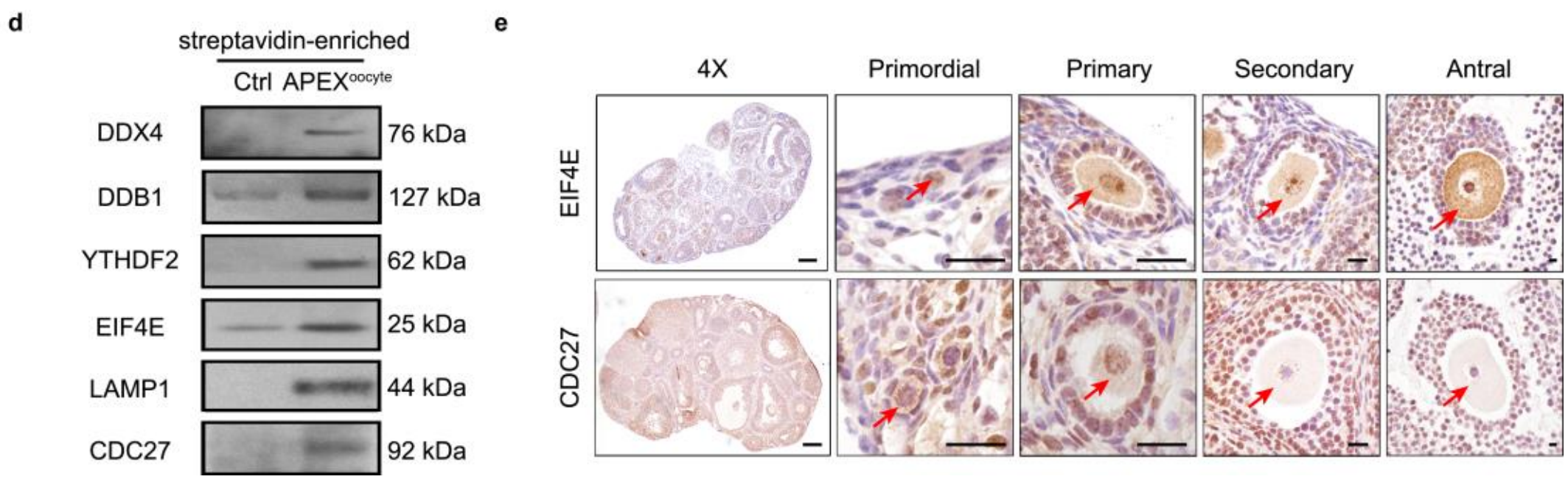

Supplementary Figure 3. Quality assessment and validation of the streptavidin-enriched oocyte proteome. d Western blot analysis of representative proteins in streptavidin-enriched fractions from control (Ctrl) and APEX^{oocyte} ovaries. **e** Immunohistochemical staining of eukaryotic translation initiation factor 4E (EIF4E) and cell division cycle 27 (CDC27) in PD21 ovarian sections. Images include low-magnification overview and higher-magnification views of primordial, primary, secondary, and antral follicles. Arrows indicate oocytes. Scale bars, 100 μm in 4× microscopy and 20 μm in enlarged views.

Figure 3

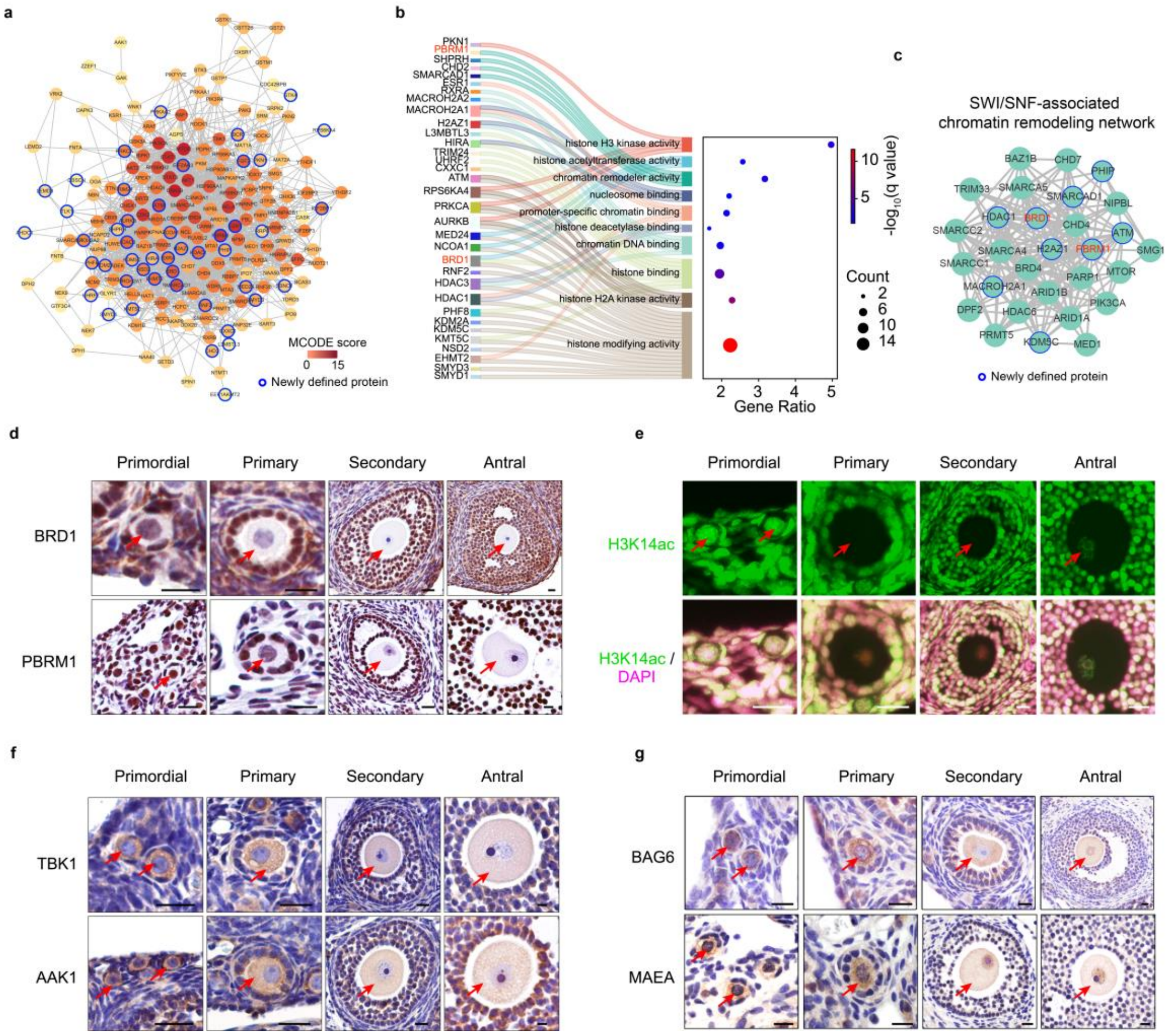

Figure 3. Expression dynamics of representative regulatory proteins in oocytes. a Protein–protein interaction network of chromatin regulation–associated proteins identified in the oocyte proteome. Nodes represent proteins, with newly defined proteins indicated by blue-bordered circles. Red nodes represent proteins with higher MCODE scores, indicating highly interconnected proteins within the network. **b** Sankey plot showing the relationship between newly defined proteins (left) and functional categories (right). The size and color of the bubbles on the right correspond to the gene number (Count) and –log(P-value), respectively, representing the enrichment level of each functional category. **c** Subnetwork of SWI/SNF-associated chromatin remodeling proteins. Newly defined proteins are indicated by blue-bordered circles. **d** Immunohistochemical staining of bromodomain containing 1 (BRD1) and polybromo 1 (PBRM1) in ovarian sections across follicle stages (primordial, primary, secondary, and antral). Arrows indicate oocytes. Scale bars, 20 μm. **e** Immunofluorescence staining of histone H3 lysine 14 acetylation (H3K14ac) (green) in ovarian sections across follicle stages. Merged images with DAPI (magenta) are

shown. Arrows indicate oocytes. Scale bars, 20 μm. **f** Immunohistochemical staining of TANK binding kinase 1 (TBK1) and AP2 associated kinase 1 (AAK1) in ovarian sections across follicle stages. Arrows indicate oocytes. Scale bars, 20 μm. **g** Immunohistochemical staining of BAG cochaperone 6 (BAG6) and macrophage erythroblast attacher (MAEA) in ovarian sections across follicle stages. Arrows indicate oocytes. Scale bars, 20 μm.

Supplementary Figure 4

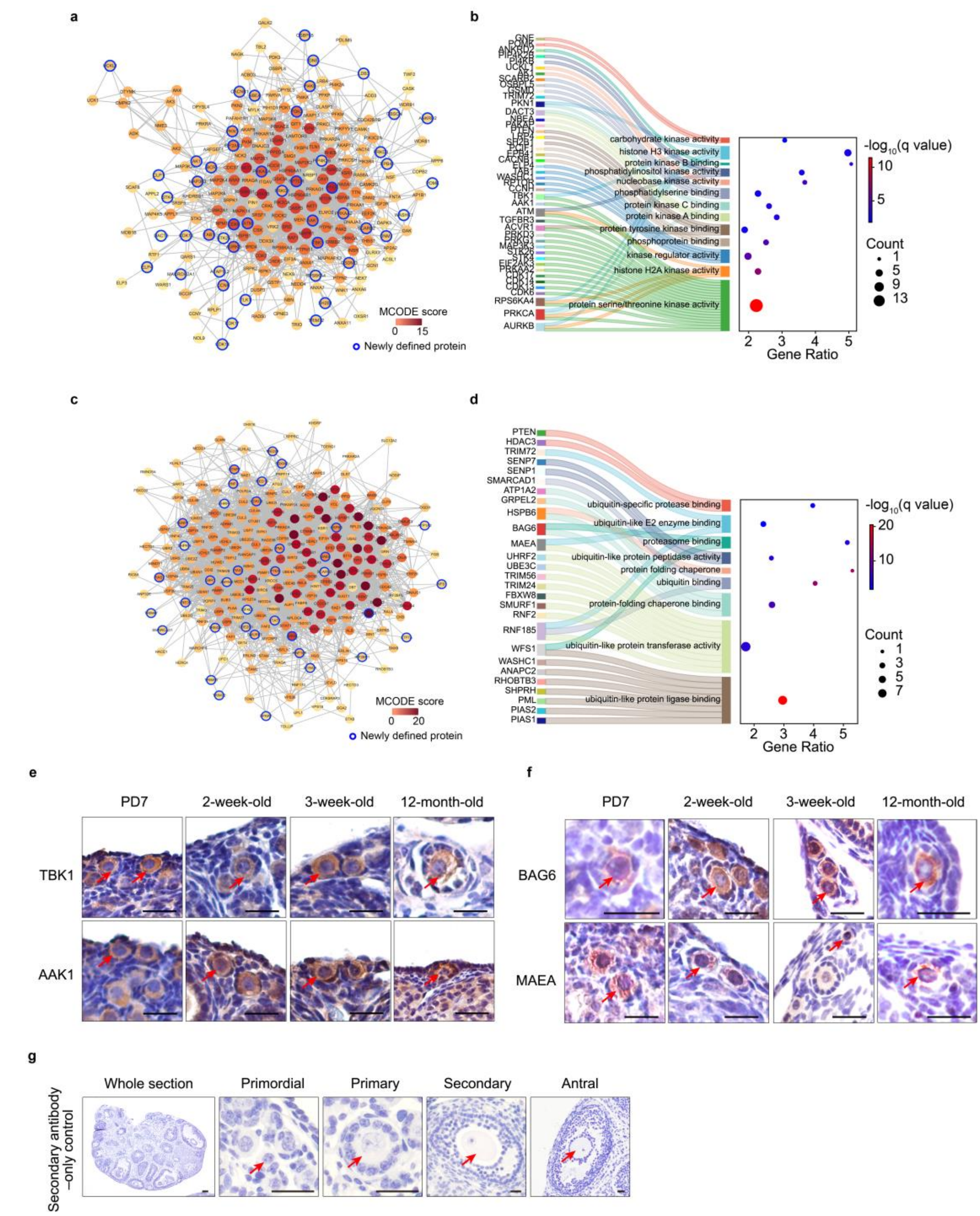

Supplementary Figure 4. Interaction networks and age-independent expression of regulatory proteins in oocytes. a Protein–protein interaction network of kinase-related regulatory proteins identified in the oocyte proteome. Nodes represent proteins, with newly defined proteins indicated by blue-bordered circles. Node color intensity reflects MCODE score. **b** Functional enrichment analysis of proteins in the kinase-associated network. The Sankey diagram links newly defined proteins (left) to enriched molecular function categories (right). In the bubble plot, bubble size represents protein count and color indicates $-\log_{10}$ (q value). **c** Protein–protein interaction network of proteins associated with protein stability and ubiquitin–proteasome regulation. Newly defined proteins are indicated by blue-bordered circles. Node color intensity reflects MCODE score. **d** Functional enrichment analysis of proteins in the protein homeostasis network. The Sankey diagram links newly defined proteins (left) to enriched molecular function categories (right). In the bubble plot, bubble size represents protein count and color indicates $-\log_{10}$ (q value). **e** Immunohistochemical staining of TANK binding kinase 1 (TBK1) and AP2 associated kinase 1 (AAK1) in ovarian sections from PD7, 2-week-old, 3-week-old, and 12-month-old mice. Arrows indicate primordial follicle oocytes. Scale bars, 20 μm. **f** Immunohistochemical staining of BAG cochaperone 6 (BAG6) and macrophage erythroblast attacher (MAEA) in ovarian sections from PD7, 2-week-old, 3-week-old, and 12-month-old mice. Arrows indicate primordial follicle oocytes. Scale bars, 20 μm. **g** Secondary antibody–only control staining across follicle stages in PD21 ovarian sections. Arrows indicate oocytes. Scale bars, 100 μm in 4× microscopy and 20 μm in enlarged views.

a. Furthermore, MS of APEX-labelled proteins (Table S1) failed to detect many known markers abundantly expressed in primordial follicle oocytes, including TRP63, SOHLH1/2, and NOBOX. Authors should comment on the limitations of their approach and why these markers were not identified but DDX4 was. Is it possible that click chemistry is not efficient in primordial follicle oocytes? To confirm that APEX labels primordial oocyte proteins authors should show by

western blot that these markers are present in pulled-down proteomes, e.g., in Fig.2D.

Response: We thank the reviewer for raising this important point. In the revised proteomic dataset obtained from re-performed mass spectrometry experiments, several well-recognized primordial oocyte markers, including SOHLH1 (FC = 2.23), NOBOX (FC = 3.22), and DDX4 (FC = 4.38), were significantly enriched. In addition, multiple proteins known to be expressed or functionally implicated in primordial-stage oocytes, such as DDB1 (FC = 6.12), EIF4E (FC = 6.62), and YTHDF2 (FC = 5.34), were also detected, with enrichment further confirmed by Western blotting **(Supplementary Fig. 3d, text lines 225–234 in revised manuscript; Also see below)**.

The underrepresentation of some markers in the MS dataset likely reflects multiple factors. Highly abundant and stable oocyte proteins (e.g., DDX4) are more readily detected, whereas low-abundance transcriptional regulators such as TRP63 and SOHLH2 are inherently more difficult to capture in proteomic studies. In addition, APEX-dependent biotinylation is influenced by the local structural environment and the accessibility of reactive residues (primarily tyrosines), which can vary among proteins and affect their recovery.

Taken together, the updated dataset, complemented by Western blot validation, provides a refined protein list that highlights factors present in primordial follicle oocytes.

Supplementary Figure 3

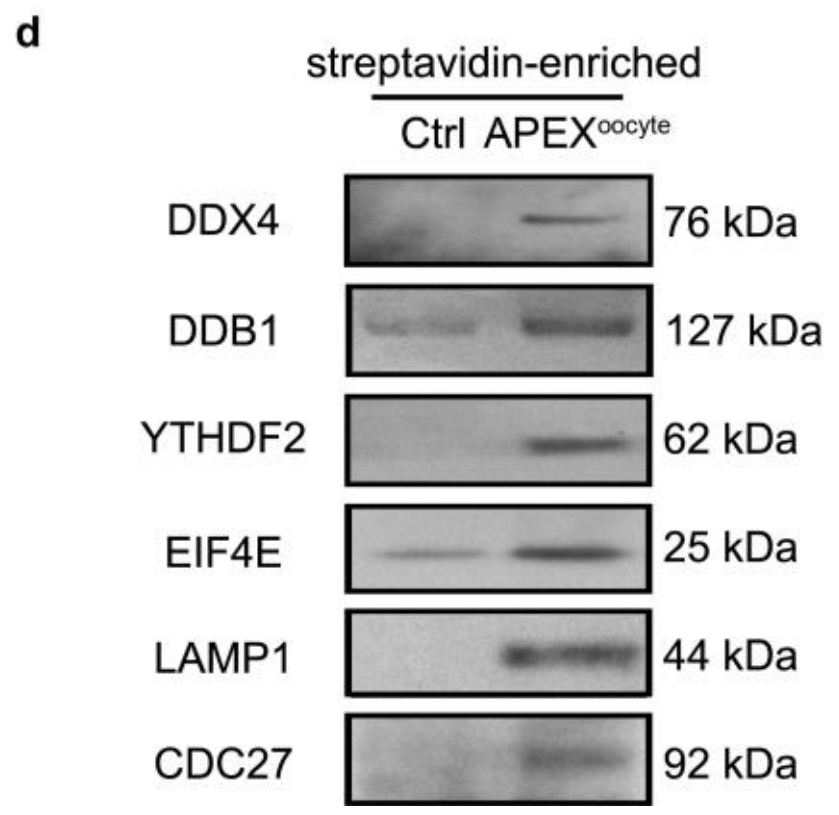

Supplementary Figure 3. Quality assessment and validation of the streptavidin-enriched oocyte proteome. d Western blot analysis of representative proteins in streptavidin-enriched fractions from control (Ctrl) and APEX^{oocyte} ovaries.

b. Similar lack of primordial oocyte markers in transcriptome data: In lines 289-291: authors state that "a large number of germ cell markers were included in the transcriptome"; however, Table S2 lacked major oocyte marker genes such as Nobox, Sohlh1/2, and Trp63. Authors should mark few classical germ cell and granulosa marker genes in the plot in Fig. S4E and validate their presence by QPCR in pulled down RNA.

Response: We thank the reviewer for this valuable suggestion. After re-examining our transcriptome dataset **(Supplementary Data 3)**, we found that APEX-seq detected several primordial oocyte transcripts, including *Ddx4* (FC = 2.02), *Gdf9* (FC = 1.51), *Bmp15* (FC = 1.87), *Dazl* (FC = 1.75), *Mos* (FC = 1.97), *Zar1* (FC = 1.56), *Sohlh1* (FC = 1.67), and *Nobox* (FC = 1.96), together with additional oocyte-associated genes involved in early oocyte development and meiotic programs, *Padi6* (FC = 1.64), *Sycp1* (FC = 2.20), *Sycp3* (FC = 2.03), and *Stag3* (FC = 3.48). These findings were further validated by RT-qPCR **(Fig. 4c, text lines 311–320 in revised manuscript; Also see below)**. In addition, we have revised **Supplementary Fig. 5e (Also see below)** to highlight several classical germ cell and granulosa markers, thereby demonstrating that RNA from primordial oocytes was indeed captured and enriched following APEX labeling.

We acknowledge that some canonical oocyte markers (e.g., *Sohlh2*, *Trp63*) were underrepresented in Supplementary Data 3. This likely reflects their low abundance in primordial oocytes at PD7 and the sensitivity limits of bulk RNA-seq in rare cell populations. Nevertheless, the combined transcriptome annotation and qPCR validation confirm that our dataset provides a representative profile of early follicle oocytes.

Figure 4

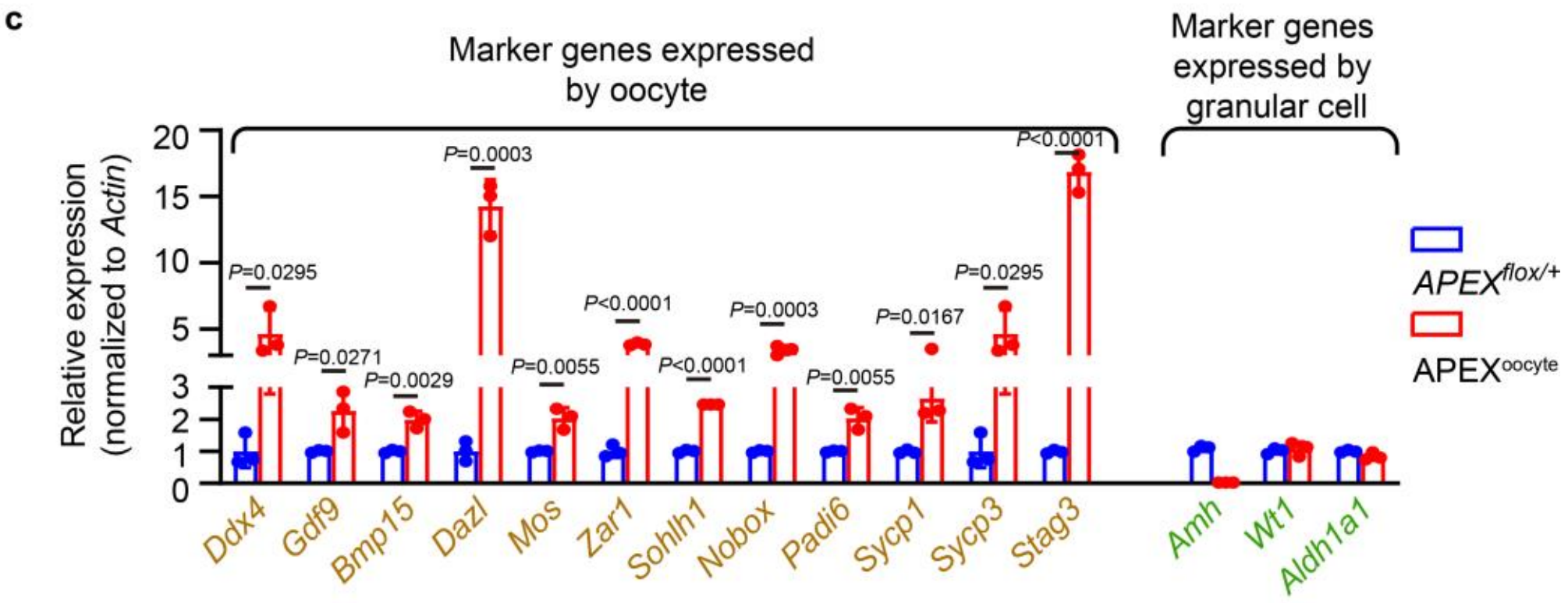

Figure 4. Characterization of the transcriptome of early follicle oocytes using APEX^oocyte labeling system. c Quantitative PCR (qPCR) analysis of oocyte- and granulosa cell-specific marker genes in biotinylated RNAs from APEX^oocyte mice. Strong enrichment of oocyte-specific transcripts (e.g., *Ddx4, Gdf9, Bmp15*) and minimal enrichment of granulosa cell markers (e.g., *Amh*). Three biologically independent replicates were performed. Mean ± SEM. *P* values are calculated by unpaired two-tailed Student's t tests.

Supplementary Figure 5

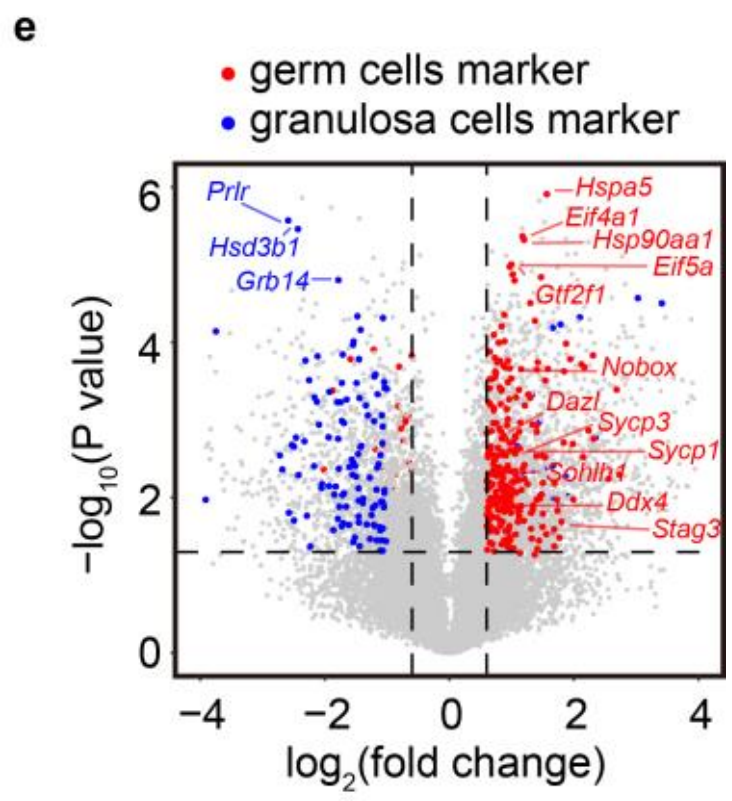

Supplementary Figure 5. Quality assessment and cellular specificity of the APEX-enriched oocyte transcriptome. e Volcano plot highlighting germ cell marker genes (red) and granulosa cell marker genes (blue) in the comparison between APEX^oocyte and control transcriptomes.

3. In Fig. S4I and lines 295-299, the authors report that APEX-transcriptome sequencing failed to overlap with ~50% of proteins detected in oocytes. This unexpected finding—where proteins are present without corresponding RNA—warrants discussion. The limitations of APEX labeling should be acknowledged, and potential reasons for this discrepancy explained.

Response: We thank the reviewer for raising this important point. Overall, the RNA and protein expression profiles were positively correlated **(Supplementary Fig. 5i, text lines 339–342 in revised manuscript; Also see below)**, yet a subset of genes displayed weak RNA–protein concordance, suggesting extensive post-transcriptional regulation and an intermediate state of gene expression during oocyte development. The apparent discrepancy between proteomic and transcriptomic datasets can be attributed to both biological and methodological factors. At the biological level, mRNA and protein abundances are often decoupled in oocytes. In our dataset, for example, PBRM1 and AAK1 were detected at the protein level by mass spectrometry and validated by immunohistochemistry, whereas their corresponding transcripts were not enriched in the APEX-seq dataset that was further validated by qRT-PCR **(Fig. R1a; See below)**. Conversely, *Gdf11, Nsun7,* and *Papd5* transcripts were enriched in RNA-seq but the protein was not detected in the proteome, a result corroborated by qPCR validation of RNA presence **(Fig. R1b; See below)**. These observations are consistent with well-documented cases in oocyte biology, such as maternal mRNAs regulated by CPEB1 (e.g., *Btg4*) that remain untranslated until GVBD [1], or proteins like CFP1 and IMP2 that show asynchronous degradation of RNA and protein across oocyte maturation [2, 3]. Together, these findings highlight that both delayed translation of stored transcripts and prolonged stability of legacy proteins can give rise to mismatches between RNA and protein detection. From a methodological perspective, the transcriptomic data were derived from a very limited input of primordial oocytes. Although RNA-seq is highly sensitive due to amplification, stochastic dropout of low-abundance transcripts can occur in rare cell populations. On the other hand, mass spectrometry may preferentially detect stable or abundant proteins, contributing to partial mismatch between RNA and protein. Overall, the divergence observed between proteomic and transcriptomic

signals is consistent with known features of oocyte biology and the inherent limitations of analyzing small cell populations.

Supplementary Figure 5

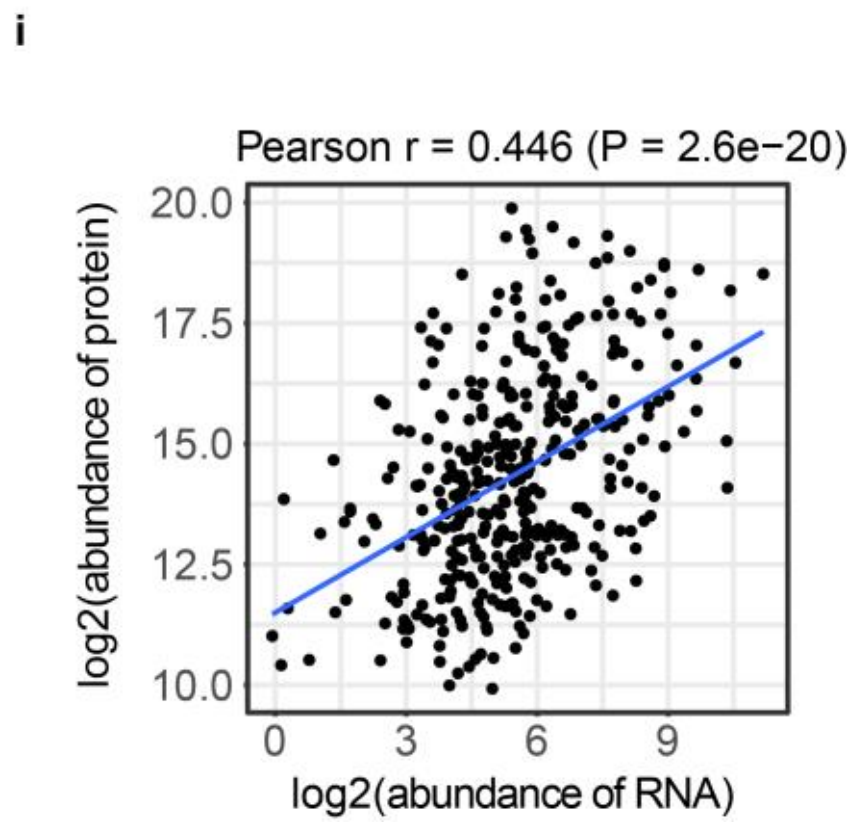

Supplementary Figure 5. Quality assessment and cellular specificity of the APEX-enriched oocyte transcriptome. i Correlation analysis between RNA abundance and protein abundance across shared genes.

Figure R1

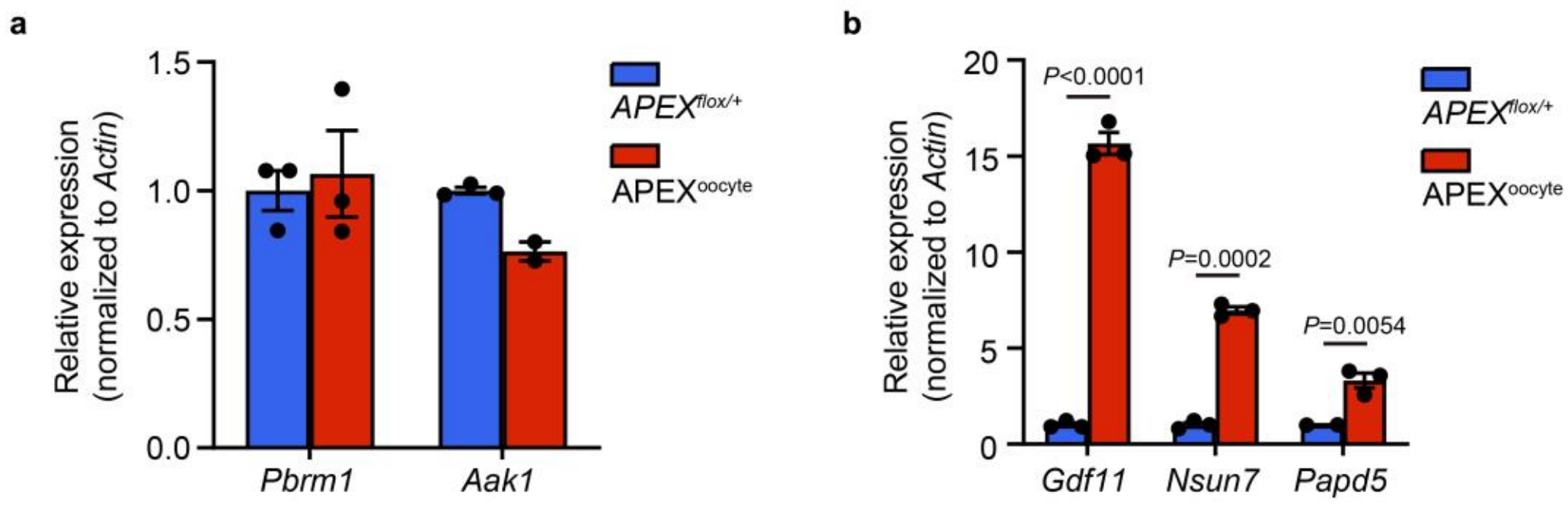

Figure R1. qRT-PCR validation of selected gene expression. a qRT-PCR analysis showing that transcripts of *Pbrm1* and *Aak1* are not significantly enriched in APEX^{oocyte} samples compared with *APEX*$^{flox/+}$ controls. n = 3 for each group. **b** In contrast, transcripts of representative oocyte-enriched genes (*Gdf11*, *Nsun7*, and *Papd5*) show robust and significant enrichment in APEX^{oocyte} samples. Mean ± SEM. n = 3 for each group. *P* values are calculated by unpaired two-tailed Student's t tests.

4. Missed opportunity for higher dose cisplatin labeling: Not performing APEX proteome labeling after a higher dose of cisplatin represents a missed opportunity. This higher dose induces DNA damage and significant primordial follicle depletion and may offer better targets for follicle protection. While the lower dose had no impact on oocyte morphology and DNA damage, the authors suggest that RNAseH2A protein abundance was increased due to RNA:DNA hybrids during DSB repair (lines 357-366). The authors also show an increase in the abundance of ATM kinase, which responds to DSBs. To address this, other markers (e.g., RAD51 or RPA) could be used to detect DSBs in oocytes after low-dose cisplatin. Additionally, identifying other DDR proteins in volcano plot in Figure 5K is recommended.

Response: We thank the reviewer for this insightful suggestion. In our study, we intentionally selected a lower cisplatin dose (2 mg/kg b.w.) because conventional assessments indicate that this regimen does not cause detectable harm to primordial follicles, whereas a higher dose (5 mg/kg b.w.) rapidly induces DNA damage, follicle apoptosis, and oocyte abnormalities. Under such high-dose conditions, the proteomic landscape would be dominated by proteins associated with DDR, apoptosis, and cell death, which could obscure other cisplatin-sensitive factors of primary interest. In addition, the primordial follicle pool is severely depleted at 5 mg/kg, leaving insufficient material for reliable biotinylation-based proteome analysis. By contrast, the lower dose preserves follicle numbers and ovarian morphology, thereby ensuring both the technical feasibility of APEX labeling and that the captured proteomic changes reflect the genuine oocyte response to cisplatin exposure. This design allowed us to identify the key molecular factors mediating the oocyte response without the confounding effects of extensive follicle loss.

To address the reviewer's point about DDR activation at the lower dose, we complemented our earlier observations (γH2AX, ATM, R-loops) with additional markers. Immunostaining confirmed the nuclear upregulation of RPA2, RAD51, and XRCC1 in PD7 oocytes following 2 mg/kg cisplatin exposure, as shown by significantly increased fluorescence intensity relative to controls **(Figs. 5m-o, text lines**

401–406 in revised manuscript; Also see below). And these DDR proteins were also annotated in the revised volcano plot **(Fig. 5k, text lines 396–397 in revised manuscript; Also see below)**. These results indicate that canonical DSB repair pathways are indeed engaged under sublethal treatment, even in the absence of overt histological changes.

Taken together, these data demonstrate that the selected dosing strategy is well-suited to capture oocyte stress–related proteomic signatures under conditions that preserve follicle integrity.

Figure 5

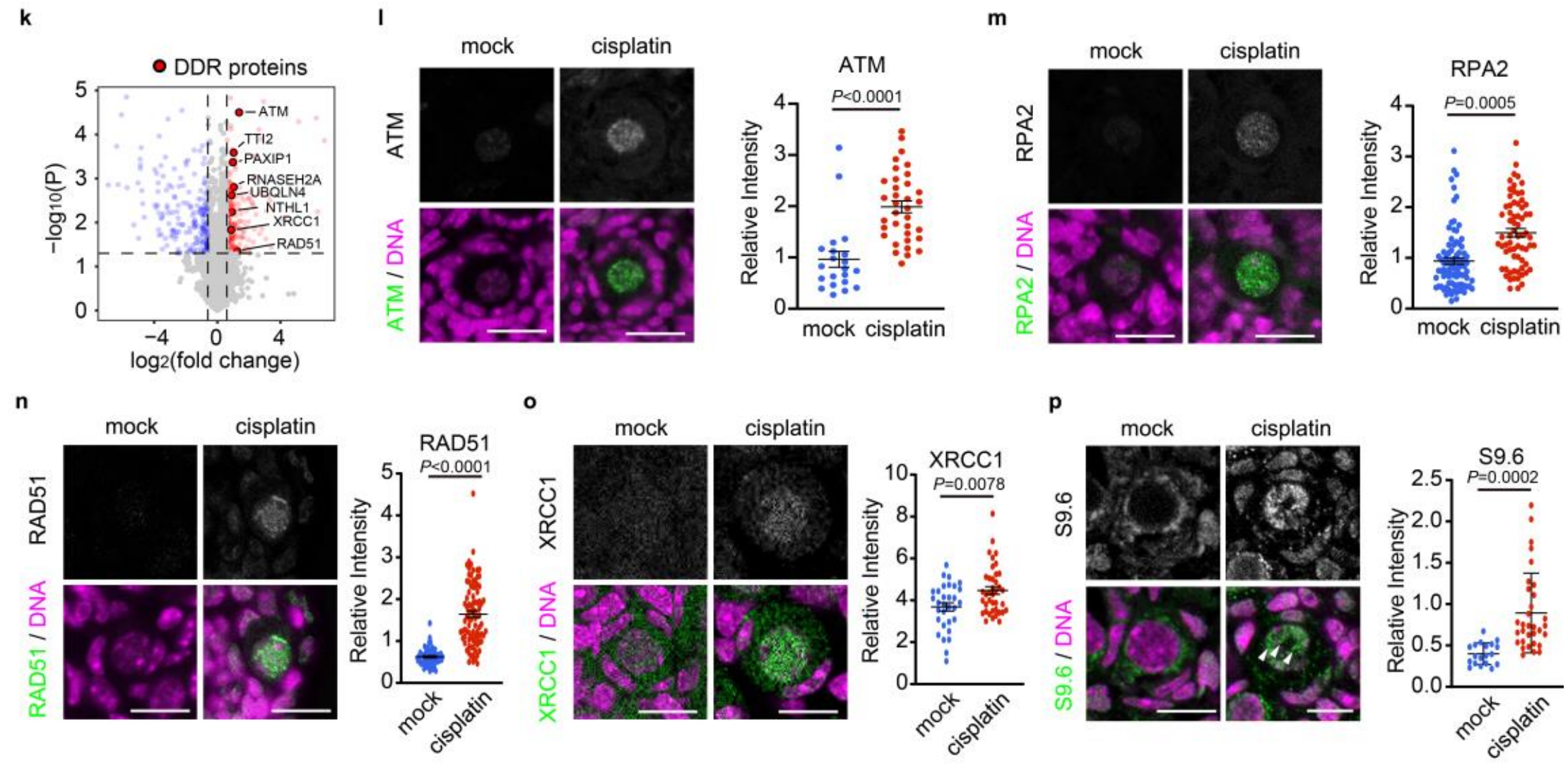

Figure 5. Cisplatin induces proteomic alterations in primordial follicle oocytes. k Volcano plot highlighting DNA damage response (DDR) proteins. **l** ATM immunostaining and quantification before and after cisplatin treatment from PD7 mice. Scale bar, 20 μm. Mean ± SEM. n = 21 and 35 for the mock and cisplatin groups, respectively. *P* values are calculated by unpaired two-tailed Student's t tests. **m** RPA2 immunostaining and quantification before and after cisplatin treatment. Scale bar, 20 μm. Mean ± SEM. n = 87 and 66 for the mock and cisplatin groups, respectively. *P* values are calculated by unpaired two-tailed Student's t tests. **n** RAD51 immunostaining and quantification before and after cisplatin treatment from PD7 mice. Scale bar, 20 μm. Mean ± SEM. n = 56 and 88 for the mock and cisplatin groups, respectively. *P* values are calculated by unpaired two-tailed Student's t tests. **o** XRCC1 immunostaining and quantification before and after cisplatin treatment from PD7 mice. Scale bar, 20 μm. Mean ± SEM. n = 31 and 38 for the mock and cisplatin groups, respectively. *P*

values are calculated by unpaired two-tailed Student's t tests. **p** S9.6 staining detecting RNA–DNA hybrids (R-loops) and quantification before and after cisplatin treatment from PD7 mice. White arrows indicate puncta signals in the treated oocytes. Scale bar, 20 µm. Mean ± SEM. n = 19 and 32 for the mock and cisplatin groups, respectively. *P* values are calculated by unpaired two-tailed Student's t tests.

5. EZH2 as a target for oocyte protection and fertility preservation: The authors identified EZH2 as a protein with changed abundance after cisplatin treatment in PD7 ovaries and a potential target for follicle protection. The authors then conclude in lines 375-376 that epigenetic abnormalities such as H3K27me3 related to changes in EZH2 expression may affect subsequent oocyte maturation and female fertility. This led authors to test EZH2 inhibition during cisplatin treatment and its effect on cisplatin-treated oocytes. However, it is unclear why authors used treated FGO rather than PD7 ovaries with primordial follicles to assess maturation defects. The manuscript lacks evidence that primordial follicles exposed to cisplatin at PD7 had maturation defects when they reached the FGO state. Treating FGO with EZH2 inhibitor and cisplatin seems unrelated to the rest of the manuscript. Furthermore, details on FGO treatment with cisplatin (dose, duration, etc.) are missing in methods. Authors should clarify the rationale for using FGO or consider treating PD7 ovaries with cisplatin (both low and high doses) and GSK126 to demonstrate a protective effect.

Response: We thank the reviewer for this thoughtful comment. We initially aimed to expose mice at PD7 and follow them to adulthood to directly assess the long-term consequences of cisplatin treatment on fertility. However, PD7 mice treated with cisplatin did not survive to adulthood, likely due to a combination of impaired maternal care for stressed litters and systemic toxicity at this early age. Thus, long-term follow-up of PD7-treated mice was not technically feasible. In addition, neonatal ovaries are extremely difficult to culture *ex vivo* for more than about one week, which precludes tracking primordial follicle development through to the FGO stage.

To address this limitation, we have performed complementary experiments. PD7 female mice were treated with cisplatin with or without the EZH2 inhibitor GSK126, and ovaries were collected at 1, 3, and 7 days post-treatment. Cisplatin induced a dynamic increase in H3K27me3 in primordial follicle oocytes, peaking at day 3. Co-treatment with GSK126 largely suppressed this accumulation, keeping levels close to control **(Figs. 6a-b; Also see below)**. Although H3K27me3 levels largely returned to baseline by day 7, irreversible follicle damage was observed, as apoptotic and necrotic primordial follicles were already present at day 1 and aberrant activation of primordial follicles was evident by day 3, suggesting premature depletion of the follicle pool. Notably, co-treatment with GSK126 alleviated these effects, reducing follicle apoptosis and aberrant activation **(Fig. 6c, Supplementary Fig. 7a; Also see below)**.

We further examined the functional consequences at the growing oocyte stage. PD14 mice were treated with cisplatin, with or without GSK126, and FGOs were collected seven days later. Nuclear maturation rates (SN/NSN ratio, GVBD) were comparable across groups, but cisplatin markedly impaired meiotic progression, as indicated by a reduced PBE rate. Importantly, co-treatment with GSK126 partially alleviated this defect, supporting a protective role for EZH2 inhibition during cisplatin exposure **(Fig. 6d, Supplementary Figs. 7b-c; Also see below)**.

Taken together, these data demonstrate that cisplatin induces both epigenetic dysregulation and apoptosis/over-activation of primordial follicles, and that these early insults subsequently compromise the maturation capacity of oocytes at later stages. Moreover, inhibition of EZH2 attenuates H3K27me3 accumulation, reduces follicle abnormalities, and partially rescues maturation defects, highlighting EZH2 as a potential target for fertility preservation. We have added these data to the **revised Figure (Figs. 6a-d, Supplementary Figs. 7a-c; also see below)** and updated **the corresponding text (lines 438–454)** in the Results section. In addition, detailed descriptions of cisplatin and GSK126 treatment for FGOs (including dose and duration) have now been incorporated into the Methods section **(lines 551–555 and 572–574)**.

Figure 6

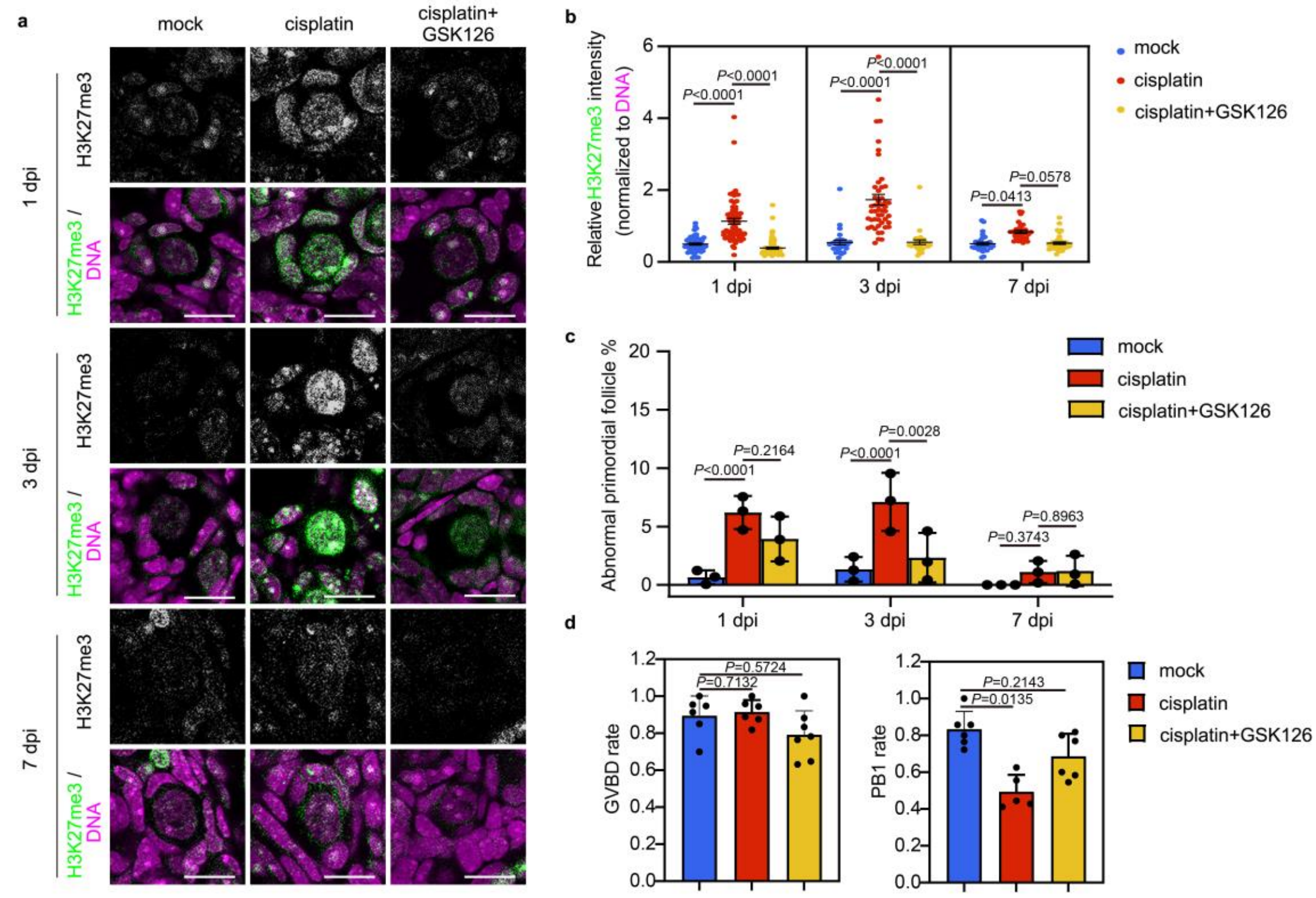

Figure 6. EZH2 inhibition mitigates cisplatin-induced oocyte defects. a Representative immunofluorescence images of H3K27me3 (green) and DNA (magenta) in primordial follicle oocytes at 1, 3, and 7 days post-injection (dpi) in mock, cisplatin, and cisplatin+GSK126 groups treated at PD7. Scale bar, 20 μm. **b** Quantification of H3K27me3 fluorescence intensity in **a** (normalized to DAPI). Mean ± SEM. 1 dpi (n = 49, 28, 33), 3 dpi (n = 61, 49, 34), 7 dpi (n = 79, 27, 37) for mock, cisplatin, and cisplatin + GSK126 groups, respectively. *P* values are calculated by unpaired two-tailed one-way ANOVA. **c** Percentage of morphologically abnormal primordial follicles at indicated time points. Mean ± SEM. n = 3 for each group. *P* values are calculated by unpaired two-tailed one-way ANOVA. **d** Meiotic maturation outcomes of FGOs collected 7 days after cisplatin treatment at PD14, with or without GSK126 co-treatment, followed by *in vitro* maturation analysis. Mean ± SEM. GVBD (n = 6, 6, 7) and PB1 (n = 6, 5, 6) for mock, cisplatin, and cisplatin + GSK126 groups, respectively. *P* values are calculated by unpaired two-tailed one-way ANOVA.

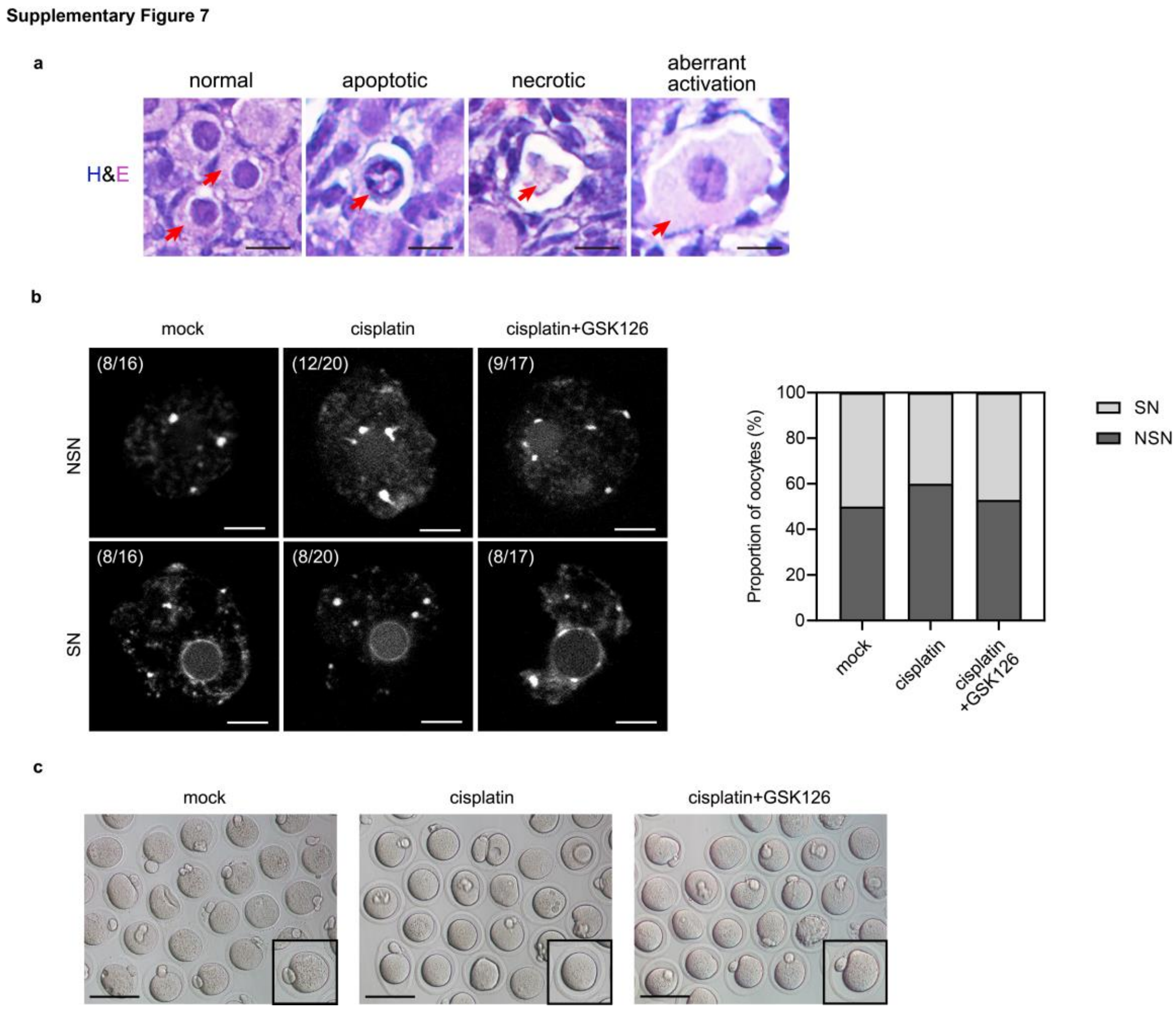

Supplementary Figure 7. Histological and meiotic phenotypes of oocytes after cisplatin exposure and rescue by EZH2 inhibition. a Representative hematoxylin and eosin (H&E) staining showing morphological features of normal, apoptotic, necrotic, and aberrantly activated primordial follicles (arrows indicate oocytes). Scale bars, 20 µm. **b** Representative FGOs showing non-surrounded nucleolus (NSN) and surrounded nucleolus (SN) chromatin configurations, collected from PD21 mice collected at 7 days after *in vivo* cisplatin treatment at PD14, with or without GSK126 co-treatment. Bar graph shows the proportion of SN and NSN oocytes. **c** Bright-field images of *in vitro*–matured oocytes under indicated treatments. Insets show representative oocytes. Scale bar, 100 µm.

6. H2O2 injection and oxidative stress: The system relies on H2O2 injection, which may induce oxidative stress in oocytes. The authors should address whether this

dose of H2O2 causes damage in the ovary and potentially confounds proteome studies. Additional experiments to assess oxidative damage would be valuable.

Response: We thank the reviewer for raising this important concern. We have carefully evaluated the potential impact of our labeling procedure, including H_2O_2 injection, on ovarian physiology, oocyte function, and the proteome.

Histological analysis of ovaries at PD7 and PD21 revealed normal gross morphology, with stage-based follicle counts and total follicle numbers comparable between BP+H_2O_2- and PBS-treated groups (all n.s., $P > 0.05$). Consistently, cleaved caspase-3 staining at PD21 showed similar numbers of apoptotic follicles across groups, with no $CC3^+$ cells detected at the primordial or primary stages. TUNEL assays at PD7 further confirmed no increase in apoptotic primordial follicles in the BP+H_2O_2 group relative to PBS (all n.s., $P > 0.05$). In addition, FGOs collected at P21 after *in vivo* labeling exhibited normal *in vitro* maturation, with GVBD and PBE rates as well as spindle morphology comparable to controls **(Supplementary Figs. 2a–i; Also see below)**. These results indicate that the labeling procedure did not measurably affect follicle/oocyte morphology or developmental competence.

To address the possible effects of H_2O_2 treatment on the ovarian proteome, we performed differential expression analysis of PD7 ovaries with or without H_2O_2 injection. Among the 6055 proteins detected, only 142 proteins were differentially expressed (45 upregulated and 97 downregulated), accounting for only ~2% of the total proteome. No oxidative stress–related pathways were significantly enriched among the dysregulated proteins, further supporting that the proteomic impact of H_2O_2 treatment was minimal **(Supplementary Figs. 2j–m; Also see below)**.

Taken together, evidence from histological analysis, apoptosis assays, oocyte maturation studies, and proteomic profiling consistently demonstrates that the H_2O_2 conditions applied in our APEX labeling system do not induce measurable oxidative stress or damage. Therefore, this procedure does not confound the reliability of our proteomic analysis. We have added these data to the **revised Figure (Supplementary Fig. 2; also see below)** and updated **the corresponding text (lines 182–192 and 200–207)** in the Results section.

Supplementary Figure 2

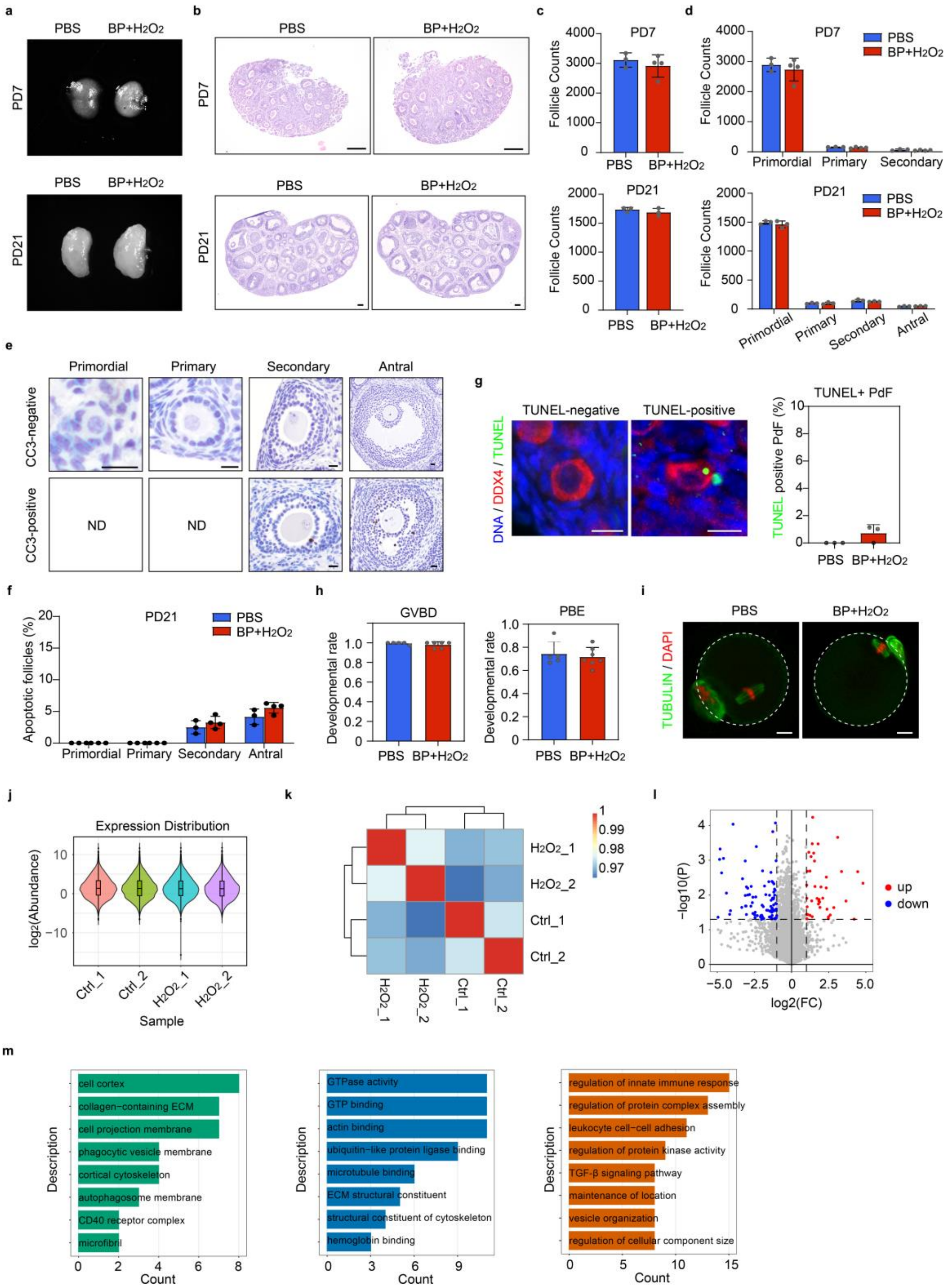

Supplementary Figure 2. The effects of APEX labeling reaction on ovarian physiological processes. a Gross morphology of ovaries collected at PD7 and PD21 from PBS- or BP+H_2O_2-treated mice. **b** H&E-stained ovarian sections showing

comparable follicular architecture between groups at PD7 and PD21. Scale bars, 100 μm. **c–d** Quantification of total follicle numbers (**c**) and stage-specific follicle counts (**d**) at PD7 (top) and PD21 (bottom). Mean ± SEM. PD7 (n = 3, 4) and PD21 (n = 3, 3) for PBS and BP + H_2O_2, respectively. **e** Representative images of cleaved caspase-3 (CC3) staining in follicles at different stages at PD21. "ND", not detected. Scale bars, 20 μm. **f** Quantification of apoptotic follicles detected by CC3 staining at PD21. Mean ± SEM. PD21 (n = 3, 4) for PBS and BP + H_2O_2, respectively. **g** TUNEL staining of primordial follicles at PD7 showing representative TUNEL-negative and TUNEL-positive examples (left) and statistical comparison of $TUNEL^+$ primordial follicles per ovary (right). Scale bars, 20 μm. Mean ± SEM. n = 3 for each group. **h** *In vitro* maturation of FGOs collected at PD21 following *in vivo* labeling. Mean ± SEM. GVBD (n = 5, 6) and PBE (n = 5, 7) for PBS and BP + H_2O_2, respectively. **i** Representative spindle morphology of *in vitro* matured oocytes, visualized by α-tubulin (green) and DNA staining (red), demonstrating intact meiotic spindle formation in both groups. Scale bars, 20 μm. **j** Distribution of all ovarian proteins expressed in control (Ctrl) and H_2O_2-treated mice, with log2-transformed abundance across biological replicates. Center line, median; box limits, upper and lower quartiles; whiskers, 1.5× interquartile range; points, outliers. **k** Correlation heatmap showing the expression similarity between Ctrl and H_2O_2 treated groups based on total protein expression data. **l** Volcano plot of differentially expressed genes comparing H_2O_2 treatment and control groups. Red dots represent upregulated genes, blue dots represent downregulated genes. **m** Gene ontology (GO) term enrichment analysis of differentially expressed genes. Cellular components (green), molecular functions (blue), and biological processes (orange) significantly enriched in treated vs control groups.

7. Novelty of in-situ imaging and follicle quantification: While the authors emphasize the novelty of their system for in-situ imaging of oocytes and follicle quantification, it's essential to acknowledge existing studies. Previous research has demonstrated 3D ovarian structure and performed follicle quantification using

immunostaining and clearing methods (e.g., PMID: 31965156, PMID: 34542534, PMID: 38798456, PMID: 37464244). Although this method is antibody-free and quicker, its applicability to complex genetic models (e.g., conditional knockouts) should be considered.

Response: We thank the reviewer for highlighting these important prior studies and fully agree that several groups have elegantly demonstrated 3D ovarian architecture and follicle quantification using immunostaining combined with tissue clearing (PMID: 31965156, 34542534, 38798456, 37464244). We have now cited and discussed these works in the revised manuscript **(lines 503–508)**.

In our study, the APEX mouse model was primarily designed for *in vivo* protein labeling and identification in specific cell types. The 3D visualization derived from EGFP fluorescence is therefore not the main focus but rather an additional feature, enabling the spatial distribution of the targeted cell type to be observed within intact tissues and organs. To support this, we established a 3D *in situ* imaging workflow tailored for APEX-EGFP labeling, rather than replicating existing immunostaining/clearing approaches. This workflow provides spatial localization of the targeted cells as an anatomical reference, complementing the proteomic and transcriptomic data obtained from the same system.

Compared with previous works, our system offers a rapid, antibody-free in-situ imaging workflow that substantially reduces processing time and avoids antibody penetration issues. It can also be readily extended to diverse Cre-driver lines, including conditional knockouts, without requiring additional antibody optimization. At the same time, we recognize that the approach depends on genetically engineered mouse models, which is an inherent requirement of the APEX system and may be viewed as a technical limitation relative to antibody-based approaches. The revised manuscript now provides a balanced description of both the novelty of our workflow and the practical considerations associated with its application **(text lines 503–518 in revised manuscript)**.

8. Follicle stage identification and immunostaining examples: Authors incorrectly identified follicle stages and used incorrect stages as examples in immunostaining figures. This should be corrected, and authors are encouraged to consult with follicle development specialists if they lack expertise in this area.

Response: We thank the reviewer for pointing out this issue. All ovarian sections and follicle stage classifications were carefully re-examined, with misclassified stages in the original figures corrected, and quantification analyses also re-checked. We apologize for the oversight and believe these corrections enhance the accuracy of our presentation.

Reviewer #3:

Manuscript background information:

In this manuscript by Jiang, et al, the authors develop a proteomic profile of oocytes in primordial follicles using a novel ascorbate peroxidase (APEX)-based protein labeling mouse model. Using this model, the authors are able to visualize the ovary in 3D, capture and identify proteins specific to oocytes, and capture and sequence RNA from oocytes. This model and findings can be applied in future research to improve tools for determining reproductive capability in females, particularly those undergoing chemotherapy treatments. Cisplatin, a common cancer treatment, is known to negatively affect female fertility. More specifically, cisplatin induces apoptosis in oocytes and mitochondrial damage to immature oocytes. Therefore, it is worthwhile to investigate proteomic shifts in oocytes from primordial follicles after cisplatin treatment. Comparing APEX-labeled ovaries from mice treated with cisplatin with control mice, they found there were rapid changes in proteins involved in DNA damage repair and histone modification. With these findings, the authors were able to protect oocytes from the effects of cisplatin using a EZH2 inhibitor, GSK126. This study validates the first in vivo APEX-based proteins labeling model and characterizes the

proteome of oocytes from primordial follicles both in normal development and when perturbed by cisplatin exposure.

Major comments/limitations:

1. The authors claim that by performing their captures in PD7 ovaries, they are profiling only primordial follicles, as they are the dominant population in postnatal day 7 ovaries. However, they do not seem to account for the population of growing oocytes present in the medullary region of the ovary at this stage. While it is true that this population is much smaller (as the authors show in figure 1), it is possible that their proteome could be over-represented in the dataset, as these are much bigger and active oocytes. This is also suggested by the fact that many of the proteins the authors identify are clearly found in the oocyte throughout follicle growth, as illustrated by the IHC experiments in figures 3 and 4. One way the authors could address this would be to perform APEX profiling using a growing-oocyte specific cre line, such as Zp3-Cre (Lewandowski et al, 1997) and cross-reference this profile to the present one to identify primordial-specific proteins. I recognize this is a big experiment, so if the authors are not in a position to perform this, I suggest softening the conclusions and language throughout the text to clarify that this is not a primordial-specific profile.

Response: We thank the reviewer for this insightful comment. We acknowledge that PD7 ovaries, while predominantly containing primordial follicles, also include a small population of growing primary follicles, which may contribute to some of the proteins detected in our analysis. We agree with the reviewer that using a growing-oocyte specific Cre line such as Zp3-Cre could help to partially exclude proteins highly expressed in growing oocytes, but implementing such an experiment would be highly time- and labor-intensive. As an alternative, we focused on optimizing our experimental design to minimize the contribution of growing oocytes to the proteomic dataset. Specifically, we shortened the BP incubation time to 1 hour to limit substrate diffusion only into the ovarian cortex, thereby minimizing unintended labeling in growing follicles and ensuring that the proteomic signals predominantly reflect primordial

oocytes. Additionally, we increased the biological input to 12 ovaries per replicate (up from 5), optimized the enrichment procedure with stringent wash steps (1 M KCl, 0.1 M Na_2CO_3, and 2 M urea, each performed twice) to reduce nonspecific binding, and used an updated mass spectrometer, improving peptide coverage and quantitative accuracy.

In the revised dataset, a total of 2,772 proteins were designated as a high-confidence oocyte proteome associated with primordial and early-stage ovarian follicles (fold change > 1.5, $P < 0.05$) **(Supplementary Data 1, Fig. 2e; Also see below)**. Functional enrichment analyses demonstrated that these proteins are strongly aligned with key biological features of early oocytes, including chromatin regulation, RNA metabolism, kinase-mediated regulation, and protein stability control **(Figs. 2h-i; Also see below)**. To further reduce potential confounding from labeling of growing follicles, we specifically focused on proteins that had not been detected in previously published proteomic datasets of fully grown oocytes. Multiple well-recognized primordial follicle and early oocyte–associated proteins were identified, and representative proteins from major functional categories were further examined by streptavidin pull-down followed by Western blotting and immunohistochemical analyses. Among these, chromatin regulatory proteins associated with histone H3 lysine 14 acetylation (H3K14ac), including polybromo 1 (PBRM1) and bromodomain containing 1 (BRD1), showed relatively higher abundance in primordial follicle oocytes, followed by a decline at later follicular stages, consistent with the elevated H3K14ac levels observed in primordial oocytes. In parallel, representative proteins from other enriched categories, including the kinases TANK-binding kinase 1 (TBK1) and AP2-associated kinase 1 (AAK1), as well as protein homeostasis regulators such as BAG cochaperone 6 (BAG6) and macrophage erythroblast attacher (MAEA), were detectable in oocytes across folliculogenesis, with enrichment evident at early follicular stages **(Fig. 3, Supplementary Figs. 3d-e, Supplementary Fig. 4; Also see below)**. Together, these analyses provide strong support for the reliability and biological relevance of the revised proteomic dataset.

Taken together, the revised dataset provides a more accurate and refined protein list enriched for oocytes from early-stage follicles, with predominant contribution from primordial follicles. Together with experimental validation, these data offer new insights into early oocyte molecular organization. We also have softening the language throughout the text to ensure more accurate and cautious conclusions. We have included these updates in the revised manuscript **(text lines 207–302 in the Results section)**.

Figure 2

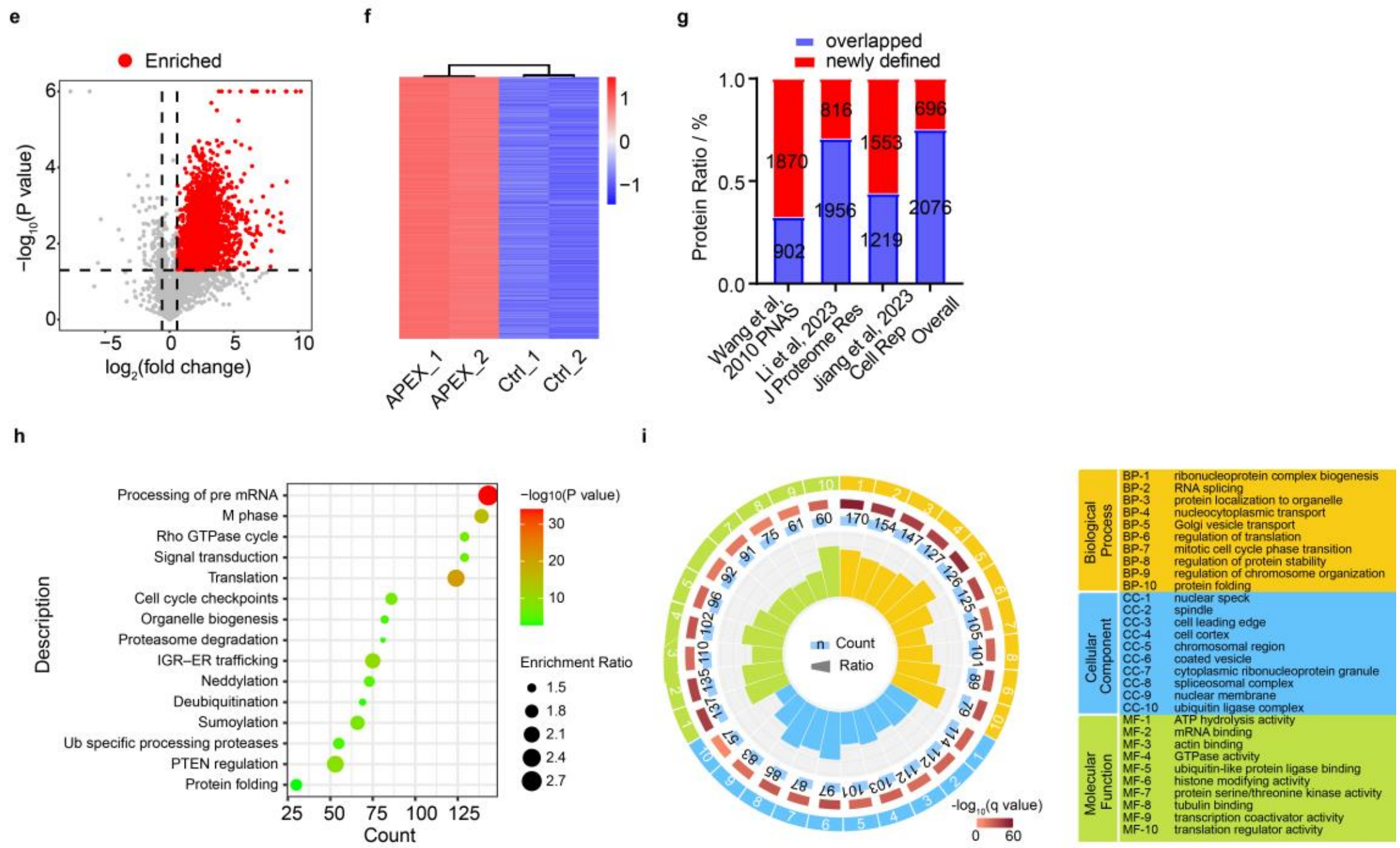

Figure 2. *In situ* APEX-based proteomic profiling of oocytes from early-stage ovarian follicles. e Volcano plot of proteins detected by LC–MS/MS comparing APEX^{oocyte} and *APEX$^{flox/+}$* control samples. Significantly enriched proteins are highlighted (red dots, fold change > 1.5, $P < 0.05$). **f** Heatmap of enriched proteins across biological replicates of APEX^{oocyte} and *APEX$^{flox/+}$* (Ctrl) samples. **g** Overlap analysis of the identified proteome with previously published GV-stage oocyte proteomes. Stacked bars indicate overlapped and newly defined proteins. **h** Bubble plot of enriched Reactome pathways. The x-axis shows the gene number (Count), the y-axis lists pathway names, bubble size reflects enrichment ratio, and color indicates significance (–$\log_{10}$ [*P*-value]). **i** Circular plot of the top 10 enriched gene ontology (GO) terms in biological process (yellow), cellular component (blue), and molecular function (green). From inside to outside: enrichment factor (Ratio), gene number

(Count), statistical significance (–$\log_{10}$ [q-value]), and GO term IDs corresponding to the descriptions on the right.

Supplementary Figure 3

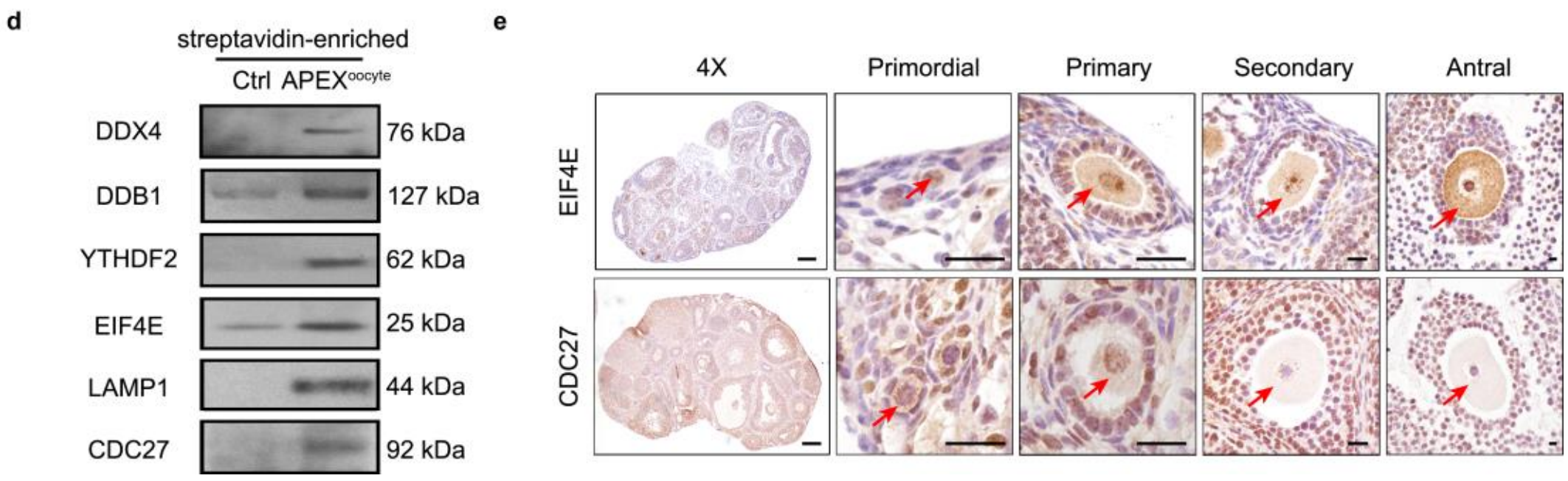

Supplementary Figure 3. Quality assessment and validation of the streptavidin-enriched oocyte proteome. d Western blot analysis of representative proteins in streptavidin-enriched fractions from control (Ctrl) and $APEX^{oocyte}$ ovaries. **e** Immunohistochemical staining of eukaryotic translation initiation factor 4E (EIF4E) and cell division cycle 27 (CDC27) in PD21 ovarian sections. Images include low-magnification overview and higher-magnification views of primordial, primary, secondary, and antral follicles. Arrows indicate oocytes. Scale bars, 100 µm in 4× microscopy and 20 µm in enlarged views.

Figure 3

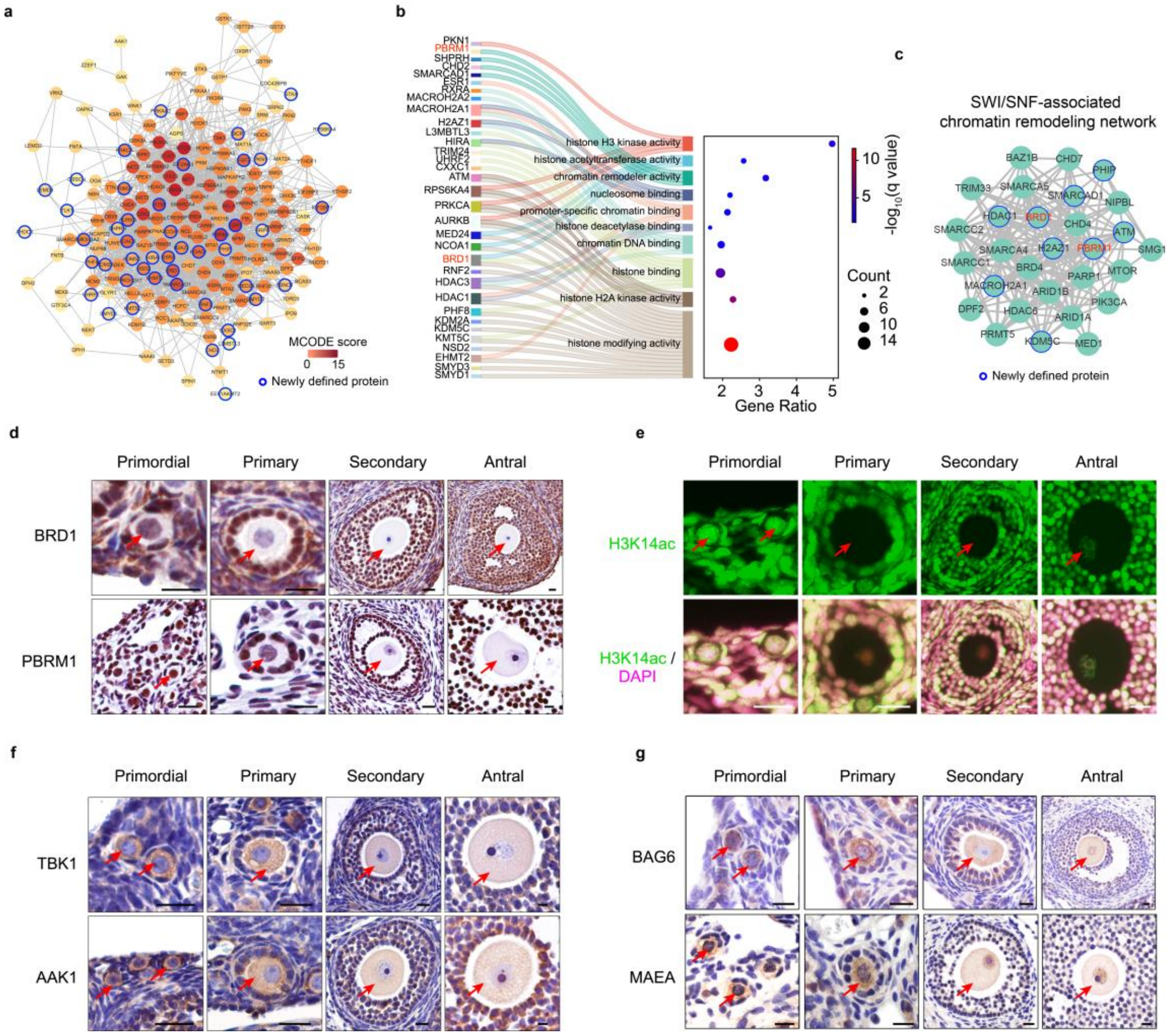

Figure 3. Expression dynamics of representative regulatory proteins in oocytes. a Protein–protein interaction network of chromatin regulation–associated proteins identified in the oocyte proteome. Nodes represent proteins, with newly defined proteins indicated by blue-bordered circles. Red nodes represent proteins with higher MCODE scores, indicating highly interconnected proteins within the network. **b** Sankey plot showing the relationship between newly defined proteins (left) and functional categories (right). The size and color of the bubbles on the right correspond to the gene number (Count) and –log(P-value), respectively, representing the enrichment level of each functional category. **c** Subnetwork of SWI/SNF-associated chromatin remodeling proteins. Newly defined proteins are indicated by blue-bordered circles. **d** Immunohistochemical staining of bromodomain containing 1 (BRD1) and polybromo 1 (PBRM1) in ovarian sections across follicle stages (primordial, primary, secondary, and antral). Arrows indicate oocytes. Scale bars, 20 µm. **e** Immunofluorescence staining of histone H3 lysine 14 acetylation (H3K14ac) (green) in ovarian sections across follicle stages. Merged images with DAPI (magenta) are

shown. Arrows indicate oocytes. Scale bars, 20 μm. **f** Immunohistochemical staining of TANK binding kinase 1 (TBK1) and AP2 associated kinase 1 (AAK1) in ovarian sections across follicle stages. Arrows indicate oocytes. Scale bars, 20 μm. **g** Immunohistochemical staining of BAG cochaperone 6 (BAG6) and macrophage erythroblast attacher (MAEA) in ovarian sections across follicle stages. Arrows indicate oocytes. Scale bars, 20 μm.

Supplementary Figure 4

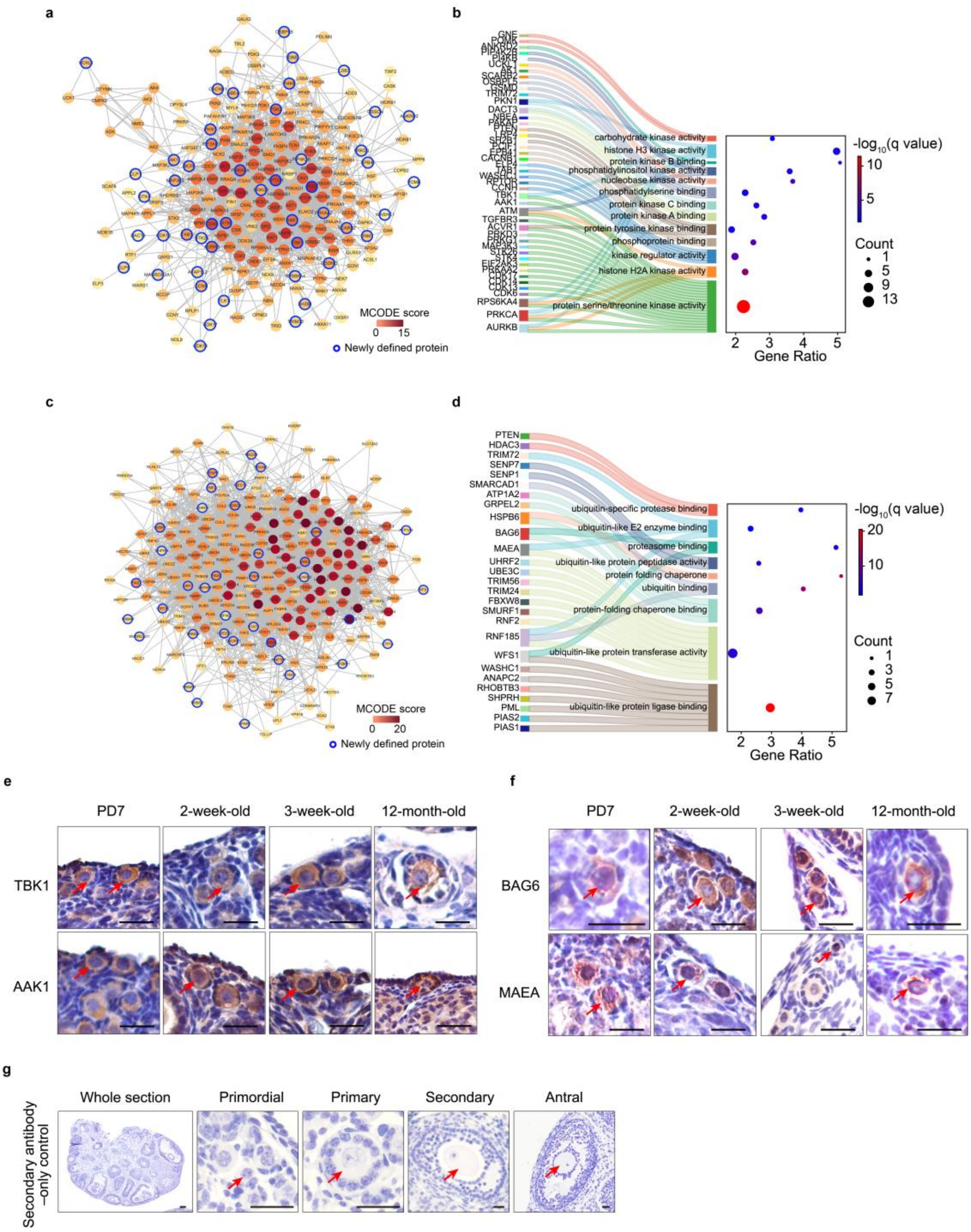

Supplementary Figure 4. Interaction networks and age-independent expression of regulatory proteins in oocytes. a Protein–protein interaction network of kinase-related regulatory proteins identified in the oocyte proteome. Nodes represent proteins, with newly defined proteins indicated by blue-bordered circles. Node color intensity reflects MCODE score. **b** Functional enrichment analysis of proteins in the kinase-associated network. The Sankey diagram links newly defined proteins (left) to enriched molecular function categories (right). In the bubble plot, bubble size represents protein count and color indicates $-\log_{10}$ (q value). **c** Protein–protein interaction network of proteins associated with protein stability and ubiquitin–proteasome regulation. Newly defined proteins are indicated by blue-bordered circles. Node color intensity reflects MCODE score. **d** Functional enrichment analysis of proteins in the protein homeostasis network. The Sankey diagram links newly defined proteins (left) to enriched molecular function categories (right). In the bubble plot, bubble size represents protein count and color indicates $-\log_{10}$ (q value). **e** Immunohistochemical staining of TANK binding kinase 1 (TBK1) and AP2 associated kinase 1 (AAK1) in ovarian sections from PD7, 2-week-old, 3-week-old, and 12-month-old mice. Arrows indicate primordial follicle oocytes. Scale bars, 20 μm. **f** Immunohistochemical staining of BAG cochaperone 6 (BAG6) and macrophage erythroblast attacher (MAEA) in ovarian sections from PD7, 2-week-old, 3-week-old, and 12-month-old mice. Arrows indicate primordial follicle oocytes. Scale bars, 20 μm. **g** Secondary antibody–only control staining across follicle stages in PD21 ovarian sections. Arrows indicate oocytes. Scale bars, 100 μm in 4× microscopy and 20 μm in enlarged views.

2. Authors should include more details about the methods across the manuscript. As they often use multiple ages of mice in this study, including that information is crucial. Additionally, in images of oocytes/follicles, it is not clear if these are isolated oocytes or oocytes within the whole ovary. If these are isolated oocytes, their method of extraction should be clear.

Response: We thank the reviewer for this helpful suggestion. In the revised manuscript,

we have clarified the ages of mice used in each experiment in both the figure legends and the Methods section. The legends now also specify whether the images represent ovarian tissue sections or isolated oocytes. The procedures for oocyte isolation are described in detail in the Methods **(text lines 557–577 in the Results section)**. With these revisions, the developmental stages of the animals and the origin of the imaged oocytes are clearly defined, ensuring that the experimental workflow is transparent and reproducible.

3. More detail is needed in the Methods section, especially pertaining to the methods of IHC and IF image analysis. How is labeling intensity being measured? Adding an image of tissue treated only with secondary antibodies would be useful for convincing the reader that the signal they see is specific staining.

Response: We thank the reviewer for this valuable suggestion. In the revised manuscript, we have added detailed descriptions of the immunohistochemistry (IHC) and immunofluorescence (IF) image analysis procedures in the Methods section **(text lines 618–621 in the Results section)**. Labeling intensity was quantified using ImageJ by measuring the mean gray value within the region of interest (ROI) after background subtraction. BUB3 immunofluorescence intensity was quantified and normalized to CREST signals to account for variations in staining and imaging conditions. To confirm staining specificity, ovarian sections were incubated with secondary antibodies only, and the resulting control images showed no detectable signal **(Supplementary Fig. 4g, text lines 268–270 in the revised manuscript; also see below)**. These additions not only clarify the image quantification procedure but also reinforce the reliability and specificity of the IHC and IF results presented in the study.

Supplementary Figure 4

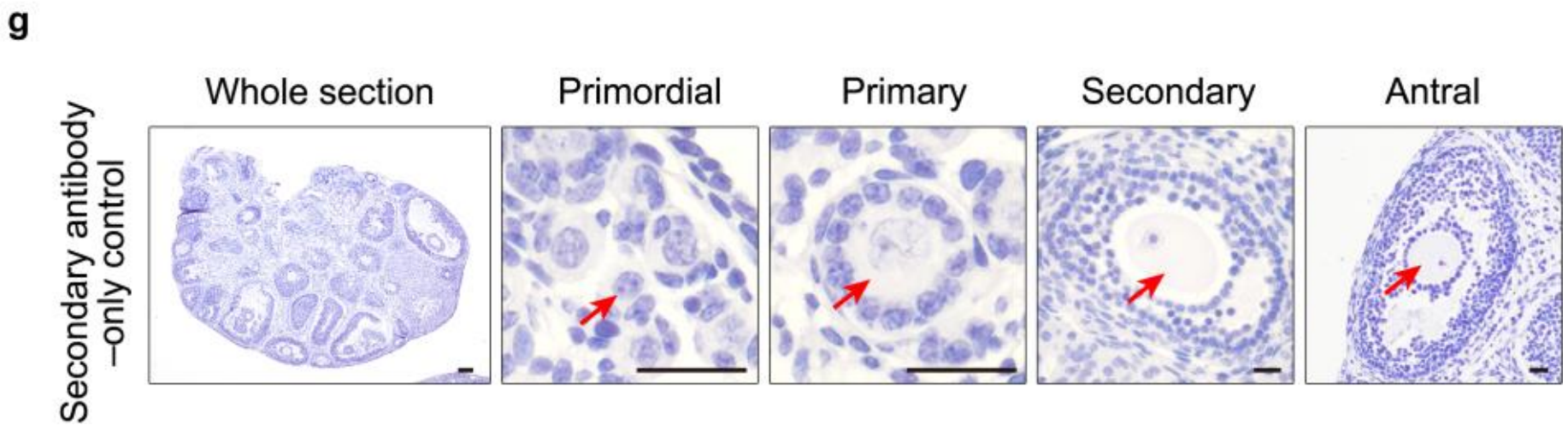

Supplementary figure 4. Interaction networks and age-independent expression of regulatory proteins in oocytes. g Secondary antibody–only control staining across follicle stages in PD21 ovarian sections. Arrows indicate oocytes. Scale bars, 100 μm in 4× microscopy and 20 μm in enlarged views.

4. The authors state that this system is advantageous because they are able to characterize the in situ proteome. I'm having trouble understanding how this model is in situ. I think clarifying this in the introduction would be beneficial.

Response: We thank the reviewer for this valuable comment. In our study, the term *in situ* refers to protein labeling performed within the intact, living tissue microenvironment, before any tissue disruption occurs. This approach is distinct from traditional proteomics, which often relies on isolating specific cell types from complex tissues through lengthy processes such as digestion, labeling, and sorting. Aside from being inefficient, these methods may introduce artificial effects, including loss of the cellular context and disruption of protein interactions, which are essential for understanding the proteins' roles in their native environment. In contrast, our method allows the covalent tagging of proteins within their original spatial and molecular context. Specifically, the APEX2 enzyme is genetically expressed in the target cell type (e.g., oocytes), and upon triggering the reaction, it covalently tags proximal proteins within their natural tissue environment. Following tissue collection and cell lysis, the labeled proteins are enriched and identified, providing a more accurate representation of the proteins as they exist within the tissue microenvironment. We have now clarified this conceptual and methodological framework in the Introduction **(text lines 73–87 in**

the revised manuscript), highlighting the advantages of our *in situ* approach over traditional isolation-based methods.

5. There are already many established protocols to visualize the entire perinatal ovary. I recommend adding citations to this previous body of work to credit them for their contributions. For example, we recommend citing Soygur et al. Sci. Adv. (2021). DOI:10.1126/sciadv.abc6747, Boateng et al. J. Vis. Exp. (2021).doi:10.3791/62972, Faire et al. Developmental Biology.(2015). https://doi.org/10.1016/j.ydbio.2015.04.006., McKey et al. eLife.(2022). https://doi.org/10.7554/eLife.81088, Folts et al. bioRxiv.(2024).doi: https://doi.org/10.1101/2024.05.13.593993, McKey et al. PNAS. (2019). https://doi.org/10.1073/pnas.1814930116, Feng et al. Sci Rep (2017). https://doi.org/10.1038/srep44810.

Response: We thank the reviewer for highlighting the importance of acknowledging prior contributions to the field. We agree that several studies have established protocols for visualizing the entire perinatal ovary. We have now cited and discussed these works in the revised manuscript **(lines 503–508)**. Compared with previous works, our system offers a rapid, antibody-free in-situ imaging workflow that substantially reduces processing time and avoids antibody penetration issues. It can also be readily extended to diverse Cre-driver lines, including conditional knockouts, without requiring additional antibody optimization. At the same time, we recognize that the approach depends on genetically engineered mouse models, which is an inherent requirement of the APEX system and may be viewed as a technical limitation relative to antibody-based approaches. The revised manuscript now provides a balanced description of both the novelty of our workflow and the practical considerations associated with its application **(text lines 503–518 in revised manuscript)**.

6. The APEX-labeling system to visualize the whole ovary is useful for their proteomic approach but seems impractical as a platform to image the whole perinatal ovary. Consider moving Figure 1 to supplemental data as validation that the APEX-labeling system does target oocytes efficiently. If the authors compared their findings from Figure 1 to previously described 3D ovary imaging techniques, that would make it more significant.

Response: We thank the reviewer for this suggestion. We agree that the primary purpose of the APEX system in our study is *in vivo* protein labeling for proteomic analysis, and that the 3D ovarian visualization serves as a complementary feature rather than the main application. Our 3D imaging workflow visualizes oocytes through EGFP fluorescence within the ovary, requiring subsequent tissue clearing and nuclear staining *ex vivo*, rather than enabling direct *in vivo* imaging of the entire ovary or all ovarian cell types. Although not designed as a standalone platform for whole perinatal ovary imaging, this workflow still offers practical advantages over antibody-based 3D imaging methods, such as eliminating lengthy antibody penetration steps, reducing processing time, and achieving intrinsic specificity for genetically defined oocyte populations across developmental stages without the need for marker optimization. Given these benefits and the fact that Figure 1 provides essential validation that the $APEX^{oocyte}$ system efficiently targets oocytes and enables quantitative follicle reconstruction, we have retained this figure in the main manuscript. At the same time, we have expanded the discussion by comparing our approach and results with previously reported 3D ovarian imaging techniques to better clarify the scope, strengths, and applicability of our method in relation to previous studies **(text lines 503–518 in revised manuscript)**.

7. The authors do not describe the mouse lines they used for their experiments (Line 487 - 494) How was the APEX line generated, and which Gdf9-cre line was used? How was it obtained? This is absolutely critical information for reproducibility. The authors should also confirm that Gdf9-Cre targets all oocytes,

including primordial follicles, since the GDF9 protein is typically only expressed in growing follicles. If they do confirm expression of the transgene in primordial oocytes, they should clearly address this difference in expression pattern.

Response: We thank the reviewer for this important comment. The *APEX*$^{flox/flox}$ mice, which enable conditional expression of APEX2–EGFP following Cre recombination, were generated and are maintained in our laboratory. To generate the Cre-dependent APEX2-EGFP mouse, we performed CRISPR/Cas9-assisted gene targeting in zygotes. Briefly, pronuclear-stage zygotes were obtained by mating C57BL/6 J males with superovulated C57BL/6 J females. A mixture containing Cas9 mRNA, sgRNA, and the targeting vector was microinjected into the male pronucleus using a microinjection system. The targeting vector inserted the APEX2 transgene cassette into the *Rosa26* locus, driven by a CAG promoter and flanked by loxP sites. After genotyping and confirming positive transmission, heterozygous mice were intercrossed to generate homozygous LSL-APEX2-EGFP$^{f/f}$ mice. These mice were then crossed with *Gdf9*-Cre transgenic mice to generate *Gdf9*-Cre; *APEX*$^{flox/+}$ offspring (referred to as APEX^{oocyte} mice), in which APEX2 is selectively expressed in oocytes, including those at the primordial follicle stage. These details have been added to the Methods section **(text lines 543–548 in revised manuscript)**.

The *Gdf9*-Cre mouse line used in this study was derived from Austin J. Cooney's lab, as previously described [4] **(text lines 106–110 in revised manuscript)**. In this model, the *Gdf9* promoter drives Cre recombinase expression specifically in oocytes, including those in primordial follicles as well as in more mature follicles within the ovary. In the study "Differential oocyte-specific expression of Cre recombinase activity in *GDF-9-iCre*, *Zp3cre*, and *Msx2Cre* transgenic mice" [4], the authors demonstrated that *Gdf9*-Cre begins expressing in primordial oocytes around postnatal days 3-5. This Cre line has been used in our previous studies to generate several conditional knockout mouse models, including those with deficiencies in oocytes at the primordial follicle stage for genes such as *Ddb1* [5], *Yap1* [6], *Erk1/2* [7], *Dcaf13* [8], and *Pabpn1* [9].

To confirm that APEX expression labels all oocytes, we have first performed co-immunostaining with GFP and DDX4, a well-established germ cell marker, to visualize oocytes. DDX4 and GFP co-localized in nearly all oocytes, confirming that APEX labeling specifically targets oocytes **(Fig. 1e, text lines 132–135 in revised manuscript; Also see below)**. Subsequently, to assess APEX labeling, we have also performed co-immunostaining with Streptavidin and DDX4 on histological sections of ovaries after the APEX reaction, at both PD7 and PD21. Substantial overlap of Streptavidin and DDX4 signals was observed, indicating that APEX expression, driven by *Gdf9*-Cre, labels oocytes, including those in primordial follicles. Notably, Streptavidin-positive signals were also detected in quiescent primordial follicles at PD21, suggesting that while *Gdf9*-Cre activity is weaker in these oocytes, it remains sufficient for APEX-mediated labeling **(Fig. 2c, text lines 173–182 in revised manuscript; Also see below)**.

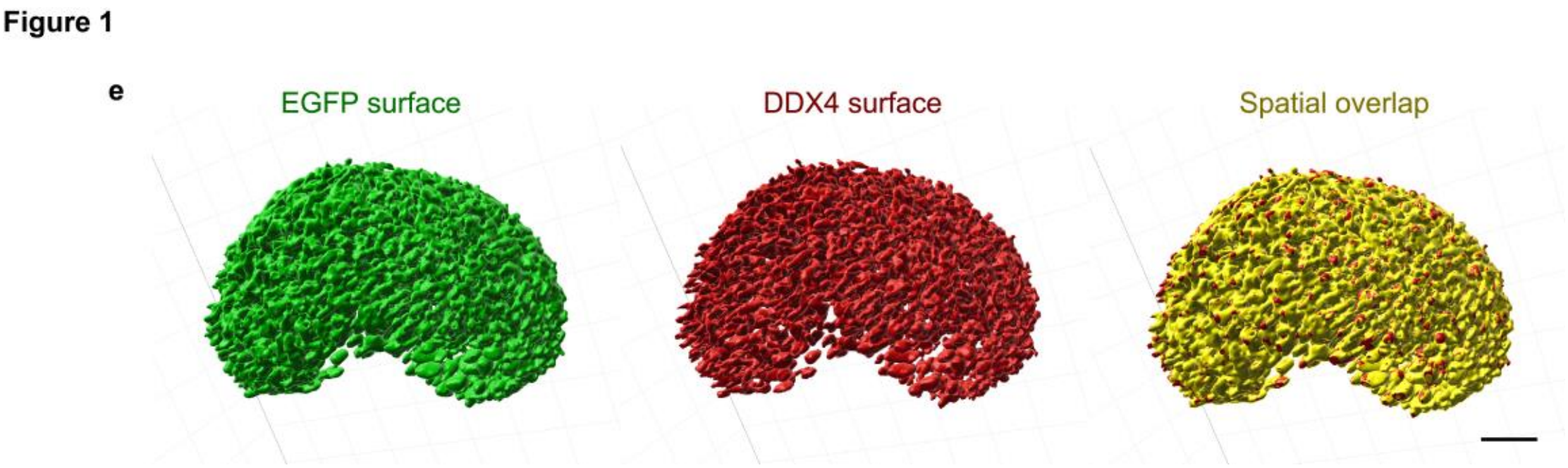

Figure 1. Targeting active APEX2 to *in vivo* follicle-enclosed oocytes. e Surface rendering of EGFP signal and DDX4 immunostaining in a PD7 ovary. EGFP-positive oocytes are shown in green, DDX4-positive germ cells in red, and their spatial overlap in yellow. Scale bars, 100 μm.

Figure 2

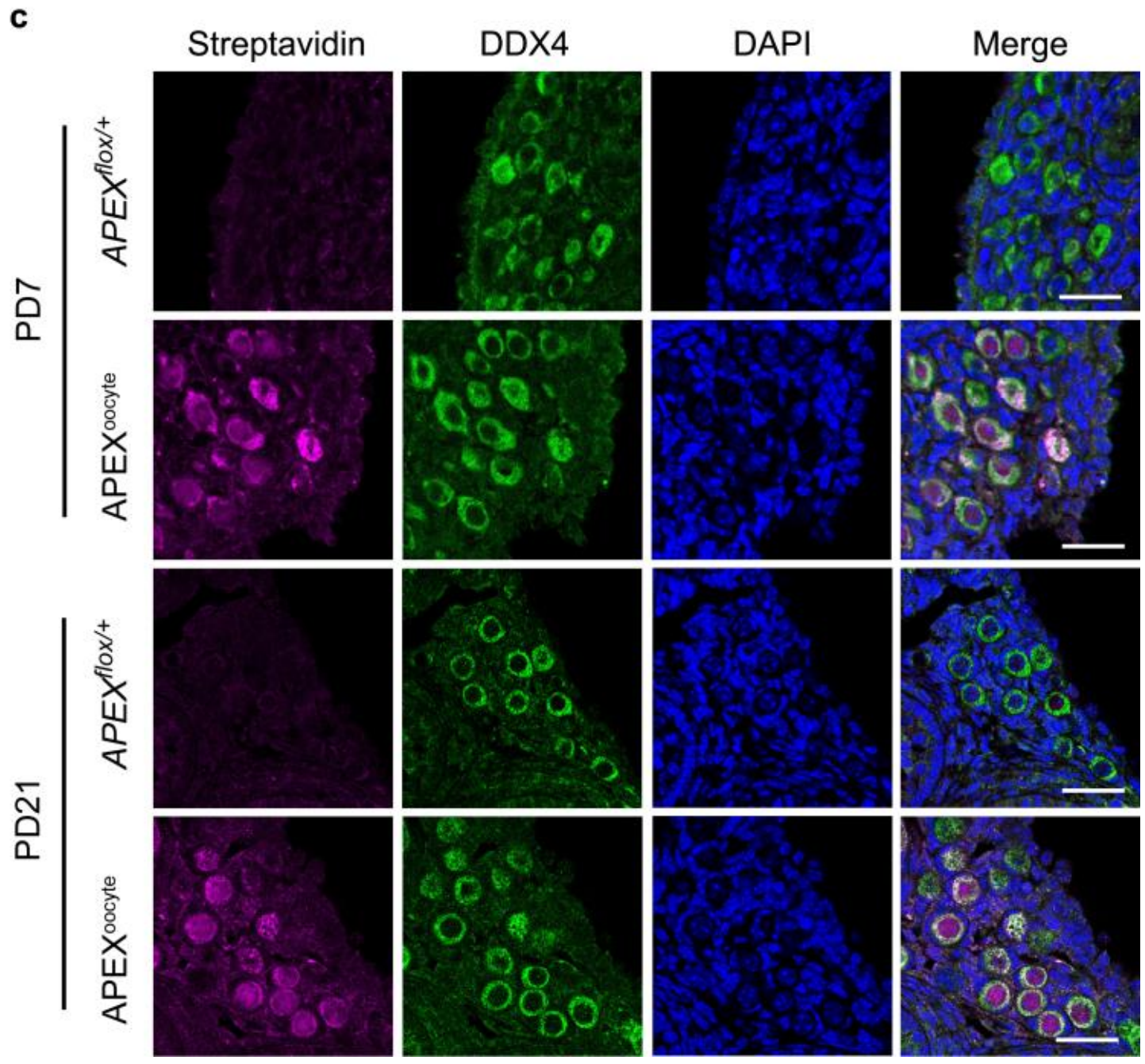

Figure 2. ***In situ*** **APEX-based proteomic profiling of oocytes from early-stage ovarian follicles. c** Streptavidin labeling in ovarian sections at PD7 and PD21 in APEX^{oocyte} and *APEX*$^{flox/+}$ mice, respectively. Co-staining for streptavidin (magenta), DDX4 (green), and DAPI (blue) is shown. Scale bars, 50 μm.

8. In the explanation of Figure 1, provide more detail on image analysis and how you account for oocyte segmentation and oocytes that were miss-segmented. We also recommend adding an overlay in Figures 1E and 1H with the corresponding IF image to illustrate the segmentation efficiency.

Response: We thank the reviewer for this constructive comment. The 3D modeling and quantification presented in Figure 1 were performed using Imaris (version 10.1.1). For total follicle counting, we used *Spot* modeling based on EGFP fluorescence, rather than *Surface* modeling. Although we initially tested surface reconstruction, it frequently merged adjacent oocytes into a single surface, and manual correction proved prohibitively time- and computation-intensive for large datasets. In contrast, *Spot* modeling is well suited for object-counting applications. We therefore applied expected spot diameters of 18 μm for primordial follicular oocytes and 45 μm for growing

oocytes, which allowed accurate and efficient quantification of total follicle numbers. To illustrate segmentation fidelity, we have added overlays of the spot-modeling masks with the corresponding IF images, demonstrating the accuracy of follicle counting **(Fig. R2; See below)**.

For the quantitative analyses shown in Figure 1H, oocyte diameter, follicle volume, and granulosa-cell number were obtained by combining manual *Contour* definition with automated *Surface* and *Spot* modeling in Imaris. Follicular boundaries were delineated based on nuclear (DAPI) staining, and within each manually defined contour, EGFP signals were used for surface modeling of oocytes, while nuclear signals were used for spot modeling of granulosa cells. This workflow enabled precise measurement of follicular morphology and cellular composition. Representative examples of each modeled follicle type are shown in Figure 1G, where segmentation masks are overlaid with corresponding IF images to illustrate modeling accuracy across developmental stages. In total, nine primordial, seven primary, and four secondary follicles were reconstructed and analyzed, representing the size range and morphological diversity across follicular developmental stages. These details have been added to the Methods section **(text lines 593–603 in revised manuscript)**.

Figure R2

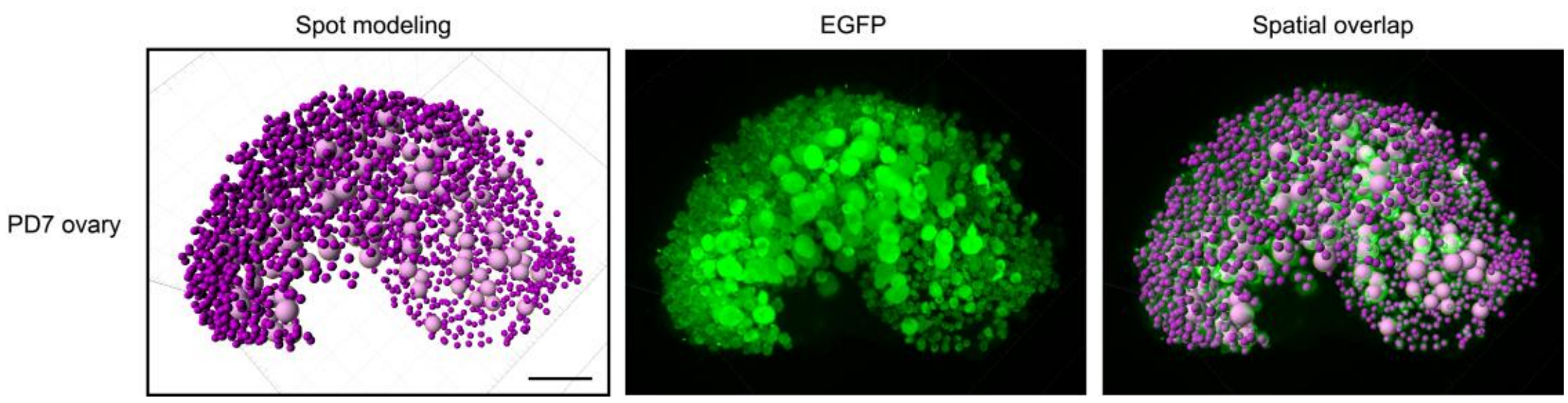

Figure R2. Spot modeling and immunofluorescence (IF) overlay for follicle counting. Spot modeling was applied with expected diameters of 18 μm for primordial follicular oocytes (deep pink) and 45 μm for growing oocytes (light pink), allowing accurate quantification of total follicle numbers. Overlays of the spot-modeling masks with the corresponding IF images demonstrate the fidelity of segmentation, with deep

pink representing small follicle modeling and light pink representing large follicle modeling. Scale bars, 100 μm.

9. In Figure 1, further validation is needed to show that all oocytes are being labeled through the APEX system. I recommend co-staining with DDX4, a germ cell marker, and GFP. If the APEX system is capturing all oocytes, then DDX4 and GFP will co-localize in all oocytes. Additionally, more information is needed on how image analysis was performed, specifically how oocyte diameter and follicle volume was calculated.

Response: We thank the reviewer for this insightful comment. To confirm that APEX expression labels all oocytes, we have performed new co-immunostaining experiments with GFP and DDX4, a well-established germ cell marker. DDX4 and GFP co-localized in nearly all oocytes, confirming that APEX labeling specifically targets oocytes **(Fig. 1e, text lines 132–135 in revised manuscript; Also see below)**. The image analysis workflow has also been clarified in the revised manuscript. As detailed in the response to Comment #8, for follicle quantification, *Spot* modeling was applied to the EGFP fluorescence channel, as it provided reliable object-based counting without merging adjacent oocytes, a common issue encountered in *Surface* reconstruction. For the measurement of oocyte diameter, follicle volume, and granulosa-cell number, manual *Contour* definition was combined with automated *Surface* and *Spot* modeling in Imaris. Follicular boundaries were delineated based on nuclear (Hoechst 33342) staining, and within each manually defined contour, EGFP signals were used for oocyte *Surface* modeling, while nuclear signals were used for granulosa-cell *Spot* modeling. This workflow enabled precise measurement of follicular morphology and cellular composition across different developmental stages. These details have been added to the Methods section **(text lines 593–603 in revised manuscript)**.

Figure 1

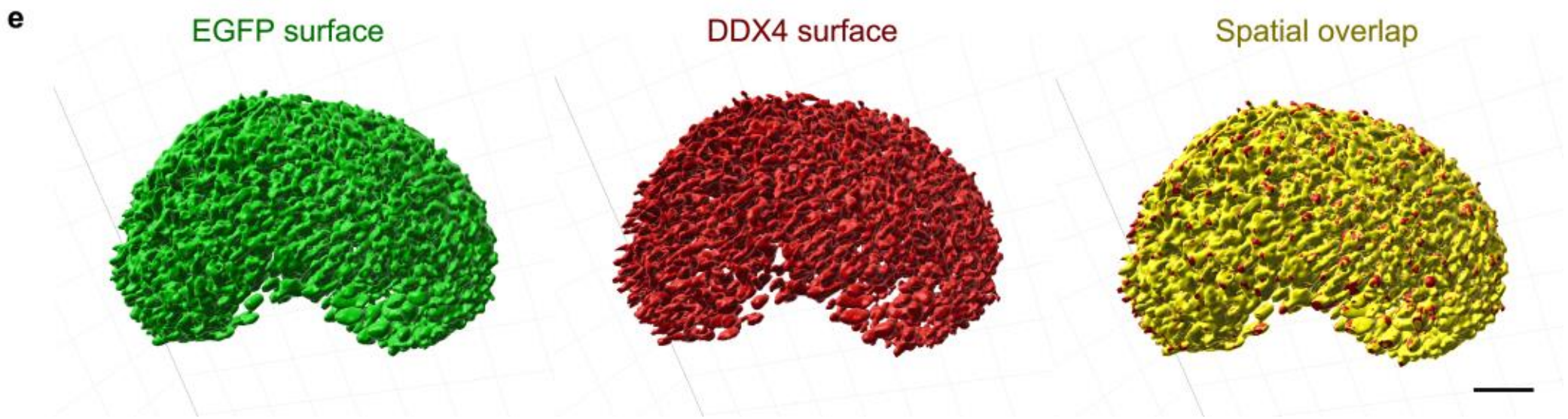

Figure 1. Targeting active APEX2 to *in vivo* follicle-enclosed oocytes. e Surface rendering of EGFP signal and DDX4 immunostaining in a PD7 ovary. EGFP-positive oocytes are shown in green, DDX4-positive germ cells in red, and their spatial overlap in yellow. Scale bars, 100 μm.

10. Fig 2 - 2C - With the current image, I am not convinced that all the primordial follicles are being labeled with streptavidin. It looks like growing follicles but not primordial follicles are labeled with streptavidin. I recommend performing a co-stain with DDX4, a germ cell marker, to show that all the oocytes, especially the primordial oocytes, are captured with APEX.

Response: We thank the reviewer for this valuable comment. To further verify that APEX labeling captures all oocytes, including those in primordial follicles, we have performed additional co-immunostaining with Streptavidin and DDX4, a well-established germ cell marker, on histological sections of ovaries after the APEX reaction at both PD7 and PD21. Substantial overlap of Streptavidin and DDX4 signals was observed, demonstrating that APEX expression driven by *Gdf9*-Cre labels oocytes across all developmental stages, including primordial follicles. Notably, Streptavidin-positive signals were clearly detected in quiescent primordial follicles at PD21, suggesting that although *Gdf9*-Cre activity is lower in these oocytes, it remains sufficient for APEX-mediated labeling **(Fig. 2c, text lines 173–182 in revised manuscript; Also see below)**.

Figure 2

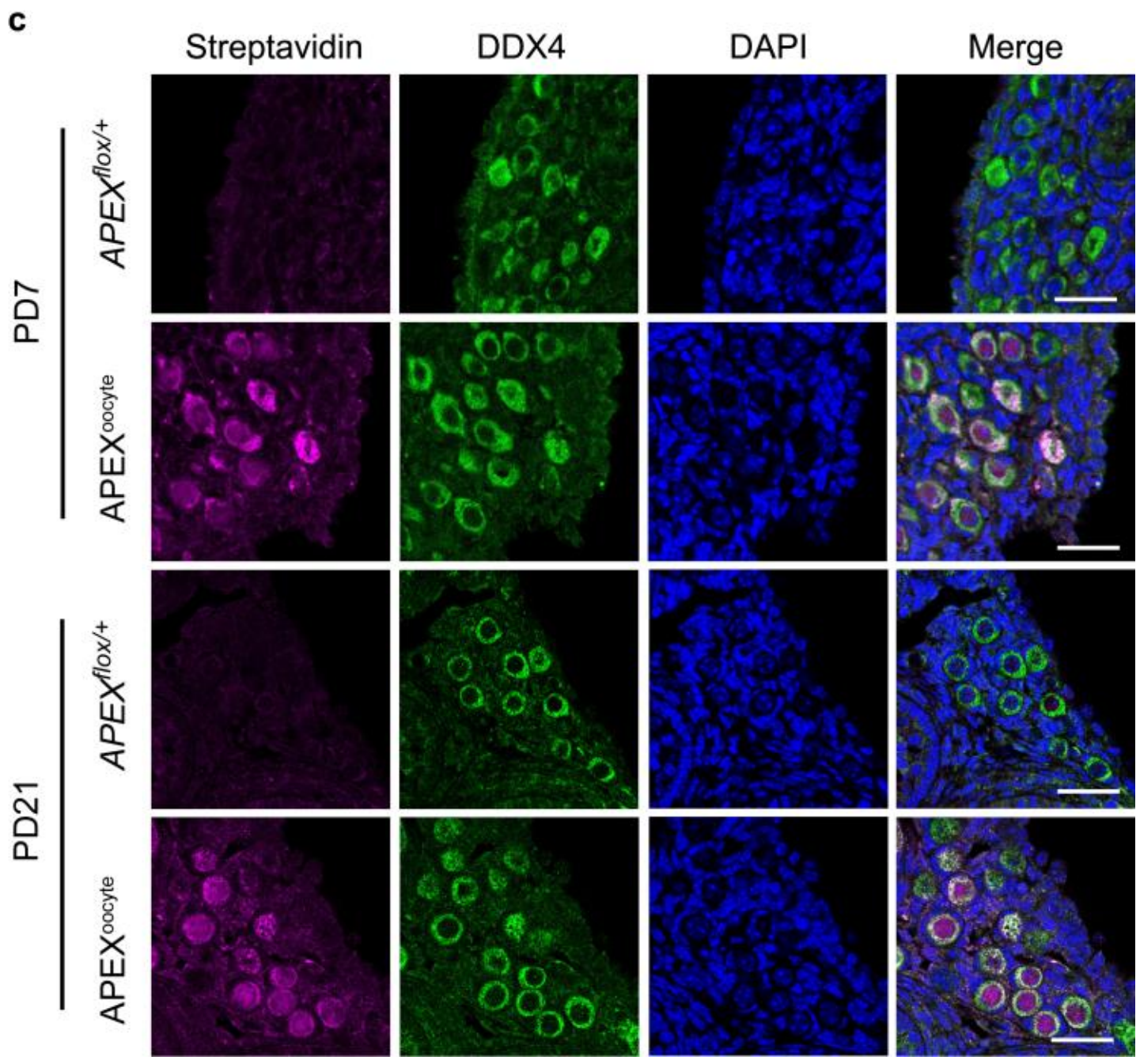

Figure 2. *In situ* APEX-based proteomic profiling of oocytes from early-stage ovarian follicles. c Streptavidin labeling in ovarian sections at PD7 and PD21 in $APEX^{oocyte}$ and $APEX^{flox/+}$ mice, respectively. Co-staining for streptavidin (magenta), DDX4 (green), and DAPI (blue) is shown. Scale bars, 50 μm.

11. Line 150 - It is unclear how hydrogen peroxide is administered. Is the mouse injected for 5 minutes or is the mouse injected and collected after 5 minutes? Injecting hydrogen peroxide does not seem healthy for the animal. Did you confirm that hydrogen peroxide injections do not negatively impact the health of the mouse, more specifically did you confirm that this inject does not affect ovarian health? Consider looking at apoptosis in the ovary after treatment and/or confirming these mice are still fertile after this treatment.

Response: We thank the reviewer for raising this important point. In our experiments, mice were intraperitoneally injected with H_2O_2 (1mM) once following BP incubation, and ovaries were collected 5 minutes after the injection and immediately placed in quenching buffer to terminate the APEX reaction, rather than being injected continuously for 5 minutes. We have carefully evaluated the potential impact of our

labeling procedure, including H_2O_2 injection, on ovarian physiology, oocyte function, and the proteome.

Histological analysis of ovaries at PD7 and PD21 revealed normal gross morphology, with stage-based follicle counts and total follicle numbers comparable between BP+H_2O_2- and PBS-treated groups (all n.s., $P > 0.05$). Consistently, cleaved caspase-3 staining at PD21 showed similar numbers of apoptotic follicles across groups, with no $CC3^+$ cells detected at the primordial or primary stages. TUNEL assays at PD7 further confirmed no increase in apoptotic primordial follicles in the BP+H_2O_2 group relative to PBS (all n.s., $P > 0.05$). In addition, FGOs collected at P21 after *in vivo* labeling exhibited normal *in vitro* maturation, with GVBD and PBE rates as well as spindle morphology comparable to controls (**Supplementary Figs. 2a–i; Also see below**). These results indicate that the labeling procedure did not measurably affect follicle/oocyte morphology or developmental competence.

To address the possible effects of H_2O_2 treatment on the ovarian proteome, we performed differential expression analysis of PD7 ovaries with or without H_2O_2 injection. Among the 6055 proteins detected, only 142 proteins were differentially expressed (45 upregulated and 97 downregulated), accounting for only ~2% of the total proteome. No oxidative stress–related pathways were significantly enriched among the dysregulated proteins, further supporting that the proteomic impact of H_2O_2 treatment was minimal **(Supplementary Figs. 2j–m; Also see below)**.

Taken together, evidence from histological analysis, apoptosis assays, oocyte maturation studies, and proteomic profiling consistently demonstrates that the H_2O_2 conditions applied in our APEX labeling system do not induce measurable oxidative stress or damage. Therefore, this procedure does not confound the reliability of our proteomic analysis. We have added these data to the **revised Figure (Supplementary Fig. 2; also see below** and updated **the corresponding text (lines 182–192 and 200–207)** in the Results section.

Supplementary Figure 2

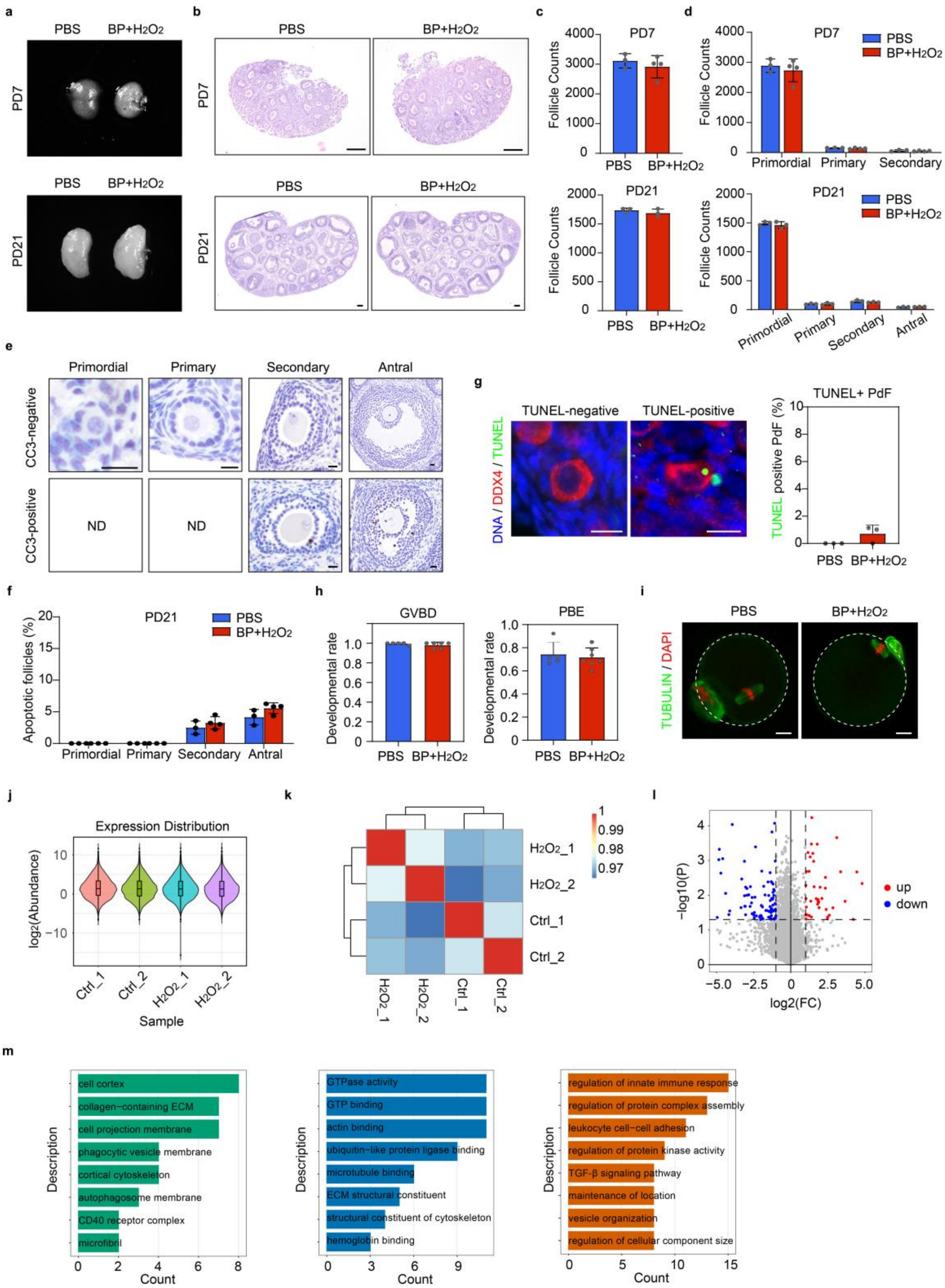

Supplementary Figure 2. The effects of APEX labeling reaction on ovarian physiological processes. a Gross morphology of ovaries collected at PD7 and PD21 from PBS- or BP+H_2O_2-treated mice. **b** H&E-stained ovarian sections showing comparable follicular architecture between groups at PD7 and PD21. Scale bars, 100

μm. **c–d** Quantification of total follicle numbers (**c**) and stage-specific follicle counts (**d**) at PD7 (top) and PD21 (bottom). Mean ± SEM. PD7 (n = 3, 4) and PD21 (n = 3, 3) for PBS and BP + H_2O_2, respectively. **e** Representative images of cleaved caspase-3 (CC3) staining in follicles at different stages at PD21. "ND", not detected. Scale bars, 20 μm. **f** Quantification of apoptotic follicles detected by CC3 staining at PD21. Mean ± SEM. PD21 (n = 3, 4) for PBS and BP + H_2O_2, respectively. **g** TUNEL staining of primordial follicles at PD7 showing representative TUNEL-negative and TUNEL-positive examples (left) and statistical comparison of $TUNEL^+$ primordial follicles per ovary (right). Scale bars, 20 μm. Mean ± SEM. n = 3 for each group. **h** *In vitro* maturation of FGOs collected at PD21 following *in vivo* labeling. Mean ± SEM. GVBD (n = 5, 6) and PBE (n = 5, 7) for PBS and BP + H_2O_2, respectively. **i** Representative spindle morphology of *in vitro* matured oocytes, visualized by α-tubulin (green) and DNA staining (red), demonstrating intact meiotic spindle formation in both groups. Scale bars, 20 μm. **j** Distribution of all ovarian proteins expressed in control (Ctrl) and H_2O_2-treated mice, with log2-transformed abundance across biological replicates. Center line, median; box limits, upper and lower quartiles; whiskers, 1.5× interquartile range; points, outliers. **k** Correlation heatmap showing the expression similarity between Ctrl and H_2O_2 treated groups based on total protein expression data. **l** Volcano plot of differentially expressed genes comparing H_2O_2 treatment and control groups. Red dots represent upregulated genes, blue dots represent downregulated genes. **m** Gene ontology (GO) term enrichment analysis of differentially expressed genes. Cellular components (green), molecular functions (blue), and biological processes (orange) significantly enriched in treated vs control groups.

12. I recommend the authors add more citations on current techniques and knowledge in the ovarian biology field. There are claims of novel findings that have been previously reported in the literature, such as the ability to optically clear, segment and count follicles in the 3D ovary (lines 140-145), or the fact that the ovarian cortex contains more primordial follicles (line 463 to 465), which was

clearly demonstrated in Cordeiro et al, 2015: doi: 10.1095/biolreprod.115.131227.

Response: We thank the reviewer for highlighting the importance of acknowledging prior contributions to the field. We agree that several studies have established protocols for visualizing the entire perinatal ovary. We have now cited and discussed these works in the revised manuscript **(lines 503–508)**. Compared with previous works, our system offers a rapid, antibody-free in-situ imaging workflow that substantially reduces processing time and avoids antibody penetration issues. It can also be readily extended to diverse Cre-driver lines, including conditional knockouts, without requiring additional antibody optimization. At the same time, we recognize that the approach depends on genetically engineered mouse models, which is an inherent requirement of the APEX system and may be viewed as a technical limitation relative to antibody-based approaches. The revised manuscript now provides a balanced description of both the novelty of our workflow and the practical considerations associated with its application **(text lines 503–518 in revised manuscript)**. In addition, we have revised the sentence to accurately reflect that our observations are consistent with established knowledge, such as that reported by Cordeiro et al. (2015), while emphasizing that our method enables direct and quantitative visualization of this distribution in intact ovarian tissue **(text lines 511–518 in revised manuscript)**.

13. It is important to consider how the proteome of oocytes might be different between early postnatal ovaries and adult ovaries, especially considering how the hormonal environment differs between early postnatal and adult mice. This could be discussed as a limitation of the study.

Response: We thank the reviewer for this insightful comment. We agree that even oocytes at the same follicular stage may exhibit proteomic differences between early postnatal and adult ovaries, given the distinct hormonal and physiological environments at these stages. In our study, we focused on postnatal day 7 (PD7) ovaries because this developmental stage is dominated by primordial follicles, whereas in later stages, the proportion of growing follicles increases substantially, making it difficult to selectively

capture the proteome of primordial oocytes. To further address this point, we have now examined the expression of representative oocyte proteins across multiple developmental stages, including postnatal (PD7), adolescent (2-week-old), adult (3–4-week-old), and aged (12-month-old) ovaries. Immunostaining results showed that all analyzed proteins, including TBK1, AAK1, BAG6, and MAEA, were expressed in primordial follicles across ages. Notably, although at lower abundance, MAEA remained detectable in 12-month-old mouse ovaries **((Supplementary Figs. 4e–f, text lines 295–299 in revised manuscript; Also see below)**. We have also expanded the Discussion section to acknowledge that age-related differences in the hormonal milieu may influence the oocyte proteome, which represents an important consideration and limitation of our current study **(text lines 525–527 in revised manuscript)**.

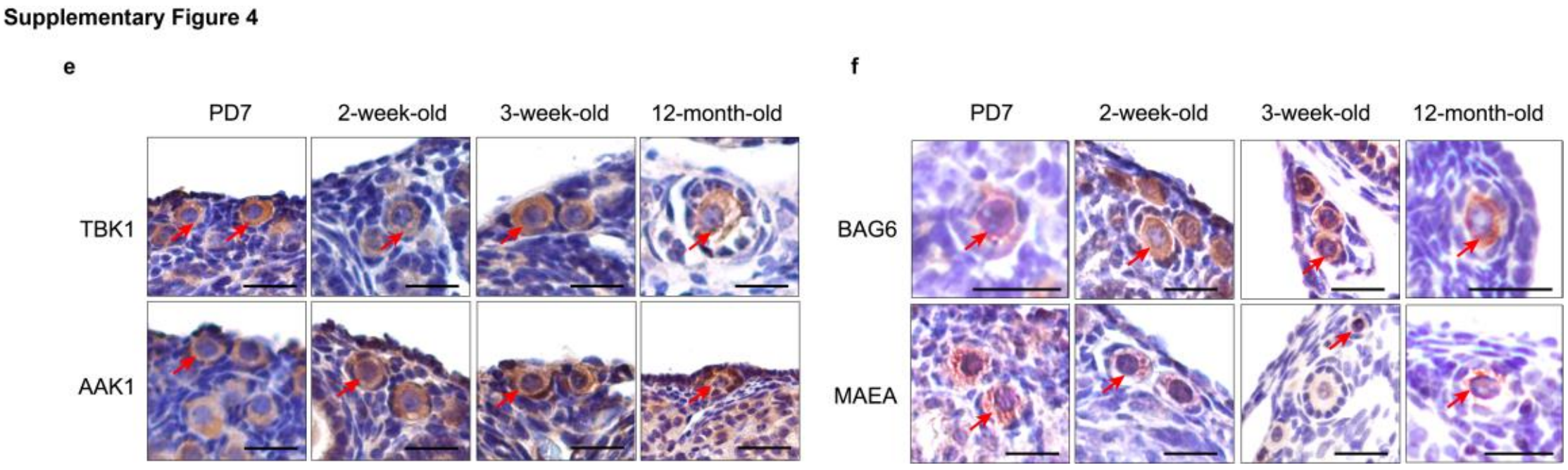

Supplementary figure 4. Interaction networks and age-independent expression of regulatory proteins in oocytes. e Immunohistochemical staining of TANK binding kinase 1 (TBK1) and AP2 associated kinase 1 (AAK1) in ovarian sections from PD7, 2-week-old, 3-week-old, and 12-month-old mice. Arrows indicate primordial follicle oocytes. Scale bars, 20 µm. **f** Immunohistochemical staining of BAG cochaperone 6 (BAG6) and macrophage erythroblast attacher (MAEA) in ovarian sections from PD7, 2-week-old, 3-week-old, and 12-month-old mice. Arrows indicate primordial follicle oocytes. Scale bars, 20 µm.

14. I recommend changing the title and discussion to reflect that APEX is capturing primordial, primary, and secondary follicles. Based on validation in

Figure 1 and Figure 2, it seems that APEX is not capturing many primordial follicles.

Response: We thank the reviewer for this thoughtful suggestion. We acknowledge that although PD7 ovaries are predominantly composed of primordial follicles, a small proportion of primary and secondary follicles are also present at this stage. Therefore, it is possible that a minor fraction of proteins originating from early growing oocytes were included in our dataset. To minimize this potential influence, we optimized several parameters, including shortening the BP incubation period to 1 hour to restrict substrate diffusion, increasing biological input, applying stringent washing procedures, and refining data filtering by excluding proteins previously reported only in fully grown oocytes. Validation through immunostaining further confirmed the expression of representative oocyte proteins within primordial follicles, supporting that the identified proteome is largely derived from primordial follicle oocytes **(Figs. 2e-i, Fig. 3, Supplementary Figs. 3d-e, Supplementary Fig. 4, text lines 207–302 in revised manuscript; Also see below)**.

In addition, we have verified that the APEX system effectively labels oocytes in primordial follicles. Co-immunostaining of Streptavidin and DDX4, a well-established germ cell marker, was performed on ovarian sections collected at PD 7 and PD21. Substantial overlap between Streptavidin and DDX4 signals was observed, indicating that *Gdf9*-Cre–driven APEX expression labels oocytes across follicular stages, including primordial follicles. Notably, Streptavidin-positive signals were also detected in quiescent primordial follicles at PD21, confirming that the APEX system successfully captures these oocytes, even though Cre activity in them is relatively weak **(Fig. 2c, text lines 174–182 in revised manuscript; Also see below)**.

Taken together, these refinements ensure that the revised proteomic dataset predominantly reflects primordial follicle oocytes while acknowledging a limited contribution from early growing follicles. Accordingly, we have slightly modified the title to "***In Vivo* Labeling–Based Proteomic Analysis of Early Follicle Oocytes and Cisplatin-Induced Alterations in Mice**". This adjustment provides a more balanced

description of the dataset, clarifying that while the focus remains on primordial follicles, the analysis encompasses the earliest stages of follicular development.

Figure 2

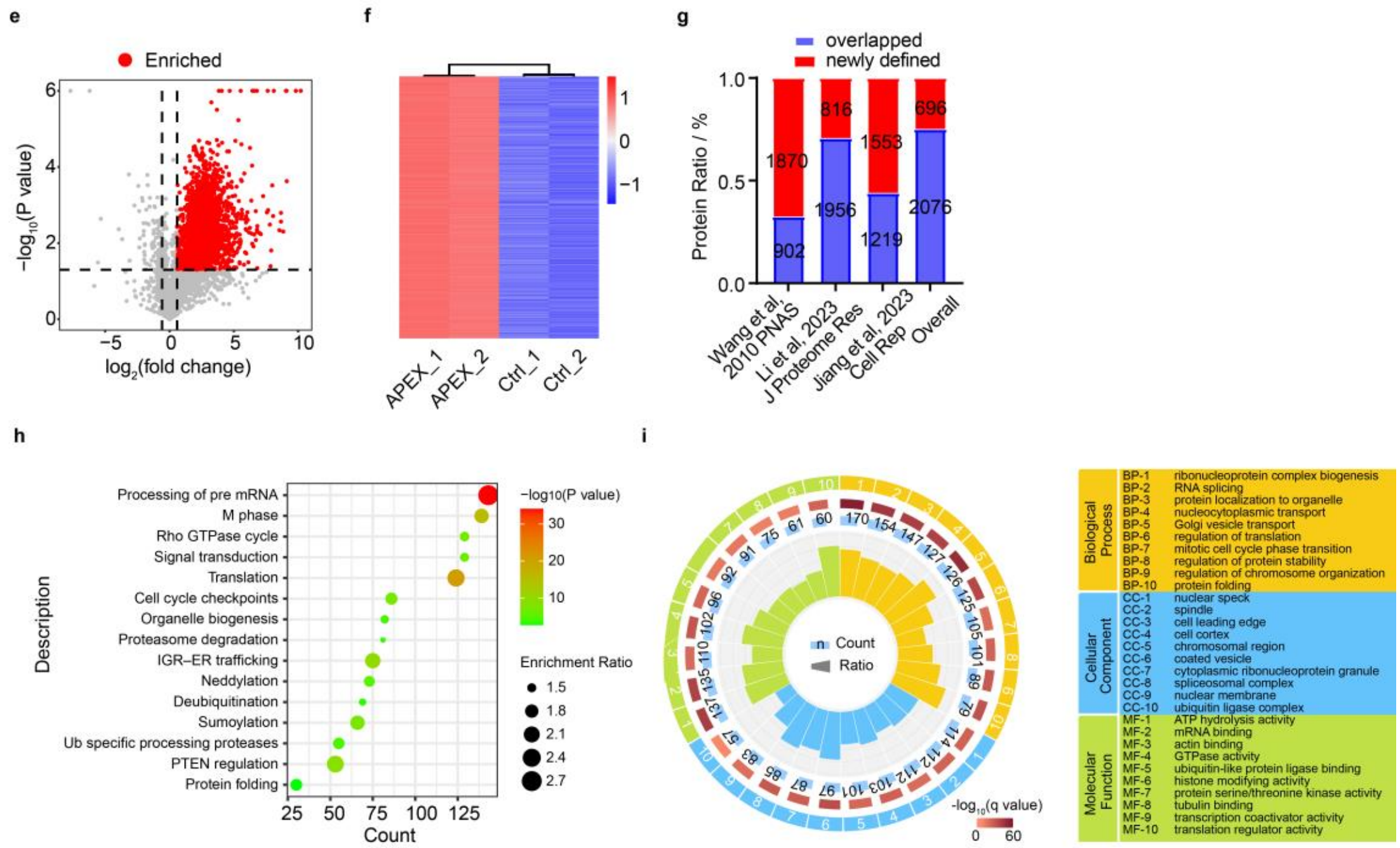

Figure 2. *In situ* APEX-based proteomic profiling of oocytes from early-stage ovarian follicles. e Volcano plot of proteins detected by LC–MS/MS comparing $APEX^{oocyte}$ and $APEX^{flox/+}$ control samples. Significantly enriched proteins are highlighted (red dots, fold change > 1.5, $P < 0.05$). **f** Heatmap of enriched proteins across biological replicates of $APEX^{oocyte}$ and $APEX^{flox/+}$ (Ctrl) samples. **g** Overlap analysis of the identified proteome with previously published GV-stage oocyte proteomes. Stacked bars indicate overlapped and newly defined proteins. **h** Bubble plot of enriched Reactome pathways. The x-axis shows the gene number (Count), the y-axis lists pathway names, bubble size reflects enrichment ratio, and color indicates significance ($-\log_{10}$ [*P*-value]). **i** Circular plot of the top 10 enriched gene ontology (GO) terms in biological process (yellow), cellular component (blue), and molecular function (green). From inside to outside: enrichment factor (Ratio), gene number (Count), statistical significance ($-\log_{10}$ [q-value]), and GO term IDs corresponding to the descriptions on the right.

Supplementary Figure 3

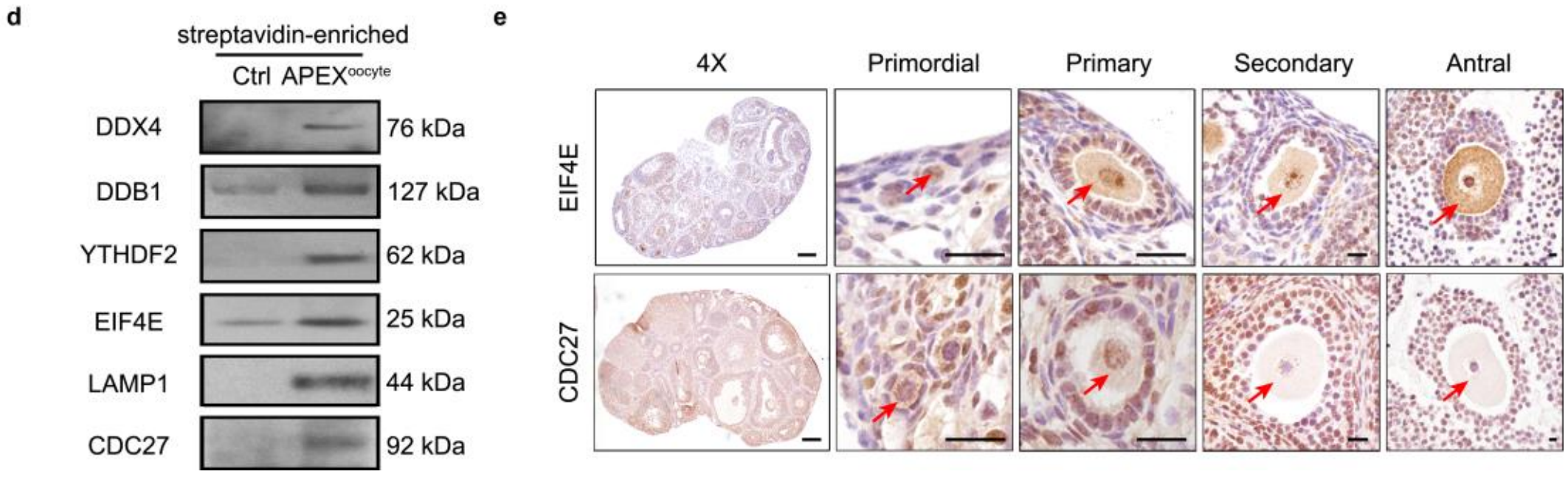

Supplementary Figure 3. Quality assessment and validation of the streptavidin-enriched oocyte proteome. d Western blot analysis of representative proteins in streptavidin-enriched fractions from control (Ctrl) and APEX^{oocyte} ovaries. **e** Immunohistochemical staining of eukaryotic translation initiation factor 4E (EIF4E) and cell division cycle 27 (CDC27) in PD21 ovarian sections. Images include low-magnification overview and higher-magnification views of primordial, primary, secondary, and antral follicles. Arrows indicate oocytes. Scale bars, 100 μm in 4× microscopy and 20 μm in enlarged views.

Figure 3

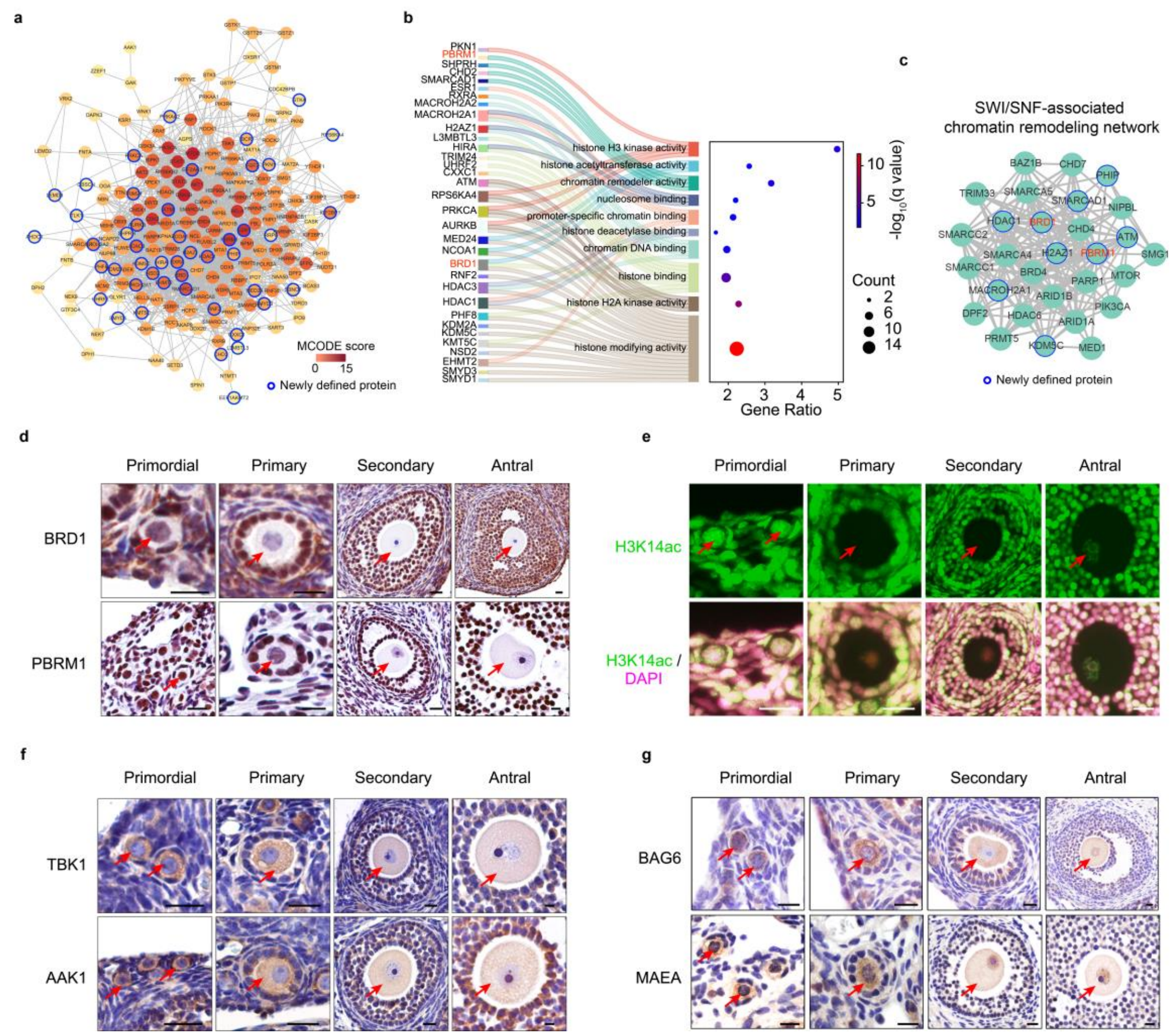

Figure 3. Expression dynamics of representative regulatory proteins in oocytes. a Protein–protein interaction network of chromatin regulation–associated proteins identified in the oocyte proteome. Nodes represent proteins, with newly defined proteins indicated by blue-bordered circles. Red nodes represent proteins with higher MCODE scores, indicating highly interconnected proteins within the network. **b** Sankey plot showing the relationship between newly defined proteins (left) and functional categories (right). The size and color of the bubbles on the right correspond to the gene number (Count) and –log(P-value), respectively, representing the enrichment level of each functional category. **c** Subnetwork of SWI/SNF-associated chromatin remodeling proteins. Newly defined proteins are indicated by blue-bordered circles. **d** Immunohistochemical staining of bromodomain containing 1 (BRD1) and polybromo 1 (PBRM1) in ovarian sections across follicle stages (primordial, primary, secondary, and antral). Arrows indicate oocytes. Scale bars, 20 μm. **e** Immunofluorescence staining of histone H3 lysine 14 acetylation (H3K14ac) (green) in ovarian sections across follicle stages. Merged images with DAPI (magenta) are

shown. Arrows indicate oocytes. Scale bars, 20 μm. **f** Immunohistochemical staining of TANK binding kinase 1 (TBK1) and AP2 associated kinase 1 (AAK1) in ovarian sections across follicle stages. Arrows indicate oocytes. Scale bars, 20 μm. **g** Immunohistochemical staining of BAG cochaperone 6 (BAG6) and macrophage erythroblast attacher (MAEA) in ovarian sections across follicle stages. Arrows indicate oocytes. Scale bars, 20 μm.

Supplementary Figure 4

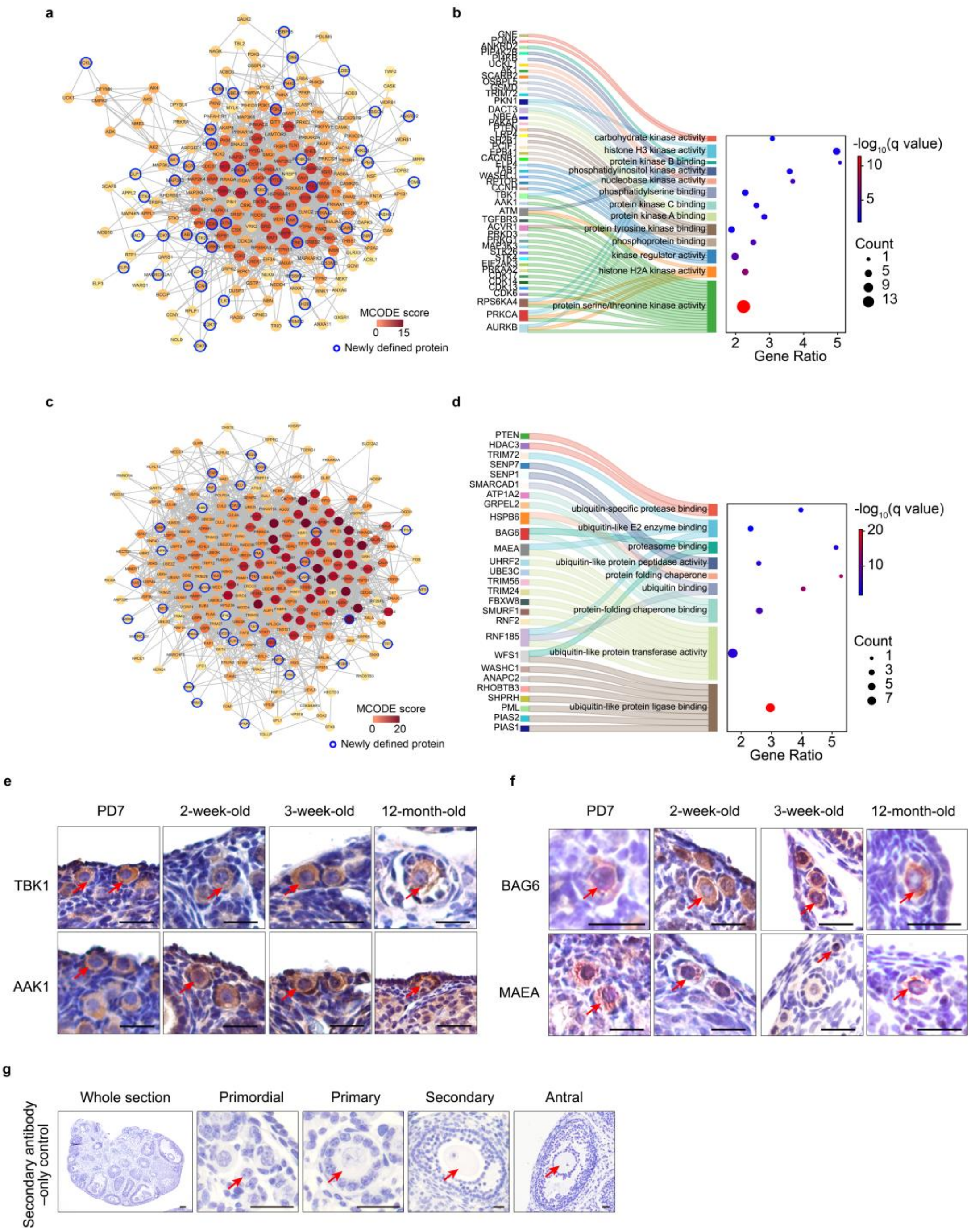

Supplementary Figure 4. Interaction networks and age-independent expression of regulatory proteins in oocytes. a Protein–protein interaction network of kinase-related regulatory proteins identified in the oocyte proteome. Nodes represent proteins, with newly defined proteins indicated by blue-bordered circles. Node color intensity reflects MCODE score. **b** Functional enrichment analysis of proteins in the kinase-associated network. The Sankey diagram links newly defined proteins (left) to enriched molecular function categories (right). In the bubble plot, bubble size represents protein count and color indicates $-\log_{10}$ (q value). **c** Protein–protein interaction network of proteins associated with protein stability and ubiquitin–proteasome regulation. Newly defined proteins are indicated by blue-bordered circles. Node color intensity reflects MCODE score. **d** Functional enrichment analysis of proteins in the protein homeostasis network. The Sankey diagram links newly defined proteins (left) to enriched molecular function categories (right). In the bubble plot, bubble size represents protein count and color indicates $-\log_{10}$ (q value). **e** Immunohistochemical staining of TANK binding kinase 1 (TBK1) and AP2 associated kinase 1 (AAK1) in ovarian sections from PD7, 2-week-old, 3-week-old, and 12-month-old mice. Arrows indicate primordial follicle oocytes. Scale bars, 20 μm. **f** Immunohistochemical staining of BAG cochaperone 6 (BAG6) and macrophage erythroblast attacher (MAEA) in ovarian sections from PD7, 2-week-old, 3-week-old, and 12-month-old mice. Arrows indicate primordial follicle oocytes. Scale bars, 20 μm. **g** Secondary antibody–only control staining across follicle stages in PD21 ovarian sections. Arrows indicate oocytes. Scale bars, 100 μm in 4× microscopy and 20 μm in enlarged views.

Figure 2

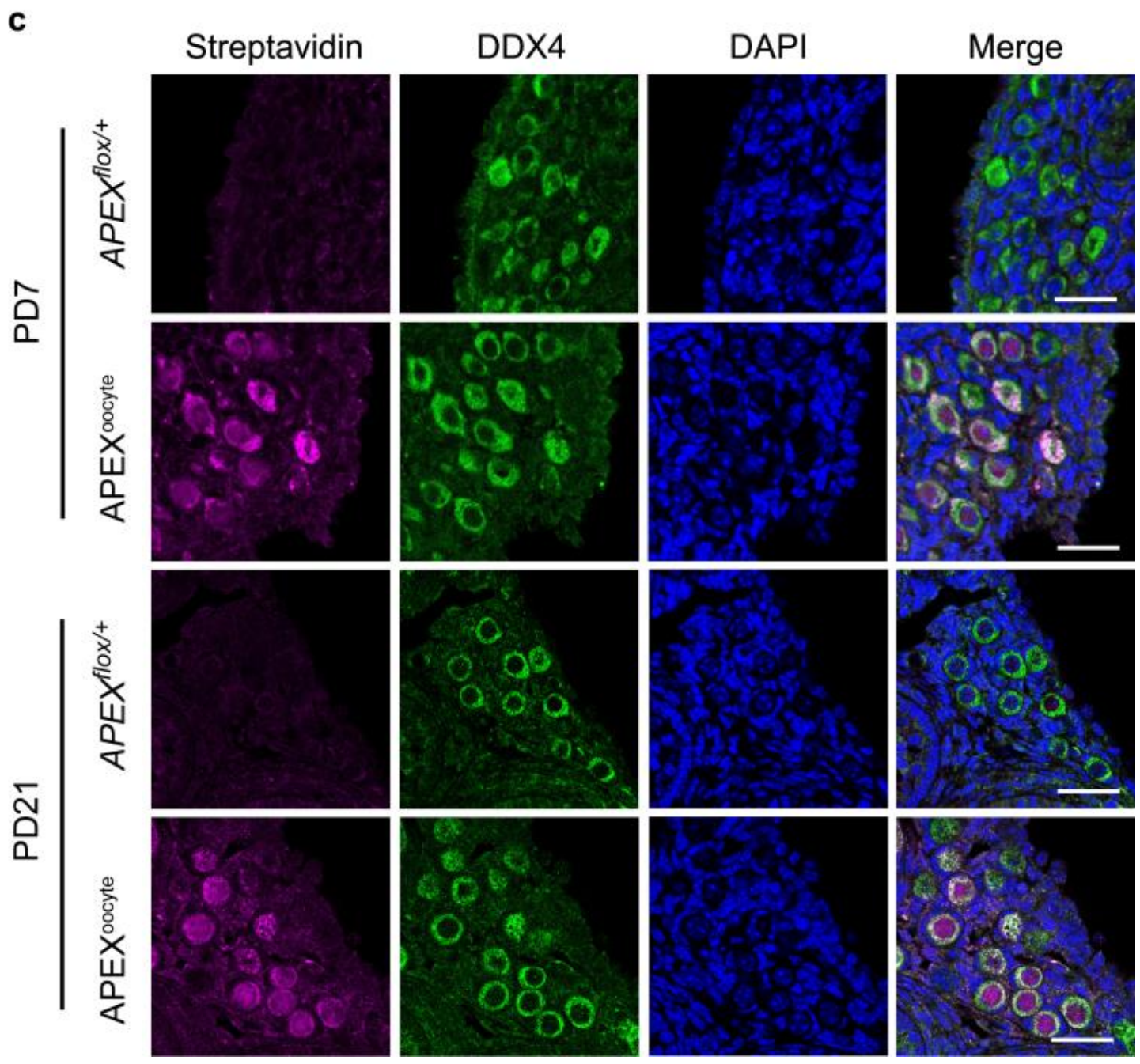

Figure 2. *In situ* APEX-based proteomic profiling of oocytes from early-stage ovarian follicles. c Streptavidin labeling in ovarian sections at PD7 and PD21 in APEX^{oocyte} and *APEX$^{flox/+}$* mice, respectively. Co-staining for streptavidin (magenta), DDX4 (green), and DAPI (blue) is shown. Scale bars, 50 μm.

Minor comments/limitations:

Line 30 - The word "realized" in this sentence is confusing. Consider rewording.

Response: We thank the reviewer for this suggestion. The revised sentence now reads: "With these mice, we reconstructed the ovary in 3D, enabling precise quantification of follicles…" **(text lines 30 in revised manuscript)**.

Line 73 - A more in depth explanation of APEX systems is needed.

Response: We thank the reviewer for this suggestion. A more detailed explanation of APEX systems has now been added to the revised manuscript **(lines 78–87)**.

Line 83 - Reconsider using the word "panorama" to describe a 3D image.

Response: We thank the reviewer for this suggestion. The revised sentence now reads: "Using EGFP-tagged APEX signals, we generated a 3D reconstruction of the entire ovary…" **(text lines 89 in revised manuscript)**.

Line 124 - Compare the number of oocytes counted to previously published counts. Are all the oocytes being captured?

Response: We thank the reviewer for this valuable suggestion. In our 3D reconstruction of PD7 mouse ovaries, we identified 2803±102, 100±10, and 55±11 oocytes within primordial, primary, and early secondary follicles, respectively (Fig. 1E, F). These counts closely matched those obtained from histological section analyses in this study (3035±483, 137±27, and 69±32 follicles at corresponding stages; Fig S1B). Importantly, our values fall within the previously reported range for PD7 mouse ovaries, in which the number of primordial follicles typically varies between 2,000 and 3,000 [10, 11, 12, 13]. Minor variations in follicle numbers among studies are expected, likely reflecting differences in mouse strain background, parental genotype, and environmental or nutritional conditions. These literature comparisons have been added to the revised manuscript **(lines 139–140)**.

Line 138 - How is the border of the cortex being defined?

Response: We appreciate the reviewer's question. In our analysis, the cortex was defined morphologically rather than by a strict anatomical border, as no sharp demarcation exists between the ovarian cortex and medulla. Specifically, the cortex was delineated as the peripheral region extending from the ovarian surface epithelium to the zone densely populated with small, uniform primordial follicles (corresponding to the green regions in Fig. 1i), while the inner region containing larger, well-separated growing follicles was designated as the medulla (corresponding to the pink regions in Fig. 1i). The transition between these two regions was defined at the point where the compact distribution of primordial follicles became discontinuous and growing follicles began to predominate. This consistent morphological criterion allowed reproducible identification of the cortical area across samples. These details have been added to the

Methods section **(text lines 603–606 in revised manuscript)**.

Line 162 - Should "7D ovaries" be PN7 ovaries?

Response: We thank the reviewer for noting this. The term "7D ovaries" has been corrected to "PD7 ovaries" (postnatal day 7 ovaries) throughout the revised manuscript for consistency with standard developmental biology nomenclature **(text lines 180 in revised manuscript)**.

Figure 3A - What plot is this? What are the axes? I think this plot could be explained better in the text.

Response: We thank the reviewer for this question. The updated Figure 3a now reflects the revised protein list following dataset refinement. It presents a protein–protein interaction (PPI) network generated using Cytoscape with the STRING plugin. Each node represents an individual protein, and edges indicate known or predicted interactions. The network was visualized using the *Spring-embedder* algorithm, which arranges proteins based on interaction strength rather than Cartesian coordinates, and therefore the plot does not have defined axes. In the updated figure **(Fig. 3a; Also see below)**, nodes represent proteins, with newly defined proteins indicated by blue-bordered circles. Red nodes represent proteins with higher MCODE scores, indicating highly interconnected proteins within the network. The revised figure legend now explicitly describes the generation and visualization of the network, as well as the meaning of each node type **(text lines 1093–1097 in revised manuscript)**.

Figure 3

a

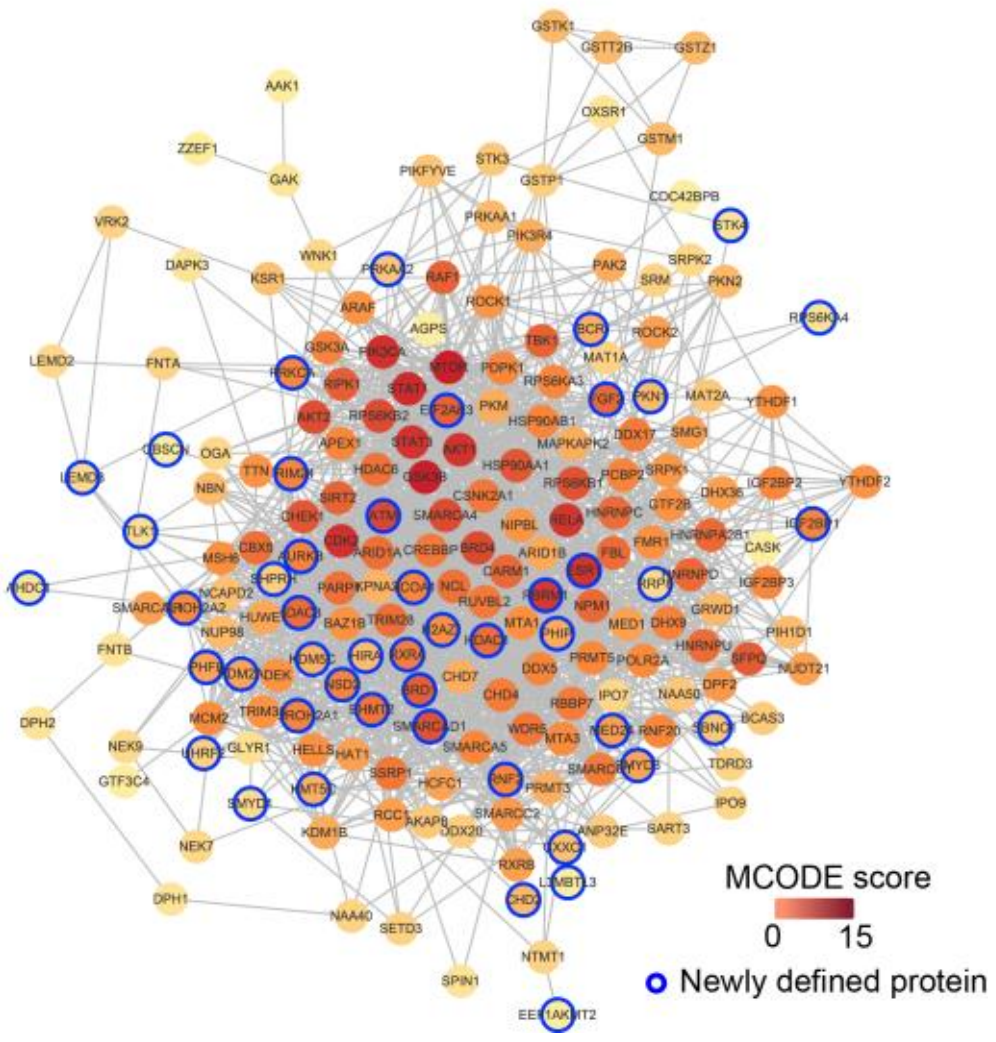

Figure 3. Expression dynamics of representative regulatory proteins in oocytes. a Protein–protein interaction network of chromatin regulation–associated proteins identified in the oocyte proteome. Nodes represent proteins, with newly defined proteins indicated by blue-bordered circles. Red nodes represent proteins with higher MCODE scores, indicating highly interconnected proteins within the network.

Figure 4F - Text behind the pink bubbles can't be read. Consider reformatting this plot.

Response: Thanks for the kind reminder. We have altered the position of the text now **(Fig. 4f; Also see below)**.

Figure 4

f

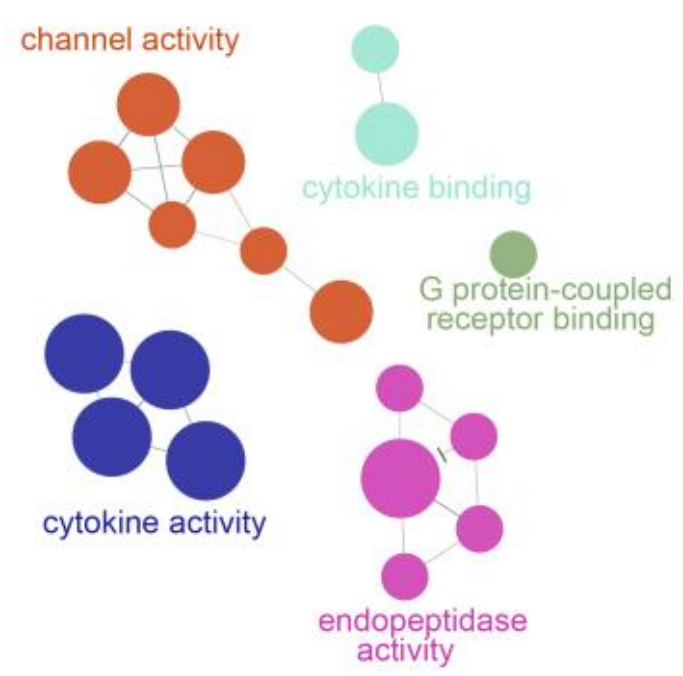

Figure 4. Characterization of the transcriptome of early follicle oocytes using APEX^{oocyte} labeling system. f GO enrichment analysis of newly detected early follicle oocyte transcripts.

Figure 5M - It would be beneficial to add arrows to show the reader where the spots are.

Response: Thank you for the helpful suggestion. We have added arrows in the figure to indicate the R-loop signal foci **(Fig. 5p; Also see below)**. In addition, we revised the description in the manuscript to use the more precise term "punctate nuclear R-loop signals" instead of "spots" **(text lines 406–408 in revised manuscript)**.

Figure 5

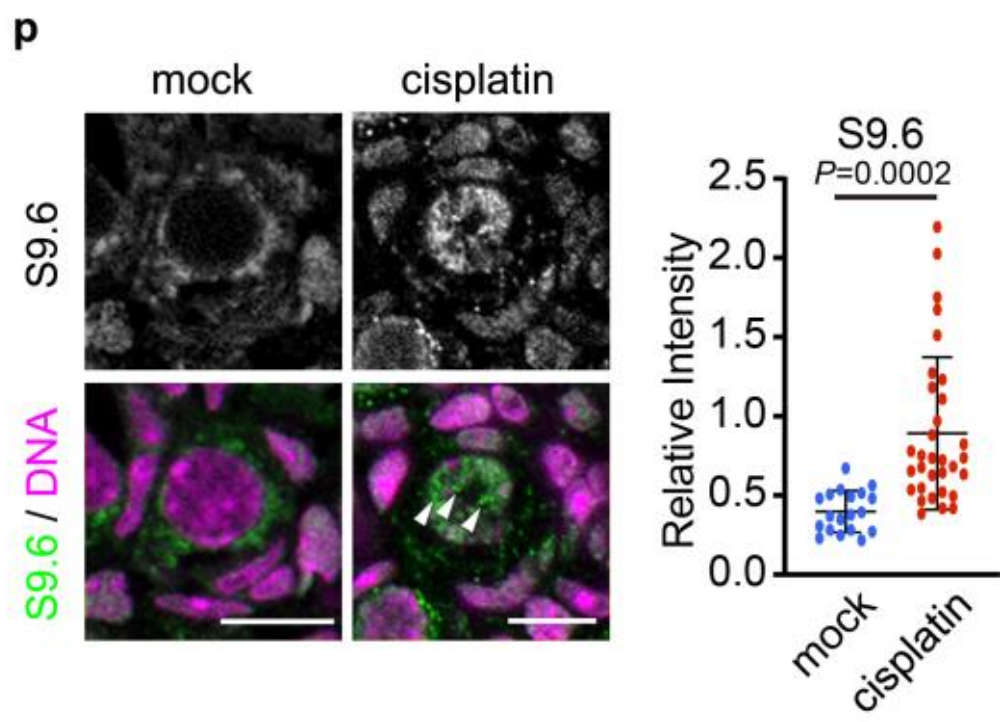

Figure 5. Cisplatin induces proteomic alterations in primordial follicle oocytes. p S9.6 staining detecting RNA–DNA hybrids (R-loops) and quantification before and after cisplatin treatment from PD7 mice. White arrows indicate puncta signals in the treated oocytes. Scale bar, 20 μm. Mean ± SEM. n = 19 and 32 for the mock and cisplatin groups, respectively. *P* values are calculated by unpaired two-tailed Student's t tests.

Line 454 - Include citations.

Response: Thanks for the reminder. We have added the citations here **(text lines 501 in revised manuscript)**.

Line 457 - The sentence beginning with "The conventional cross sections.." could be reworded for clarity.

Response: We thank the reviewer for this suggestion. The revised sentence now reads: "In research settings, ovarian cross-sections have traditionally been used to estimate follicle numbers, but this approach is inherently limited as it only shows granulosa cells surrounding the oocyte in a single plane and does not allow precise follicle counting." **(text lines 503–506 in revised manuscript)**.

Line 463 - This has been confirmed before, add citation (Cordeiro et al, 2015)

Response: Thanks for the kind reminder. The citation has been added here **(text lines 516 in revised manuscript)**.

Line 478 - Good discussion of limitations.

Response: We thank the reviewer for the positive feedback regarding our discussion of the study's limitations. In addition, we have further expanded this section to include consideration of potential age-related differences in oocyte proteomes **(text lines 525–527 in revised manuscript)**.

Line 996 - In the legend for Figure 1C, It would be helpful to include how these oocytes were isolated and what age the mice were.

Response: We thank the reviewer for this helpful suggestion. We have updated the Figure 1c legend to include details on oocyte isolation and mouse age. The revised text now reads: "COCs and FGOs were collected from 3–4-week-old female mice and released from antral follicles by gentle puncture." **(text lines 1046–1051 in revised manuscript)**.

Line 1015 - In the legend for Figure 2B, include if these are isolated follicles or if these images are from whole ovaries.

Response: We thank the reviewer for this helpful suggestion. We have clarified in the Figure 2b legend that the images were obtained from cumulus–oocyte complexes (COCs) collected from whole ovaries of 3–4-week-old female mice after biotinylation **(text lines 1072–1076 in revised manuscript)**.

Line 1043 - In the legend for Figure 3D, include how these oocytes were isolated and what age the mice were.

Response: We thank the reviewer for this helpful comment. The immunofluorescence results for oocytes in Figure 3 have been removed as they were not central to the focus of this paper. The oocytes used in the current manuscript were sourced from 3- to 4-week-old female mice, and the processing methods are now clarified in the updated Methods section and the Figure legend **(text lines 557–577 in revised manuscript)**.

Line 1128 - Include a sentence or two to summarize the graphical abstract.

Response: We thank the reviewer for the suggestion. We have added a brief description summarizing the graphical abstract: "Schematic overview of the *in vivo* APEX labeling strategy used in this study. BP and H_2O_2 were sequentially administered to induce APEX-mediated biotinylation of proteins within oocytes of living mice. The labeled ovaries were subsequently used for 3D imaging, proteomic profiling, and analysis of cisplatin-induced proteome shifts." **(text lines 1211–1214 in revised manuscript)**.

Reviewer #4:

I co-reviewed this manuscript with one of the reviewers who provided the listed reports. This is part of the Nature Communications initiative to facilitate training in peer review and to provide appropriate recognition for Early Career Researchers who co-review manuscripts.

Response: We appreciate the reviewer and their co-reviewer for their constructive and insightful comments that helped us improve the manuscript.

References

1. Yu C*, et al.* BTG4 is a meiotic cell cycle-coupled maternal-zygotic-transition licensing factor in oocytes. *Nature structural & molecular biology* **23**, 387-394 (2016).

2. Yu C*, et al.* CFP1 Regulates Histone H3K4 Trimethylation and Developmental Potential in Mouse Oocytes. *Cell reports* **20**, 1161-1172 (2017).

3. Liu HB*, et al.* RNA-Binding Protein IGF2BP2/IMP2 is a Critical Maternal Activator in Early Zygotic Genome Activation. *Advanced science (Weinheim, Baden-Wurttemberg, Germany)* **6**, 1900295 (2019).

4. Lan ZJ, Xu X, Cooney AJ. Differential oocyte-specific expression of Cre recombinase activity in GDF-9-iCre, Zp3cre, and Msx2Cre transgenic mice. *Biology of reproduction* **71**, 1469-1474 (2004).

5. Yu C*, et al.* CRL4 complex regulates mammalian oocyte survival and reprogramming by activation of TET proteins. *Science* **342**, 1518-1521 (2013).

6. Yu C*, et al.* Oocyte-expressed yes-associated protein is a key activator of the early zygotic genome in mouse. *Cell research* **26**, 275-287 (2016).

7. Sha QQ*, et al.* A MAPK cascade couples maternal mRNA translation and degradation to meiotic cell cycle progression in mouse oocytes. *Development (Cambridge, England)* **144**, 452-463 (2017).

8. Zhang J*, et al.* Mammalian nucleolar protein DCAF13 is essential for ovarian follicle maintenance and oocyte growth by mediating rRNA processing. *Cell death and differentiation* **26**, 1251-1266 (2019).

9. Dai XX*, et al.* PABPN1 functions as a hub in the assembly of nuclear poly(A) domains that are essential for mouse oocyte development. *Science advances* **8**, eabn9016 (2022).

10. Kerr JB, Duckett R, Myers M, Britt KL, Mladenovska T, Findlay JK. Quantification of healthy follicles in the neonatal and adult mouse ovary: evidence for maintenance of primordial follicle supply. *Reproduction (Cambridge, England)* **132**, 95-109 (2006).

11. Ren Y*, et al.* Lhx8 regulates primordial follicle activation and postnatal folliculogenesis. *BMC biology* **13**, 39 (2015).

12. Wang Y*, et al.* Doxorubicin obliterates mouse ovarian reserve through both primordial follicle atresia and overactivation. *Toxicology and applied pharmacology* **381**, 114714 (2019).

13. Chen YY, *et al.* Single-cell transcriptomics of staged oocytes and somatic cells reveal novel regulators of follicle activation. *Reproduction (Cambridge, England)* **164**, 55-70 (2022).

Response Letter

Dear Editor and Reviewers,

This is the second round of revision. We are very grateful for the constructive suggestions from the Editor and the Reviewers. As requested, we have point-by-point addressed both the concerns by Reviewer 1 and Reviewer 2. We hope this revised version will meet the quality standards for publication. For details, please see the response below.

REVIEWER COMMENTS

Reviewer #1:

The authors present a highly significant spatial proteomics study that maps the first in situ proteome of early-stage oocytes and uncovers the epigenetic mechanism underlying cisplatin-induced ovarian damage. While the authors have made significant efforts to optimize the proteomic workflow and improve the data quality during revision, there are still inherent technical limitations to the current methodology that must be explicitly acknowledged in the Discussion/Limitations section of the manuscript. I request the authors to add brief discussions addressing the following two points:

1. Although the authors employed stringent washing steps (e.g., High-salt, Urea) to reduce background noise, traditional streptavidin pull-down cannot entirely eliminate the risk of false positives. Specifically, non-biotinylated proteins that strongly interact with actual biotinylated proteins can be co-precipitated, especially during the post-lysis process. Because Super-Resolution Proximity Labeling (SR-PL) or direct identification of biotinylation sites at the peptide level was not performed, the dataset inherently carries the risk of including these indirect interactors. This limitation must be explicitly addressed in a Discussion part.

Response: We thank the reviewer for this valuable comment. We have now explicitly acknowledged this limitation in the Discussion section of the manuscript, where we clarify that the dataset may include indirectly associated proteins due to the nature of

the streptavidin pull-down method. The relevant details can be found in revised manuscript **(text lines 573-580)**.

2. The labeling intensity and the resulting proteomic profile are highly dependent on the administered dose and duration of BP and H2O2. Since the labeling is performed in vivo, pharmacokinetic variations can significantly affect the actual concentration of probes reaching the target ovary. As a dose-dependent optimization of the probes to maximize ovarian bioavailability was not performed, the potential variation in labeling outcomes depending on dose and time should be clearly stated in a Discussion or Method part.

Response: We thank the reviewer for this helpful comment. We have now clearly stated this point in the Discussion section, noting that because the actual concentration of probes reaching the ovary during *in vivo* labeling is influenced by the administered dose and pharmacokinetics, the labeling outcomes may vary without dose- and time-dependent optimization. The relevant details can be found in revised manuscript **(text lines 580-583)**.

Minor:

There are several typos in figure numbers and grammatical errors that need to be corrected before publication.

Response: We thank the reviewer for pointing this out. We have carefully checked the revised manuscript and corrected the typos in figure numbers as well as the grammatical errors throughout the text.

Reviewer #2:

The authors have undertaken a substantial revision of this manuscript, including additional samples, expanded datasets, new co-immunostaining experiments (DDX4/streptavidin), comprehensive H_2O_2 toxicity assessment, additional DDR protein validation (RAD51, RPA2, XRCC1), in vivo PD7 cisplatin ± GSK126

treatment experiments (although limited to short-term outcomes), and an appropriately modified title and softened language throughout. These represent a commendable amount of new work. However, the authors should ensure that all new results presented in the rebuttal are fully incorporated into the manuscript text, not only as figures. Several remaining concerns require attention before publication.

Response: We thank the reviewer for this helpful comment. We have carefully revised the manuscript to describe more explicitly in the main text the new results presented in our response letters during the revision process, rather than relying mainly on figure display. The relevant details can be found in revised manuscript **(text lines 188-196, 207-215, and 219-223)**.

Specific comments

1. Primordial follicle representation in the proteome/transcriptome: The additional data confirming that the proteome and transcriptome include primordial follicle oocytes are appreciated and substantially address the original concern. However, the manuscript (p. 10, lines 222–223) still describes the population of growing follicles in the PD7 ovary as "a few primary and secondary follicles," whereas the authors' own quantification data show roughly 200 growing follicles—a population that could contribute substantially to the labeled proteome. This understatement should be corrected. Additionally, the authors performed experiments with a shortened BP incubation time, claiming this should limit labeling in growing oocytes; however, this was not validated experimentally. Cross-sections of the ovary labeled with DDX4/streptavidin under both the original and shortened labeling protocols should be presented to support the claim that labeling in growing oocytes is decreased without compromising labeling efficiency in primordial follicles.

Response: We thank the reviewer for this thoughtful comment. First, we agree that the previous wording, "a few primary and secondary follicles," was not sufficiently objective. We have therefore revised this statement in the previous version of the Discussion section (Page 22, Line 524) to read: "primary and secondary follicles did

exist in PD7 mouse ovaries" **(lines 584-585 in revised manuscript)**. Second, to validate the labeling pattern under the shortened BP incubation protocol, we performed additional ovary section staining with DDX4/streptavidin co-immunostaining to compare the original and shortened BP incubation conditions (2 h and 1 h, respectively). The results showed that the labeling intensity of growing oocytes in the inner medullary region was markedly reduced in the 1 h group, while the labeling efficiency in primordial follicles was not compromised. We have added these data to the **revised Figure (Supplementary Fig. 2a; Also see below)** and updated **the corresponding text (lines 183–185)** in the Results section.

Supplementary Figure 2

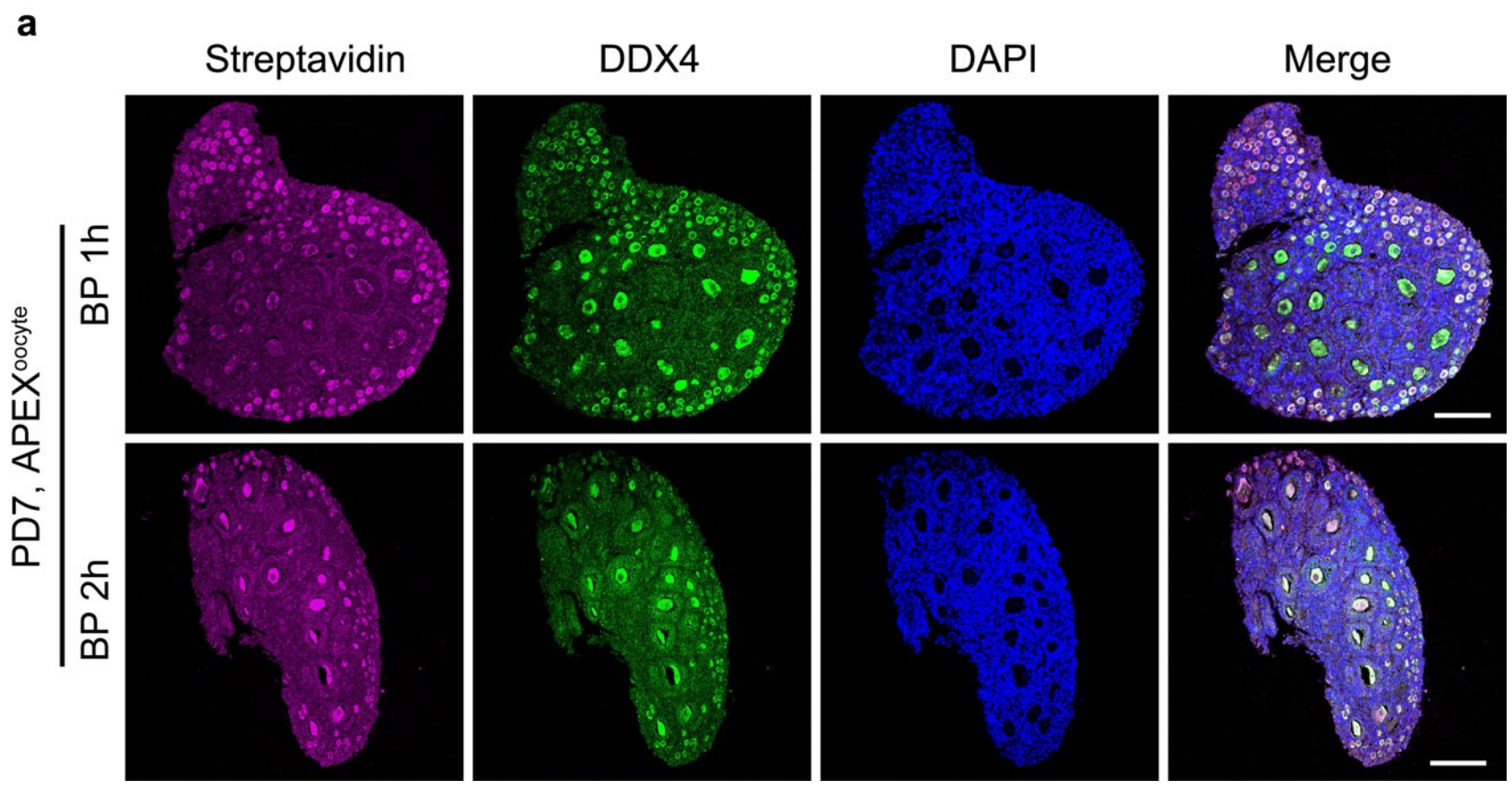

Supplementary Fig. 2a Representative ovarian sections from PD7 APEX^{oocyte} mice after *in vivo* APEX labeling with 1 h or 2 h BP incubation. Sections were stained with streptavidin, DDX4, and DAPI. Scale bars, 100 µm.

2. H_2O_2 toxicity and oxidative stress: The authors have provided comprehensive data addressing this concern, including histological analysis showing normal ovarian morphology and comparable follicle numbers, no increase in apoptosis, and normal meiotic maturation. Although some minor changes were detected, the presented data effectively eliminate the concern that H_2O_2 confounds the proteomic data. However, the methodology for the H_2O_2 differential proteomic analysis is not described in the Methods section. This should be corrected.

Response: We thank the reviewer for this helpful comment and careful reminder. We have now added a new section in the Methods titled "H_2O_2-induced differential proteomic analysis" to provide the detailed methodology for this experiment. The relevant details can be found in revised manuscript **(text lines 780-790)**.

3. Connecting cisplatin exposure in early-stage oocytes to later meiotic defects: The authors have added considerable new experimental data showing in vivo treatment of neonatal ovaries with cisplatin ± GSK126. However, these data still do not support their statement that cisplatin treatment in early-stage oocytes leads to later meiotic defects (p. 20, lines 470–474). First, treatment with cisplatin ± GSK126 at PD14 followed by FGO collection 7 days later is too short to capture damage originating in primordial or primary oocytes, as it takes considerably longer than 7 days for these to develop into FGOs. This experiment effectively shows the consequences of cisplatin-induced toxicity on secondary follicle oocytes already present in the PD21 ovary. The rebuttal explains that PD7-treated mice did not survive to adulthood, precluding direct long-term follow-up of primordial follicles—this is an important practical limitation that should be explicitly stated in the manuscript. Whether primordial follicles that survive low-dose cisplatin exposure at PD7 eventually develop meiotic competence defects remains undemonstrated. The authors should clearly acknowledge in the Discussion that the maturation defect evidence derives from treating growing oocytes (PD14 ovary treatment and in vitro FGO), not from tracing the fate of cisplatin-exposed primordial follicles. The current text at times implies this connection without stating the limitation. For instance, the Discussion states that "EZH2 was overexpressed in oocytes and led to enhanced H3K27me3 levels after cisplatin treatment, accompanied by meiotic maturation defects" (p. 21, lines 493–500) without specifying which experimental paradigm supports each part of this claim. Moreover, this new set of experiments raises the question of whether primordial follicles treated with low-dose cisplatin survive beyond 7 days when co-treated

with GSK126. Figure 6c shows the percentage of abnormal primordial follicles in GSK126-treated ovaries, but the authors should also provide absolute follicle numbers to demonstrate whether GSK126 treatment prevents follicle loss, not merely delays morphological abnormalities.

Response: We thank the reviewer for this insightful comment. We agree that some statements in the previous version of the manuscript over-interpreted the relationship between cisplatin exposure in early-stage oocytes and later meiotic defects. We have revised these statements in the Results and Discussion sections to more accurately reflect the experimental evidence **(text lines 457-459, 469-471, 493-497, and 517-530)**. Specifically, regarding the experiment "treatment with cisplatin ± GSK126 at PD14 followed by FGO collection 7 days later," the purpose was previously mischaracterized. We have clarified it as follows: "We next examined the functional consequences of cisplatin exposure at the growing oocyte stage, focusing on oocytes from secondary follicles. For this purpose, PD14 mice were treated with cisplatin, with or without GSK126, and FGOs were collected seven days later." **(text lines 469-472)**. Furthermore, we have explicitly discussed in the revised manuscript the current limitations in directly tracing the long-term impact of cisplatin exposure on PD7 primordial follicles and their subsequent development into FGOs **(text lines 526-530)**. We have also revised the specific Discussion statement highlighted by the reviewer to clarify the supporting experimental paradigms. We now state that EZH2 upregulation and H3K27me3 accumulation were observed in primordial follicle oocytes after PD7 cisplatin treatment, whereas meiotic incompetence was observed in *in vitro* maturation of oocytes retrieved after PD14 ovary treatment and subjected to direct cisplatin incubation, rather than by directly tracing cisplatin-exposed primordial follicles **(text lines 517-526)**. To address the reviewer's request for absolute follicle numbers, we have now added quantification in Supplementary Figs. 7a–b. This shows a sharp decrease in the primordial follicle population in the cisplatin-treated group. Although primordial and total follicle numbers also decreased in the cisplatin + GSK126 group, the reduction was less severe than in the cisplatin-only group. These results confirm that GSK126 partially protects

primordial follicles from cisplatin-induced loss **(Supplementary Figs. 7b-c, text lines 467–469 in revised manuscript; Also see below)**.

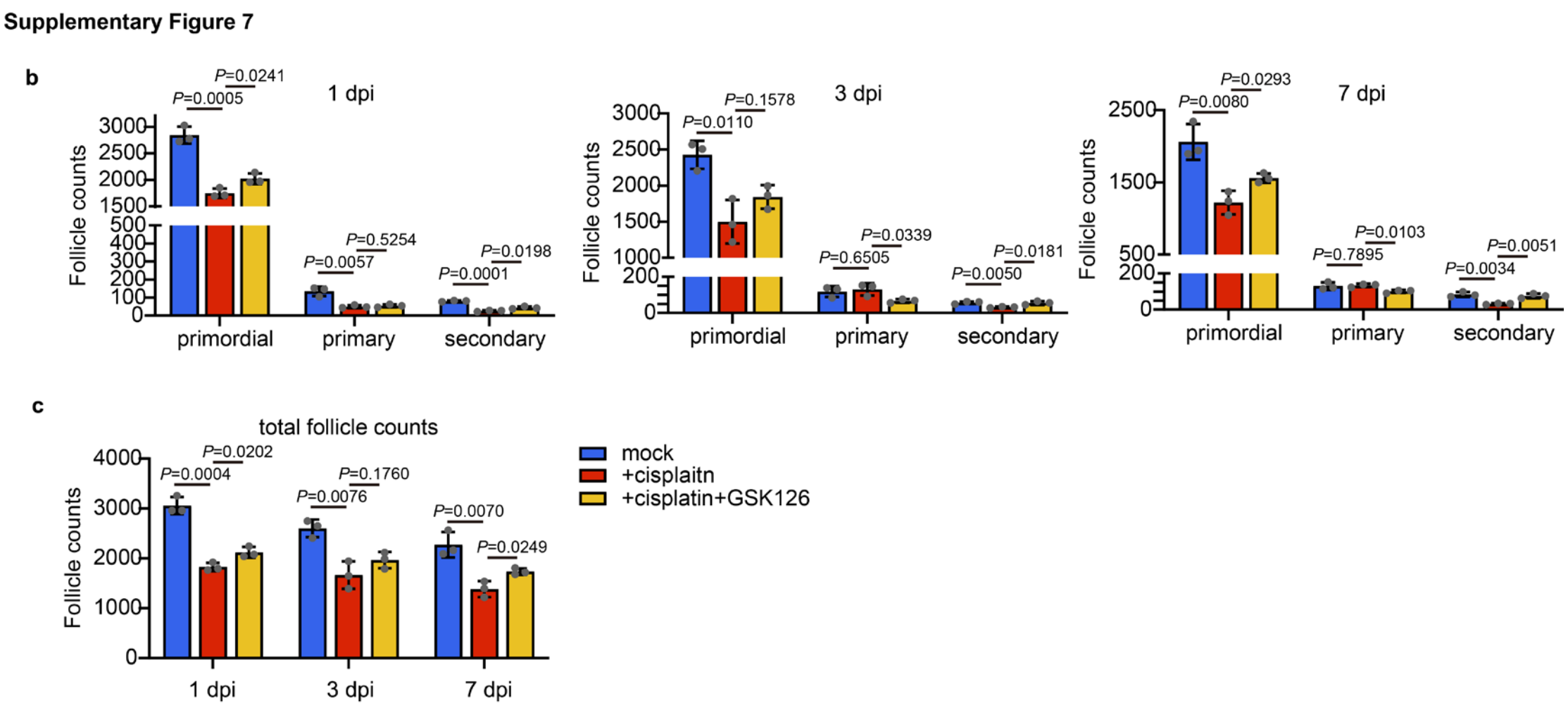

Supplementary Figs. 7b–c Quantification of primordial, primary, secondary (b), and total follicle numbers (c) in mock, cisplatin-treated, and cisplatin + GSK126-treated ovaries at 1, 3, and 7 dpi. Mean ± SEM. n = 3 for each group. *P* values are calculated by unpaired two-tailed one-way ANOVA.

4. Role of EZH2 in DNA repair versus epigenetic regulation: Based on changes in H3K27me3 levels, the authors suggest that cisplatin induces early EZH2-mediated epigenetic changes that lead to later meiotic defects. However, the authors neglect that H3K27me3 is also recruited to DNA damage sites as part of the DNA repair process (e.g., PMID: 31395347). The accumulation of H3K27me3 following cisplatin treatment may therefore reflect a direct role of EZH2/PRC2 in the DNA damage response rather than—or in addition to—epigenetic reprogramming. The dual role of EZH2 in epigenetic regulation versus DNA repair should be discussed, as this distinction has important implications for interpreting whether GSK126 is mitigating epigenetic damage or interfering with DNA repair at damage sites.

Response: We thank the reviewer for this important and insightful comment. We agree that H3K27me3 should not be interpreted solely as an epigenetic reprogramming mark in the context of cisplatin-induced DNA damage. As noted by the reviewer and previous

studies, H3K27me3 and EZH2/PRC2 can be recruited to DNA damage sites and participate in DNA damage-associated chromatin responses, including transcriptional repression and repair-associated chromatin regulation. Therefore, the increased H3K27me3 observed after cisplatin treatment may reflect a DNA damage-associated chromatin response, broader epigenetic dysregulation, or both. We have revised the manuscript to discuss this dual role of EZH2/H3K27me3 more explicitly. We also clarified that our current data cannot directly distinguish between these mechanisms. Future genome-wide profiling of H3K27me3 together with DNA damage markers, such as by ChIP-seq or CUT&Tag, in oocytes from different treatment groups may help resolve this question. In our experiments, GSK126 did not aggravate the damage phenotypes but instead improved follicle survival and meiotic progression, suggesting that EZH2 inhibition does not appear to severely compromise essential repair processes under our experimental conditions. The protective effect of GSK126 further suggests that excessive or sustained EZH2 activity / H3K27me3 abundance may be detrimental in cisplatin-induced oocyte injury. We speculate that GSK126 may protect oocytes by limiting excessive EZH2/H3K27me3-mediated chromatin repression after cisplatin-induced DNA damage, thereby preserving a chromatin state more compatible with follicle survival and oocyte maturation. However, the precise protective mechanism of GSK126 in this context remains to be further investigated. This discussion has been added to the revised manuscript **(text lines 533-550)**.

5. Internal contradiction regarding DNA damage after low-dose cisplatin: The authors' conclusions about DNA damage following low-dose cisplatin (2 mg/kg, 4 h) contain an internal contradiction that undermines one of the manuscript's central claims. On one hand, the text states that γH2AX staining showed "the higher, but not the lower, dose induced marked DNA double-strand breaks" (p. 16, lines 381–386), framing the low dose as causing no overt DNA damage by conventional assessment. On the other hand, the authors demonstrate that the same low-dose cisplatin induces ATM nuclear foci, RAD51 and RPA2 nuclear

accumulation, and XRCC1 upregulation in primordial follicle oocytes—all of which are direct, canonical markers of active DSB repair. These proteins cannot accumulate in the absence of DSBs. Therefore, the APEX-detected proteomic changes do not reveal a hidden molecular response invisible to conventional methods, as the authors claim, but rather a standard DNA damage response that should have been assessed alongside γH2AX with appropriate sensitivity. The authors should acknowledge that low-dose cisplatin does induce DSBs and reframe the narrative around what APEX proteomics reveals beyond what immunostaining already shows.

Response: We thank the reviewer for pointing out this important issue. Our previous wording could be interpreted as suggesting that low-dose cisplatin did not induce DNA damage, which was not our intention. We have revised the manuscript to acknowledge that low-dose cisplatin does induce DSB-associated responses, although to a lesser extent than the higher dose and without overt follicular morphological abnormalities **(text lines 397-399)**. We also agree that the DDR proteins detected by APEX proteomics, including ATM, RAD51, RPA2, and XRCC1, represent canonical DNA damage responses rather than a completely hidden molecular response. We have therefore reframed the narrative accordingly. The rationale for using low-dose cisplatin was to avoid overwhelming cell death-associated changes caused by high-dose treatment, which could obscure other early molecular responses. Under this low-dose condition, APEX-based proteomic analysis captured not only canonical DDR components consistent with low-level DSBs, but also broader molecular alterations, including stress-related pathways, metabolic changes, and chromatin regulatory machinery, with EZH2/H3K27me3 as a representative example. We have revised the Results sections to clarify this point **(text lines 399-403, 410-414, and 434-439)**. This revised wording acknowledges that low-dose cisplatin induces DNA damage while emphasizing that APEX proteomics provides a systematic view of the early molecular response beyond individual immunostaining markers.

6. 3D imaging novelty: The revised Discussion now appropriately cites prior 3D ovarian imaging studies and repositions the 3D visualization as a complementary feature rather than the primary innovation. The advantages (antibody-free, rapid, compatible with Cre-driver lines) and limitations (requires genetically engineered mice) are clearly stated. However, the authors still overstate the novelty of their 3D imaging workflow. The same results could be achieved with other Cre-driver lines crossed to any fluorescent reporter (e.g., a TdTomato-expressing Ai9 line) combined with DNA staining to visualize granulosa cell nuclei. The text should more accurately reflect this aspect of APEX mouse model.

Response: We thank the reviewer for this helpful comment. We have revised the Discussion to clarify that similar 3D visualization could also be achieved using other Cre-dependent LSL fluorescent reporter mice, such as LSL-tdTomato/Ai9 lines. Accordingly, we have revised the text to avoid overstating the novelty of the 3D imaging aspect. These changes have been incorporated into the revised Discussion section **(text lines 561-568)**.

7. Inconsistent fold-change thresholds and statistical reporting: The revised manuscript contains significant inconsistencies in fold-change thresholds and notation across analyses that must be resolved. (i) For the proteome: the volcano plot axis is labeled $\log_2$(fold change), and the text states "FC > 1.5, P < 0.05," which appears to mean linear fold change > 1.5. The rebuttal to Reviewer 1 confirms this. However, the original submission used $\log_2$FC > 1 (equivalent to linear FC > 2)—an entirely different threshold. The switch is not discussed. (ii) For the transcriptome: the Methods section indicates FC > 2 and adjusted P-value < 0.05, yet the Results text states "FC > 1.5." With FC > 2, there are 1,137 DEGs; with FC > 1.5, approximately 2,002—neither matches the 2,878 reported. (iii) For cisplatin-responsive transcriptomics: $\log_2$FC > 1 is used (equivalent to FC > 2), inconsistent with FC > 1.5 used for the baseline transcriptome. (iv) For cisplatin proteomics: upregulated proteins are reported with "FC > 1.5" and

downregulated with "FC < 0.67," which is the reciprocal—consistent with linear FC—but the original text used log_2FC. The authors must standardize all thresholds, use consistent notation (always specifying whether FC is linear or log_2-transformed), report adjusted P-values where applicable, and ensure that all reported numbers match the supplementary data tables. This is essential for reproducibility.

Response: We thank the reviewer for pointing out these important inconsistencies. We agree that the fold-change thresholds and statistical descriptions should be clearly defined and consistently reported throughout the manuscript. Both 1.5-fold and 2-fold changes are commonly used thresholds in proteomic and transcriptomic analyses, and the choice of threshold often depends on the characteristics of the dataset. In the original submission, we used FC > 2 as the threshold for both proteomic and transcriptomic analyses. During revision and re-analysis of the datasets, we found that several oocyte markers and functionally relevant proteins/genes showed enrichment between 1.5- and 2-fold. We therefore adopted a linear FC > 1.5 threshold to better capture biologically relevant oocyte-associated proteins and genes. In the revised manuscript, the Results text and figure legends have been standardized to use linear FC > 1.5. For volcano plots, the x-axis is labeled as log_2(FC) because the fold-change values are log-transformed for visualization, but the threshold described in the text refers to linear FC. For the transcriptomic analysis, the previous Methods section incorrectly retained the original criterion of FC > 2 and adjusted P-value < 0.05. We have now corrected this to linear FC > 1.5 and $P < 0.05$ **(text lines 853-855)**, consistent with the Results section and supplementary tables. In addition to the 1,948 genes identified by FC > 1.5 and $P <$ 0.05, 930 genes detected only in the APEX group were also considered APEX-enriched genes. Together, these two categories account for the 2,878 genes reported in the manuscript. This classification has now been clarified in the Methods section and annotated in the corresponding supplementary table **(Supplementary Data 3, text lines 853-855 in revised manuscript)**. For the cisplatin-responsive transcriptomic analysis, we have also standardized the threshold to linear FC > 1.5 and FC < 0.67, and the relevant Results text and figures have been revised accordingly **(Supplementary Data**

6, Supplementary Fig. 6i, text lines 441–443 in revised manuscript; Also see below). For the cisplatin-responsive proteomic analysis, the threshold had indeed already been standardized in the previous revision from FC > 2 / FC < 0.5 to linear FC > 1.5 for upregulated proteins and FC < 0.67 for downregulated proteins. We have systematically checked the manuscript, figure legends, and supplementary tables to ensure that all thresholds, notations, and reported numbers are now consistent.

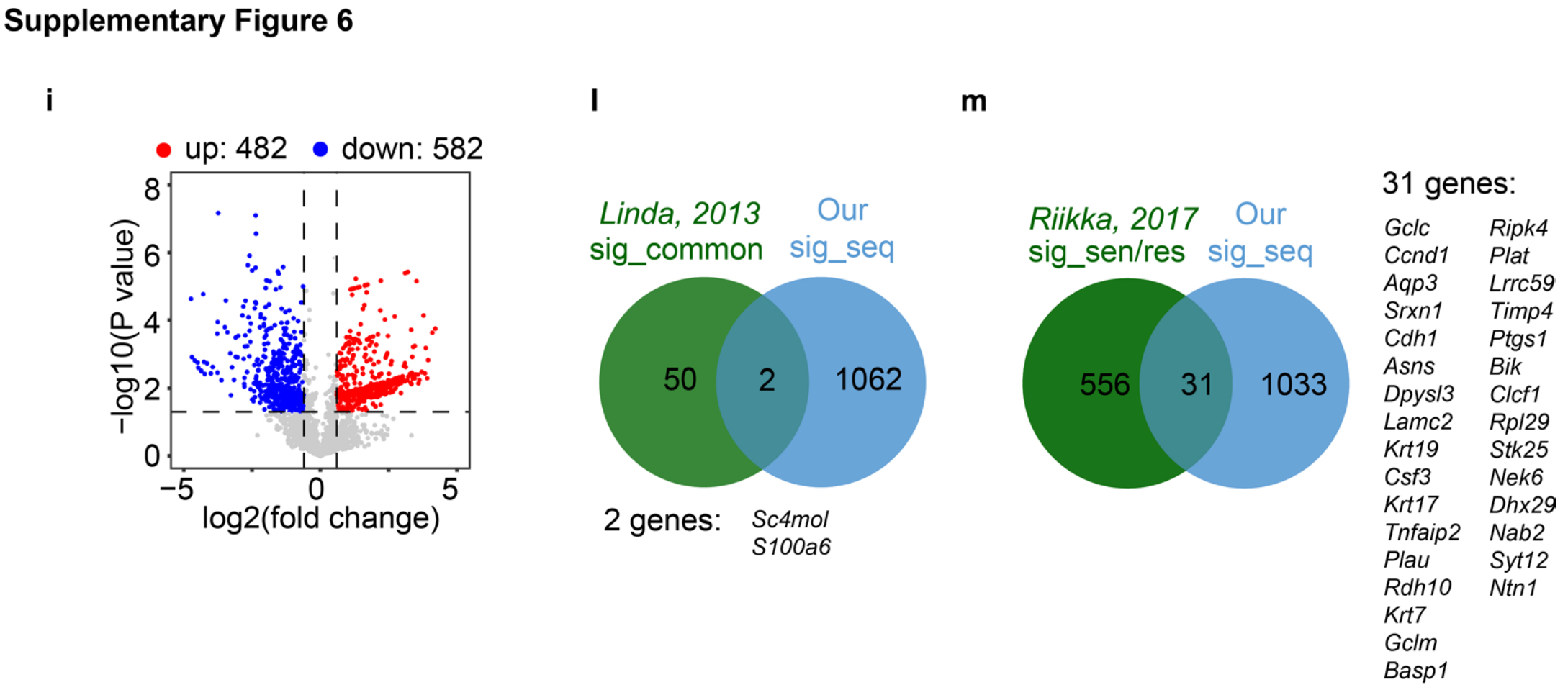

Supplementary Figure 6i, l-m. i Volcano plot of differentially expressed genes after low-dose cisplatin treatment detected in the transcriptome (upregulated in red, downregulated in blue). **l-m** Comparison of cisplatin-induced mRNA changes in primordial follicle oocytes identified in this study with those previously reported in tumor cells.

8. Figure cross-references: Several figure cross-references in the revised text appear incorrect. These should be systematically checked and corrected throughout the manuscript.

Response: We thank the reviewer for pointing this out. We have systematically checked all figure and supplementary figure cross-references throughout the revised manuscript and corrected the incorrect citations where necessary.

Reviewer #3:

The authors have provided a very thorough response to reviewers and have clearly addressed all concerns raised by the reviewers. The clarity and rigor of the manuscript are much improved, and I appreciate the authors' efforts to discuss limitations of their study, to add acknowledgments of previous work in this area, and to soften their conclusions where needed. I have no further concerns.
