# Supplementary material for: In vivo labeling–based proteomic analysis of early follicle oocytes and cisplatin-induced alterations in mice
Source: Nat Commun. 2026 Aug 15;17:9815. doi: 10.1038/s41467-026-76665-3 (PMC13574812; doi:10.1038/s41467-026-76665-3)
Supplement: Supplementary file 3 [file 41467_2026_76665_MOESM1_ESM.pdf]

SUPPLEMENTARY INFORMATION

In Vivo Labeling–Based Proteomic Analysis of Early Follicle Oocytes and Cisplatin-Induced Alterations in Mice

Zhi-Yan Jiang[1, 2, †], Meiyu Bi[2, 3, 4, †], Xuan Wu[1], Miaomiao Ban[2, 3, 4], Yuyao Lu[5], Jiangli Cao[5], Jie Li[2, 3, 4], Qilong Wang[2, 3, 4], Xin Zhu[2, 3, 4], Mo Li[2, 3, 4, 8, *], Heng-Yu Fan[1, 6, 7, *], Bingteng Xie[5, *]

[1] MOE Key Laboratory for Biosystems Homeostasis and Protection and Innovation Center for Cell Signaling Network, Life Sciences Institute, Zhejiang University, Hangzhou 310058, China

[2] National Clinical Research Center for Obstetrics and Gynecology, State Key Laboratory of Female Fertility Promotion, Department of Obstetrics and Gynecology, Peking University Third Hospital, Beijing 100191, China

[3] Key Laboratory of Assisted Reproduction (Peking University), Ministry of Education, Beijing 100191, China

[4] Beijing Key Laboratory of Reproductive Endocrinology and Assisted Reproductive Technology, Beijing 100191, China

[5] Key Laboratory of Molecular Medicine and Biological Diagnosis and Treatment (Ministry of Industry and Information Technology), School of Life Science, Beijing Institute of Technology, Beijing 100081, China

[6] Assisted Reproduction Unit, Department of Obstetrics and Gynecology, Sir Run Run Shaw Hospital, School of Medicine, Zhejiang University, Hangzhou 310016, China

[7] Center for Biomedical Research, Shaoxing Institute, Zhejiang University, Shaoxing 312000, China

[8] Lead contact

† These authors contributed equally to this work.

* Correspondence: limo@hsc.pku.edu.cn (M. L.), hyfan@zju.edu.cn (H. F.), and xiebingteng@bit.edu.cn (B. X.)

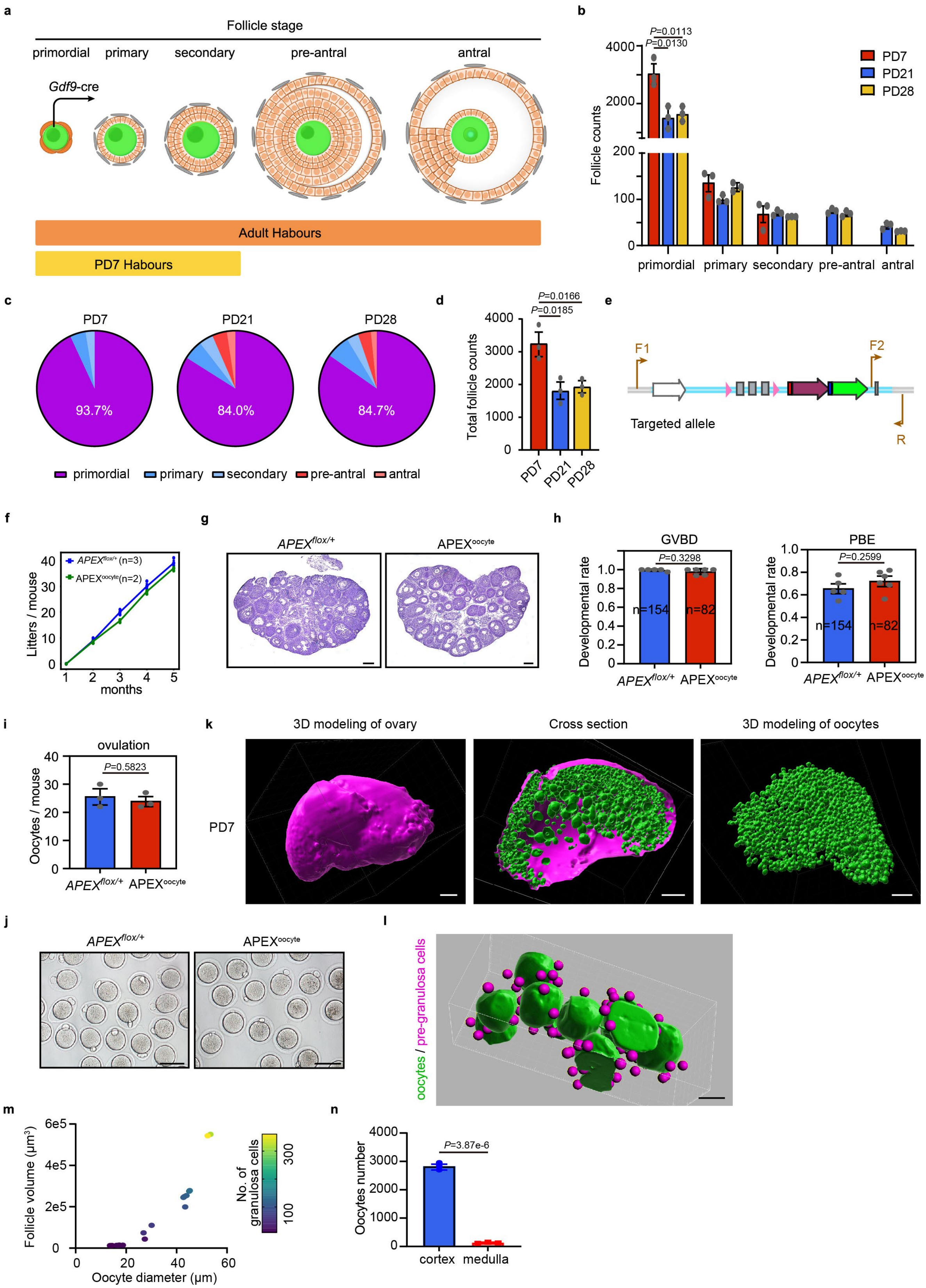

a
Follicle stage
primordial
primary
secondary
pre-antral
antral
Gdf9-cre
Adult Habours
PD7 Habours
b
P=0.0113
P=0.0130
Follicle counts
PD7
PD21
PD28
primordial
primary
secondary
pre-antral
antral
c
PD7
PD21
PD28
93.7%
84.0%
84.7%
primordial
primary
secondary
pre-antral
antral
d
P=0.0166
P=0.0185
Total follicle counts
PD7
PD21
PD28
e
F1
F2
Targeted allele
R
f
APEX flox/+ (n=3)
APEX oocyte (n=2)
Litters / mouse
months
g
APEX flox/+
APEX oocyte
h
GVBD
P=0.3298
Developmental rate
n=154
n=82
APEX flox/+
APEX oocyte
PBE
P=0.2599
i
ovulation
P=0.5823
Oocytes / mouse
APEX flox/+
APEX oocyte
k
3D modeling of ovary
Cross section
3D modeling of oocytes
PD7
j
APEX flox/+
APEX oocyte
l
oocytes / pre-granulosa cells
m
Follicle volume (μm³)
Oocyte diameter (μm)
No. of granulosa cells
n
P=3.87e-6
Oocytes number
cortex
medulla

Supplementary Figure 1. APEX^{oocyte} female mice presented normal fertility. a Illustration of follicle developmental stages and temporal window of *Gdf9*-Cre activity. Primordial follicles predominate around PD7, whereas all follicle stages are present in adult ovaries. PD, post-natal day. **b** Quantification of different staged follicles PD7, PD21, and PD28 mice. n = 3 biologically independent ovaries. Statistical significance was assessed using one-way ANOVA. **c** Proportional distribution of follicles at PD7, PD21, and PD28. **d** Total follicle counts at the indicated postnatal stages. n = 3 biologically independent ovaries. Statistical significance was assessed using one-way ANOVA. **e** PCR genotyping strategy for the targeted APEX allele. F1/R primers amplified the wild-type allele (+) and F2/R primers amplified the transgenic APEX2 (flox). F, forward. R, reverse. **f** Cumulative litter numbers per female over time for *APEX$^{flox/+}$* (n = 3 biologically independent females) and APEX^{oocyte} (n = 2) mice. **g** Representative ovarian histology from 3-week-old *APEX$^{flox/+}$* and APEX^{oocyte} females. Scale bar, 100 µm. **h** Germinal vesicle breakdown (GVBD) and first polar body 1 emission (PBE) rates of *in vitro* cultured FGOs from both genotypes. n = analyzed oocytes, obtained from 5 and 6 independent experiments for the *APEX$^{flox/+}$* and APEX^{oocyte} groups, respectively. *P* values are calculated by unpaired two-tailed Student's t tests. **i** Number of ovulated oocytes per mouse (3-week-old) following hormonal stimulation. n = 3 biologically independent females. *P* values are calculated by unpaired two-tailed Student's t tests. **j** Representative bright-field images of MII oocytes from 3-week-old females. Scale bar, 100 µm. **k** 3D reconstruction of a PD7 ovary, cross section view, and surface rendering of EGFP-positive oocytes of the APEX^{oocyte} mouse. Magenta, ovarian surface; Green, oocytes. Scale bars, 100 µm. **l** 3D modeling of primordial follicles from APEX^{oocyte} females showing oocytes (green) and pre-granulosa cells (magenta). Scale bar, 10 µm. **m** Relationship among oocyte diameter, follicle volume, and granulosa cell number. Color indicates granulosa cell number per follicle. **n** Quantification of oocytes in the cortex and medulla of PD7 ovary. n = 3 biologically independent ovaries per group. *P* values are calculated by unpaired two-tailed Student's t tests.

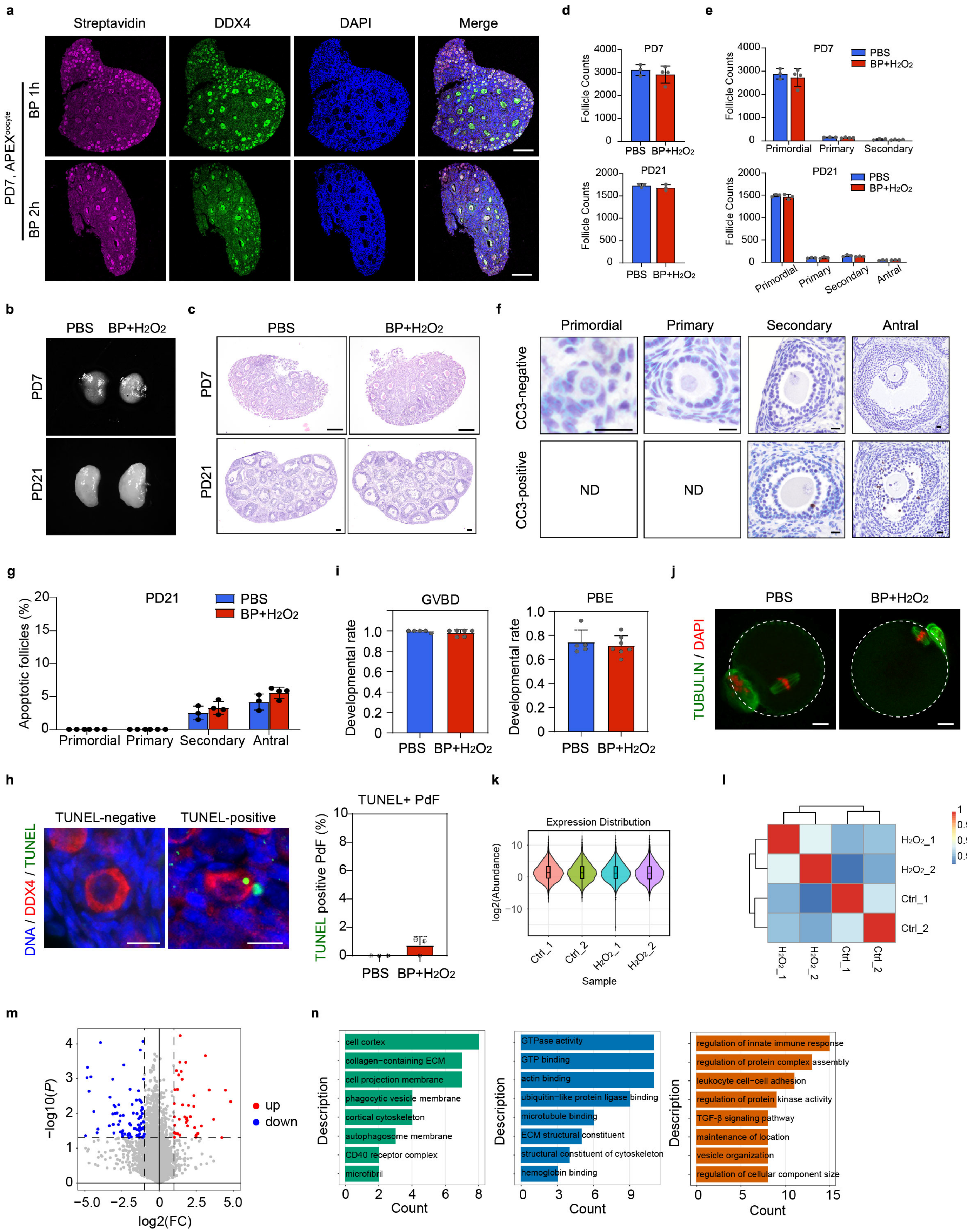
a
Streptavidin
DDX4
DAPI
Merge
PD7, APEX^oocyte
BP 1h
BP 2h
d
PD7
PD21
Follicle Counts
PBS
BP+H2O2
e
PD7
PD21
PBS
BP+H2O2
Primordial
Primary
Secondary
Antral
b
PBS
BP+H2O2
PD7
PD21
c
PBS
BP+H2O2
PD7
PD21
f
Primordial
Primary
Secondary
Antral
CC3-negative
CC3-positive
ND
ND
g
PD21
Apoptotic follicles (%)
Primordial
Primary
Secondary
Antral
i
GVBD
PBE
Developmental rate
PBS
BP+H2O2
j
PBS
BP+H2O2
TUBULIN / DAPI
h
TUNEL-negative
TUNEL-positive
DNA / DDX4 / TUNEL
TUNEL+ PdF
TUNEL positive PdF (%)
k
Expression Distribution
log2(Abundance)
Ctrl_1
Ctrl_2
H2O2_1
H2O2_2
Sample
l
H2O2_1
H2O2_2
Ctrl_1
Ctrl_2
m
−log10(P)
log2(FC)
up
down
n
Description
Count
cell cortex
collagen−containing ECM
cell projection membrane
phagocytic vesicle membrane
cortical cytoskeleton
autophagosome membrane
CD40 receptor complex
microfibril
GTPase activity
GTP binding
actin binding
ubiquitin−like protein ligase binding
microtubule binding
ECM structural constituent
structural constituent of cytoskeleton
hemoglobin binding
regulation of innate immune response
regulation of protein complex assembly
leukocyte cell−cell adhesion
regulation of protein kinase activity
TGF-β signaling pathway
maintenance of location
vesicle organization
regulation of cellular component size

Supplementary Figure 2. Evaluation of *in vivo* APEX labeling conditions and their effects on ovarian physiology. a Representative ovarian sections from PD7 $APEX^{oocyte}$ mice after *in vivo* APEX labeling with 1 or 2 h of BP incubation. Sections were stained with streptavidin, DDX4, and DAPI. Scale bars, 100 μm. **b** Gross morphology of PD7 and PD21 ovaries from PBS- or BP+H_2O_2-treated mice. **c** H&E-stained ovarian sections showing comparable follicular architecture between groups at PD7 and PD21. Scale bars, 100 μm. **d–e** Quantification of total (**d**) and stage-specific (**e**) follicles at PD7 (top) and PD21 (bottom). Biologically independent mice at PD7 (n = 3, 4) and PD21 (n = 3, 3) for PBS and BP + H_2O_2 were analyzed. **f** Representative cleaved caspase-3 (CC3) staining of follicles at PD21. ND, not detected. Scale bars, 20 μm. **g** Quantification of $CC3^+$ apoptotic follicles at PD21. Biologically independent ovaries were analyzed at PD21 (n = 3, 4) for PBS and BP + H_2O_2, respectively. **h** TUNEL staining of primordial follicles at PD7 showing representative $TUNEL^-$ and $TUNEL^+$ examples (left) and quantification of $TUNEL^+$ primordial follicles per ovary (right). Scale bars, 20 μm. n = 3 biologically independent ovaries. **i** *In vitro* maturation of FGOs collected at PD21 following *in vivo* labeling. Biologically independent female mice were examined GVBD (n = 5, 6) and PBE (n = 5, 7) for PBS and BP + H_2O_2. **j** Representative spindle morphology of *in vitro* matured oocytes stained for α-tubulin (green) and DNA (red). Scale bars, 20 μm. **k** Distribution of ovarian protein abundance in control (Ctrl) and H_2O_2-treated mice across biological replicates. Center line, median; box limits, upper and lower quartiles; whiskers, 1.5× interquartile range; points, outliers. **l** Correlation heatmap of total protein abundance between Ctrl and H_2O_2-treated groups. **m** Volcano plot of differentially expressed proteins between H_2O_2-treated and control groups. Red and blue dots indicate upregulated and downregulated proteins, respectively. *P* values were calculated using a two-sided unpaired Student's t-test, without adjustment for multiple comparisons. **n** Gene Ontology (GO) enrichment analysis of differentially expressed proteins. Cellular component, molecular function, and biological process terms are shown in green, blue, and orange, respectively. All the IHC and IF results presented here are representative of three independent experiments.

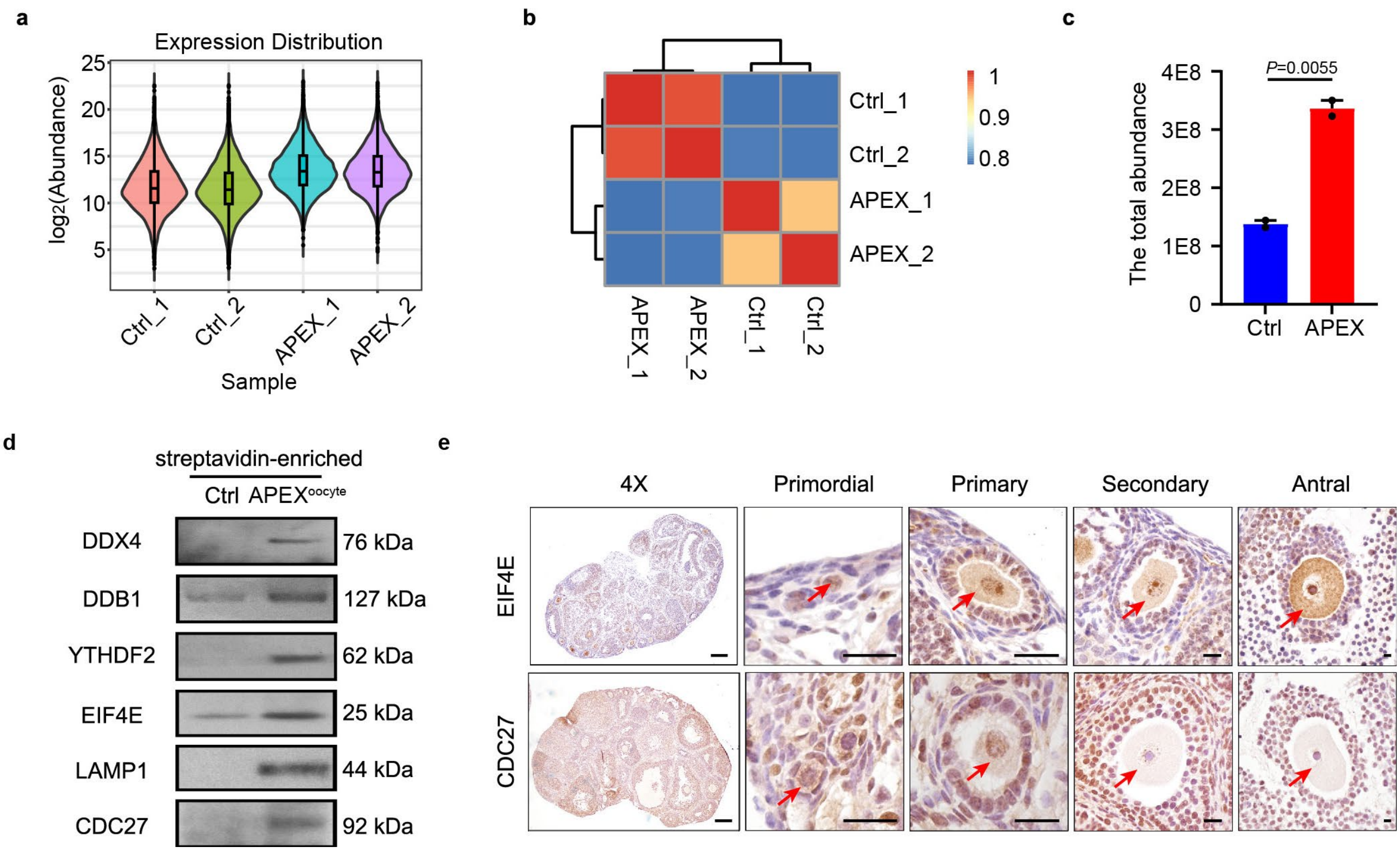

Supplementary Figure 3. Quality assessment and validation of the streptavidin-enriched oocyte proteome. a Distribution of protein abundance across samples shown as $\log_2$-transformed intensity values. Center line, median; box limits, upper and lower quartiles; whiskers, 1.5× interquartile range; points, outliers. **b** Sample correlation heatmap based on protein abundance profiles. **c** Total protein abundance detected in control and APEX samples. Mean ± SEM. n = 2 biological repeats for each group. *P* values are calculated by unpaired two-tailed Student's t tests. **d** Western blot analysis of representative proteins in streptavidin-enriched fractions from control (Ctrl) and APEX^{oocyte} ovaries. **e** Immunohistochemical staining of eukaryotic translation initiation factor 4E (EIF4E) and cell division cycle 27 (CDC27) in PD21 ovarian sections. Images include low-magnification overview and higher-magnification views of primordial, primary, secondary, and antral follicles. Arrows indicate oocytes. Scale bars, 100 μm in 4× microscopy and 20 μm in enlarged views. All the IHC results presented here are representative of three independent experiments.

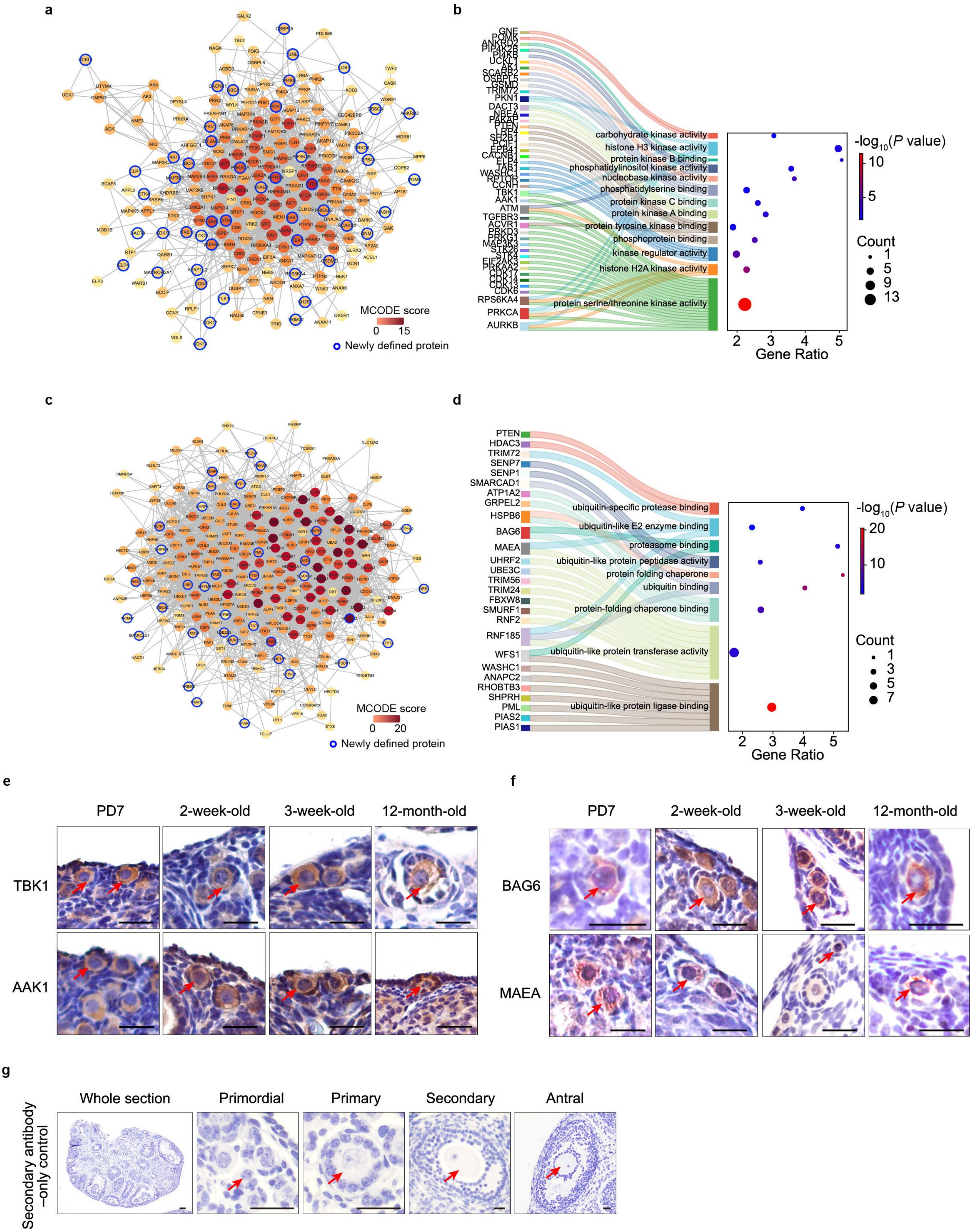
a
MCODE score
0 15
Newly defined protein
b
GNE
POMK
ANKRD2
PIP4K2B
PI4KB
UCKL1
AK1
SCARB2
OSBPL5
GSMD
TRIM72
PKN1
DACT3
NBEA
PAK2P
PTEN
LRP4
SH2B1
PCIF1
EPB41
CACNB1
ELP4
TAB1
WASHC1
RPTOR
CCNH
TBK1
AAK1
ATM
TGFBR3
ACVR1
PRKD3
PRKD2
MAP3K3
STK26
EIF2AK3
PRKAA2
CDK17
CDK14
CDK13
CDK6
RPS6KA4
PRKCA
AURKB
carbohydrate kinase activity
histone H3 kinase activity
protein kinase B binding
phosphatidylinositol kinase activity
nucleobase kinase activity
phosphatidylserine binding
protein kinase C binding
protein kinase A binding
protein tyrosine kinase binding
phosphoprotein binding
kinase regulator activity
histone H2A kinase activity
protein serine/threonine kinase activity
-log10(P value)
10
5
Count
1
5
9
13
2 3 4 5
Gene Ratio
c
MCODE score
0 20
Newly defined protein
d
PTEN
HDAC3
TRIM72
SENP7
SENP1
SMARCAD1
ATP1A2
GRPEL2
HSPB6
BAG6
MAEA
UHRF2
UBE3C
TRIM56
TRIM24
FBXW8
SMURF1
RNF2
RNF185
WFS1
WASHC1
ANAPC2
RHOBTB3
SHPRH
PML
PIAS2
PIAS1
ubiquitin-specific protease binding
ubiquitin-like E2 enzyme binding
proteasome binding
ubiquitin-like protein peptidase activity
protein folding chaperone
ubiquitin binding
protein-folding chaperone binding
ubiquitin-like protein transferase activity
ubiquitin-like protein ligase binding
-log10(P value)
20
10
Count
1
3
5
7
2 3 4 5
Gene Ratio
e
PD7
2-week-old
3-week-old
12-month-old
TBK1
AAK1
f
PD7
2-week-old
3-week-old
12-month-old
BAG6
MAEA
g
Whole section
Primordial
Primary
Secondary
Antral
Secondary antibody
–only control

Supplementary Figure 4. Interaction networks and age-independent expression of regulatory proteins in oocytes. a Protein–protein interaction network of kinase-related regulatory proteins identified in the oocyte proteome. Nodes represent proteins, with newly defined proteins indicated by bold-bordered circles. Node color intensity reflects MCODE score. **b** Functional enrichment analysis of proteins in the kinase-associated network. The Sankey diagram links newly defined proteins (left) to enriched molecular function categories (right). The size and color of the bubbles on the right correspond to the gene number (Count) and $-\log_{10}(P$ value). Significance was assessed using a one-sided hypergeometric test with Benjamini–Hochberg correction for multiple comparisons. **c** Protein–protein interaction network of proteins associated with protein stability and ubiquitin–proteasome regulation. Newly defined proteins are indicated by bold-bordered circles. Node color intensity reflects MCODE score. **d** Functional enrichment analysis of proteins in the protein homeostasis network. The Sankey diagram links newly defined proteins (left) to enriched molecular function categories (right). The size and color of the bubbles on the right correspond to the gene number (Count) and $-\log_{10}(P$ value). Significance was assessed using a one-sided hypergeometric test with Benjamini–Hochberg correction for multiple comparisons. **e** Immunohistochemical staining of TANK binding kinase 1 (TBK1) and AP2 associated kinase 1 (AAK1) in ovarian sections from PD7, 2-week-old, 3-week-old, and 12-month-old mice. Arrows indicate primordial follicle oocytes. Scale bars, 20 μm. **f** Immunohistochemical staining of BAG cochaperone 6 (BAG6) and macrophage erythroblast attacher (MAEA) in ovarian sections from PD7, 2-week-old, 3-week-old, and 12-month-old mice. Arrows indicate primordial follicle oocytes. Scale bars, 20 μm. **g** Secondary antibody–only control staining across follicle stages in PD21 ovarian sections. Arrows indicate oocytes. Scale bars, 100 μm in 4× microscopy and 20 μm in enlarged views. All the IHC results presented here are representative of three independent experiments.

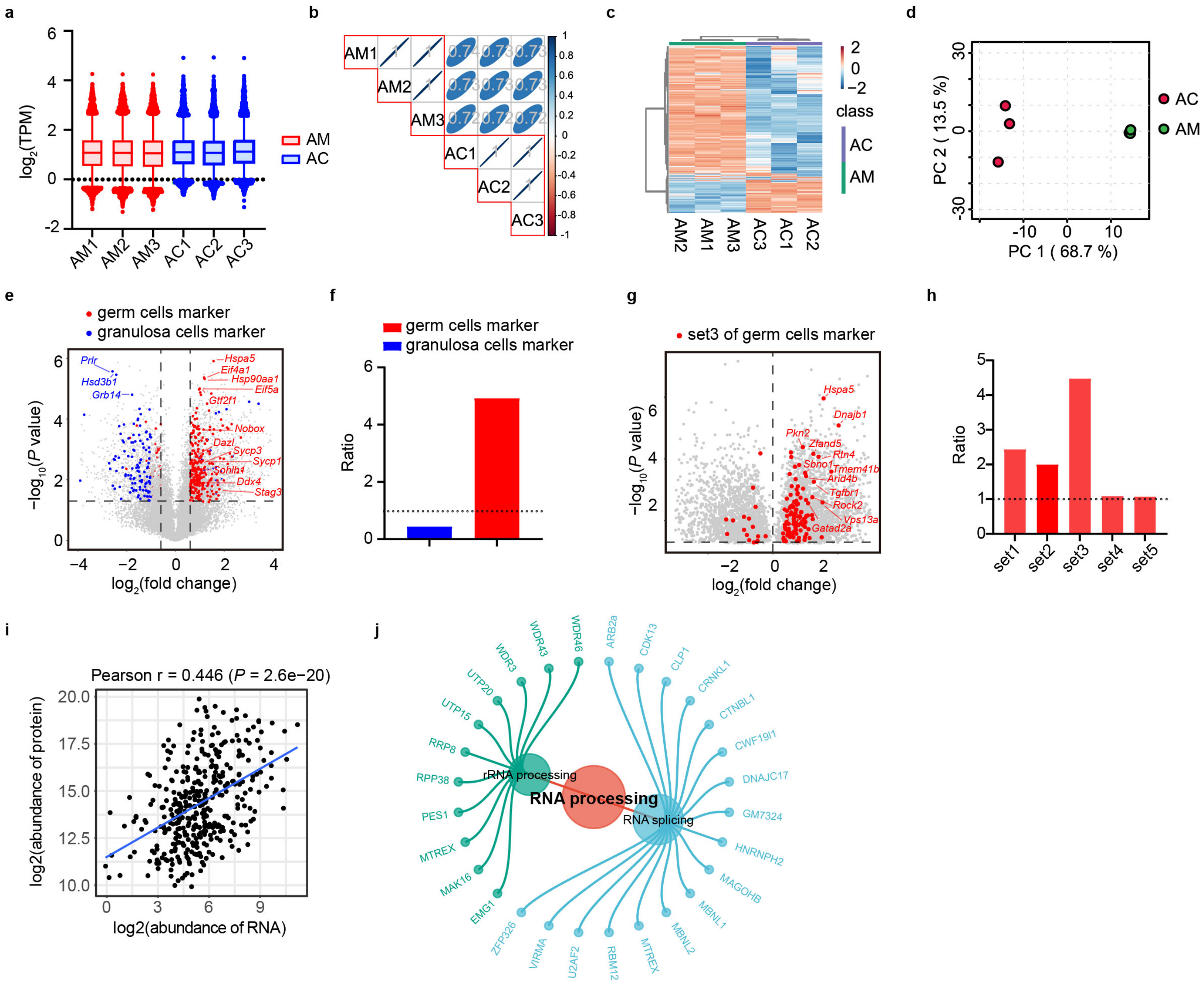

Supplementary Figure 5. Quality assessment and cellular specificity of the APEX-enriched oocyte transcriptome. a The box plot showed that transcripts abundance followed the normal distribution in all samples. AM, APEX^{oocyte} mice treated with PBS; AC, APEX^{oocyte} mice treated with cisplatin. Center line, median; box limits, upper and lower quartiles; whiskers, 1.5× interquartile range; points, outliers. **b** Correlation among the three replicate measurements using the TPM for each transcript of indicated groups. The correlation coefficient is calculated by Pearson's correlation. **c** Heat map of transcripts among different groups. Transcripts expressed in oocytes were presented with altered expression levels upon cisplatin treatment. **d** PCA of the indicated groups. The triplicates were well correlated within each group. **e** Volcano plot highlighting germ cell marker genes (red) and granulosa cell marker genes (blue) in the

comparison between APEX^{oocyte} and control transcriptomes. *P* values were calculated using a two-sided unpaired Student's t-test, without adjustment for multiple comparisons. **f** Ratio of germ cell marker genes and granulosa cell marker genes detected in the enriched transcriptome. **g** Volcano plot showing set 3 germ cell marker genes. *P* values were calculated using a two-sided unpaired Student's t-test, without adjustment for multiple comparisons. **h** Ratio of germ cell marker genes of gene sets (set1–set5) detected in the enriched transcriptome. **i** Correlation analysis between RNA abundance and protein abundance across shared genes. **j** Newly defined proteins of RNA processing and RNA splicing–related proteins identified in the oocyte proteome.

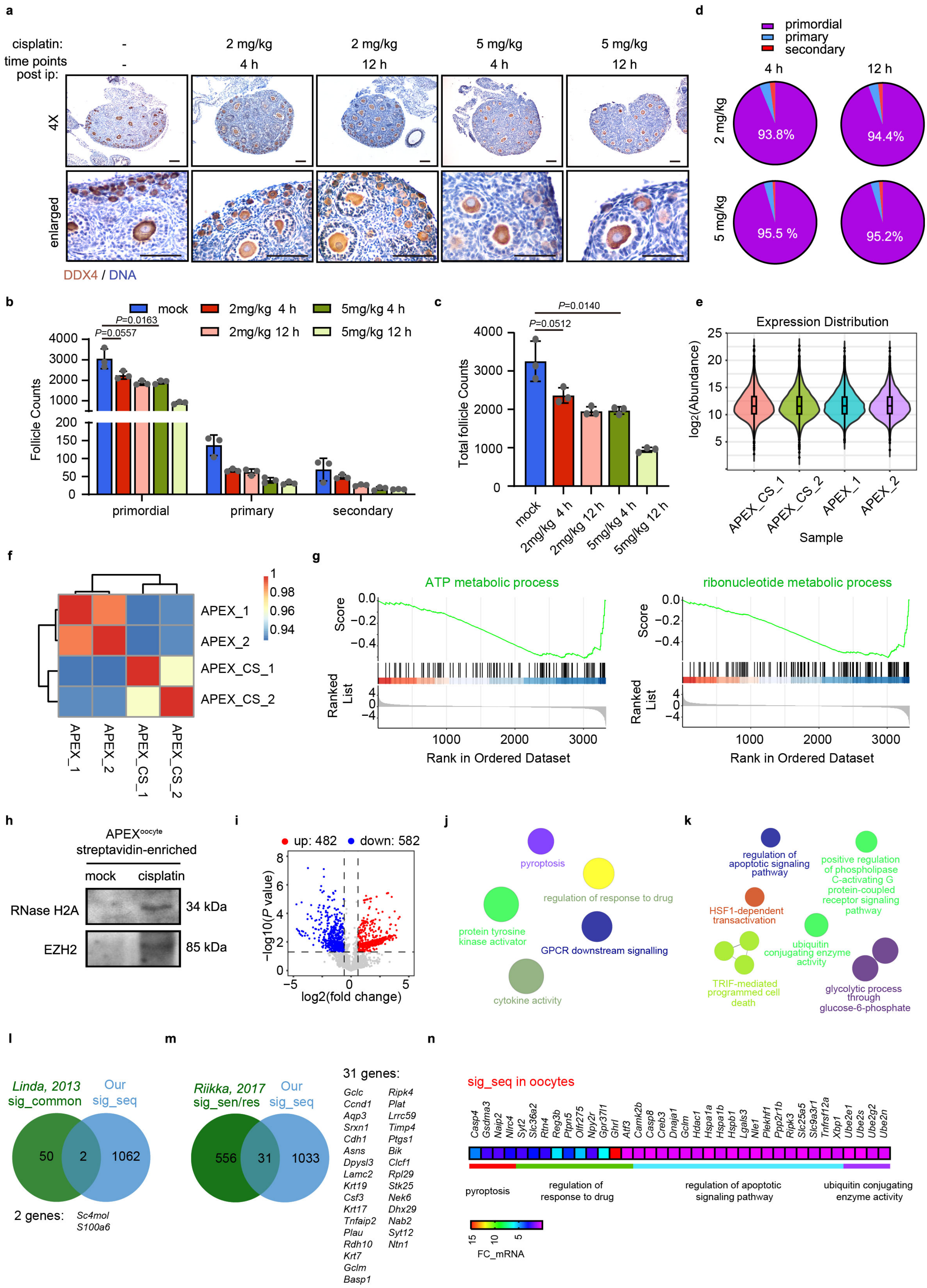

a
cisplatin: - 2 mg/kg 2 mg/kg 5 mg/kg 5 mg/kg
time points post ip: - 4 h 12 h 4 h 12 h
4X
enlarged
DDX4 / DNA
b
mock 2mg/kg 4 h 5mg/kg 4 h 2mg/kg 12 h 5mg/kg 12 h
P=0.0163
P=0.0557
Follicle Counts
primordial primary secondary
c
P=0.0140
P=0.0512
Total follicle Counts
mock 2mg/kg 4 h 2mg/kg 12 h 5mg/kg 4 h 5mg/kg 12 h
d
primordial
primary
secondary
4 h 12 h
2 mg/kg
5 mg/kg
93.8% 94.4% 95.5 % 95.2%
e
Expression Distribution
log2(Abundance)
APEX_CS_1 APEX_CS_2 APEX_1 APEX_2
Sample
f
APEX_1
APEX_2
APEX_CS_1
APEX_CS_2
g
ATP metabolic process
ribonucleotide metabolic process
Score
Ranked List
Rank in Ordered Dataset
h
APEX^oocyte streptavidin-enriched
mock cisplatin
RNase H2A 34 kDa
EZH2 85 kDa
i
up: 482 down: 582
-log10(P value)
log2(fold change)
j
pyroptosis
regulation of response to drug
protein tyrosine kinase activator
GPCR downstream signalling
cytokine activity
k
regulation of apoptotic signaling pathway
positive regulation of phospholipase C-activating G protein-coupled receptor signaling pathway
HSF1-dependent transactivation
ubiquitin conjugating enzyme activity
TRIF-mediated programmed cell death
glycolytic process through glucose-6-phosphate
l
Linda, 2013 sig_common
Our sig_seq
50 2 1062
2 genes: Sc4mol S100a6
m
Riikka, 2017 sig_sen/res
Our sig_seq
556 31 1033
31 genes:
Gclc Ccnd1 Aqp3 Srxn1 Cdh1 Asns Dpysl3 Lamc2 Krt19 Csf3 Krt17 Tnfaip2 Plau Rdh10 Krt7 Gclm Basp1
Ripk4 Plat Lrrc59 Timp4 Ptgs1 Bik Clcf1 Rpl29 Stk25 Nek6 Dhx29 Nab2 Syt12 Ntn1
n
sig_seq in oocytes
Casp4 Gsdma3 Naip2 Nlrc4 Syt2 Slc36a2 Rtn4 Reg3b Ptpn5 Olfr275 Npy2r Gpr37l1 Ghrl Atf3 Camk2b Casp8 Creb3 Dnaja1 Gclm Hdac1 Hspa1a Hspa1b Hspb1 Lgals3 Nle1 Plekhf1 Ppp2r1b Ripk3 Slc25a5 Slc9a3r1 Tnfrsf12a Xbp1 Ube2e1 Ube2s Ube2g2 Ube2n
pyroptosis
regulation of response to drug
regulation of apoptotic signaling pathway
ubiquitin conjugating enzyme activity
FC_mRNA

Supplementary Figure 6. Dose- and time-dependent ovarian effects of cisplatin and associated molecular alterations. a Representative PD7 ovarian sections (4× overview and enlarged views) stained for DDX4 (brown) and DNA (blue) after cisplatin treatment (2 or 5 mg/kg; 4 h or 12 h). Scale bar, 100 µm. **b** Quantification of PD7 ovarian follicle counts by stage (primordial, primary, secondary). Mean ± SEM. n = 3 biologically independent ovaries. Statistical significance was assessed using one-way ANOVA. **c** Total follicle counts under the indicated conditions from PD7 mice. Mean ± SEM. n = 3 biologically independent ovaries. Statistical significance was assessed using one-way ANOVA. **d** Pie charts of the proportion of primordial, primary and secondary follicles in PD7 ovaries after cisplatin treatment. Percentages of primordial follicles in each group were labeled in the pie. **e** Global protein abundance distribution across control (APEX) and cisplatin-treated (APEX_CS) samples, showing comparable overall intensity ranges. Center line, median; box limits, upper and lower quartiles; whiskers, 1.5× interquartile range; points, outliers. **f** Sample correlation heatmap demonstrating clustering of biological replicates. **g** Gene set enrichment analysis plots showing enrichment of "ATP metabolic process" and "ribonucleotide metabolic process" pathways that were downregulated by cisplatin treatment. **h** Streptavidin-enriched immunoblot validation of representative cisplatin-responsive proteins, including RNase H2A and EZH2. **i** Volcano plot of differentially expressed genes after low-dose cisplatin treatment detected in the transcriptome (upregulated in red, downregulated in blue). *P* values were calculated using a two-sided unpaired Student's t-test, without adjustment for multiple comparisons. **j–k** Enrichment analyses of upregulated (**j**) and downregulated (**k**) genes upon cisplatin treatment. **l-m** Comparison of cisplatin-induced mRNA changes in primordial follicle oocytes identified in this study with those previously reported in tumor cells. **n** Heatmap of representative significantly changed genes across functional categories including pyroptosis, regulation of response to drug, regulation of apoptotic signaling pathway, and ubiquitin conjugating enzyme activity.

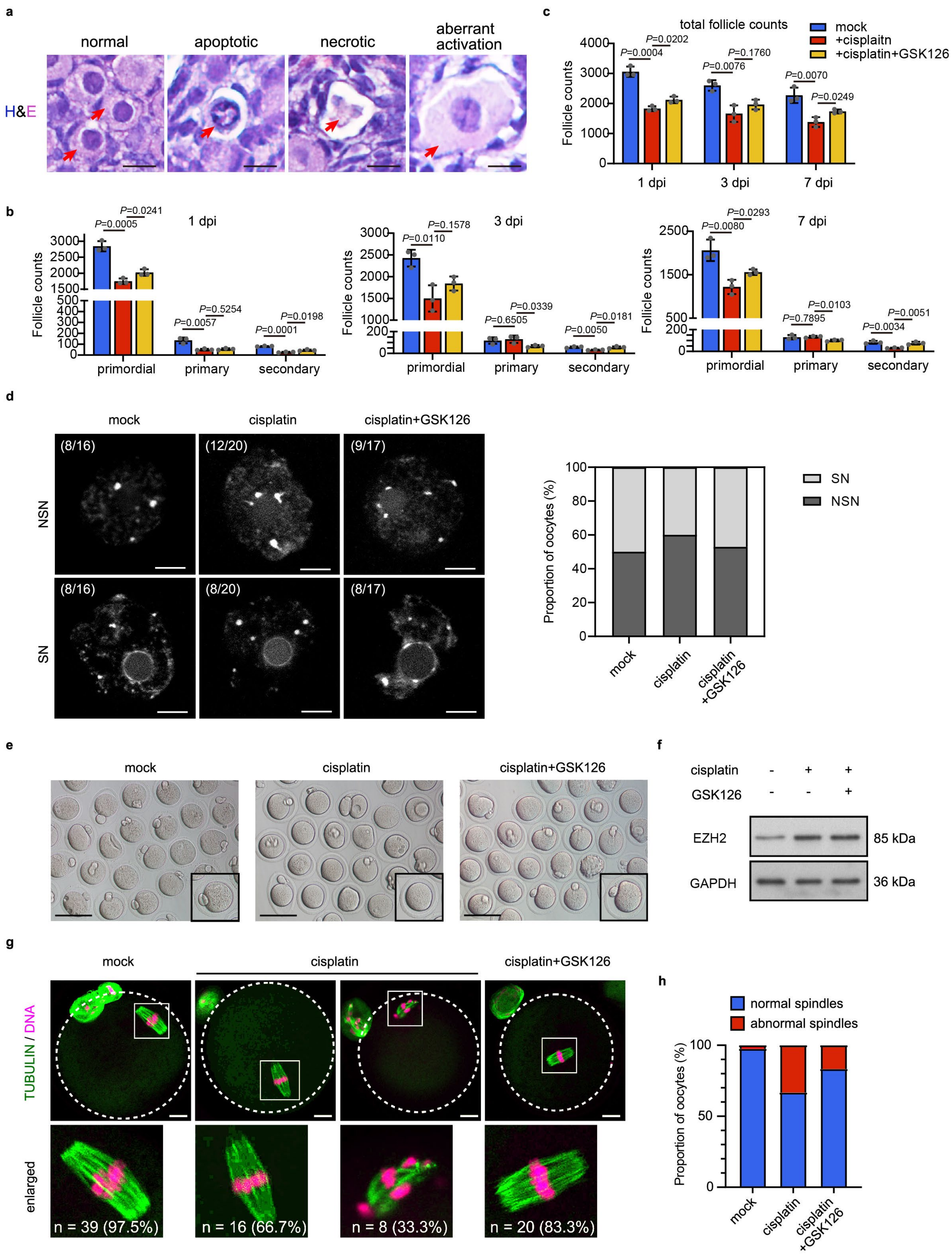
a
normal
apoptotic
necrotic
aberrant activation
H&E
c
total follicle counts
mock
+cisplaitn
+cisplatin+GSK126
Follicle counts
P=0.0202
P=0.0004
P=0.1760
P=0.0076
P=0.0070
P=0.0249
1 dpi
3 dpi
7 dpi
b
1 dpi
P=0.0241
P=0.0005
P=0.5254
P=0.0057
P=0.0198
P=0.0001
primordial
primary
secondary
3 dpi
P=0.1578
P=0.0110
P=0.0339
P=0.6505
P=0.0181
P=0.0050
7 dpi
P=0.0293
P=0.0080
P=0.0103
P=0.7895
P=0.0051
P=0.0034
d
mock
cisplatin
cisplatin+GSK126
NSN
SN
(8/16)
(12/20)
(9/17)
(8/16)
(8/20)
(8/17)
Proportion of oocytes (%)
SN
NSN
mock
cisplatin
cisplatin +GSK126
e
mock
cisplatin
cisplatin+GSK126
f
cisplatin - + +
GSK126 - - +
EZH2
85 kDa
GAPDH
36 kDa
g
mock
cisplatin
cisplatin+GSK126
TUBULIN / DNA
enlarged
n = 39 (97.5%)
n = 16 (66.7%)
n = 8 (33.3%)
n = 20 (83.3%)
h
normal spindles
abnormal spindles
Proportion of oocytes (%)
mock
cisplatin
cisplatin +GSK126

Supplementary Figure 7. Histological and meiotic phenotypes of oocytes after cisplatin exposure and rescue by EZH2 inhibition. a Representative hematoxylin and eosin (H&E) staining showing morphological features of normal, apoptotic, necrotic, and aberrantly activated primordial follicles (arrows indicate oocytes). Scale bars, 20 µm. **b–c** Quantification of primordial, primary, secondary (b), and total follicle numbers (c) in mock, cisplatin-treated, and cisplatin + GSK126-treated ovaries at 1, 3, and 7 dpi. Mean ± SEM. n = 3 biologically independent ovaries. Statistical significance was assessed using one-way ANOVA. **d** Representative FGOs showing non-surrounded nucleolus (NSN) and surrounded nucleolus (SN) chromatin configurations, collected from PD21 mice collected at 7 days after *in vivo* cisplatin treatment at PD14, with or without GSK126 co-treatment. Bar graph shows the proportion of SN and NSN oocytes. **e** Bright-field images of *in vitro*–matured oocytes under indicated treatments. Insets show representative oocytes. Scale bar, 100 µm. **f** Immunoblot analysis of EZH2 protein levels in oocytes after cisplatin and/or GSK126 treatment; GAPDH serves as loading control. **g** Immunofluorescence of spindle microtubules (tubulin, green) and DNA (magenta) in metaphase II oocytes with and without cisplatin and GSK126 treatment. Enlarged panels show spindle morphology. The number of spindles (n) and the proportion of oocytes (x%) were indicated. Scale bar, 20 µm. **h** Quantification of normal versus abnormal spindle organization among treatment groups. All the IHC and IF results presented here are representative of three independent experiments.

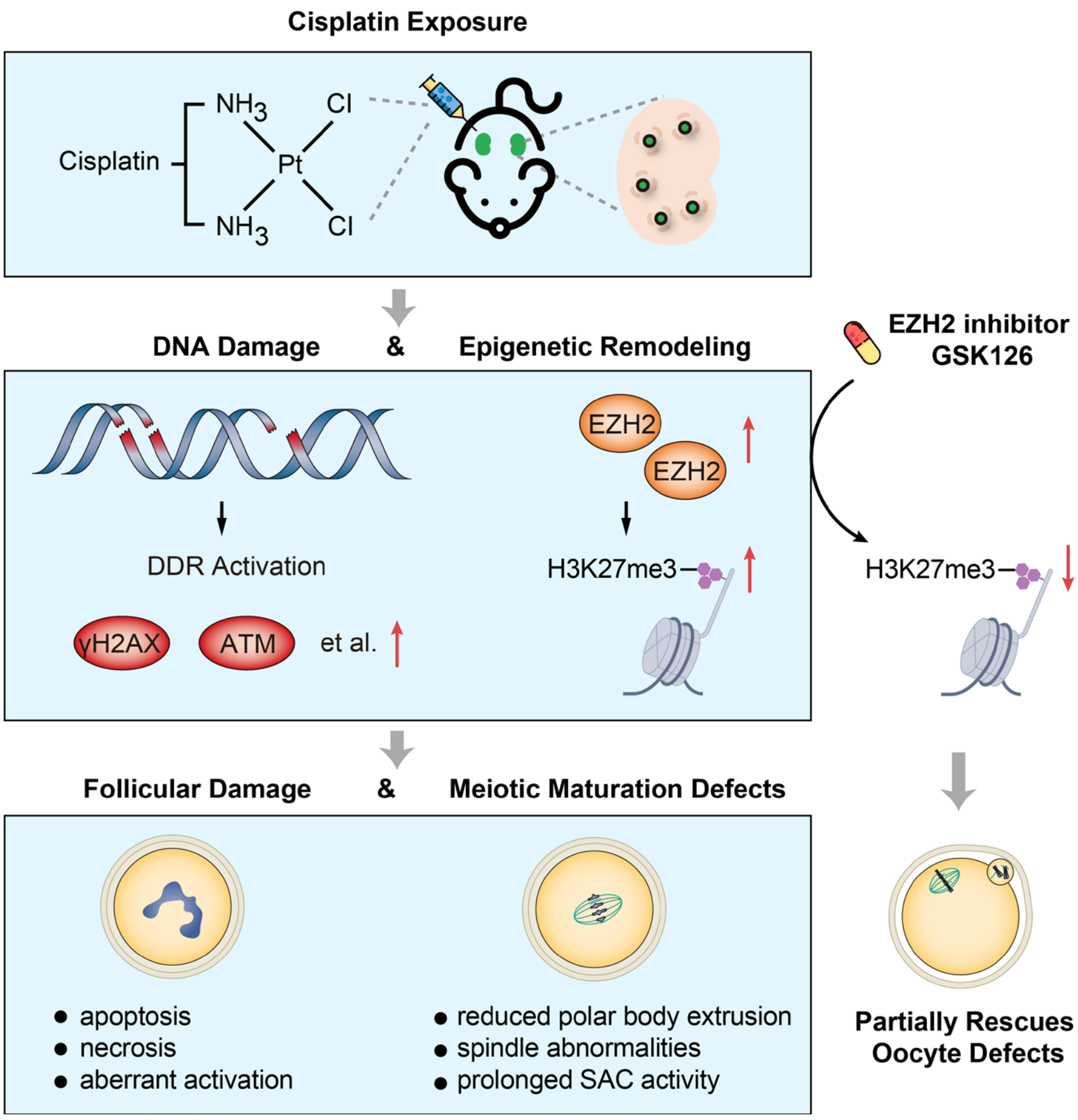

Supplementary Figure 8. Schematic model illustrating oocyte-specific molecular events following cisplatin treatment. Cisplatin induces DNA double-strand breaks in primordial follicle oocytes and activates the DNA damage response (DDR) pathway. Concurrently, cisplatin promotes epigenetic remodeling, characterized by EZH2 accumulation and increased H3K27me3 levels. These alterations lead to follicular damage, including apoptosis and necrosis of primordial follicles, aberrant follicular activation, and meiotic maturation defects such as reduced polar body extrusion, spindle abnormalities, and prolonged spindle assembly checkpoint (SAC) activity. Treatment with the EZH2 inhibitor GSK126 partially rescues these defects.
