# Supplementary material for: In vivo labeling–based proteomic analysis of early follicle oocytes and cisplatin-induced alterations in mice
Source: Nat Commun. 2026 Aug 15;17:9815. doi: 10.1038/s41467-026-76665-3 (PMC13574812; doi:10.1038/s41467-026-76665-3)
Supplement: Supplementary file 4 [file 41467_2026_76665_MOESM2_ESM.pdf]

Title: Supplementary Data 1.

Description: Enriched proteins in the APEX^{oocyte} mice group compared to the control group, representing a primordial follicle–enriched oocyte proteome.

Title: Supplementary Data 2.

Description: Comparison of APEX^{oocyte} -enriched oocyte proteome with prior GV-stage oocyte proteomes.

Title: Supplementary Data 3.

Description: Enriched mRNAs in the APEX^{oocyte} group compared to the *APEX*$^{flox/+}$ group, representing a primordial follicle-enriched oocyte transcriptome.

Title: Supplementary Data 4.

Description: Significant alternative splicing events (ASEs) between primordial follicle oocyte and fully-grown oocyte (FGO) transcriptomes.

Title: Supplementary Data 5.

Description: Cisplatin-induced alterations in the oocyte proteome of APEX^{oocyte} mice.

Title: Supplementary Data 6.

Description: Cisplatin-induced alterations in the oocyte transcriptome of APEX^{oocyte} mice.

Title: Supplementary Data 7.

Description: Antibody information.

Title: Supplementary Data 8.

Description: Peptide Information from *APEX*$^{flox/+}$, APEX^{oocyte}, and cisplatin-treated APEX^{oocyte} groups.

Title: Supplementary Data 9.

Description: Primer sequences.
